# Organic two-dimensional van der Waals heterostructures

**DOI:** 10.1038/s41586-026-11074-6

**Published:** 2026-09-23

**Authors:** Anupam Prasoon, Nguyen Ngan Nguyen, Mike Hambsch, Puja Singhvi, Sophia Terres, Zijie Xiao, Naveen Goyal, Haoyuan Qi, David Mücke, Florian Auras, Zhiyong Wang, Sein Chung, Miroslav Položij, Hai I. Wang, Kilwon Cho, Ute Kaiser, Alexey Chernikov, Mischa Bonn, Stefan C. B. Mannsfeld, Thomas Heine, Xinliang Feng

**Affiliations:** 1 Max Planck Institute for Microstructure Physics, Halle (Saale), Germany; 2 Center for Advancing Electronics Dresden (cfaed) and Faculty of Chemistry and Food Chemistry, Technische Universität Dresden, Dresden, Germany; 3 Faculty of Electrical and Computer Engineering and Center for Advancing Electronics Dresden (cfaed), Technische Universität Dresden, Dresden, Germany; 4 Chair of Theoretical Chemistry, Technische Universität Dresden, Dresden, Germany; 5 Institute of Applied Physics and Würzburg-Dresden Cluster of Excellence ct.qmat, Technische Universität Dresden, Dresden, Germany; 6 Max Planck Institute for Polymer Research, Mainz, Germany; 7 Central Facility for Electron Microscopy, Electron Microscopy of Materials Science, Ulm University, Ulm, Germany; 8 Department of Chemical Engineering, Pohang University of Science and Technology, Pohang, Republic of Korea; 9 Helmholtz-Zentrum Dresden-Rossendorf, Center for Advanced Systems Understanding (CASUS), Görlitz, Germany; 10 Nanophotonics, Debye Institute for Nanomaterials Science, Utrecht University, Utrecht, The Netherlands; 11 Department of Chemistry and IBS Center for Nanomedicine, Yonsei University, Seoul, Republic of Korea

**Keywords:** Conjugated polymers, Electronic materials

## Abstract

Van der Waals heterostructures based on graphene and inorganic two-dimensional (2D) crystals enable exquisite control of interlayer coupling, and emergent electronic and optical phenomena^1,2^. Extending this concept to organic 2D crystals has been hindered by weak, non-directional interlayer interactions that frustrate lattice registry. Here we introduce a bottom-up strategy for programmable lattice engineering in organic van der Waals heterostructures of 2D polymers. Sequential on-water-surface assembly enables layer-by-layer stacking of chemically distinct 2D polymers with defined lattice registry, stacking sequence and thickness, yielding both lattice-matched and controlled lattice-mismatched heterostructures. Structural characterization reveals commensurate epitaxy in lattice-matched and small-mismatched systems, whereas large-mismatch interfaces exhibit moiré features and strain-relief distortions. Ultrafast spectroscopy demonstrates efficient interfacial charge separation and first-principles calculations reveal built-in electric fields and interfacial potential steps arising from interfacial dipole alignment. Devices exhibit diode-like rectification ratios exceeding 10^7^ that systematically decrease with increasing lattice mismatch, establishing organic 2D polymer van der Waals heterostructures as a lattice-engineered platform for (opto)electronic and quantum phenomena.

## Abstract (web summary)

Sequential on-water-surface assembly enables layer-by-layer stacking of two-dimensional polymers to create both lattice-matched and controlled lattice-mismatched van der Waals heterostructures with defined lattice registry, stacking sequence and thickness.

## Main

Since the discovery of graphene^3^, van der Waals (vdW) heterostructures assembled from atomically thin inorganic two-dimensional (2D) materials—including graphene, hexagonal boron nitride and transition-metal dichalcogenides—have emerged as a versatile platform for exploring interlayer-coupled phenomena and enabling functional device applications^1,2,4–12^. Precise control over lattice alignment, stacking order and twist angle has enabled systematic tuning of interlayer hybridization, charge transfer and electronic band structure^13–18^. As a result, vdW heterostructures host emergent electronic, optical and quantum states involving electrons, photons, spins and ions that are fundamentally distinct from the intrinsic properties of isolated individual 2D layers^2,8,11,19–24^.

Despite these advances, vdW heterostructures based on organic 2D crystals (O2DCs) remain largely unexplored. O2DCs—including covalently bonded 2D polymers (2DPs), their multilayer-stacked analogues 2D covalent organic frameworks (2D COFs) and coordination-bonded 2D metal-organic frameworks (2D MOFs)—are *π*-conjugated materials characterized by strong in-plane bonding and rich chemical tunability^25–28^. Their out-of-plane interactions give rise to diverse stacking motifs, which critically govern interlayer coupling and electronic interactions^29–31^. Incorporating this molecular-level design freedom into vdW heterostructures would offer an unprecedented opportunity to programme band alignment, interfacial dipoles and local fields in ways that are difficult to realize in compositionally limited inorganic stacks.

However, fully exploiting this potential requires control knobs that are fundamentally different from those in inorganic systems. In graphene and transition-metal dichalcogenides, interfacial properties are largely set by twist angle and layer sequence at fixed lattice parameters and limited chemistries; these platforms excel at moiré physics and band-structure engineering but offer comparatively little freedom to tune the intrinsic polarity, dipole strength or frontier orbitals of each layer^32–35^. By contrast, O2DCs can, in principle, combine independently designed backbones, functional groups and symmetries within a single heterostructure, enabling simultaneous control over lattice constants and electronic structure at the molecular level^36^—provided that their lattices can be brought into defined registry. Precise lattice matching during stacking is therefore essential to access emergent interfacial phenomena in vdW heterostructures^17,19,20,37–40^. In inorganic systems, commensurate or near-commensurate interfaces are readily obtained by mechanical assembly and twist-angle control^8^. In contrast, O2DCs rely on weak, non-directional dispersive forces between aromatic moieties, electrostatic interactions and low material area density, offering little control over lattice registry^41–43^. Consequently, realizing well-defined, lattice-matched vdW heterostructures at (or near) zero twist remains a major synthetic challenge for 2DPs, 2D COFs and 2D MOFs, and the putative advantages of organic vdW platforms have so far remained largely inaccessible.

Here we introduce a bottom-up route to organic vdW heterostructures in which chemically distinct crystalline 2D polymers are stacked with defined lattice registry and near-zero twist, and where the lattice mismatch between layers (0%, 1.6%, 16.6%, 18.0%) is deliberately programmed at the molecular level. In contrast to our previous surfactant-monolayer-assisted interfacial synthesis studies that focused on single 2D polymer layers^44^ or organic–inorganic hybrids^45^, the present work realizes all-organic 2D–2D heterostructures with tunable lattice mismatch and directly correlates lattice registry with interfacial band alignment and vertical diode behaviour. Using in situ sequential assembly on the surface of water, we achieve layer-by-layer stacking of chemically distinct 2DPs with controlled lattice registry, stacking sequence and thickness. By combining five structurally and electronically distinct 2DPs—polyimine, polyimide, polybenzimidazole, polybenzothiazole and polyboronate ester—we realize 10 vdW heterostructures spanning lattice mismatches from 0% to 18%, including fully lattice-matched (0%), near-matched (1.6%) and strongly mismatched (16.6% and 18.0%) interfaces. Grazing-incidence X-ray diffraction (GIXD) and grazing-incidence wide-angle X-ray scattering (GIWAXS) measurements and high-resolution transmission electron microscopy (HRTEM) reveal epitaxial growth with commensurate alignment and chemically sharp interfaces in all lattice-matched 2DP vdW heterostructures. Optical-pump–terahertz-probe and transient photoluminescence measurements show ultrafast interfacial charge separation and corresponding strong photoluminescence quenching of up to 90%. First-principles calculations indicate built-in electric fields and interfacial potential steps of 0.3–2.1 V arising from dipole alignment at the sharp interface, consistent with vertical transport measurements: diode-like rectification ratios of 10^7^–10^4^ are found for lattice-matched vdW heterostructure devices, decreasing to 10^3^–10^2^ and approximately 10 for small- and large-mismatch systems, respectively. This exceptional rectification performance features rectification ratios exceeding 10^7^, surpassing previously reported values for various oxide-based heterostructure and single-molecule rectifiers (approximately 10^2^–10^5^)^46^. These results establish programmable lattice engineering as a general principle for constructing organic vdW heterostructures, and their interfacial design is a powerful lever for creating functional electronic materials.

## Synthesis of 2D polymer vdW heterostructures

**Fig. 1:**
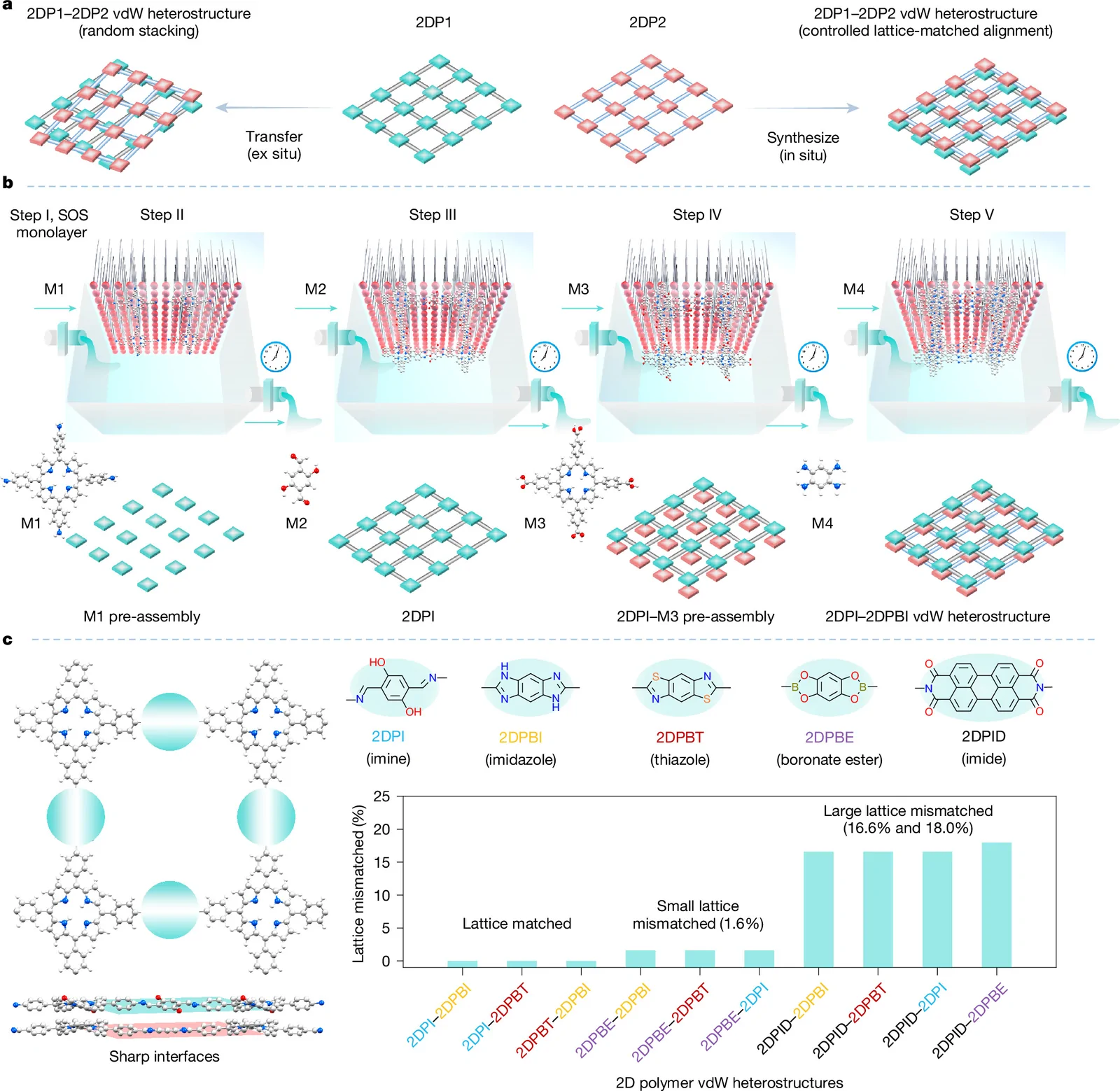
Design and synthesis of lattice-matched and lattice-mismatched 2DP vdW heterostructures with sharp interfaces. **a**, Schematic comparison of ex situ-stacked vdW heterostructures (random orientation) versus in situ-synthesized vdW heterostructures with controlled lattice alignment between two different 2DP layers (2DP1 and 2DP2). **b**, Stepwise synthesis process of vdW heterostructures, demonstrating precise alignment and lattice matching between adjacent layers. **c**, Molecular structures of various 2D polymers: 2DPI, 2DPBI, 2DPBT, 2DPBE and 2DPID, along with their vdW heterostructures exhibiting different lattice matching and lattice mismatching.

Ten distinct 2D polymer vdW heterostructures, encompassing both lattice-matched and mismatched configurations, were synthesized by pairwise combination of five chemically distinct 2DPs—2D polyimine (2DPI), 2D polyimide (2DPID), 2D polybenzimidazole (2DPBI), 2D polybenzothiazole (2DPBT) and 2D polyboronate ester (2DPBE)—via a stepwise (step I to step V) in situ sequential bottom-up assembly on the surface of water^44,47,48^ under ambient conditions (Fig. 1a,b). The lattice-matched 2DPI–2DPBI vdW heterostructure is presented as a representative example; other vdW heterostructures are provided in Supplementary Figs. 1–6. In step I, a charged surfactant monolayer was formed on the water surface by spreading sodium oleyl sulfate (SOS). In step II, an acidic solution of 5,10,15,20-(tetra-4-aminophenyl)porphyrin (M1) was injected into the subphase, resulting in electrostatic adsorption beneath the surfactant monolayer. After adsorption, the subphase was gradually exchanged with fresh Milli-Q water, and ultraviolet–visible spectroscopy was used to ensure complete removal of excess, unadsorbed M1 before subsequent monomer addition. In step III, an aqueous solution of 2,5-dihydroxyterephthalaldehyde (M2) was added to the subphase to initiate diffusion towards the pre-organized M1 layer, and 2D polymerization was carried out under ambient conditions to yield 2DPI; the subphase was then exchanged to remove excess unreacted M2. Brewster angle microscopy on the water surface and optical microscopy after transfer onto a substrate (SiO_2_/Si) revealed continuous, homogeneous coverage of the 2DPI film over large areas (Supplementary Fig. 1). Next, in step IV, a solution of 5,10,15,20-(tetra-4-carboxyphenyl)porphyrin (M3) was injected into the subphase to initiate diffusion towards the underlying 2DPI layer; after adsorption, the subphase was exchanged to remove non-adsorbed M3. In step V, an aqueous solution of 1,2,4,5-benzenetetramine (M4) was added, promoting diffusion towards the 2DPI–M3 interface, and 2D polymerization was carried out at room temperature to synthesize the 2DPI–2DPBI vdW heterostructure. Detailed experimental conditions are provided in Methods and Supplementary Figs. 2–6.

We note that the 2DPI–2DPBI vdW heterostructure comprises few-layer 2DPI and few-layer 2DPBI grown sequentially to form a vertically lattice-matched interface, and the thickness of each layer can be tuned by the reaction time. Typically, for thinner individual layers (<10 nm) within the vdW heterostructure, subphase exchange was performed after each monomer addition step (steps II–V) to remove excess monomers, whereas for thicker individual layers (>10 nm), exchange was limited to steps II and IV to maintain higher monomer concentrations during 2D polymerization (for a detailed discussion, see Supplementary Figs. 2–6). Similarly, by varying the monomer combinations, we synthesized a series of vdW heterostructures, including three commensurate lattice-matched pairs (2DPI–2DPBI, 2DPI–2DPBT and 2DPBT–2DPBI), three small-mismatch (1.6%) configurations (2DPBE–2DPBI, 2DPBE–2DPBT and 2DPBE–2DPI) and four large-mismatch configurations (16.6%: 2DPID–2DPBI, 2DPID–2DPBT and 2DPID–2DPI; 18.0%: 2DPID–2DPBE; Fig. 1c). As a control, individual 2DPI and 2DPBI were synthesized respectively in isolated beakers and subsequently stacked by manual transfer to form a mechanically assembled 2DPI–2DPBI vdW heterostructure (ex situ; Supplementary Fig. 7).

## Assembly mechanism and structural characterization

We therefore group the 2D polymer vdW heterostructures into three representative lattice-mismatch regimes: lattice-matched (0%, exemplified by 2DPI–2DPBI), small-mismatch (1.6%, exemplified by 2DPBI–2DPBE) and large-mismatch (18.0%, exemplified by 2DPID–2DPBE). In the 0% case, grazing-incidence X-ray scattering and electron diffraction reveal a single square-lattice reflection series, whereas for 1.6% and 18.0% mismatch the in-plane reflections split into two sets corresponding to the constituent lattices and, for the largest mismatch, are accompanied by moiré features and strain-relief distortions (Fig. 1c and Supplementary Figs. 8–14). These three regimes provide a structural framework for discussing how lattice registry and mismatch govern interfacial electronic coupling and transport in the following sections.

**Fig. 2:**
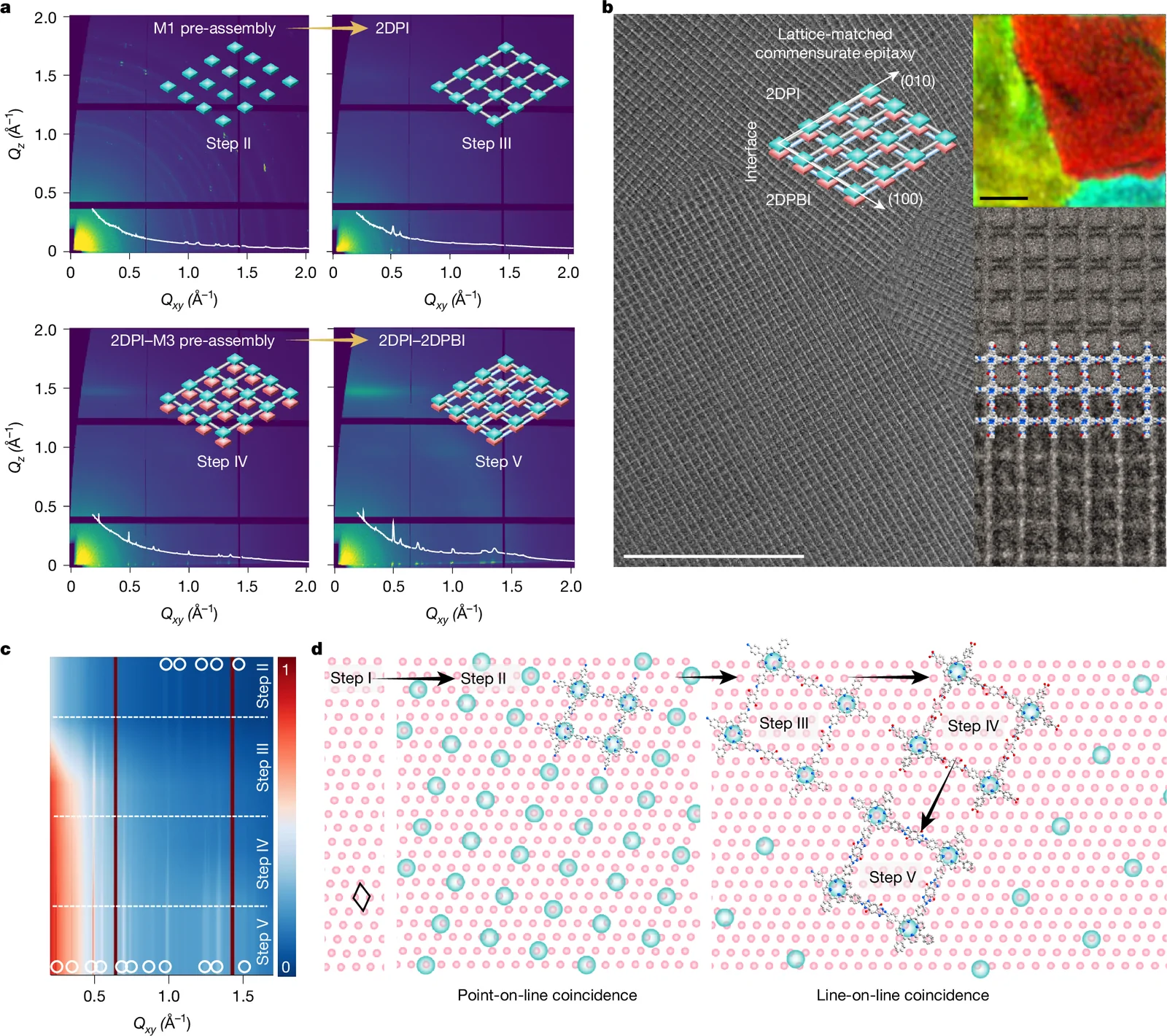
Structural characterization and epitaxial evolution of lattice-matched 2D polymer vdW heterostructures. **a**, Ex situ 2D GIWAXS patterns recorded during the sequential assembly process, illustrating the structural evolution from the M1 pre-assembly (step II) to the formation of 2DPI (step III), followed by the pre-assembly of the 2DPI–M3 intermediate (step IV), and culminating in the formation of the lattice-matched 2DPI–2DPBI vdW heterostructure (step V). The diffraction features reveal the progressive development of long-range crystalline order and preservation of epitaxial alignment throughout the assembly sequence. **b**, Aberration-corrected HRTEM image of the 2DPI–2DPBI vdW heterostructure, showing highly ordered periodic lattice domains with a lattice parameter of approximately 2.5 nm, consistent with the GIWAXS-derived unit cell and image simulations. The inset shows the corresponding structural model together with orientation mapping obtained by Fourier-filter analysis, revealing extended aligned crystalline domains. No pronounced moiré contrast is observed, consistent with the near-zero lattice mismatch between the two stacked 2D polymer layers. Scale bars, 50 nm, 100 nm (inset). **c**, Layer-by-layer GIWAXS analysis of the sequential assembly process, demonstrating the systematic evolution of diffraction intensity and the retention of common in-plane Bragg peak positions throughout heterostructure formation. The colour bar represents the normalized intensity (a.u.). **d**, Schematic illustration of the epitaxial assembly mechanism at the molecular level. The process evolves from point-on-line coincidence during monomer pre-organization to line-on-line epitaxial coincidence after polymerization, enabling aligned stacking and lattice-matched integration of multiple 2D polymer layers.

We monitored the structural evolution of the lattice-matched 2DPI–2DPBI vdW heterostructures by stepwise GIXD, measured directly on the water surface (in situ) and GIWAXS on transferred films (ex situ) after each step (Supplementary Fig. 15). GIXD measurements commenced at step I, corresponding to the formation of the SOS surfactant monolayer, and revealed sharp diffraction peaks consistent with a unit cell described by *a* = 4.5 Å, *b* = 4.5 Å and *γ* = 67.52° (ref. ^47^; Supplementary Fig. 16). In step II, involving the pre-organization of M1 beneath the surfactant monolayer, the ex situ GIWAXS patterns recorded 2 hours after M1 addition to the water subphase exhibited distinct diffraction rings, consistent with a 2D unit cell of *a* = 13.22 Å, *b* = 15.29 Å and *γ* = 85.24° (Fig. 2a and Supplementary Fig. 17). To probe the structural interplay between the crystalline SOS monolayer and the M1 assembly, in situ GIXD measurements focused on the characteristic (100) and (110) Bragg peaks of the surfactant at *Q*_*xy*_ = 1.51 Å^−1^ and *Q*_*xy*_ =1.68 Å^−1^ before and after M1 addition, where *Q*_*xy*_ is the in-plane momentum transfer vector (Supplementary Fig. 18). The (100) peak remained unchanged after 4 hours of diffusion, and the ($\overline{3}$10) diffraction ring of M1 coincided with the surfactant (100) peak, indicating epitaxial growth of the M1 superstructure beneath the surfactant monolayer. Within the experimental accuracy, this epitaxial alignment can be classified as point-on-line epitaxy, in which M1 lattice points register along [1, 0] lattice lines of the SOS surfactant lattice, establishing an energetically favourable one-dimensional registry^49^ (Supplementary Fig. 19). On the basis of the unit-cell areas of SOS (18.7 Å^2^) and M1 (203.0 Å^2^), the areal density ratio of surfactant to M1 molecules (*N*_SOS_/*N*_M1_) is calculated as 203.0 Å^2^/18.7 Å^2^ ≈ 10.86 ≈ 43:4, indicating that 4 M1 molecules interact with approximately 43 surfactant molecules (for a detailed discussion, see Supplementary Fig. 18). The isotropic in-plane GIWAXS rings observed for the M1 pre-assembly correspond to an azimuthally disordered ensemble of small crystalline domains; the schematics in Fig. 2d therefore depict the local point-on-line and line-on-line registry within individual domains rather than a macroscopic single-crystal orientation, and no large-scale chain rearrangement is required upon polymerization (Supplementary Figs. 20 and 21).

In step III, following the addition of M2 to the water subphase, M2 reacts with the pre-assembled M1 layer to undergo 2D polymerization. The GIWAXS profile recorded 12 hours after M2 addition exhibited sharp diffraction peaks corresponding to a square 2D unit cell of *a* = 25.14 Å, *b* = 25.14 Å and *γ* = 90.00° (Fig. 2), indicating lattice expansion relative to the M1 pre-assembly in step II (Supplementary Figs. 21 and 22). The epitaxial alignment can now best be described as line-on-line epitaxy, wherein extended lattice lines of 2DPI along the [6, 3] direction coincide with the [1, 1] crystallographic lines of the SOS surfactant lattice, establishing a form of one-dimensional lattice matching (Fig. 2d and Supplementary Figs. 22–24). Similar epitaxial evolutions were observed using surfactants with different head-group charges (anionic and cationic), highlighting the roles of electrostatic interactions and crystalline monolayer structure in guiding monomer pre-organization on the water surface (Supplementary Figs. 25–27). Next, in step IV, M3, the monomer required for the second 2D polymer (2DPBI), was introduced into the water subphase, resulting in its pre-organization beneath the 2DPI layer. The GIWAXS pattern recorded 2 hours after M3 addition exhibited increased diffraction intensity, whereas the Bragg peak positions in both *Q*_*xy*_ and *Q*_*z*_ remained unchanged, indicating that epitaxial growth proceeded along the pre-established line-on-line registry (Fig. 2b,d). In step V, M4 was added to initiate the 2D polymerization of M3 into 2DPBI, resulting in the formation of the 2DPI–2DPBI vdW heterostructure. Bragg reflections were observed at the same *Q*_*xy*_ and *Q*_*z*_ positions in the GIWAXS diffraction, with further intensity enhancement, confirming that the epitaxial alignment was preserved throughout (Supplementary Figs. 28 and 29). The epitaxial relationship between the 2DPI and 2DPBI layers is commensurate, with coinciding unit-cell parameters and alignment along the (100) and (010) planes in the resulting vdW heterostructure (Supplementary Fig. 29). This structural assignment is further supported by calculated X-ray diffraction patterns, which are in close agreement with the experimental GIWAXS data and confirm lattice matching and epitaxial alignment at the interface (Supplementary Fig. 30).

Aberration-corrected HRTEM images of the lattice-matched 2DPI–2DPBI vdW heterostructures revealed a highly ordered square lattice with a lattice parameter of 2.5 nm, in good agreement with simulated images (Fig. 2b) and the unit cell derived from the GIWAXS measurements. Selected-area electron diffraction (SAED) patterns showed first-order reflections at 0.4 nm^−1^, corresponding to a 2.5-nm lattice parameter, along with higher-order reflections, confirming the high crystallinity of the vdW heterostructures (Supplementary Fig. 31). Orientation-dependent Fourier filtering of the stitched HRTEM image resolved the orientation of individual grains, and no extended amorphous regions were observed within the investigated areas (Fig 2b). The absence of moiré fringes further supports a well-aligned, lattice-matched epitaxial relationship between the 2DPI and 2DPBI layers (Supplementary Fig. 32). In contrast, SAED of the ex situ mechanically assembled 2DPI–2DPBI vdW heterostructure shows multiple sets of discrete reflections at identical scattering radii but different azimuthal angles, indicating lattice-matched layers (both exhibit the same reciprocal-lattice vector magnitude, |**g**| ≈ 0.4 nm^−1^) with random in-plane rotational registry (Supplementary Fig. 33). Furthermore, for the small-mismatch 1.6% 2DPBI–2DPBE vdW heterostructure, GIWAXS resolves two closely spaced in-plane reflection series, with (100) peaks at *Q*_*xy*_ = 0.251 and *Q*_*xy*_ = 0.255 Å^−1^, corresponding to lattice parameters of *a* = *b* = 2.50 nm for 2DPBI and *a* = *b* = 2.46 nm for 2DPBE, respectively. SAED further shows closely spaced two superimposed diffraction lattices corresponding to both lattice periodicities, and HRTEM confirms continuous ordered crystalline domains without warping of the 2D polymer layers. For the large-mismatch 18.0% 2DPID–2DPBE vdW heterostructure, GIWAXS shows two clearly separated in-plane reflections at *Q*_*xy*_ ≈ 0.20 and *Q*_*xy*_ = 0.25 Å^−1^, assigned to 2DPID and 2DPBE, respectively. In addition, SAED shows two sets of diffraction spots from the constituent 2D polymer lattices, corresponding to lattice parameters of *a* = *b* = 3.00 nm for 2DPID and *a* = *b* = 2.46 nm for 2DPBE, along with distinct moiré reflections. Large-area HRTEM images show different crystalline domains with moiré contrast, and higher-magnification HRTEM reveals wrinkling and warping and long-range edge dislocations. Together, these reciprocal-space and real-space observations suggest that strain relaxation in the large-mismatch heterostructure occurs through a combination of local lattice distortion, dislocation formation and out-of-plane deformation (Supplementary Figs. 8–14).

Atomic force microscopy (AFM) measurements showed uniform, continuous films with a thickness of approximately 10.2 nm for the lattice-matched 2DPI–2DPBI vdW heterostructures (Supplementary Fig. 41). The progressive increase in thickness over time reflects controlled layer growth, which could be readily tuned from 4.6 nm to 30 nm by adjusting the monomer adsorption time during in situ assembly (Supplementary Fig. 34). Depth-profile X-ray photoelectron spectroscopy confirmed the formation of a well-defined interface between the 2DPI and 2DPBI layers. Full X-ray photoelectron spectroscopy survey spectra showed a sharp chemical transition during etching, with a strong decrease of the O 1*s* signal from the oxygen-containing 2DPI layer to the oxygen-free 2DPBI layer. Consistently, high-resolution N 1*s* spectra showed a corresponding transition from imine nitrogen in 2DPI to 1,3-diazole nitrogen in 2DPBI, with peaks centred at 399.71 eV and 400.01 eV, respectively (Supplementary Fig. 35). Angle-dependent near-edge X-ray absorption fine-structure spectroscopy further confirmed the formation and ordered stacking of the lattice-matched 2DPI–2DPBI vdW heterostructure. Distinct π* and σ* transitions from both 2DPI and 2DPBI were observed at 286 eV and 290 eV, respectively. The angular dependence of the spectral intensity revealed an in-plane orientation of the vdW heterostructure (Supplementary Fig. 36).

## Interfacial electronic coupling

**Fig. 3:**
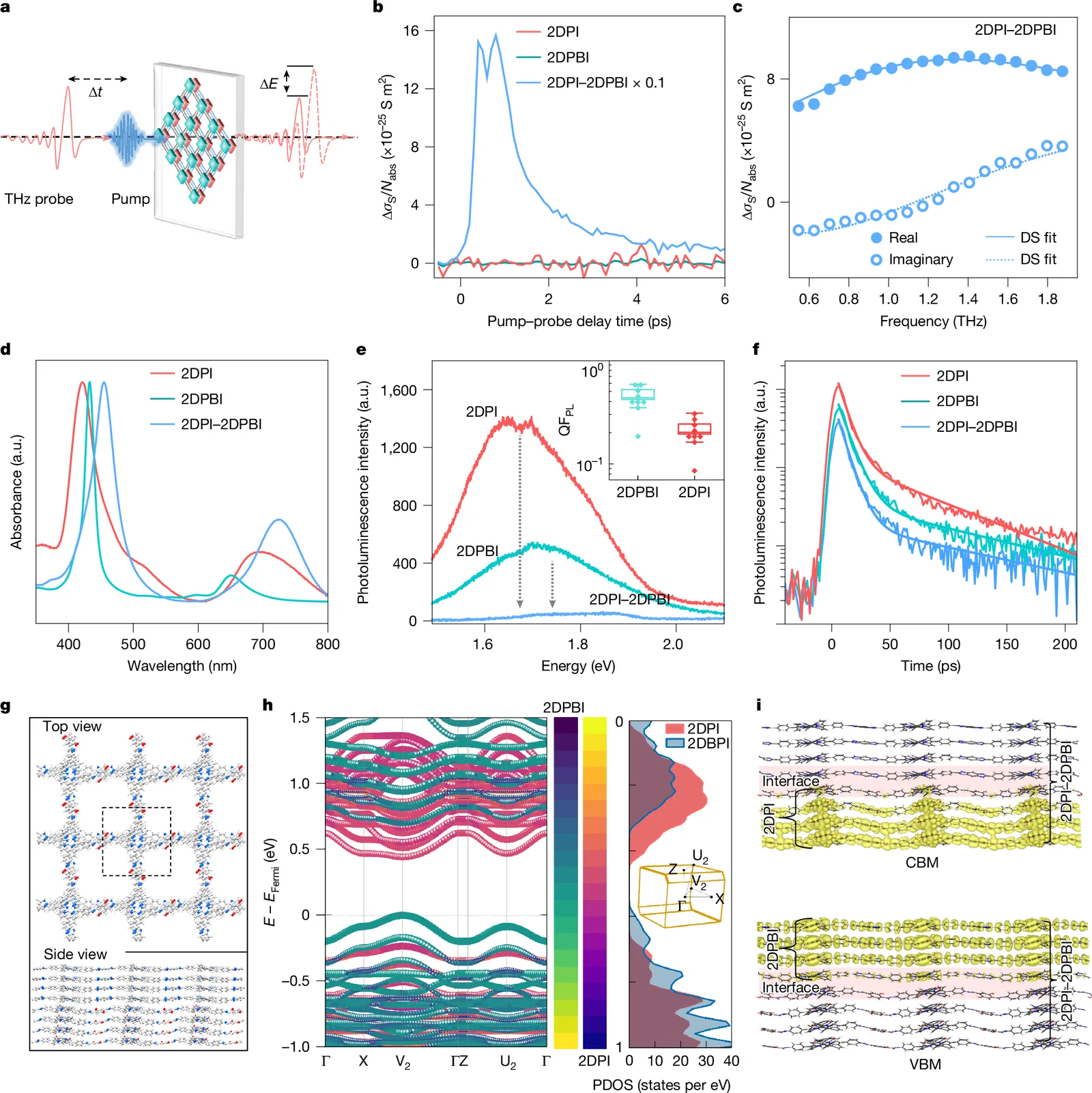
Interfacial charge dynamics of lattice-matched 2D polymer vdW heterostructures. **a**, Schematic of optical-pump–terahertz-probe spectroscopy used to investigate ultrafast charge transport across the interface. An optical-pump pulse (in blue) photoexcites charge carriers in the sample, and their subsequent dynamics are probed by a terahertz (THz) pulse with a controllable time delay (Δ*t*). The presence of free carriers leads to absorption of the THz radiation, resulting in attenuation of the transmitted THz electric field (Δ*E*) proportional to the sheet photoconductivity Δ*σ*_S_. **b**, Time-resolved Δ*σ*_S_ per absorbed photon density *N*_abs_ for 2DPI, 2DPBI and their heterostructure 2DPI–2DPBI. **c**, Frequency-resolved THz photoconductivity of the 2DPI–2DPBI heterostructure thin film recorded approximately 1 ps after optical excitation, along with the corresponding fit using the Drude–Smith (DS) model. **d**, Ultraviolet–visible absorption spectra of 2DPI, 2DPBI and their vdW heterostructure (2DPI–2DPBI), showing distinct excitonic features from each component. **e**, Representative photoluminescence spectra of both the single polymers and the heterostructure measured in high vacuum, at room temperature, for the excitation fluence of 6.6 µJ cm^−2^ using 140-fs pulses with photon energy of 2.82 eV. The arrows indicate the decrease of the photoluminescence intensity in the heterostructure with respect to the two constituents. Inset: quenching factor (QF_PL_) of the heterostructure photoluminescence intensity with respect to the single polymer photoluminescence. The error bars are standard deviations of the QFs. **f**, Corresponding photoluminescence transients, demonstrating rapid decay on a tens of picoseconds timescale, followed by slower dynamics over more than 100 ps. Solid lines represent biexponential fit curves. **g**, Density-functional-theory-optimized structural models of the 2DPI–2DPBI vdW heterostructure (top and side views), illustrating the commensurate lattice matching and epitaxial stacking geometry at the interface. **h**, Band structure (left) and projected density of states (PDOS; right) with Brillouin zone (inset). **i**, Charge-density localization maps at the conduction-band minimum (CBM) and valence-band maximum (VBM), illustrating interfacial delocalization and charge separation within the heterostructure with an isosurface value of 1.6 × 10^−3^.

We investigated the interfacial electronic coupling and charge-transfer dynamics in the 2DPI–2DPBI vdW heterostructure using optical-pump–terahertz-probe (Fig. 3a) spectroscopy (Methods). Optical excitation was performed with 400-nm pulses of approximately 50 fs duration, promoting charge carriers from the valence band to the conduction band. The high-frequency conductivity of these photogenerated carriers was subsequently measured using terahertz pulses with a bandwidth of up to 2 THz. Figure 3b presents a sizeable, rapidly (approximately 1 ps) decaying photoconductivity in the vdW heterostructure, yet no detectable signal in the individual 2DPI and 2DPBI layers. In contrast, time-resolved photoluminescence measurements (Fig. 3e,f) show clear emission and decay dynamics up to approximately 200 ps, with substantially reduced photoluminescence for the heterostructure. The absence of photoconductivity and strong photoluminescence in the individual layers is consistent with the ultrafast formation of highly localized excitonic states, which exhibit negligible conductivity and polarizability in the terahertz frequency. In contrast, the finite photoconductivity in 2DPI–2DPBI vdW heterostructure testifies to the generation of free carriers resulting from efficient charge separation across the interface, with electrons localizing in 2DPI and holes accumulating in 2DPBI (see simulated interfacial energetics in later section).

We then conducted time-domain terahertz spectroscopic analysis near the peak photoconductivity of the 2DPI–2DPBI vdW heterostructure. As shown in Fig. 3c, the vdW heterostructure showed a free-carrier response characterized by a complex photoconductivity dominated by its real component. Assuming comparable scattering for electrons in 2DPI and holes in 2DPBI, spectral fitting using the Drude–Smith model yielded a momentum scattering time of approximately 100 fs for charge carriers in the 2DPI–2DPBI vdW heterostructure. Similar charge separation effects were also observed in other lattice-matched vdW heterostructures (2DPI–2DPBT and 2DPBT–2DPBI), highlighting the generality of efficient interfacial charge delocalization facilitated by coherent lattice alignment (Supplementary Fig. 37). Ultraviolet–visible absorption spectra of the 2DPI–2DPBI vdW heterostructure retained the characteristic porphyrin Soret- and *Q*-band features of both 2DPI and 2DPBI, with enhanced absorption in the higher-wavelength region (Fig. 3d and Supplementary Fig. 38). Room-temperature photoluminescence (Fig. 3e) spectra under pulsed excitation at 2.82 eV show significant quenching, up to 90%, of the photoluminescence intensity in the 2DPI–2DPBI vdW heterostructure compared with the individual 2DPI and 2DPBI layers. This substantial quenching is consistent with highly efficient charge separation at the heterostructure interface following optical excitations. Transient photoluminescence measurements reveal similar decay dynamics for both the individual 2DPI and 2DPBI layers and the 2DPI–2DPBI vdW heterostructure, characterized by an initial rapid recombination within approximately 10 ps, followed by a slower decay extending up to 100 ps (Fig. 3f). Furthermore, spatial photoluminescence mapping over a 40 × 40 μm^2^ area shows uniform emission from the 2DPI–2DPBI vdW heterostructure, with an energy at the photoluminescence maximum of *E*_max_ = 1.700 ± 0.004 eV consistently obtained across the mapped region (Supplementary Fig. 39).

## Interfacial band alignment and dipole formation

**Fig. 4:**
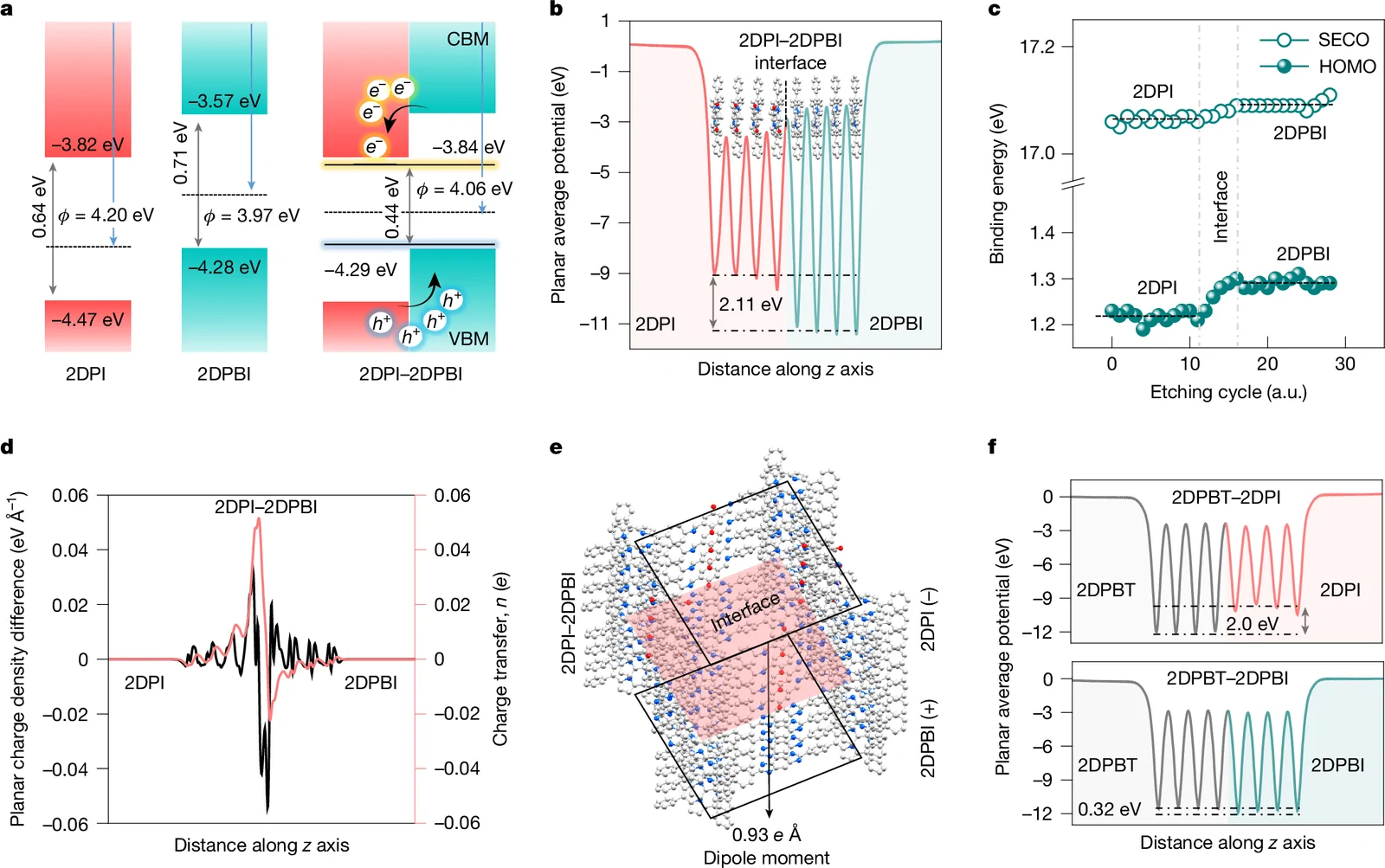
Band alignment in 2D polymer heterostructures. **a**, Energy-level diagrams of individual 2DPI and 2DPBI polymers and their heterostructure (2DPI–2DPBI), showing vacuum-level shifts, work-function changes and formation of type-II band alignment promoting charge separation. *ϕ* denotes the work function **b**, Planar-averaged electrostatic potential along the stacking direction (*z* axis) of the 2DPI–2DPBI heterostructure, indicating a built-in potential drop of 2.11 eV across the interface. **c**, Ultraviolet photoelectron spectroscopy depth profiling showing shifts in the secondary electron cut-off (SECO) and highest occupied molecular orbital (HOMO) levels across the interface. **d**, Planar charge-density difference and integrated charge-transfer profile reveal strong interfacial polarization and electron redistribution. **e**, 2DPI–2DPBI heterostructure exhibits a built-in dipole of 0.93 *e* Å. **f**, Comparison of potential profiles for different heterostructures (2DPBT–2DPI and 2DPBT–2DPBI), showing varying interface potential drops (2.0 eV and 0.32 eV), underscoring the tunability of interfacial properties through lattice-matched polymer combinations.

The 2DPI–2DPBI heterostructure forms a vdW interface, where two chemically distinct 2D polymers are stacked (Fig. 3g–i). We calculated that the 2DPI–2DPBI vdW heterostructure has a direct bandgap of 0.45 eV, smaller than those of isolated 2DPI (0.64 eV) and 2DPBI (0.71 eV). This reduction indicates strong electronic coupling across the interface, arising from a stacking-dependent proximity effect between the two layers^50^, despite the absence of directional interlayer bonding (Fig. 4a–c). The valence-band maximum is mainly localized on 2DPBI, whereas the conduction-band minimum is on 2DPI, forming a type-II band alignment (Fig. 3h). This configuration naturally promotes charge separation: electrons move to the lower-energy conduction band of 2DPI, whereas holes stay in the higher-energy valence band of 2DPBI. These same trends can be seen for the other lattice-matched 2DPI–2DPBT and 2DPBT–2DPBI heterostructures (Supplementary Figs. 40–52).

The calculated work functions of the individual layers are 4.20 eV for 2DPI and 3.97 eV for 2DPBI, giving a work-function offset of 0.23 eV. When the two layers come into contact, Fermi-level equilibration occurs. This results in a work function of 4.06 eV for the 2DPI–2DPBI vdW heterostructure, with the conduction- and valence-band edges positioned at −3.84 eV and −4.29 eV relative to vacuum, respectively (Fig. 4a). The 0.23-eV offset drives electrons from 2DPBI (lower work function) to 2DPI (higher work function), creating an internal electric field directed from 2DPBI towards 2DPI (Fig. 4b). This built-in field causes downwards band bending in 2DPI and upwards band bending in 2DPBI. Charge-density difference analysis further clarifies the microscopic picture. Electrons are depleted from 2DPBI and accumulated on 2DPI, with an estimated net transfer of about 0.05 *e* per unit cell (Fig. 4d). This redistribution produces an interfacial dipole moment of 0.93 *e* Å, oriented perpendicular to the interface (Fig. 4e). Normalized by the unit-cell area, this corresponds to a surface dipole density of 1.45 × 10^−3^ *e* Å^−1^. In addition, the planar-averaged electrostatic potential shows a built-in potential step of approximately 2.11 eV across the interface (Fig. 4b). Therefore, from the bulk work function, the interfacial dipole moment and the built-in potential step, we conclude that the surface potential differs on the two faces of the heterostructure, which is a direct physical consequence of the interfacial dipole. The work-function offset is even larger for the 2DPBT containing heterostructures, up to 0.5 eV (Supplementary Figs. 38–48), but owing to band alignment, their built-in potential steps are lower; 2.0 eV and 0.32 eV for 2DPI–2DPBT and 2DPBT–2DPBI, respectively (Fig. 4f). Depth-profile ultraviolet photoelectron spectroscopy measurements support this picture, revealing clear shifts in both the highest occupied molecular orbital onset and the secondary electron cut-off after 2DPI–2DPBI vdW heterostructure formation (Fig. 4c). This intrinsic electrostatic asymmetry not only drives the charge separation shown in the ultrafast laser spectroscopy but also could give rise to rectifying behaviour and provide the energetic basis for diode-like charge transport, without requiring external doping or defect engineering.

## Out-of-plane electrical transport

**Fig. 5:**
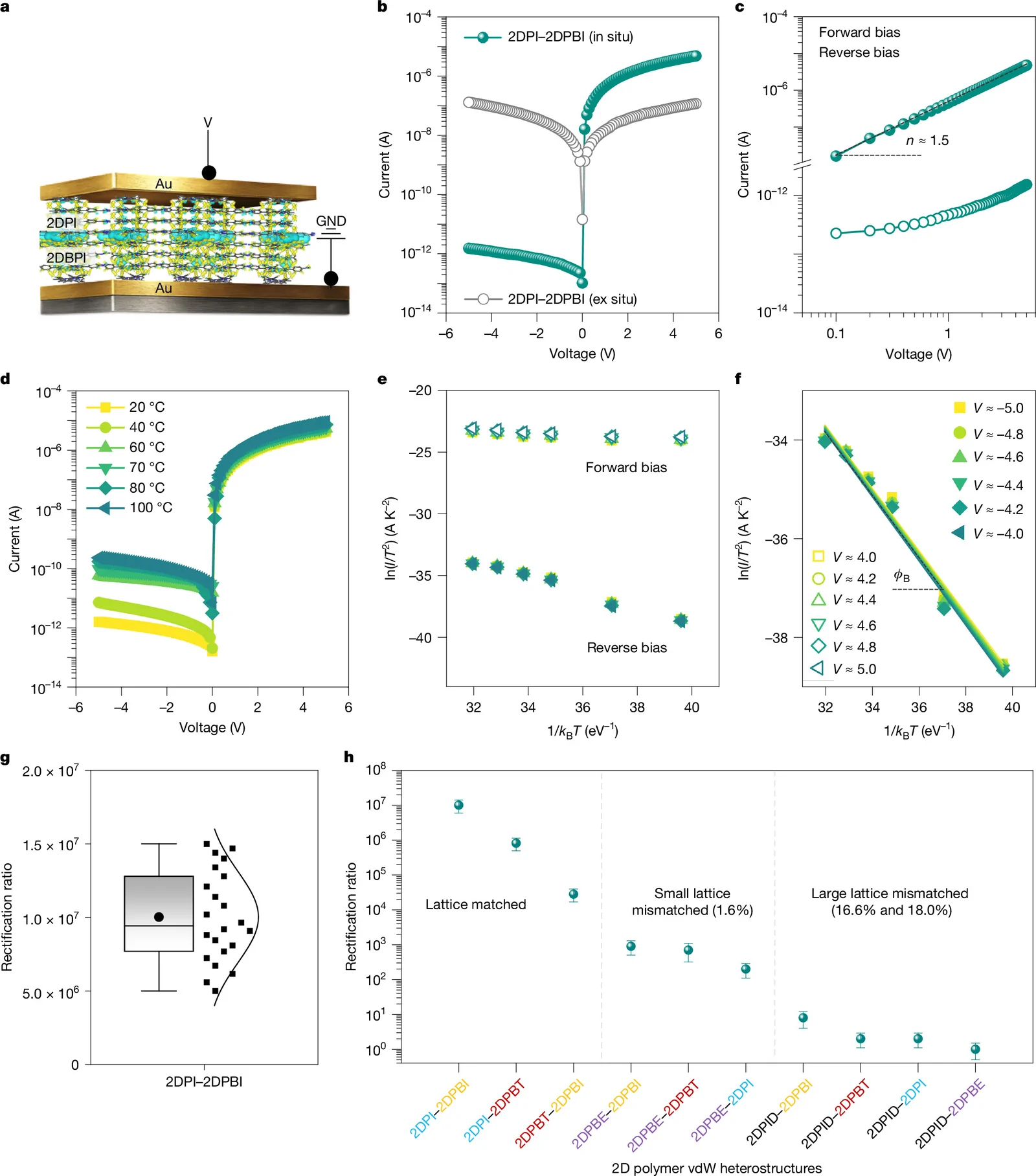
Vertical charge transport and diode-like behaviour in 2DP vdW heterostructures. **a**, Schematic of vertical heterojunction device architecture comprising stacked 2DPI and 2DPBI layers sandwiched between gold electrodes. **b**, *I*–*V* characteristics of 2DPI–2DPBI vdW heterostructures prepared via in situ versus ex situ methods (mechanical transfer), demonstrating higher current and rectification in the in situ configuration. **c**, *I*–*V* data showing power-law dependence with slope *n* ≈ 1.5. **d**, *I*–*V* curves of the 2DPI–2DPBI vdW heterostructures with varying measurement temperatures. **e**, Plots of ln(*I*/*T*^2^) versus 1/(*k*_B_*T*) showing the clear Schottky barrier height of the holes in the reverse bias regime. **f**, Richardson plots at various reverse bias voltages used to extract the effective Schottky barrier height. **g**, Statistical distribution (*n*, over 22 devices) of the rectification ratio for the lattice-matched 2DPI–2DPBI vdW heterostructure. **h**, Rectification-ratio summary of 2D polymer vdW heterostructures grouped by lattice mismatch, showing that rectification decreases with increasing lattice mismatch.

Theoretical analysis of the band alignment, built-in dipole and interfacial potential step in the lattice-matched 2DPI–2DPBI vdW heterostructure predicts rectifying behaviour. To investigate this emergent interfacial phenomenon, we studied vertical charge transport in the 2DPI–2DPBI vdW heterostructure using micro-devices and conductive scanning probe microscopy. The device comprised a 2DPI–2DPBI vdW heterostructure sandwiched between a bottom ground electrode and a top gold electrode (Fig. 5a). Fabricated devices exhibited exceptional rectification performance, with rectification ratios exceeding 10^7^ (Fig. 5b), surpassing previously reported values for various oxide-based heterostructure and single-molecule rectifiers (~10^2^–10^5^)^46^. Statistical measurements of vertical micro-devices, together with nanoscale conductive AFM, supported diode-like behaviour, with local variations attributed to grain boundaries and structural defects (Fig. 5g and Supplementary Figs. 53–57). Current–voltage (*I*–*V*) measurements of the 2DPI–2DPBI lattice-matched vdW heterostructure revealed distinct charge transport mechanisms under forward and reverse bias. Under forward bias, the current followed a power-law dependence on voltage (*I* ∝ *V*^3/2^), characteristic of Child–Langmuir’s law conduction^51^. In Child–Langmuir conduction, the ballistic transport of charge carriers between two electrodes proceeds without scattering or trapping by extrinsic molecules or defect states. Under reverse bias, the current remained strongly suppressed (*I* ∝ *V*^1/2^), clearly indicating that charge conduction was constrained by injection-limited or bulk-limited processes. In contrast, analogous heterostructures fabricated via ex situ mechanical transfer showed nearly symmetric transport without any substantial rectification, as random rotational registry disturbed coherent interfacial orbital overlap, resulting in the absence of a coherent built-in dipole (Fig. 5b; see Supplementary Figs. 53–57 for details). For the in situ-grown 2DPI–2DPBI vdW heterostructure, temperature-dependent *I*–*V* measurements show a clear increase in reverse current with increasing temperature, consistent with thermally activated conduction dominated by thermionic emission^52^ (Fig. 5c–f). The ln(*I*/*T*^2^) versus 1/*k*_B_*T* plot followed the thermionic emission model, where *k*_B_ is the Boltzmann constant (8.617 × 10^−5^ eV K^−1^), *T* is the temperature in kelvin (K), and *e* is the elementary charge:

$$I\propto {T}^{2}\exp \left(-\frac{e{\varPhi }_{{\rm{B}}}}{{k}_{{\rm{B}}}T}\right)$$

Thermionic emission describes carrier transport in which thermal energy enables charge carriers to overcome the Schottky barrier (*Φ*_B_) at the electrode–semiconductor interface. The Schottky barrier height *Φ*_B_ was extracted from the slope of the ln(*I*/*T*^2^) versus 1/*k*_B_*T* plot^53^. In the reverse bias range of −4 V to −5 V, the extracted *Φ*_B_ consistently remained around 0.65 eV, confirming the dominance of thermionic emission in this regime. This barrier height accounted for the extremely low off-current observed under reverse bias. In contrast, the forward current exhibited negligible temperature dependence, indicating the absence of a significant injection barrier. When the 2DPI layer was connected to the positive terminal, minimal injection barriers enabled efficient hole transport across the 2DPI–2DPBI lattice-matched vdW heterostructure via Child–Langmuir conduction. Conversely, under reverse bias, with the 2DPBI layer connected to the positive terminal, the approximately 0.65-eV injection barrier inhibited hole injection. As a result, only a limited number of holes reached the opposite terminal, leading to the observed low off-current. Across all lattice-matched heterostructures, the rectification ratio scaled systematically with the interfacial potential step (Supplementary Figs. 53 and 54). Rectification ratios ranged from 10^7^ to 10^4^ for lattice-matched structures, decreased to 10^3^–10^2^ for small-mismatch configurations, and approached only approximately 10 for large-mismatch heterostructures (Supplementary Fig. 57). This mismatch-dependent trend indicates that defined lattice registry is essential for maintaining coherent interfacial orbital overlap and built-in dipole formation, thereby governing diode-like rectification (Fig. 5h).

## Conclusion

We achieved bottom-up in situ synthesis of 2DP vdW heterostructures with controlled lattice-matching and -mismatching configurations. Epitaxial alignment in lattice-matched systems enabled coherent interfaces with built-in dipoles and sharp potential steps, resulting in efficient charge separation, photoluminescence quenching and ultrafast carrier dynamics. The strong interfacial electric fields and type-II band alignment had a pivotal role in driving spatial charge separation and directional charge transport across the interface. Lattice-matched vdW heterostructures exhibited diode-like rectification with on/off ratios exceeding 10^7^, governed by space-charge-limited conduction in forward bias and thermionic emission in reverse bias. The rectification ratio scaled with the interfacial potential difference, increasing from approximately 10^4^ to 10^7^ as the potential step height increased from 0.3 V to 2.1 V. These findings establish a synthetic platform for accessing quantum and electronic functionalities in 2DP vdW heterostructures via interfacial design, offering opportunities to explore emergent physical phenomena, including moiré superlattices and interfacial spin physics.

## Methods

### Computational

All geometries were first optimized using the self-consistent-charge density-functional-based tight-binding (SCC-DFTB)^54^ method as implemented in the Amsterdam Modelling Suite (AMS, ADF 2019)^55,56^. The 3ob-3-1^57^ Slater–Koster parameter set was used, and long-range dispersion forces were included using universal-force-field corrections to accurately capture vdW interactions. Bulk structures of the parent 2D polymers (2DPI, 2DPBI and 2DPBT) were modelled as four-layer stacks per unit cell, whereas the heterostructures were constructed as eclipsed 4 + 4 layer stacks to mimic experimentally observed lattice matching. Subsequent density functional theory (DFT) was carried out using the Vienna Ab initio Simulation Package (VASP, version 5.4.4)^58,59^. The exchange-correlation potential was described using the generalized gradient approximation (GGA) with the Perdew–Burke–Ernzerhof (PBE)^60^ functional whereas the core–electron interactions were treated using the projector augmented-wave method^61^. A plane-wave cut-off energy of 400 eV was adopted throughout. VdW interactions were treated using Grimme’s DFT-D3 scheme to ensure accurate description of interlayer forces. Energy and force convergence criteria were set to 10^−5^ eV and 0.01 eV Å^−1^, respectively. All calculations were performed on AA-stacked slab models with dipole corrections applied to account for the asymmetric slab geometry. The Brillouin zone was sampled using a Γ-centred 2 × 2 × 1 *k*-point mesh for both pristine layers and corresponding heterostructures. A vacuum spacing of 35 Å was applied along the out-of-plane (*z*) direction for parent 2DPs and 70 Å for heterostructures to suppress spurious periodic interactions. The work function of each system was calculated as *φ* = *V*_vacuum_ − *E*_Fermi_, where *V*_vacuum_ is the vacuum potential extracted from the plateau region of the planar-averaged electrostatic potential (LOCPOT) and *E*_Fermi_ is the Fermi energy from OUTCAR. The dipole correction (LDIPOL = T, DIPOL = 0.5 0.5 0.5) was applied in the ground-state calculation, and the interfacial dipole moment was subsequently determined from a separate single-point calculation, oriented normal to the plane of the layers. Band-structure calculations were performed at the same computational level as single-point energy evaluations. Projection operators were computed in reciprocal space using Gaussian smearing with a width of 0.05 eV; no additional augmentation grids were required. All structural models and charge-density isosurfaces were visualized using the VESTA software package^62^.

### In situ on-water-surface GIXD

The in situ GIXD measurements were performed at beamline ID10-SURF at ESRF, Grenoble, France. The energy of the beam was 8 keV and the spot size was 30 μm × 30 μm. The measured films were grown on the air–water interface in a rectangular polytetrafluoroethylene trough, which was placed in a helium-filled enclosure with Kapton windows to reduce air scattering and beam damage to the film. A Dectris Mythen 2 strip detector with Soller slits was scanned along the in-plane, horizontal angle (2*θ*) to record images (exposure time 5 s). The individual images were horizontally integrated to obtain the vertical intensity distribution (*I*(*Q*_*z*_)) at each 2*θ* angle. These one-dimensional spectra were then combined to create a 2D *Q*_*xy*_–*Q*_*z*_ intensity map that was analysed using WxDiff.

### Ex situ thin-film GIWAXS

The GIWAXS measurements of transferred films of lattice-matched heterostructures were performed at beamline ID10-SURF at ESRF, Grenoble, France. The energy of the beam was 22.5 keV and the beam had dimensions of 16 µm (vertically) × 26 µm (horizontally). The detector for recording images was a Dectris Eiger 2X 4M, which was placed 551 mm behind the sample. The sample-to-detector distance and beam centre on the detector were verified using a lanthanum hexaboride standard. The incidence angle of the beam was 0.06° and the samples were exposed to the beam for 30–60 s depending on the intensity of the scattering signal. The measured scattering images were corrected and analysed using WxDiff.

The GIWAXS measurements of transferred films of lattice-mismatched heterostructures were also performed at beamline ID10-SURF at ESRF, Grenoble, France. The energy of the beam was 22.5 keV and the beam had dimensions of 10 µm (vertically) × 60 µm (horizontally). The detector for recording images was a Dectris Eiger 2X 4M, which was placed 352 mm behind the sample. The sample-to-detector distance and beam centre on the detector were verified using a lanthanum hexaboride standard. The incidence angle of the beam was 0.04° and the samples were exposed to the beam for 30 s. The measured scattering images were corrected and analysed using WxDiff.

### Optical-pump–terahertz-probe measurements

Ultrafast carrier dynamics were investigated using an optical-pump–terahertz-probe system driven by a 1-kHz titanium:sapphire laser (Spitfire Ace, 50 fs pulse duration). Broadband terahertz pulses (bandwidth approximately 2.5 THz) were generated and detected using 1-mm ZnTe crystals via optical rectification and electro-optic sampling, respectively. Time-resolved terahertz photoconductivity was obtained by monitoring pump-induced changes in the peak terahertz electric field as a function of the pump–probe delay. All measurements were performed at room temperature under a dry nitrogen atmosphere.

### Transient photoluminescence microscopy and photoluminescence mapping

The heterostructure as well as the single polymer layer samples were placed in a micro-cryostat mounted on a motorized *x*–*y* stage for measurements in high vacuum conditions (<10^−4^ mbar). Transient photoluminescence microscopy measurements at room temperature were carried out using an 80-MHz pulsed titanium:sapphire laser (Chameleon Ultra II, Coherent, pulse width of 140 fs), frequency-doubled in a second-harmonic generator (HarmoniXX SHG, A.P.E.) to obtain an excitation photon energy of 2.82 eV. The laser was focused to a spot size of about 0.5 µm on the sample using a glass-corrected ×60 microscope objective, yielding excitation fluences of 6.6–88 µJ cm^−2^ per pulse. Residual reflected or scattered laser light was spectrally suppressed using hard-coated edgepass filters (ThorLabs). The signal was then focused onto the slit of an imaging spectrometer (Acton SpectraPro SO-2300, Princeton Instruments). A charge-coupled device camera (Pixis256, Roper Scientific) and a streak camera (C10910, Hamamatsu Photonics) were used for time-integrated and time-resolved measurements, respectively.

Photoluminescence mapping was performed on the same set-up at 2.82 eV excitation photon energy and a pump fluence of 88 µJ cm^−2^ per pulse at room temperature under vacuum. The motorized* x*–*y* stage was used to acquire photoluminescence spectra on a spatial grid with micrometre step size. In the resulting maps, each pixel corresponds to an individual photoluminescence spectrum from which the relevant peak parameters were extracted. The main emission feature was fitted using a Gaussian fit function to extract the energy of the peak maximum and the spectrally integrated photoluminescence intensity.

### HRTEM imaging and image simulation

Experiments were carried out using an image-side spherical aberration-corrected FEI Titan 80–300 microscope operated at 300 kV. The microscope was equipped with a CEOS hexapole spherical aberration coefficient corrector, enabling correction of geometrical axial aberrations up to the third order. Images were recorded using a Gatan UltraScan 1000 charge-coupled device camera. HRTEM image simulations were performed with the abTEM package using Quantum ESPRESSO plane-wave DFT calculations based on the GGA-PBE-relaxed multilayer structure.

### General characterization

Optical microscopy (Zeiss), AFM (Bruker Multimode 8 HR) and TEM (Zeiss, Libra 200 KV) were used to investigate the morphology and structure of the samples. Thin films were deposited on a silicon substrate for scanning electron microscopy measurements and on copper grids for TEM measurements. All the optical microscopy and AFM images were recorded on a 300-nm SiO_2_/Si substrate. Ultraviolet–visible absorption spectra were recorded on a ultraviolet–visible–near-infrared spectrophotometer Cary 5000 device at room temperature on a quartz glass substrate. Photoluminescence spectra were measured on the PerkinElmer fluorescence spectrometer LS 55. Attenuated total reflectance Fourier-transform infrared spectroscopy was performed on a Tensor II system (Bruker) with an attenuated total reflection unit, and the samples were prepared by depositing the thin films on a copper foil. Time-dependent surface-pressure measurements were carried out by the Langmuir–Blodgett trough (KSV NIMA). The trough was equipped with a platinum Wilhelmy plate, a Teflon dipper and a pair of Delrin barriers. Thermal annealing was carried out before device measurements. Specifically, the devices were annealed under vacuum at 80 °C for 6 h before electrical characterization. We conducted electrical characterization of the 2DPI–2DPBI vdW heterostructures utilizing conductive AFM equipped with cantilevered solid platinum tips (25Pt300B-10, Park Systems). According to the manufacturer, these tips have a nominal apex radius of 10 nm. For sample preparation, the heterostructure layers were mechanically and wet-transferred onto a 100-nm Cr–Au/n^++^ Si substrate. This substrate was subsequently mounted onto a stainless-steel AFM chuck using silver paste, which was also applied to the sample corners to ensure robust electrical contact between the substrate and the chuck. To establish consistent mechanical contact while preventing physical degradation of the 2DPI–2DPBI vdW heterostructure film, the tip pressing force was maintained between 400 nN and 550 nN; the sample surface was concurrently monitored for damage using an integrated optical microscope. Measurements were performed point-by-point by applying a direct-current bias voltage between the AFM cantilever and the stainless-steel chuck to collect the resulting current. All solvents, reagents and chemicals were purchased from commercial suppliers, such as Sigma-Aldrich and TCI and used without further purification unless specially addressed.

### 2DPI–2DPBI vdW heterostructure

The lattice-matched 2DPI–2DPBI vdW heterostructure was synthesized by in situ sequential bottom-up assembly on the surface of water. In step I, Milli-Q water (40 ml) was injected into a beaker (80 ml, diameter 6 cm) to form a static air–water interface. Then SOS (10 µl, 1 mg ml^−1^ in chloroform) was spread on the water surface to form a charged surfactant monolayer. In step II, after 10 min, an acidic solution of 5,10,15,20-(tetra-4-aminophenyl)porphyrin (M1, 0.8 ml, 1 mg ml^−1^ in 0.30 M HCl aqueous solution) was injected into the subphase, resulting in electrostatic adsorption beneath the surfactant monolayer. After 1 h, the subphase was gradually exchanged with fresh Milli-Q water. In each exchange cycle, 2 ml of fresh Milli-Q water was slowly added while 2 ml of the subphase was slowly removed to avoid disrupting the interfacial assembly. This dilution–exchange process was repeated and monitored by ultraviolet–visible spectroscopy to ensure complete removal of excess, unadsorbed M1 before the subsequent monomer addition. In step III, an aqueous solution of 2,5-dihydroxyterephthalaldehyde (M2, 0.8 ml, 1 mg ml^−1^ in aqueous solution) was added to the subphase, initiating diffusion towards the pre-organized M1 layer. The 2D polymerization proceeded for 12 h under ambient conditions, yielding 2DPI. The subphase was then exchanged using the same slow 2 ml inlet/2 ml outlet procedure to remove excess unreacted M2. In step IV, a solution of 5,10,15,20-(tetra-4-carboxyphenyl)porphyrin (M3, 0.8 ml, 1 mg ml^−1^ in 0.05 M LiOH aqueous solution) was injected into the subphase, initiating diffusion towards the underlying 2DPI layer. After 1 h of adsorption, the subphase was again exchanged using the same procedure to remove non-adsorbed M3. In step V, an aqueous solution of 1,2,4,5-benzenetetramine (M4, 0.8 ml, 1 mg ml^−1^ in 0.30 M HCl aqueous solution) was added, promoting its diffusion towards the 2DPI–M3 interface. The 2D polymerization was carried out for 12 h at room temperature to synthesize the 2DPI–2DPBI vdW heterostructure. A final subphase exchange was performed to remove excess M4. The resulting 2DPI–2DPBI heterostructure comprises few-layer 2DPI and few-layer 2DPBI sequentially grown to form a vertically lattice-matched interface.

For thinner individual layers (<10 nm) within the heterostructure, subphase exchange was performed after each monomer addition step (steps II–V) to remove excess monomers, whereas for thicker individual layers (>10 nm), exchange was limited to steps II and IV to maintain higher monomer concentrations during 2D polymerization. Similarly, by varying the monomer combinations, we synthesized a series of heterostructures, comprising three commensurate lattice-matched, three small-mismatch (1.6%) and four large-mismatch (16.6% and 18.0%) configurations. As a control, individual 2D polymers were also synthesized separately in isolated beakers and subsequently stacked via manual transfer to form mechanically assembled ex situ heterostructures.

## Online content

Any methods, additional references, Nature Portfolio reporting summaries, source data, extended data, supplementary information, acknowledgements, peer review information; details of author contributions and competing interests; and statements of data and code availability are available at https://doi.org/10.1038/s41586-026-11074-6.

## Supplementary information

Supplementary Information This file contains supplementary materials.

Peer Review File

## Data Availability

The data supporting the findings of the study are available in the paper and its Supplementary Information.

## Code availability

All calculations presented in this work are performed using publicly available standard packages. All relevant information used for reproducibility can be found in the text and Supplementary Information.
