# Supplementary material for: Organic two-dimensional van der Waals heterostructures
Source: Nature. 2026 Sep 23;658(8135):388–97. doi: 10.1038/s41586-026-11074-6 (PMC13645628; doi:10.1038/s41586-026-11074-6)
Supplement: Supplementary file 1 [file 41586_2026_11074_MOESM1_ESM.pdf]

nature portfolio

https://doi.org/10.1038/s41586-026-11074-6

Supplementary information

Organic two-dimensional van der Waals heterostructures

In the format provided by the authors and unedited

Organic two-dimensional van der Waals heterostructures

Anupam Prasoon[1,2#], Nguyen Ngan Nguyen[1,2#], Mike Hambsch[3#], Puja Singhvi[4], Sophia Terres[5], Zijie Xiao[6], Naveen Goyal[2,7], Haoyuan Qi[2,7], David Mücke[7], Florian Auras[2], Zhiyong Wang[1,2], Sein Chung[8], Miroslav Položij[4], Hai I. Wang[6,9], Kilwon Cho[8], Ute Kaiser[7], Alexey Chernikov[5], Mischa Bonn[6], Stefan C. B. Mannsfeld[3*], Thomas Heine[4,10,11*], Xinliang Feng[1,2*]

[1]Max Planck Institute for Microstructure Physics, Halle (Saale) D-06120, Germany

[2]Center for Advancing Electronics Dresden (cfaed) and Faculty of Chemistry and Food Chemistry, Technische Universität Dresden, 01062 Dresden, Germany

[3]Faculty of Electrical and Computer Engineering and Center for Advancing Electronics Dresden (cfaed), TUD Dresden University of Technology, 01062 Dresden, Germany

[4]Chair of Theoretical Chemistry, Technische Universität Dresden, 01062 Dresden, Germany

[5]Institute of Applied Physics and Würzburg-Dresden Cluster of Excellence ct.qmat, Technische Universität Dresden, 01062 Dresden, Germany

[6]Max Planck Institute for Polymer Research, Ackermannweg 10, 55128 Mainz, Germany

[7]Central Facility for Electron Microscopy, Electron Microscopy of Materials Science, Ulm University, 89081 Ulm, Germany

[8]Department of Chemical Engineering, Pohang University of Science and Technology, Pohang, 37673 Republic of Korea

[9]Nanophotonics, Debye Institute for Nanomaterials Science, Utrecht University, Utrecht 3584, Netherlands

[10]Helmholtz-Zentrum Dresden-Rossendorf, Center for Advanced Systems Understanding (CASUS), Görlitz 02826, Germany

[11]Department of Chemistry and IBS Center for Nanomedicine, Yonsei University Seodaemun-gu, Seoul 120-749, Republic of Korea

[#]These authors contributed equally to this manuscript

E-mail: stefan.mannsfeld@tu-dresden.de; thomas.heine@tu-dresden.de; xinliang.feng@mpi-halle.mpg.de

Materials

All solvents, reagents and chemicals were purchased from commercial suppliers, such as Sigma-Aldrich and TCI and used without further purification unless specially addressed.

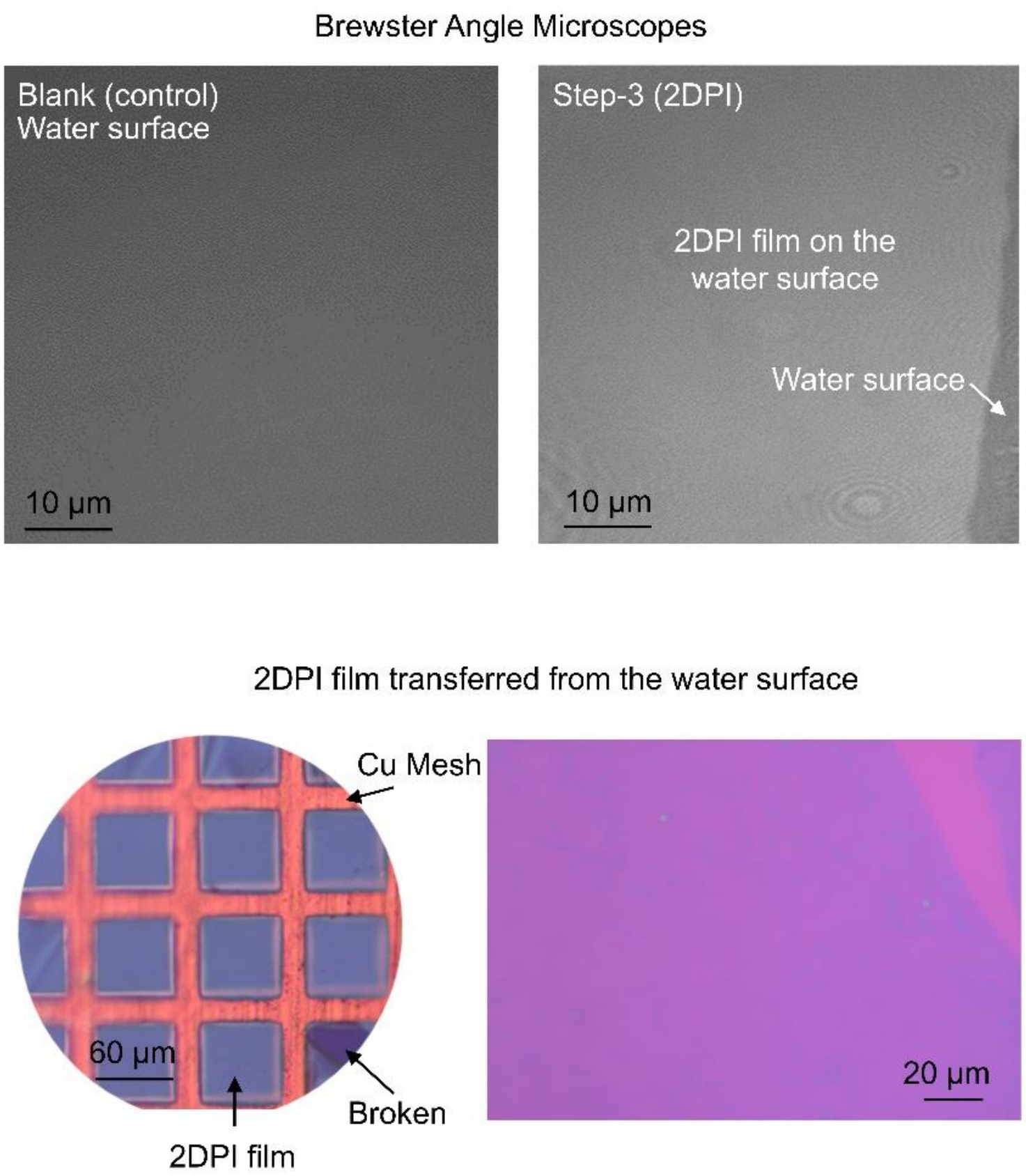

Supplementary Fig. 1 | Continuity of the 2DPI film on the water surface. Brewster angle microscopy images of a blank water surface and the 2DPI film in Step-III, showing clear film contrast on the water surface. Optical microscopy images of the transferred 2DPI film on a Cu mesh and SiO_2/Si substrate, showing large-area film continuity with visible film edges.

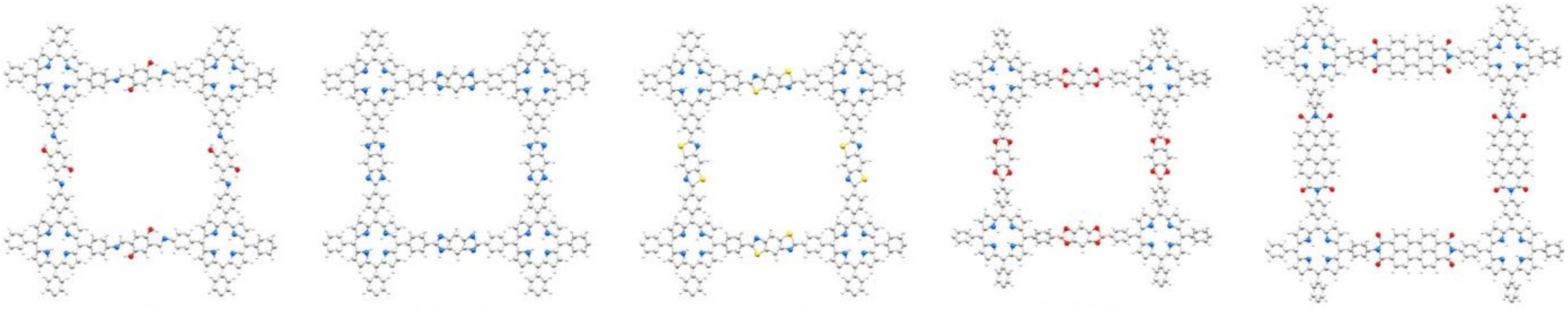

Supplementary Fig. 2 | Parent 2D polymers. Chemical structures and crystallographic lattice parameters (a, b, and γ) of five chemically distinct parent 2D polymers used in this work: 2DPI (imine), 2DPBI (imidazole), 2DPBT (thiazole), 2DPBE (boronate ester), and 2DPID (imide).

2DPI–2DPBI vdW heterostructure

The lattice-matched 2DPI–2DPBI vdW heterostructure was synthesized by in situ sequential bottom-up assembly on the water surface. In Step-I, Milli-Q water (40 mL) was injected into a beaker (80 mL, diameter = 6 cm) to form a static air–water interface. Then, sodium oleyl sulfate (SOS, 10 µL, 1 mg mL^{-1} in chloroform) was spread on the water surface to form a charged surfactant monolayer. In Step-II, after 10 min, an acidic solution of 5,10,15,20-(tetra-4-aminophenyl)porphyrin (M1, 0.8 mL, 1 mg mL^{-1} in 0.30 M HCl aqueous solution) was injected into the subphase, resulting in electrostatic adsorption beneath the surfactant monolayer. After 1 h, the subphase was gradually exchanged with fresh Milli-Q water. In each exchange cycle, 2 mL of fresh Milli-Q water was slowly added while 2 mL of the subphase was slowly removed to avoid disrupting the interfacial assembly. This dilution–exchange process was repeated and monitored by UV–vis spectroscopy to ensure complete removal of excess, unadsorbed M1 before the subsequent monomer addition. In Step-III, an aqueous solution of 2,5-dihydroxyterephthalaldehyde (M2, 0.8 mL, 1 mg mL^{-1} in aqueous solution) was added to the subphase, initiating diffusion toward the pre-organized M1 layer. The 2D polymerization proceeded for 12 h under ambient conditions, yielding 2DPI. The subphase was then exchanged using the same slow 2 mL inlet/2 mL outlet procedure to remove excess unreacted M2. In Step-IV, a solution of 5,10,15,20-(tetra-4-carboxyphenyl)porphyrin (M3, 0.8 mL, 1 mg mL^{-1} in 0.05 M LiOH aqueous solution) was injected into the subphase, initiating diffusion toward the underlying 2DPI layer. After 1 h of adsorption, the subphase was again exchanged using the same procedure to remove non-adsorbed M3. In Step-V, an aqueous solution of 1,2,4,5-benzenetetramine (M4, 0.8 mL, 1 mg mL^{-1} in 0.30 M HCl aqueous solution) was added, promoting its diffusion toward the 2DPI–M3 interface. The 2D polymerization was carried out for 12 h at room temperature to synthesize the 2DPI–2DPBI vdW heterostructure. A final subphase exchange was performed to remove excess M4. The resulting 2DPI–2DPBI heterostructure comprises few-layer 2DPI and few-layer 2DPBI sequentially grown to form a vertically lattice-matched interface.

For thinner individual layers (<10 nm) within the heterostructure, subphase exchange was performed after each monomer addition step (Steps-II–V) to remove excess monomers, whereas for thicker individual layers (>10 nm), exchange was limited to Steps-II and -IV to maintain higher monomer concentrations during 2D polymerization. Similarly, by varying the monomer combinations, we synthesized a series of heterostructures, comprising three commensurate lattice-matched, three small-mismatch (1.6%), and four large-mismatch (16.6% and 18.0%) configurations. As a control, individual 2D polymers were also synthesized separately in isolated beakers and subsequently stacked via manual transfer to form mechanically assembled ex situ heterostructures.

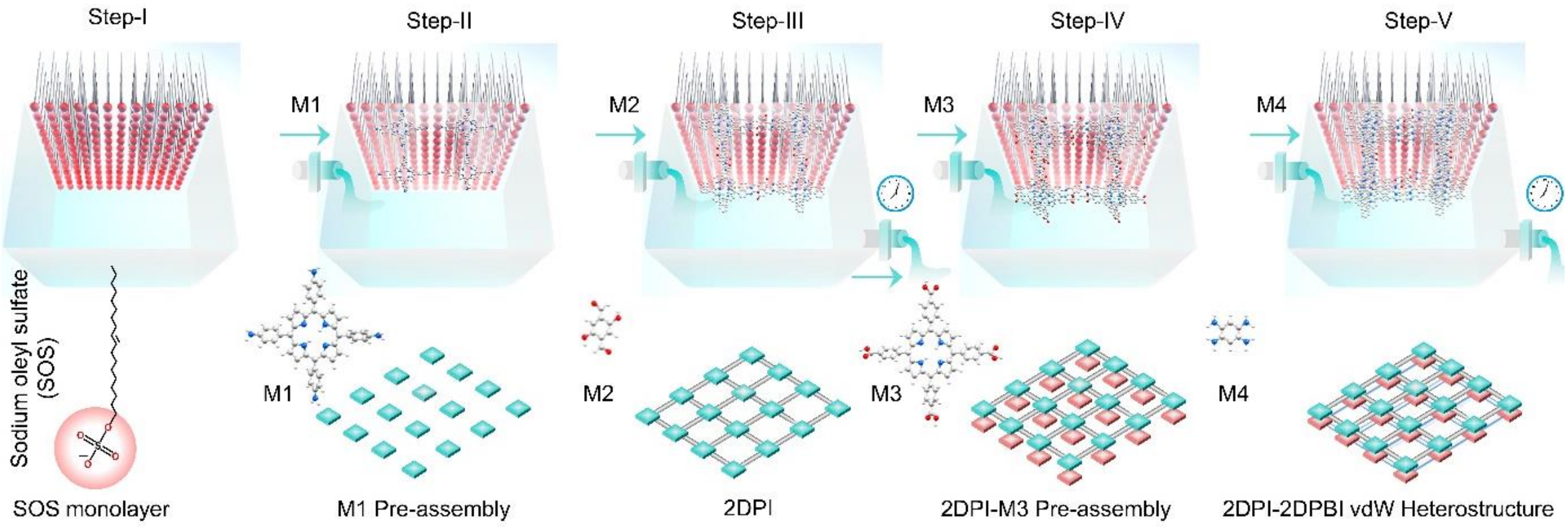

Supplementary Fig. 3 | Subphase exchange protocol and thicker samples. Subphase exchange was carried out by gradual replacement with fresh Milli-Q water (2 mL inlet, 2 mL outlet) to avoid disrupting the interfacial assembly; repeated dilution–exchange cycles were monitored by UV–vis to ensure removal of excess, unadsorbed monomers before the next addition. The heterostructure comprises a few-layer 2DPI and a few-layer 2DPBI sequentially grown to form a vertically lattice-matched interface. For thinner individual layers (<10 nm), exchange was performed after each monomer addition step (Steps-II–V). For thicker individual layers (>10 nm), the subphase was not exchanged at Step-II and Step-IV (i.e., after M1 and M3 adsorption), and exchange was limited to Steps-III and -V (after M2 and M4) so that higher monomer concentrations were maintained during 2D polymerization to promote thicker growth.

2DPI–2DPBI vdW heterostructure

The lattice-matched 2DPI–2DPBI vdW heterostructure was synthesized by in situ sequential bottom-up assembly on the water surface. In Step-I, Milli-Q water (40 mL) was injected into a beaker (80 mL, diameter = 6 cm) to form a static air-water interface. Then, sodium oleyl sulfate (SOS, 10 µL, 1 mg mL^{-1} in chloroform) was spread on the water surface to form a charged surfactant monolayer. In Step-II, after 10 min, a solution of 5,10,15,20-(tetra-4-aminophenyl)porphyrin (M1, 0.8 mL, 1 mg mL^{-1} in 0.30 M HCl aqueous solution) was injected into the subphase, resulting in electrostatic adsorption beneath the surfactant monolayer. After 1 h, the subphase was gradually exchanged with fresh Milli-Q water. In each exchange cycle, 2 mL of fresh Milli-Q water was slowly added while 2 mL of the subphase was slowly removed to avoid disrupting the interfacial assembly. This dilution-exchange process was repeated and monitored by UV-vis spectroscopy to ensure complete removal of excess, unadsorbed M1

before the subsequent monomer addition. In Step-III, a solution of 2,5-dihydroxyterephthalaldehyde (M2, 0.9 mL, 1 mg mL^{-1} in aqueous solution) was added to the subphase, initiating diffusion toward the pre-organized M1 layer. The 2D polymerization proceeded for 12 h under ambient conditions, yielding 2DPI. The subphase was then exchanged using the same slow 2 mL inlet/2 mL outlet procedure to remove excess unreacted M2. In Step-IV, a solution of 5,10,15,20-(tetra-4-carboxyphenyl)porphyrin (M3, 0.9 mL, 1 mg mL^{-1} in 0.05 M LiOH aqueous solution) was injected into the subphase, initiating diffusion toward the underlying 2DPI layer. After 1 h of adsorption, the subphase was again exchanged using the same procedure to remove non-adsorbed M3. In Step-V, a solution of 1,2,4,5-benzenetetramine (M4, 0.8 mL, 1 mg mL^{-1} in 0.30 M HCl aqueous solution) was added, promoting its diffusion toward the 2DPI-M3 interface. The 2D polymerization was carried out for 12 h at room temperature to synthesize the 2DPI–2DPBI vdW heterostructure. A final subphase exchange was performed to remove excess M4. The resulting 2DPI–2DPBI heterostructure comprises few-layer 2DPI and few-layer 2DPBI sequentially grown to form a vertically lattice-matched interface.

2DPI–2DPBT vdW heterostructure

The lattice-matched 2DPI–2DPBT vdW heterostructure was synthesized by in situ sequential bottom-up assembly on the water surface. In Step-I, Milli-Q water (40 mL) was injected into a beaker (80 mL, diameter = 6 cm) to form a static air-water interface. Then, sodium oleyl sulfate (SOS, 10 µL, 1 mg mL^{-1} in chloroform) was spread on the water surface to form a charged surfactant monolayer. In Step-II, after 10 min, a solution of 5,10,15,20-(tetra-4-aminophenyl)porphyrin (M1, 0.8 mL, 1 mg mL^{-1} in 0.30 M HCl aqueous solution) was injected into the subphase, resulting in electrostatic adsorption beneath the surfactant monolayer. After 1 h, the subphase was gradually exchanged with fresh Milli-Q water. In each exchange cycle, 2 mL of fresh Milli-Q water was slowly added while 2 mL of the subphase was slowly removed to avoid disrupting the interfacial assembly. This dilution-exchange process was repeated and monitored by UV-vis spectroscopy to ensure complete removal of excess, unadsorbed M1 before the subsequent monomer addition. In Step-III, a solution of 2,5-dihydroxyterephthalaldehyde (M2, 0.9 mL, 1 mg mL^{-1} in aqueous solution) was added to the subphase, initiating diffusion toward the pre-organized M1 layer. The 2D polymerization proceeded for 12 h under ambient conditions, yielding 2DPI. The subphase was then exchanged using the same slow 2 mL inlet/2 mL outlet procedure to remove excess unreacted M2. In Step-IV, a solution of 5,10,15,20-(tetra-4-carboxyphenyl)porphyrin (M3, 0.9 mL, 1 mg mL^{-1} in 0.05 M LiOH aqueous solution) was injected into the subphase, initiating diffusion toward the underlying 2DPI layer. After 1 h of adsorption, the subphase was again exchanged using the same procedure to remove non-adsorbed M3. In Step-V, a solution of 2,5-diamino-1,4-benzenedithiol dihydrochloride (M5, 0.9 mL, 1 mg mL^{-1} in 0.25 M HCl aqueous solution) was added, promoting its diffusion toward the 2DPI-M3 interface. The 2D polymerization was carried out for 12 h at room temperature to synthesize the 2DPI–2DPBT vdW heterostructure. A final subphase exchange

was performed to remove excess M5. The resulting 2DPI–2DPBT heterostructure comprises few-layer 2DPI and few-layer 2DPBT sequentially grown to form a vertically lattice-matched interface.

2DPBT–2DPBI vdW heterostructure

The lattice-matched 2DPBT–2DPBI vdW heterostructure was synthesized by in situ sequential bottom-up assembly on the water surface. In Step-I, Milli-Q water (40 mL) was injected into a beaker (80 mL, diameter = 6 cm) to form a static air-water interface. Then, CTAB (15 µL, 1 mg mL^{-1} in chloroform) was spread on the water surface to form a charged surfactant monolayer. In Step-II, after 10 min, a solution of 5,10,15,20-(tetra-4-carboxyphenyl)porphyrin (M3, 0.9 mL, 1 mg mL^{-1} in 0.05 M LiOH aqueous solution) was injected into the subphase, resulting in adsorption beneath the surfactant monolayer. After 1 h, the subphase was gradually exchanged with fresh Milli-Q water. In each exchange cycle, 2 mL of fresh Milli-Q water was slowly added while 2 mL of the subphase was slowly removed to avoid disrupting the interfacial assembly. This dilution-exchange process was repeated and monitored by UV-vis spectroscopy to ensure complete removal of excess, unadsorbed M3 before the subsequent monomer addition. In Step-III, a solution of 2,5-diamino-1,4-benzenedithiol dihydrochloride (M5, 0.9 mL, 1 mg mL^{-1} in 0.25 M HCl aqueous solution) was added to the subphase, initiating diffusion toward the pre-organized M3 layer. The 2D polymerization proceeded for 12 h under ambient conditions, yielding 2DPBT. The subphase was then exchanged using the same slow 2 mL inlet/2 mL outlet procedure to remove excess unreacted M5. In Step-IV, a solution of 5,10,15,20-(tetra-4-carboxyphenyl)porphyrin (M3, 0.9 mL, 1 mg mL^{-1} in 0.05 M LiOH aqueous solution) was injected into the subphase, initiating diffusion toward the underlying 2DPBT layer. After 1 h of adsorption, the subphase was again exchanged using the same procedure to remove non-adsorbed M3. In Step-V, a solution of 1,2,4,5-benzenetetramine (M4, 0.8 mL, 1 mg mL^{-1} in 0.30 M HCl aqueous solution) was added, promoting its diffusion toward the 2DPBT-M3 interface. The 2D polymerization was carried out for 12 h at room temperature to synthesize the 2DPBT–2DPBI vdW heterostructure. A final subphase exchange was performed to remove excess M4. The resulting 2DPBT–2DPBI heterostructure comprises few-layer 2DPBT and few-layer 2DPBI sequentially grown to form a vertically lattice-matched interface.

2DPBE–2DPBI vdW heterostructure

The small-mismatch 2DPBE–2DPBI vdW heterostructure was synthesized by in situ sequential bottom-up assembly on the water surface. In Step-I, Milli-Q water (40 mL) was injected into a beaker (80 mL, diameter = 6 cm) to form a static air-water interface. Then, sodium oleyl sulfate (SOS, 10 µL, 1 mg mL^{-1} in chloroform) was spread on the water surface to form a charged surfactant monolayer. In Step-II, after 10 min, a solution of 5,10,15,20-(tetra-4-dihydroxyborylphenyl)porphyrin (M6, 0.9 mL, 1 mg mL^{-1} in 0.25 M HCl aqueous solution) was injected into the subphase, resulting in electrostatic adsorption beneath the surfactant monolayer. After 1 h, the subphase was gradually exchanged with fresh Milli-Q

water. In each exchange cycle, 2 mL of fresh Milli-Q water was slowly added while 2 mL of the subphase was slowly removed to avoid disrupting the interfacial assembly. This dilution-exchange process was repeated and monitored by UV-vis spectroscopy to ensure complete removal of excess, unadsorbed M6 before the subsequent monomer addition. In Step-III, a solution of 1,2,4,5-tetrahydroxybenzene (M7, 0.9 mL, 1 mg mL^{-1} in 0.25 M HCl aqueous solution) was added to the subphase, initiating diffusion toward the pre-organized M6 layer. The polycondensation reaction proceeded for 12 h under ambient conditions, yielding 2DPBE. The subphase was then exchanged using the same slow 2 mL inlet/2 mL outlet procedure to remove excess unreacted M7. In Step-IV, a solution of 5,10,15,20-(tetra-4-carboxyphenyl)porphyrin (M3, 0.9 mL, 1 mg mL^{-1} in 0.05 M LiOH aqueous solution) was injected into the subphase, initiating diffusion toward the underlying 2DPBE layer. After 1 h of adsorption, the subphase was again exchanged using the same procedure to remove non-adsorbed M3. In Step-V, a solution of 1,2,4,5-benzenetetramine (M4, 0.8 mL, 1 mg mL^{-1} in 0.30 M HCl aqueous solution) was added, promoting its diffusion toward the 2DPBE-M3 interface. The 2D polymerization was carried out for 12 h at room temperature to synthesize the 2DPBE–2DPBI vdW heterostructure. A final subphase exchange was performed to remove excess M4. The resulting 2DPBE–2DPBI heterostructure comprises few-layer 2DPBE and few-layer 2DPBI sequentially grown to form a vertically stacked small-mismatch interface.

2DPBE–2DPBT vdW heterostructure

The small-mismatch 2DPBE–2DPBT vdW heterostructure was synthesized by in situ sequential bottom-up assembly on the water surface. In Step-I, Milli-Q water (40 mL) was injected into a beaker (80 mL, diameter = 6 cm) to form a static air-water interface. Then, sodium oleyl sulfate (SOS, 10 µL, 1 mg mL^{-1} in chloroform) was spread on the water surface to form a charged surfactant monolayer. In Step-II, after 10 min, a solution of 5,10,15,20-(tetra-4-dihydroxyborylphenyl)porphyrin (M6, 0.9 mL, 1 mg mL^{-1} in 0.25 M HCl aqueous solution) was injected into the subphase, resulting in electrostatic adsorption beneath the surfactant monolayer. After 1 h, the subphase was gradually exchanged with fresh Milli-Q water. In each exchange cycle, 2 mL of fresh Milli-Q water was slowly added while 2 mL of the subphase was slowly removed to avoid disrupting the interfacial assembly. This dilution-exchange process was repeated and monitored by UV-vis spectroscopy to ensure complete removal of excess, unadsorbed M6 before the subsequent monomer addition. In Step-III, a solution of 1,2,4,5-tetrahydroxybenzene (M7, 0.9 mL, 1 mg mL^{-1} in 0.25 M HCl aqueous solution) was added to the subphase, initiating diffusion toward the pre-organized M6 layer. The polycondensation reaction proceeded for 12 h under ambient conditions, yielding 2DPBE. The subphase was then exchanged using the same slow 2 mL inlet/2 mL outlet procedure to remove excess unreacted M7. In Step-IV, a solution of 5,10,15,20-(tetra-4-carboxyphenyl)porphyrin (M3, 0.9 mL, 1 mg mL^{-1} in 0.05 M LiOH aqueous solution) was injected into the subphase, initiating diffusion toward the underlying 2DPBE layer. After 1 h of adsorption, the subphase was again exchanged using the same procedure to remove non-adsorbed

M3. In Step-V, a solution of 2,5-diamino-1,4-benzenedithiol dihydrochloride (M5, 0.9 mL, 1 mg mL^{-1} in 0.25 M HCl aqueous solution) was added, promoting its diffusion toward the 2DPBE-M3 interface. The 2D polymerization was carried out for 12 h at room temperature to synthesize the 2DPBE–2DPBT vdW heterostructure. A final subphase exchange was performed to remove excess M5. The resulting 2DPBE–2DPBT heterostructure comprises few-layer 2DPBE and few-layer 2DPBT sequentially grown to form a vertically stacked small-mismatch interface.

2DPBE–2DPI vdW heterostructure

The small-mismatch 2DPBE–2DPI vdW heterostructure was synthesized by in situ sequential bottom-up assembly on the water surface. In Step-I, Milli-Q water (40 mL) was injected into a beaker (80 mL, diameter = 6 cm) to form a static air-water interface. Then, sodium oleyl sulfate (SOS, 10 µL, 1 mg mL^{-1} in chloroform) was spread on the water surface to form a charged surfactant monolayer. In Step-II, after 10 min, a solution of 5,10,15,20-(tetra-4-dihydroxyborylphenyl)porphyrin (M6, 0.9 mL, 1 mg mL^{-1} in 0.25 M HCl aqueous solution) was injected into the subphase, resulting in electrostatic adsorption beneath the surfactant monolayer. After 1 h, the subphase was gradually exchanged with fresh Milli-Q water. In each exchange cycle, 2 mL of fresh Milli-Q water was slowly added while 2 mL of the subphase was slowly removed to avoid disrupting the interfacial assembly. This dilution-exchange process was repeated and monitored by UV-vis spectroscopy to ensure complete removal of excess, unadsorbed M6 before the subsequent monomer addition. In Step-III, a solution of 1,2,4,5-tetrahydroxybenzene (M7, 0.9 mL, 1 mg mL^{-1} in 0.25 M HCl aqueous solution) was added to the subphase, initiating diffusion toward the pre-organized M6 layer. The polycondensation reaction proceeded for 12 h under ambient conditions, yielding 2DPBE. The subphase was then exchanged using the same slow 2 mL inlet/2 mL outlet procedure to remove excess unreacted M7. In Step-IV, a solution of 5,10,15,20-(tetra-4-aminophenyl)porphyrin (M1, 0.8 mL, 1 mg mL^{-1} in 0.30 M HCl aqueous solution) was injected into the subphase, initiating diffusion toward the underlying 2DPBE layer. After 1 h of adsorption, the subphase was again exchanged using the same procedure to remove non-adsorbed M1. In Step-V, a solution of 2,5-dihydroxyterephthalaldehyde (M2, 0.9 mL, 1 mg mL^{-1} in aqueous solution) was added, promoting its diffusion toward the 2DPBE-M1 interface. The 2D polymerization was carried out for 12 h at room temperature to synthesize the 2DPBE–2DPI vdW heterostructure. A final subphase exchange was performed to remove excess M2. The resulting 2DPBE–2DPI heterostructure comprises few-layer 2DPBE and few-layer 2DPI sequentially grown to form a vertically stacked small-mismatch interface.

2DPID–2DPBI vdW heterostructure

The large-mismatch 2DPID–2DPBI vdW heterostructure was synthesized by in situ sequential bottom-up assembly on the water surface. In Step-I, Milli-Q water (40 mL) was injected into a beaker (80 mL, diameter = 6 cm) to form a static air-water interface. Then, sodium oleyl sulfate (SOS, 10 µL, 1 mg mL^{-}

^{1} in chloroform) was spread on the water surface to form a charged surfactant monolayer. In Step-II, after 10 min, a solution of 5,10,15,20-(tetra-4-aminophenyl)porphyrin (M1, 0.8 mL, 1 mg mL^{-1} in 0.30 M HCl aqueous solution) was injected into the subphase, resulting in electrostatic adsorption beneath the surfactant monolayer. After 1 h, the subphase was gradually exchanged with fresh Milli-Q water. In each exchange cycle, 2 mL of fresh Milli-Q water was slowly added while 2 mL of the subphase was slowly removed to avoid disrupting the interfacial assembly. This dilution-exchange process was repeated and monitored by UV-vis spectroscopy to ensure complete removal of excess, unadsorbed M1 before the subsequent monomer addition. In Step-III, a solution of perylene-3,4,9,10-tetracarboxylic dianhydride (M8, 0.9 mL, 1 mg mL^{-1} in 0.08 M LiOH aqueous solution) was added to the subphase, initiating diffusion toward the pre-organized M1 layer. The 2D polymerization proceeded for 12 h under ambient conditions, yielding 2DPID. The subphase was then exchanged using the same slow 2 mL inlet/2 mL outlet procedure to remove excess unreacted M8. In Step-IV, a solution of 5,10,15,20-(tetra-4-carboxyphenyl)porphyrin (M3, 0.9 mL, 1 mg mL^{-1} in 0.05 M LiOH aqueous solution) was injected into the subphase, initiating diffusion toward the underlying 2DPID layer. After 1 h of adsorption, the subphase was again exchanged using the same procedure to remove non-adsorbed M3. In Step-V, a solution of 1,2,4,5-benzenetetramine (M4, 0.8 mL, 1 mg mL^{-1} in 0.30 M HCl aqueous solution) was added, promoting its diffusion toward the 2DPID-M3 interface. The 2D polymerization was carried out for 12 h at room temperature to synthesize the 2DPID–2DPBI vdW heterostructure. A final subphase exchange was performed to remove excess M4. The resulting 2DPID–2DPBI heterostructure comprises few-layer 2DPID and few-layer 2DPBI sequentially grown to form a vertically stacked large-mismatch interface.

2DPID–2DPBT vdW heterostructure

The large-mismatch 2DPID–2DPBT vdW heterostructure was synthesized by in situ sequential bottom-up assembly on the water surface. In Step-I, Milli-Q water (40 mL) was injected into a beaker (80 mL, diameter = 6 cm) to form a static air-water interface. Then, sodium oleyl sulfate (SOS, 10 µL, 1 mg mL^{-1} in chloroform) was spread on the water surface to form a charged surfactant monolayer. In Step-II, after 10 min, a solution of 5,10,15,20-(tetra-4-aminophenyl)porphyrin (M1, 0.8 mL, 1 mg mL^{-1} in 0.30 M HCl aqueous solution) was injected into the subphase, resulting in electrostatic adsorption beneath the surfactant monolayer. After 1 h, the subphase was gradually exchanged with fresh Milli-Q water. In each exchange cycle, 2 mL of fresh Milli-Q water was slowly added while 2 mL of the subphase was slowly removed to avoid disrupting the interfacial assembly. This dilution-exchange process was repeated and monitored by UV-vis spectroscopy to ensure complete removal of excess, unadsorbed M1 before the subsequent monomer addition. In Step-III, a solution of perylene-3,4,9,10-tetracarboxylic dianhydride (M8, 0.9 mL, 1 mg mL^{-1} in 0.08 M LiOH aqueous solution) was added to the subphase, initiating diffusion toward the pre-organized M1 layer. The 2D polymerization proceeded for 12 h under ambient conditions, yielding 2DPID. The subphase was then exchanged using the same slow 2 mL

inlet/2 mL outlet procedure to remove excess unreacted M8. In Step-IV, a solution of 5,10,15,20-(tetra-4-carboxyphenyl)porphyrin (M3, 0.9 mL, 1 mg mL^{-1} in 0.05 M LiOH aqueous solution) was injected into the subphase, initiating diffusion toward the underlying 2DPID layer. After 1 h of adsorption, the subphase was again exchanged using the same procedure to remove non-adsorbed M3. In Step-V, a solution of 2,5-diamino-1,4-benzenedithiol dihydrochloride (M5, 0.9 mL, 1 mg mL^{-1} in 0.25 M HCl aqueous solution) was added, promoting its diffusion toward the 2DPID-M3 interface. The 2D polymerization was carried out for 12 h at room temperature to synthesize the 2DPID–2DPBT vdW heterostructure. A final subphase exchange was performed to remove excess M5. The resulting 2DPID–2DPBT heterostructure comprises few-layer 2DPID and few-layer 2DPBT sequentially grown to form a vertically stacked large-mismatch interface.

2DPID–2DPI vdW heterostructure

The large-mismatch 2DPID–2DPI vdW heterostructure was synthesized by in situ sequential bottom-up assembly on the water surface. In Step-I, Milli-Q water (40 mL) was injected into a beaker (80 mL, diameter = 6 cm) to form a static air-water interface. Then, sodium oleyl sulfate (SOS, 10 µL, 1 mg mL^{-1} in chloroform) was spread on the water surface to form a charged surfactant monolayer. In Step-II, after 10 min, a solution of 5,10,15,20-(tetra-4-aminophenyl)porphyrin (M1, 0.8 mL, 1 mg mL^{-1} in 0.30 M HCl aqueous solution) was injected into the subphase, resulting in electrostatic adsorption beneath the surfactant monolayer. After 1 h, the subphase was gradually exchanged with fresh Milli-Q water. In each exchange cycle, 2 mL of fresh Milli-Q water was slowly added while 2 mL of the subphase was slowly removed to avoid disrupting the interfacial assembly. This dilution-exchange process was repeated and monitored by UV-vis spectroscopy to ensure complete removal of excess, unadsorbed M1 before the subsequent monomer addition. In Step-III, a solution of perylene-3,4,9,10-tetracarboxylic dianhydride (M8, 0.9 mL, 1 mg mL^{-1} in 0.08 M LiOH aqueous solution) was added to the subphase, initiating diffusion toward the pre-organized M1 layer. The 2D polymerization proceeded for 12 h under ambient conditions, yielding 2DPID. The subphase was then exchanged using the same slow 2 mL inlet/2 mL outlet procedure to remove excess unreacted M8. In Step-IV, a solution of 5,10,15,20-(tetra-4-aminophenyl)porphyrin (M1, 0.8 mL, 1 mg mL^{-1} in 0.30 M HCl aqueous solution) was injected into the subphase, initiating diffusion toward the underlying 2DPID layer. After 1 h of adsorption, the subphase was again exchanged using the same procedure to remove non-adsorbed M1. In Step-V, a solution of 2,5-dihydroxyterephthalaldehyde (M2, 0.9 mL, 1 mg mL^{-1} in aqueous solution) was added, promoting its diffusion toward the 2DPID-M1 interface. The 2D polymerization was carried out for 12 h at room temperature to synthesize the 2DPID–2DPI vdW heterostructure. A final subphase exchange was performed to remove excess M2. The resulting 2DPID–2DPI heterostructure comprises few-layer 2DPID and few-layer 2DPI sequentially grown to form a vertically stacked large-mismatch interface.

2DPID–2DPBE vdW heterostructure

The large-mismatch 2DPID–2DPBE vdW heterostructure was synthesized by in situ sequential bottom-up assembly on the water surface. In Step-I, Milli-Q water (40 mL) was injected into a beaker (80 mL, diameter = 6 cm) to form a static air-water interface. Then, sodium oleyl sulfate (SOS, 10 μL, 1 mg mL^{-1} in chloroform) was spread on the water surface to form a charged surfactant monolayer. In Step-II, after 10 min, a solution of 5,10,15,20-(tetra-4-aminophenyl)porphyrin (M1, 0.8 mL, 1 mg mL^{-1} in 0.30 M HCl aqueous solution) was injected into the subphase, resulting in electrostatic adsorption beneath the surfactant monolayer. After 1 h, the subphase was gradually exchanged with fresh Milli-Q water. In each exchange cycle, 2 mL of fresh Milli-Q water was slowly added while 2 mL of the subphase was slowly removed to avoid disrupting the interfacial assembly. This dilution-exchange process was repeated and monitored by UV-vis spectroscopy to ensure complete removal of excess, unadsorbed M1 before the subsequent monomer addition. In Step-III, a solution of perylene-3,4,9,10-tetracarboxylic dianhydride (M8, 0.9 mL, 1 mg mL^{-1} in 0.08 M LiOH aqueous solution) was added to the subphase, initiating diffusion toward the pre-organized M1 layer. The 2D polymerization proceeded for 12 h under ambient conditions, yielding 2DPID. The subphase was then exchanged using the same slow 2 mL inlet/2 mL outlet procedure to remove excess unreacted M8. In Step-IV, a solution of 5,10,15,20-(tetra-4-dihydroxyborylphenyl)porphyrin (M6, 0.9 mL, 1 mg mL^{-1} in 0.25 M HCl aqueous solution) was injected into the subphase, initiating diffusion toward the underlying 2DPID layer. After 1 h of adsorption, the subphase was again exchanged using the same procedure to remove non-adsorbed M6. In Step-V, a solution of 1,2,4,5-tetrahydroxybenzene (M7, 0.9 mL, 1 mg mL^{-1} in 0.25 M HCl aqueous solution) was added, promoting its diffusion toward the 2DPID-M6 interface. The polycondensation reaction was carried out for 12 h at room temperature to synthesize the 2DPID–2DPBE vdW heterostructure. A final subphase exchange was performed to remove excess M7. The resulting 2DPID–2DPBE heterostructure comprises few-layer 2DPID and few-layer 2DPBE sequentially grown to form a vertically stacked large-mismatch interface.

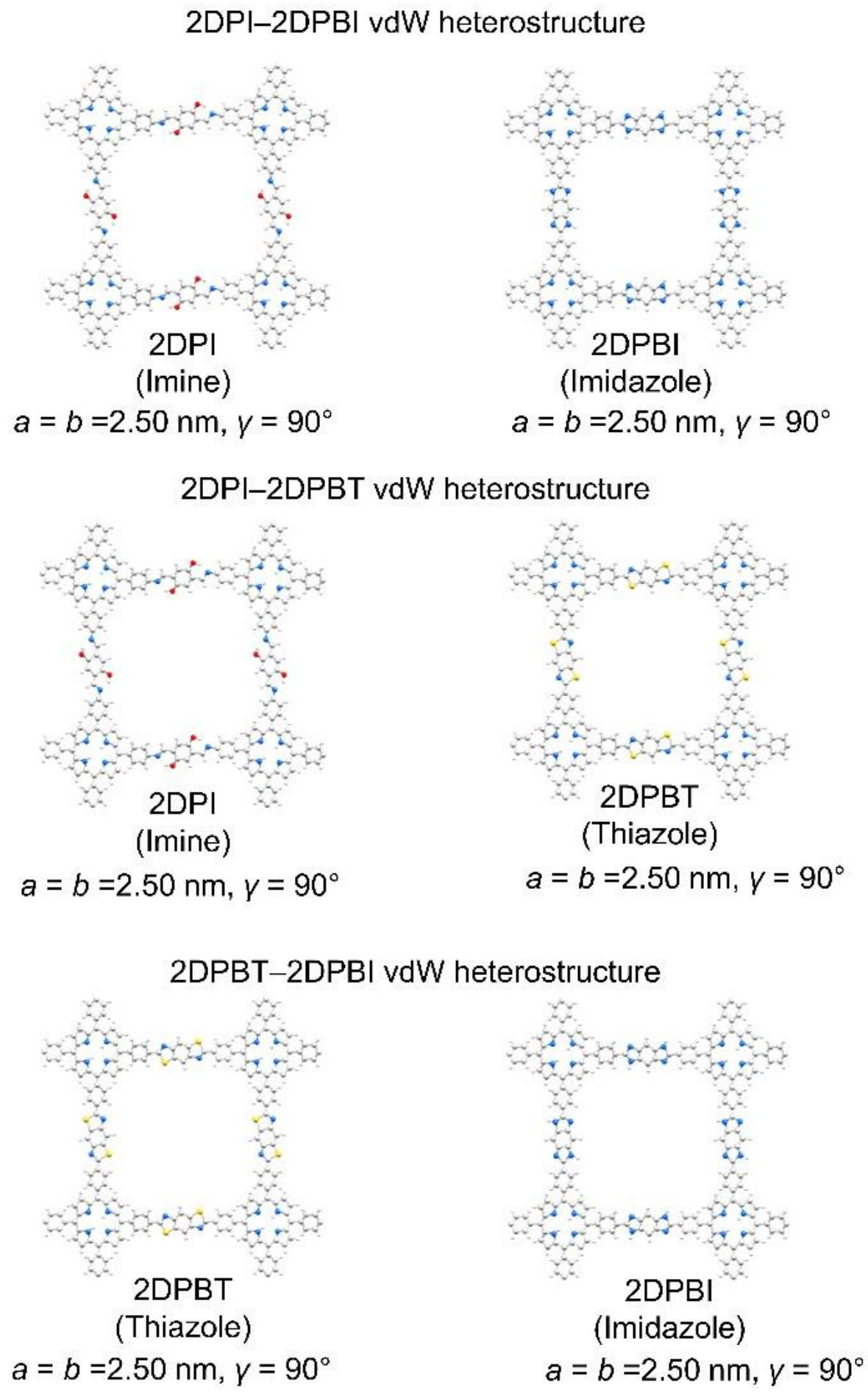

Supplementary Fig. 4 | Commensurate lattice-matched vdW heterostructures. Chemical structures and lattice parameters (a, b, and γ) of the polymer pairs forming the 2DPI–2DPBI, 2DPI–2DPBT, and 2DPBT–2DPBI vdW heterostructures; all constituents have a = b = 2.50 nm and γ = 90°.

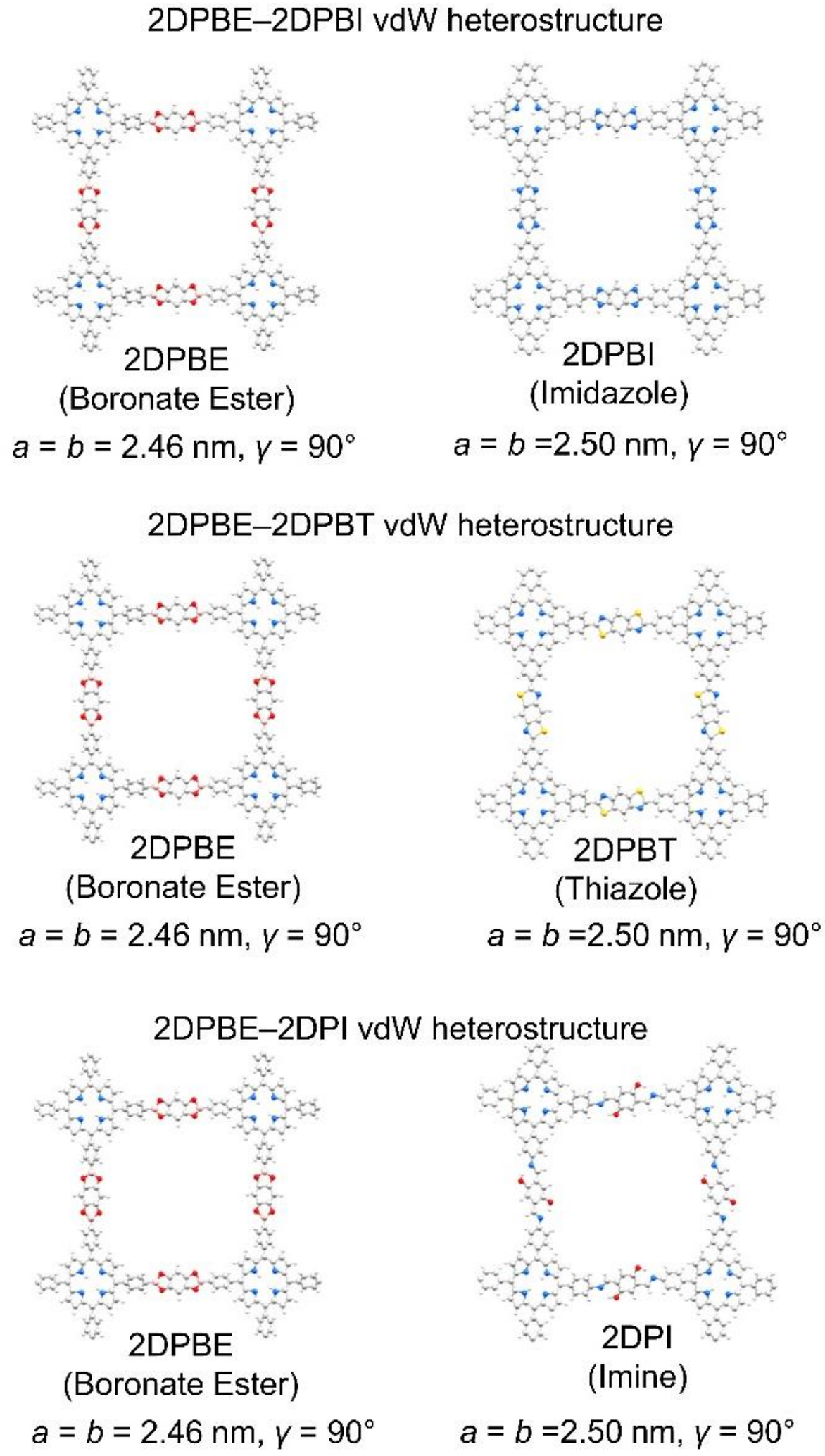

Supplementary Fig. 5 | Small-mismatch (1.6%) vdW heterostructures. Chemical structures and lattice parameters (a, b, and γ) of the polymer pairs forming the 2DPBE–2DPBI, 2DPBE–2DPBT, and 2DPBE–2DPI vdW heterostructures. 2DPBE has a = b = 2.46 nm and γ = 90°, whereas 2DPBI/2DPBT/2DPI have a = b = 2.50 nm and γ = 90°, corresponding to a 1.6% in-plane lattice mismatch.

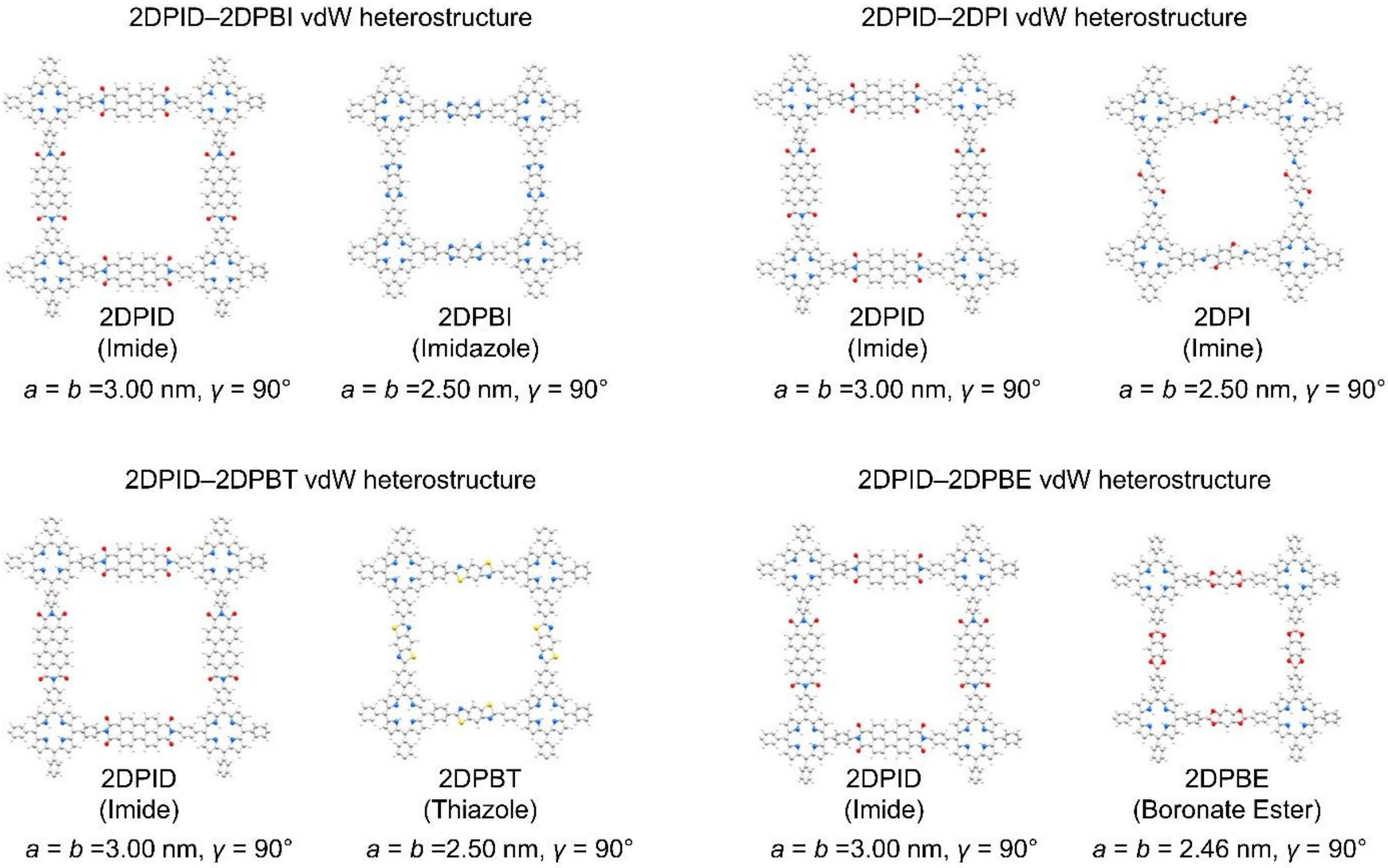

Supplementary Fig. 6 | Large-mismatch (16.6% and 18.0%) vdW heterostructures. Chemical structures and lattice parameters (a, b, and γ) of the polymer pairs forming the 2DPID–2DPBI, 2DPID–2DPI, and 2DPID–2DPBT vdW heterostructures (16.6% mismatch), and the 2DPID–2DPBE vdW heterostructure (18.0% mismatch). 2DPID has a = b = 3.00 nm and γ = 90°, compared with a = b = 2.50 nm (2DPBI/2DPI/2DPBT) or a = b = 2.46 nm (2DPBE), with γ = 90°.

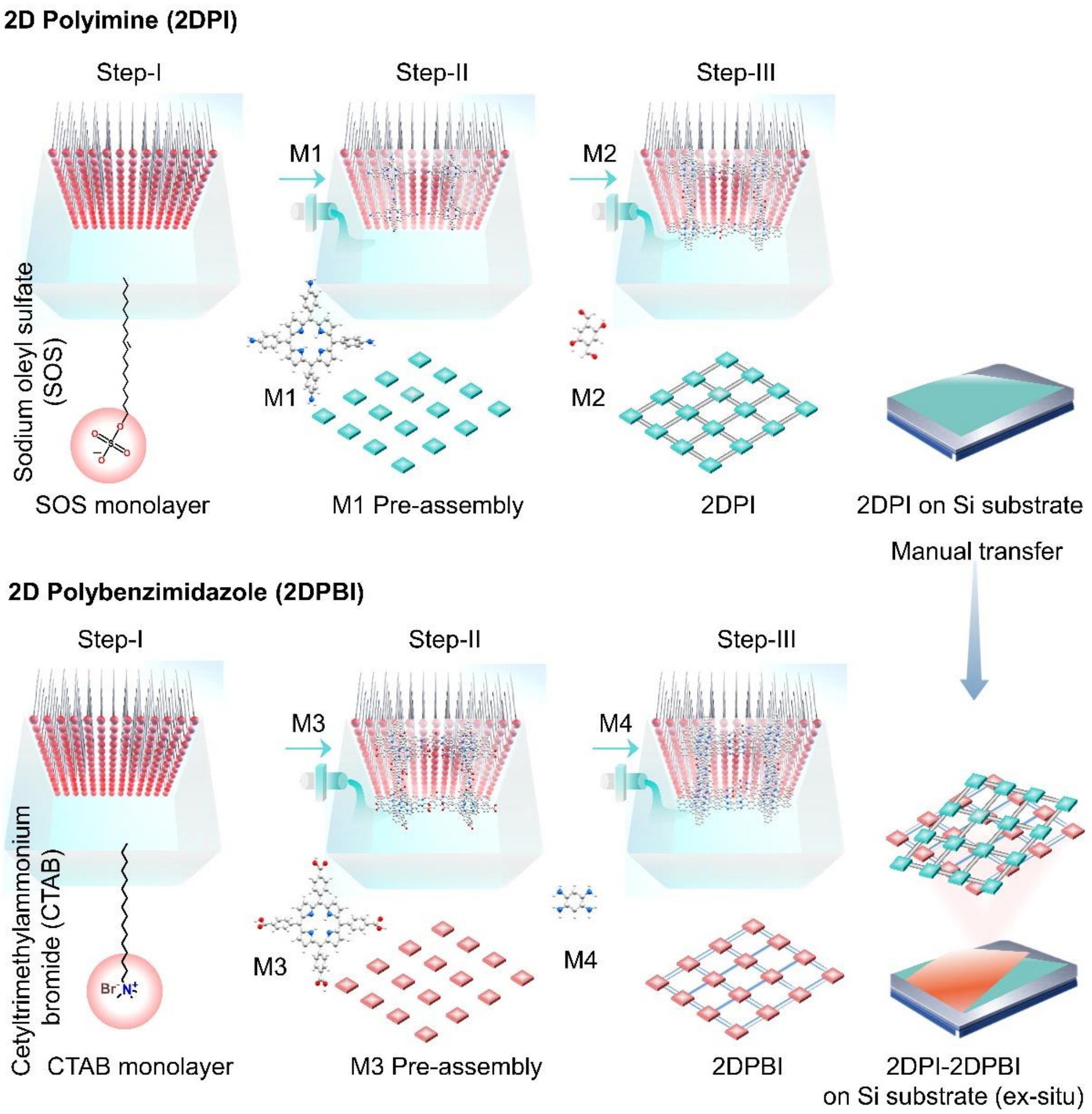

Supplementary Fig. 7 | Mechanically assembled (ex-situ) 2DPI–2DPBI vdW heterostructure. Schematic of the separate synthesis of 2DPI and 2DPBI on the water surface under charged surfactant monolayers (SOS for 2DPI; CTAB for 2DPBI) via sequential monomer adsorption (M1/M2 and M3/M4) and 2D polymerization, followed by manual transfer on Si substrates and subsequent stacking to form the 2DPI–2DPBI vdW heterostructure.

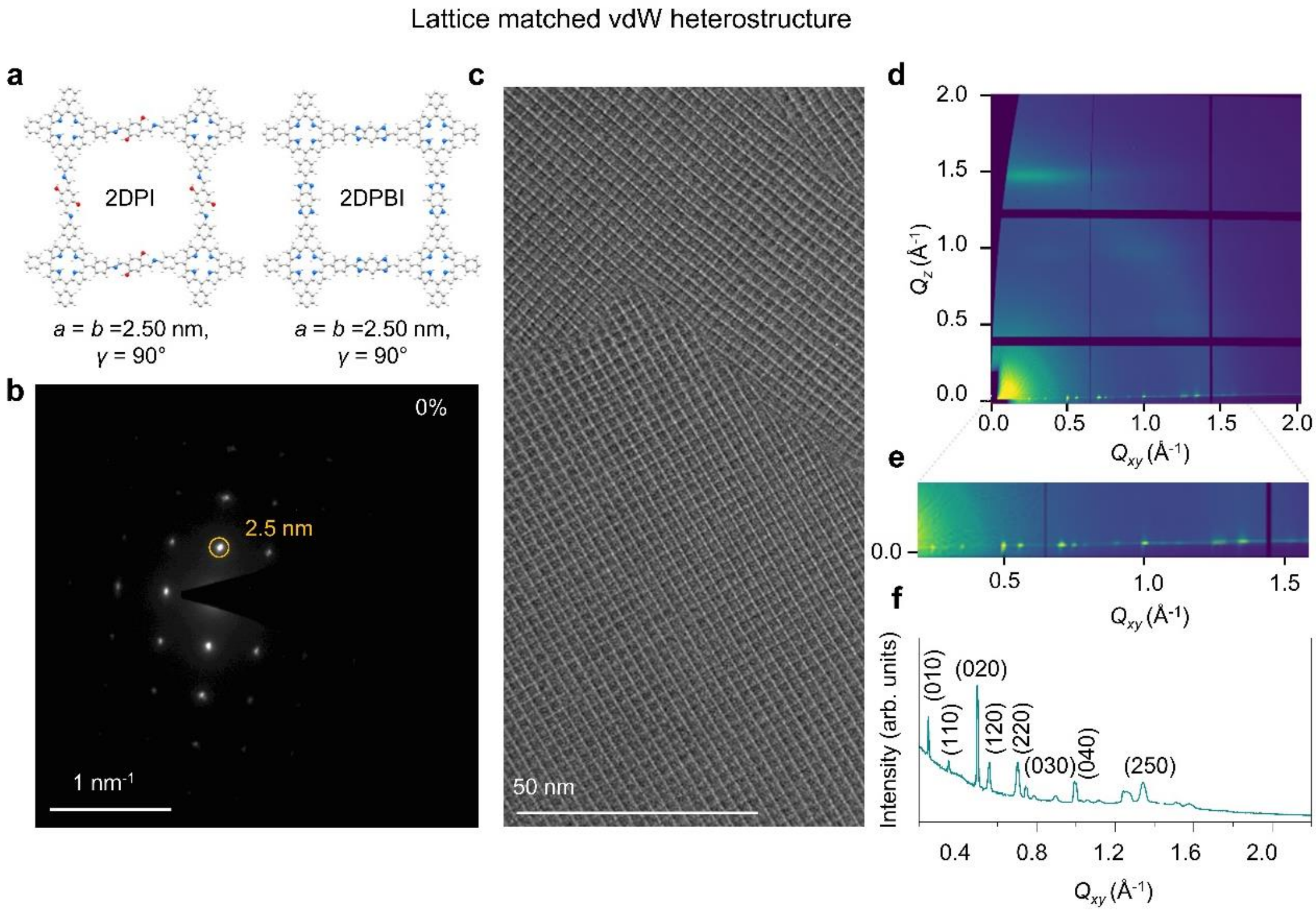

Supplementary Fig. 8 | Structural characterization of the lattice-matched 0% 2DPI–2DPBI 2D polymer vdW heterostructure. (a) Molecular structures of 2DPI and 2DPBI. (b) SAED pattern showing a single set of diffraction spots corresponding to 2.5 nm periodicity. (c) HRTEM image showing ordered lattice domains. (d,e) GIWAXS pattern and enlarged in-plane region. (f) Integrated in-plane GIWAXS profile near $Q_z = 0$, showing the indexed Bragg reflections.

We classify the 2D polymer vdW heterostructures into three types: lattice-matched 0%, small-mismatch 1.6%, and large-mismatch 18.0%. The lattice-matched 0% 2DPI–2DPBI vdW heterostructure was already characterized in the original manuscript and serves as the reference system. Both 2DPI and 2DPBI have identical square lattice parameters of a = b = 2.50 nm, γ = 90°. Therefore, their diffraction peaks overlap. The SAED pattern shows a single set of bright diffraction spots corresponding to the 2.50 nm lattice periodicity, and GIWAXS/GIXD shows one set of in-plane Bragg peaks at the same Q_{xy} positions, without peak splitting. These results confirm coherent lattice matching in the 0% 2DPI–2DPBI vdW heterostructure.

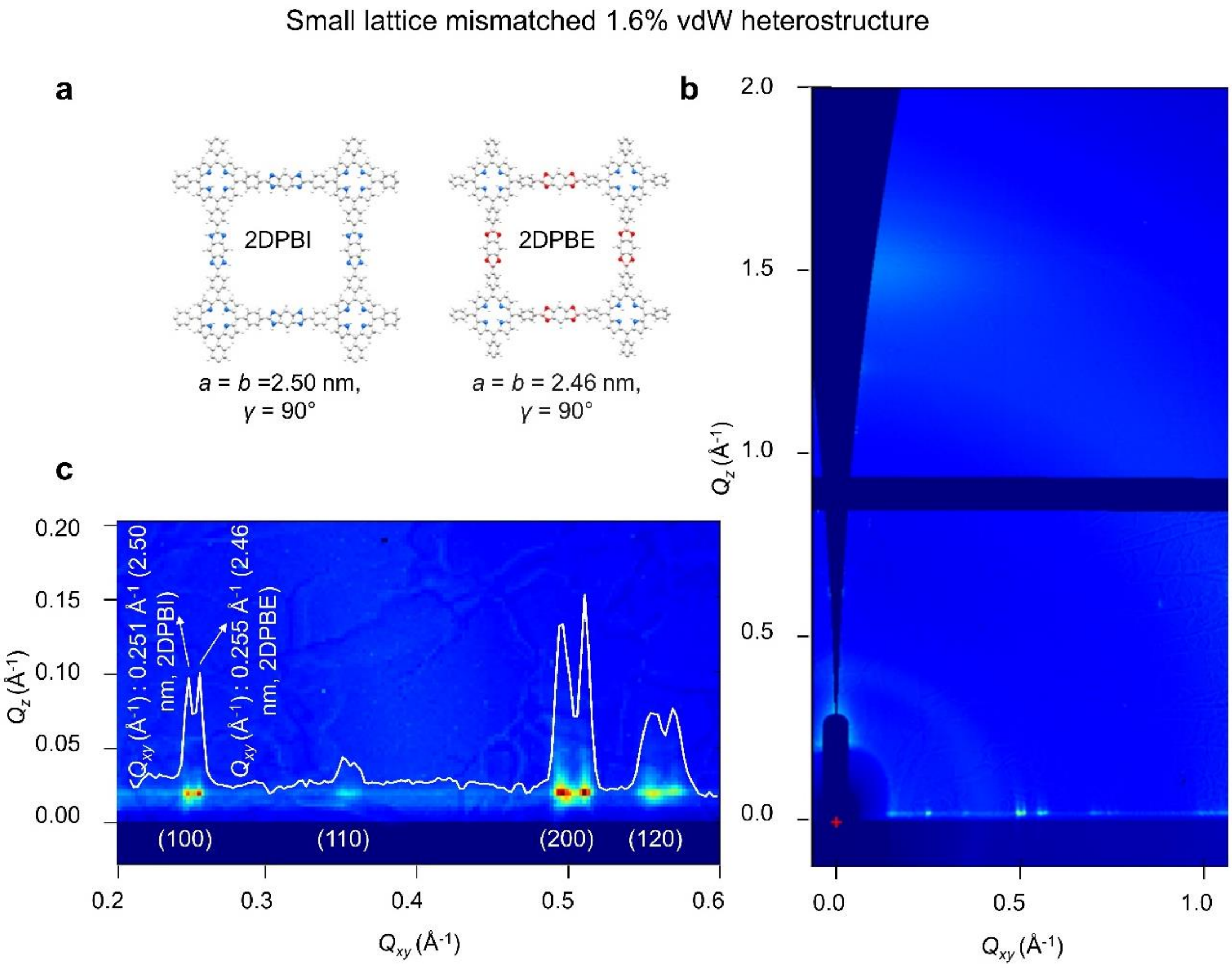

Supplementary Fig. 9 | GIWAXS characterization of the small-mismatch 1.6% 2DPBI–2DPBE 2D polymer vdW heterostructure. (a) Molecular structures of 2DPBI (a = b = 2.50 nm, γ = 90°) and 2DPBE (a = b = 2.46 nm, γ = 90°). (b) 2D GIWAXS pattern. (c) Enlarged in-plane GIWAXS region near $Q_z = 0$, showing split (100) peaks at $Q_{xy} = 0.251$ and 0.255 $Å^{-1}$, corresponding to lattice periodicities of 2.50 and 2.46 nm, respectively, with the splitting retained at higher-order reflections.

For the small-mismatch 1.6% 2DPI–2DPBE vdW heterostructure, the two 2D polymers have slightly different lattice parameters: a = b = 2.50 nm for 2DPI and a = b = 2.46 nm for 2DPBE. Because of this small lattice difference, the diffraction peaks split into two closely spaced peaks rather than appearing as a single peak. The GIWAXS image shows two distinct peaks at Q_{xy} = 0.251 and 0.255 $Å^{-1}$, corresponding to the (100) lattice periodicities with spacings of 2.50 and 2.46 nm, respectively. The same peak separation with double the distance is therefore also observed for the (200) reflections at $Q_{xy} \approx 0.503$ and 0.511 $Å^{-1}$. Consistently, SAED shows closely spaced double spots corresponding to 2.50 and 2.46 nm, confirming the coexistence of both lattices in the small-mismatch 1.6% vdW heterostructure. HRTEM further shows continuous ordered crystalline domains without pronounced warping of the 2D polymer layers. A few isolated knot-like defects are observed, as marked by the yellow arrows, while large-area HRTEM images show no amorphous regions at the domain boundaries.

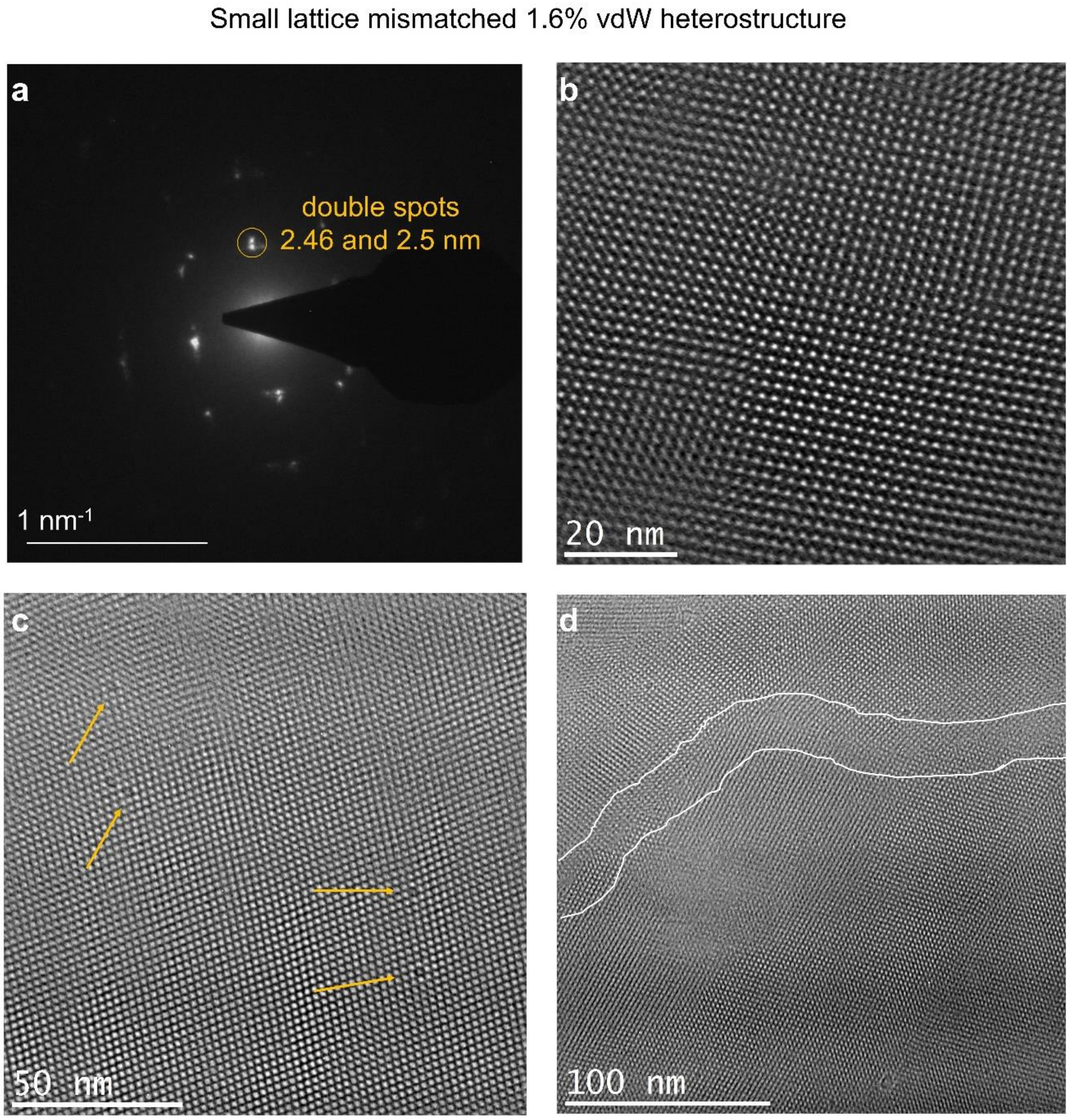

Supplementary Fig. 10 | SAED and HRTEM characterization of the small-mismatch 1.6% 2DPBI–2DPBE 2D polymer vdW heterostructure. (a) SAED pattern showing closely spaced double spots corresponding to 2.50 and 2.46 nm lattice periodicities. (b) HRTEM image showing continuous ordered crystalline domains without pronounced warping of the 2D polymer layers. (c) HRTEM image showing a few isolated knot-like defects, marked by yellow arrows. (d) Large-area HRTEM image showing continuous crystalline domains without amorphous regions at the domain boundaries.

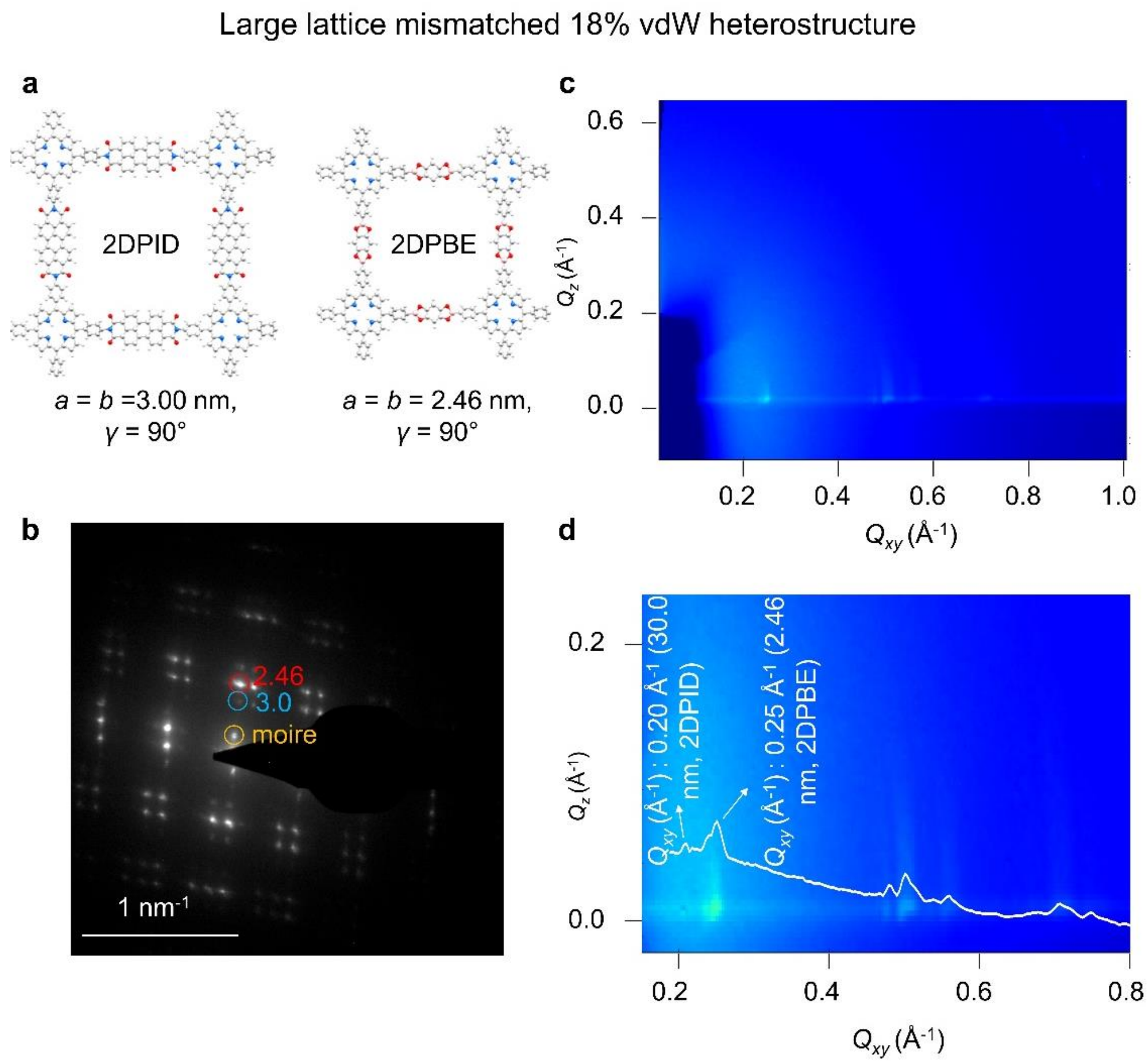

Supplementary Fig. 11 | SAED and GIWAXS characterization of the larger-mismatch 18.0% 2DPID–2DPBE 2D polymer vdW heterostructure. (a) Molecular structures of 2DPID (a = b = 3.00 nm, γ = 90°) and 2DPBE (a = b = 2.46 nm, γ = 90°). (b) SAED pattern showing double spots corresponding to 3.00 and 2.46 nm lattice periodicities, together with distinct Moiré reflections. (c) 2D GIWAXS pattern. (d) Enlarged in-plane GIWAXS region near $Q_z = 0$, showing reflections at $Q_{xy} \approx 0.20$ $Å^{-1}$ and 0.25 $Å^{-1}$, assigned to 2DPID and 2DPBE, respectively.

For the large-mismatch 18.0% 2DPID–2DPBE vdW heterostructure, the two 2D polymers have distinct lattice parameters: a = b = 3.00 nm for 2DPID and a = b = 2.46 nm for 2DPBE. SAED shows a characteristic two sets of diffraction pattern with reflections from both constituent 2D polymers, corresponding to lattice periodicities of approximately 3.00 and 2.46 nm. Distinct Moiré reflections are also observed, providing direct evidence of long-range interference between the mismatched lattices. GIWAXS further shows two in-plane reflections at $Q_{xy} \approx 0.20$ $Å^{-1}$ and $Q_{xy} \approx 0.25$ $Å^{-1}$, assigned to 2DPID and 2DPBE, respectively. Large-area HRTEM images show distinct crystalline domains, with Moiré contrast observed in all of them, as highlighted in yellow. This confirms long-range interference between the two stacked 2D polymer lattices. Higher-magnification HRTEM images reveal wrinkling/warping of the 2D polymer layers, indicating out-of-plane deformation. These deformations provide a strain-relief pathway, allowing the stacked 2D polymer lattices to release interfacial stress through local curvature. In addition, long-range edge dislocations are observed, further indicating dissipation of the mechanical mismatch within the heterostructure. Together, the reciprocal-space

signatures from SAED/GIWAXS and the real-space HRTEM features suggest that strain in the larger-mismatch 18.0% 2D polymer vdW heterostructure is relaxed through a combination of local lattice distortion, dislocation formation, and out-of-plane deformation.

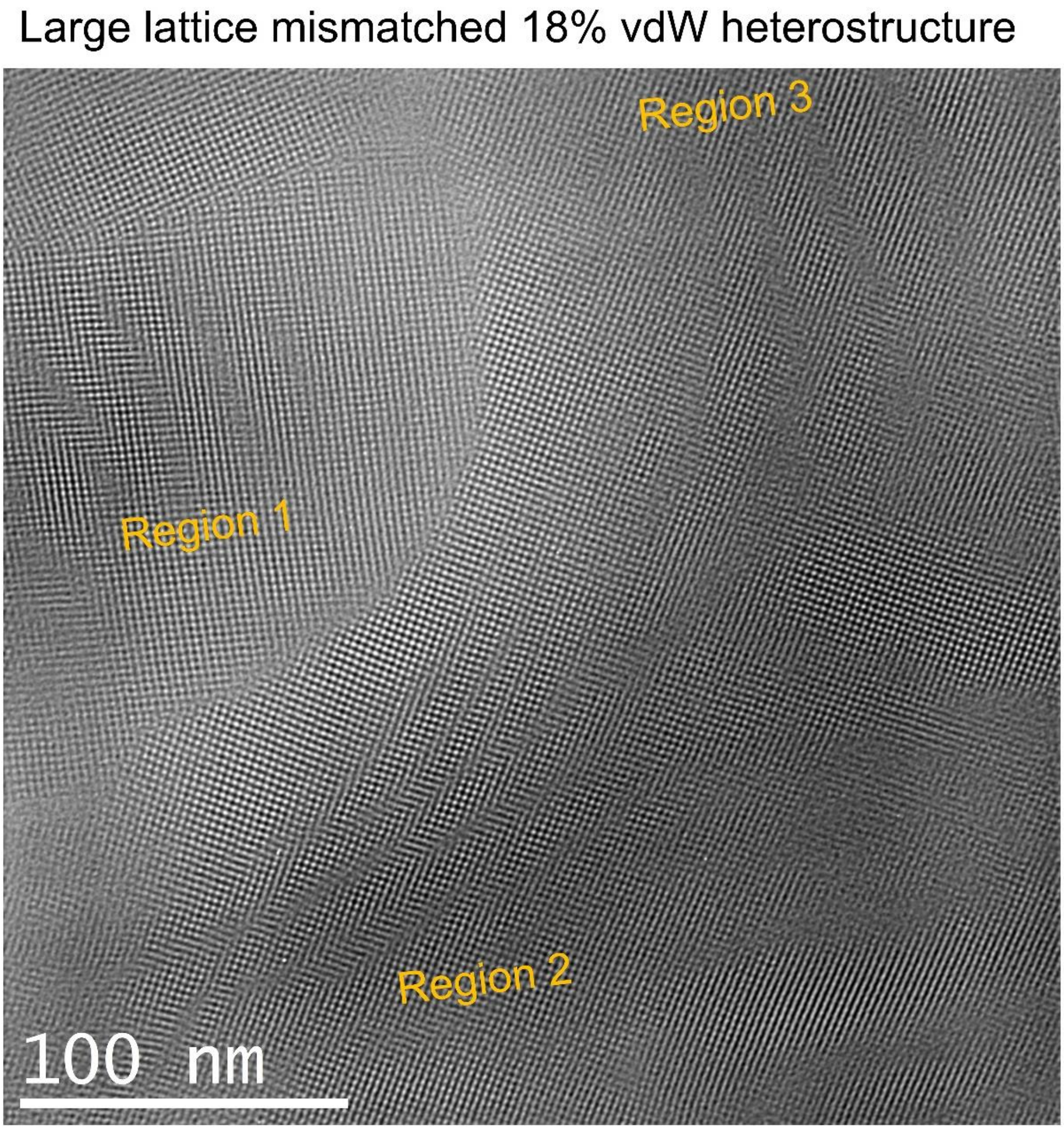

Supplementary Fig. 12 | HRTEM characterization of the larger-mismatch 18.0% 2DPID–2DPBE 2D polymer vdW heterostructure. Large-area HRTEM image showing different crystalline domains (regions 1–3), with Moiré contrast observed in all domains, confirming long-range interference between the two stacked 2D polymer lattices.

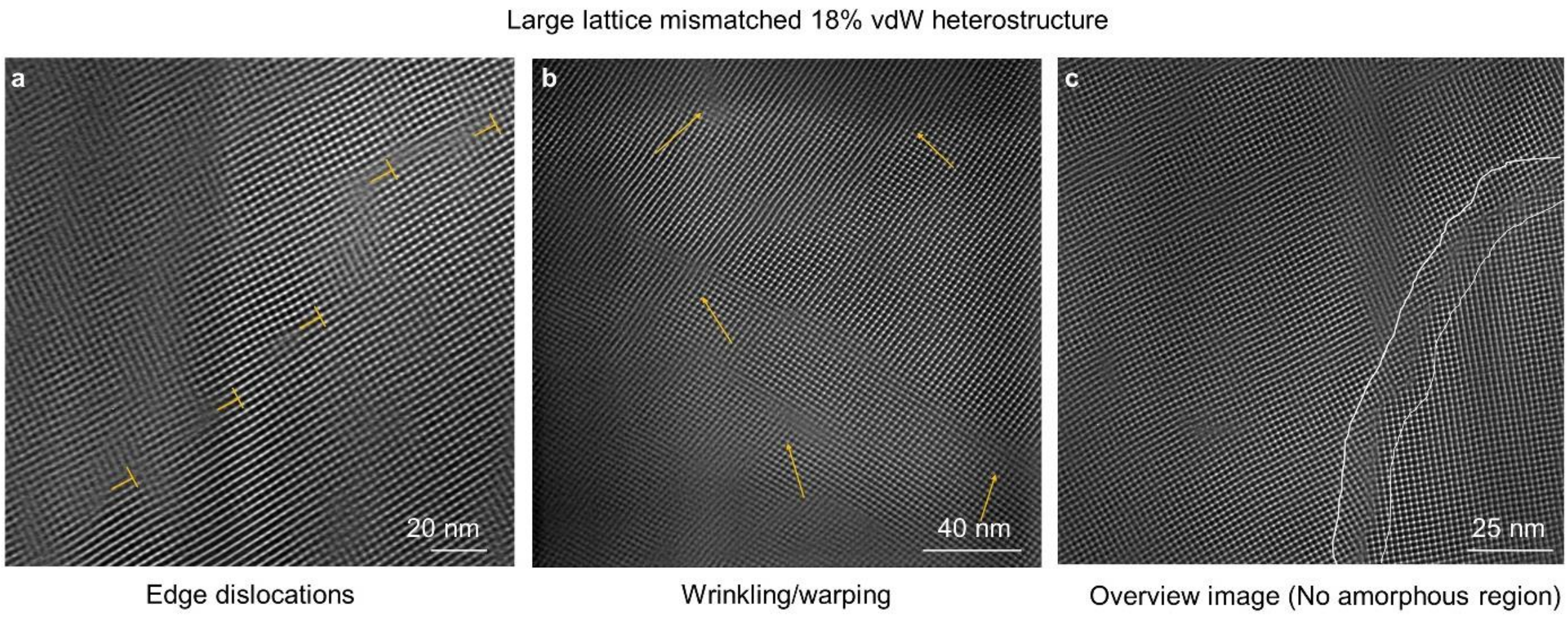

Supplementary Fig. 13 | HRTEM characterization of the larger-mismatch 18.0% 2DPID–2DPBE 2D polymer vdW heterostructure. (a) HRTEM image showing long-range edge dislocations. (b) HRTEM image showing wrinkling/warping of the 2D polymer layers. (c) Large-area HRTEM overview image showing continuous crystalline domains without amorphous regions at the domain boundaries.

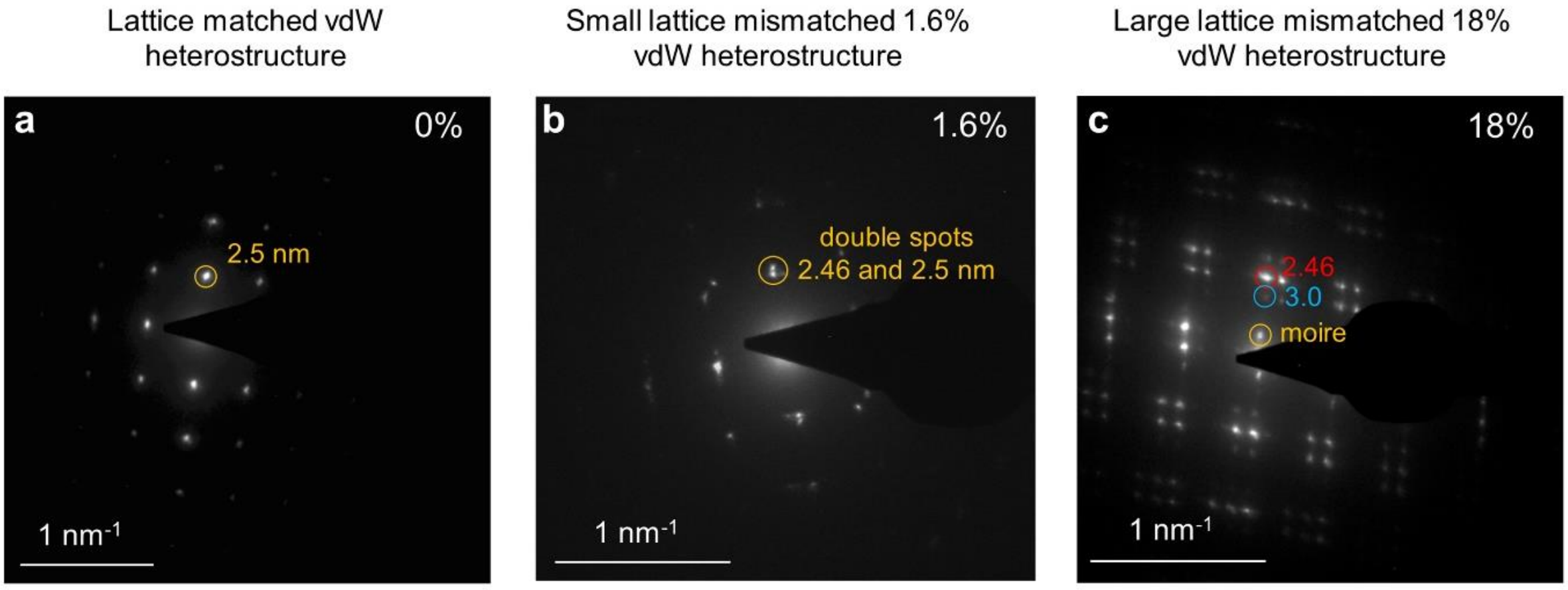

Supplementary Fig. 14 | SAED comparison of lattice-matched and lattice-mismatched 2D polymer vdW heterostructures. (a) Lattice-matched 0% vdW heterostructure showing a single set of diffraction spots corresponding to 2.50 nm periodicity. (b) Small-mismatch 1.6% vdW heterostructure showing closely spaced double spots corresponding to 2.46 and 2.50 nm periodicities. (c) Larger-mismatch 18.0% vdW heterostructure showing clearly separated diffraction spots corresponding to 2.46 and 3.00 nm periodicities, together with distinct Moiré reflections.

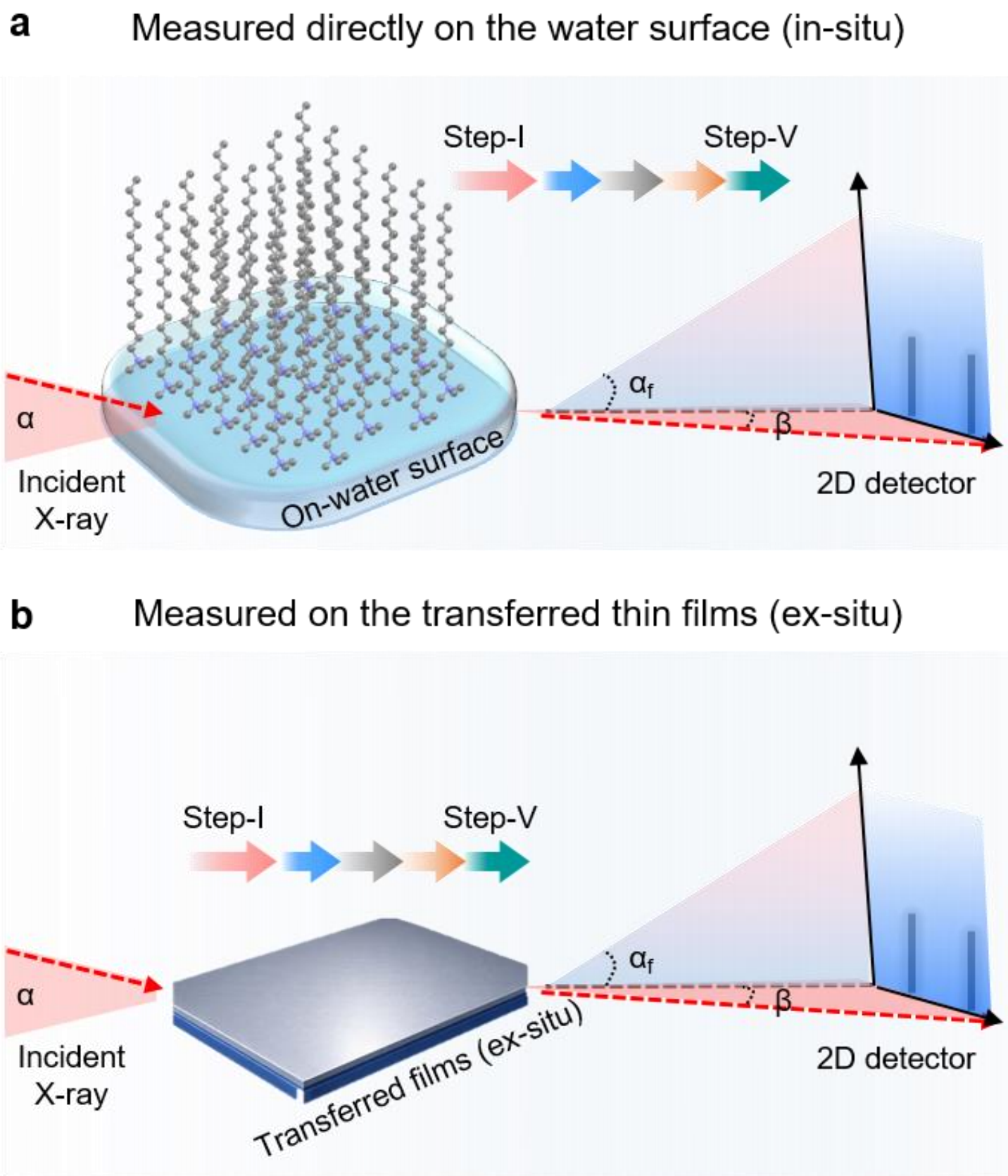

Supplementary Fig. 15 | Grazing-incidence X-ray diffraction/scattering geometries for in situ and ex situ measurements. a, In-situ acquisition directly on-water surface: an incident X-ray beam impinges at grazing angle α, and the scattered signal is collected on a 2D detector, defining the exit angle α_f and in-plane angle β. **b**, Corresponding ex-situ geometry for transferred thin films measured on a solid support. Measurements were performed sequentially after each processing step (I–V).

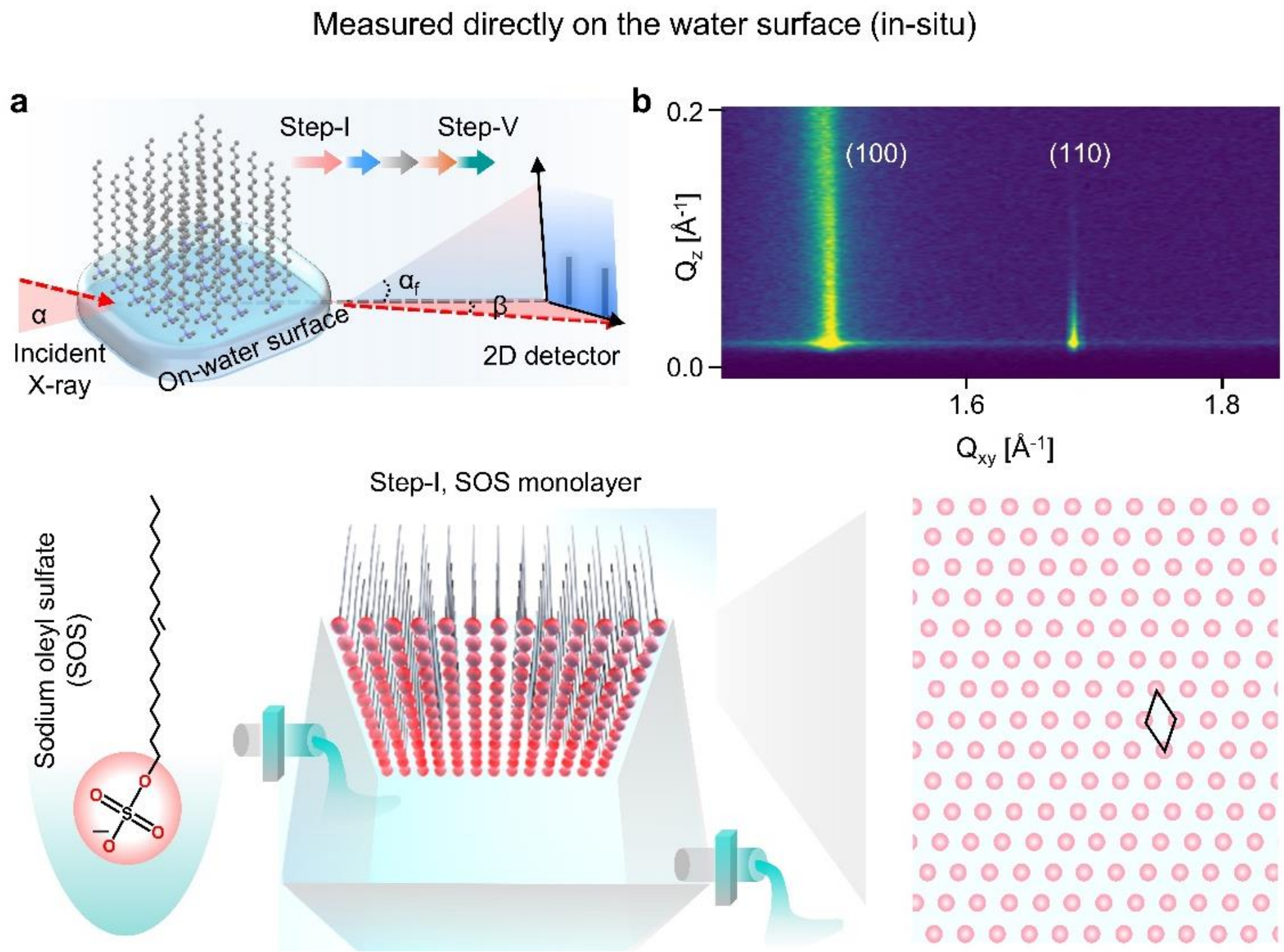

Supplementary Fig. 16 | Surfactant-assisted interfacial ordering revealed by on-water GIWAXS. Schematic representation of the in-situ GIXD measurement setup directly on the water surface, which is very sensitive towards the in-plane molecular structure of monolayers. Therefore, in-situ step by-step GIXD was performed directly on the water surface, which commences from step-I, i.e., the surfactant monolayer formation. Surfactants are amphiphilic molecules with a long hydrocarbon tail and a polar head group, which adsorb at the air-water interface with the hydrophilic heads towards the water surface and the hydrophobic tails towards the air. The corresponding measured GIXD scattering profile of the SOS surfactant exhibited distinct and sharp diffraction peaks which correspond to a unit cell with a = 4.5 Å, b = 4.5 Å, and γ = 67.52°.

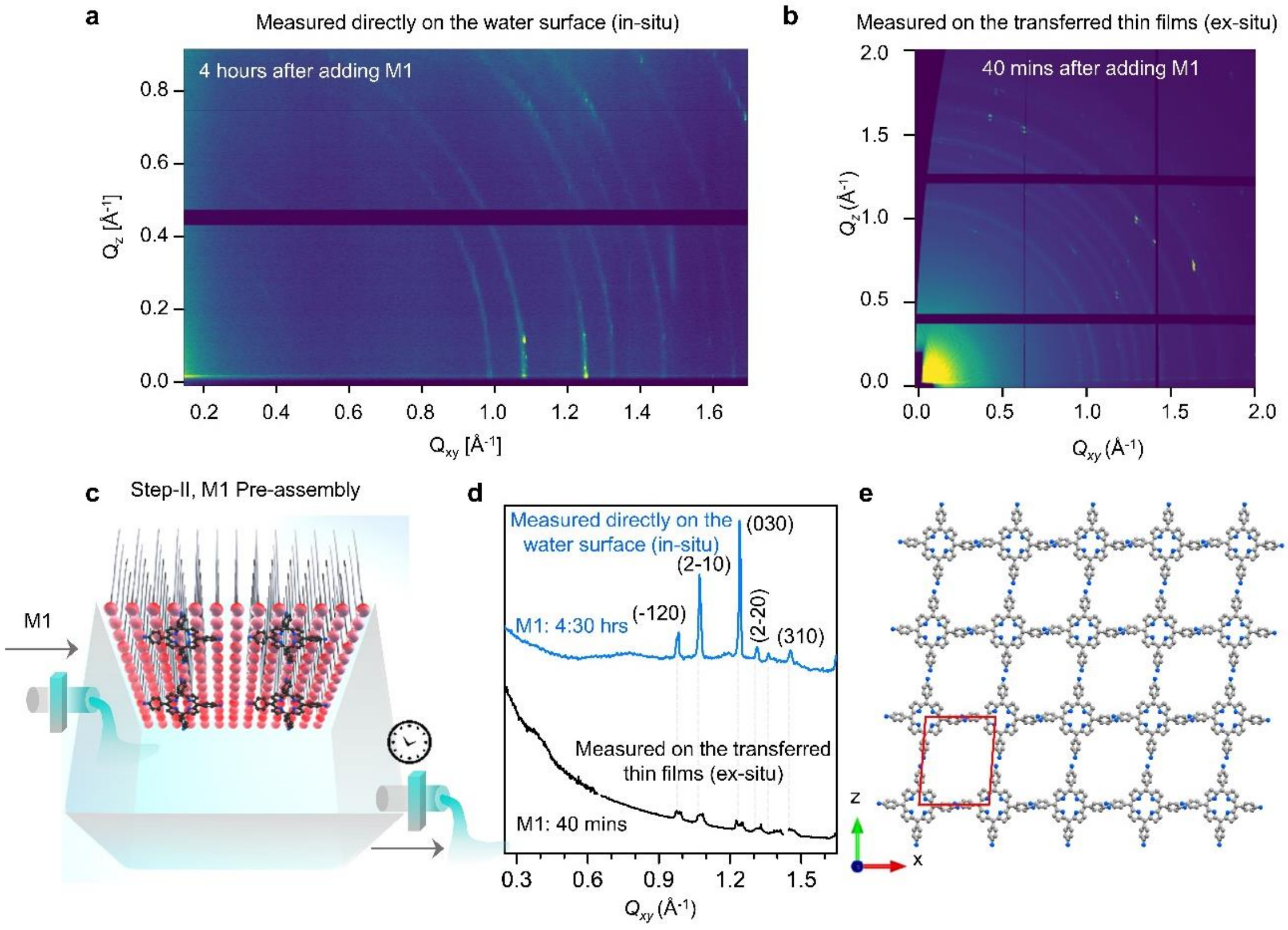

Supplementary Fig. 17 | Interfacial pre-organization of M1 during Step II resolved by in-situ GIXD and ex-situ GIWAXS. **a**, 2D GIXD pattern measured directly on the water surface (in-situ) 4:30 h after addition of M1 to the subphase. **b**, Corresponding pattern from a transferred thin film (ex-situ; shown 40 min after M1 addition), exhibiting pronounced diffraction rings. **c**, Schematic of Step-II pre-assembly of M1 beneath the surfactant monolayer on the water surface and subsequent film transfer for ex-situ characterization. **d**, Comparison of Bragg peak positions extracted from the in-situ (blue) and ex-situ (black) measurements, showing close agreement in reciprocal space; dashed lines indicate peak positions. **e**, 2D lattice model consistent with the indexed reflections, yielding a 2D unit cell of a = 13.22 Å, b = 15.29 Å, and γ = 85.24°.

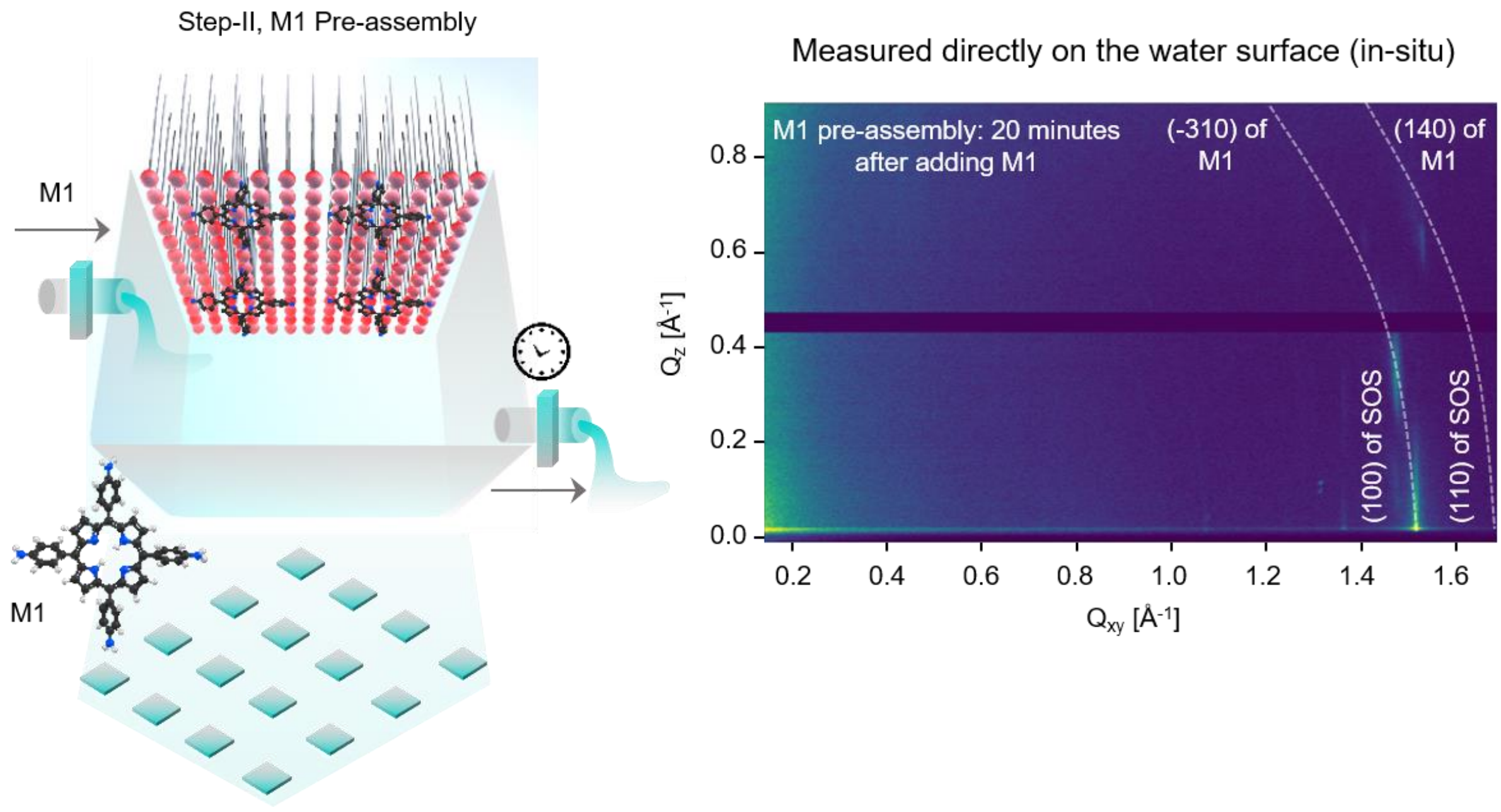

Supplementary Fig. 18 | Epitaxial registry between the SOS lattice and the M1 pre-assembly on the water surface. Schematic of M1 diffusion and pre-assembly beneath the SOS monolayer on the water surface. In-situ GIXD tracks the characteristic SOS (100) Bragg peak at $Q_{xy} = 1.51$ Å^{-1} before and after addition of R1 to the water subphase. Upon M1 adsorption, the emerging M1 diffraction rings indexed as (−310) and (140) coincide with the SOS (100) and (110) peaks, respectively (dashed guides), consistent with point-on-line epitaxy that establishes one-dimensional registry of the M1 lattice along the SOS [1,0] lattice line.

After extracting the unit cells of the surfactant monolayer (a = 4.5 Å, b = 4.5 Å, and γ = 67.52°) from the in-situ GIXD measurements and the M1 pre-assembly (a = 13.22 Å, b = 15.29 Å, and γ = 85.24°) from both the in-situ and ex-situ GIWAXS measurements we observed coincidence of some of the diffraction signals (see Figs. S16-S18) of the two layers indicating a potential epitaxial alignment between the two lattices. This was expected based on our previous studies on the pre-assembly of a very similar porphyrin molecule [4-(5,10,15-triphenylporphyrin-20-yl)aniline] underneath SOS[10]. For the epitaxial alignment calculations we used the "small" unit cell (a = 4.5 Å, b = 4.5 Å, and γ = 67.52°) for the SOS surfactant due to the fact that the electrostatic interaction between the charged head group of the surfactant and the protonated M1 molecule is relevant for the assembly. A more detailed discussion on that rationale can be found in Prasoon et al[10]. To determine the most probable type of epitaxy, we use a software which searches for epitaxial configurations between the two lattices by varying the azimuthal angle between them and letting the lattices "breathe" to look for known types of epitaxial registry. This is based on the assumption that for large, highly crystalline domains of an adlayer on a crystalline substrate, only epitaxial configurations produce energetic gain over random arrangements that ignore the substrate's crystallinity. For the calculations we defined the surfactant as the substrate (fixed lattice) and M1 as the adsorbate (slightly flexible lattice) with allowed tolerances of 0.05 Å for the lattice vectors and 0.5° for γ. The software calculates the algebraic 2x2 matrix that relates the two lattices to each other. This matrix is then used to test all possible azimuthal alignments between the two lattices by varying the lattice of M1 within the previously stated borders. The algorithm compares the two reciprocal lattices for each angle and checks for any matches and the type of potential epitaxial relationship (commensurate, point-on-line, line-on-line) based on the work from Mannsfeld et al.[11,12]. In our case, the software found multiple epitaxial configurations within the set restrictions. The solution with the highest confidence score (0.999) showed a azimuthal rotation of the M1 lattice (a = 13.27 Å, b = 15.34 Å, and γ = 85.74°) of -2.5° in respect to the surfactant lattice with an epitaxy matrix [[-3.00 0.14],[1.00 -3.66]]. A visual representation of the arrangement is shown in Fig. S19. It can be seen that the epitaxy matrix has one column with only integer values indicating that the observed configuration is a point-on-line epitaxy. In our case this means each M1 molecule (lattice point) lies directly on primitive lattice lines of SOS. It has been shown in the literature that point-on-line epitaxy in systems based on van der Waals interactions is energetically favorable compared to a random azimuthal orientation[12].

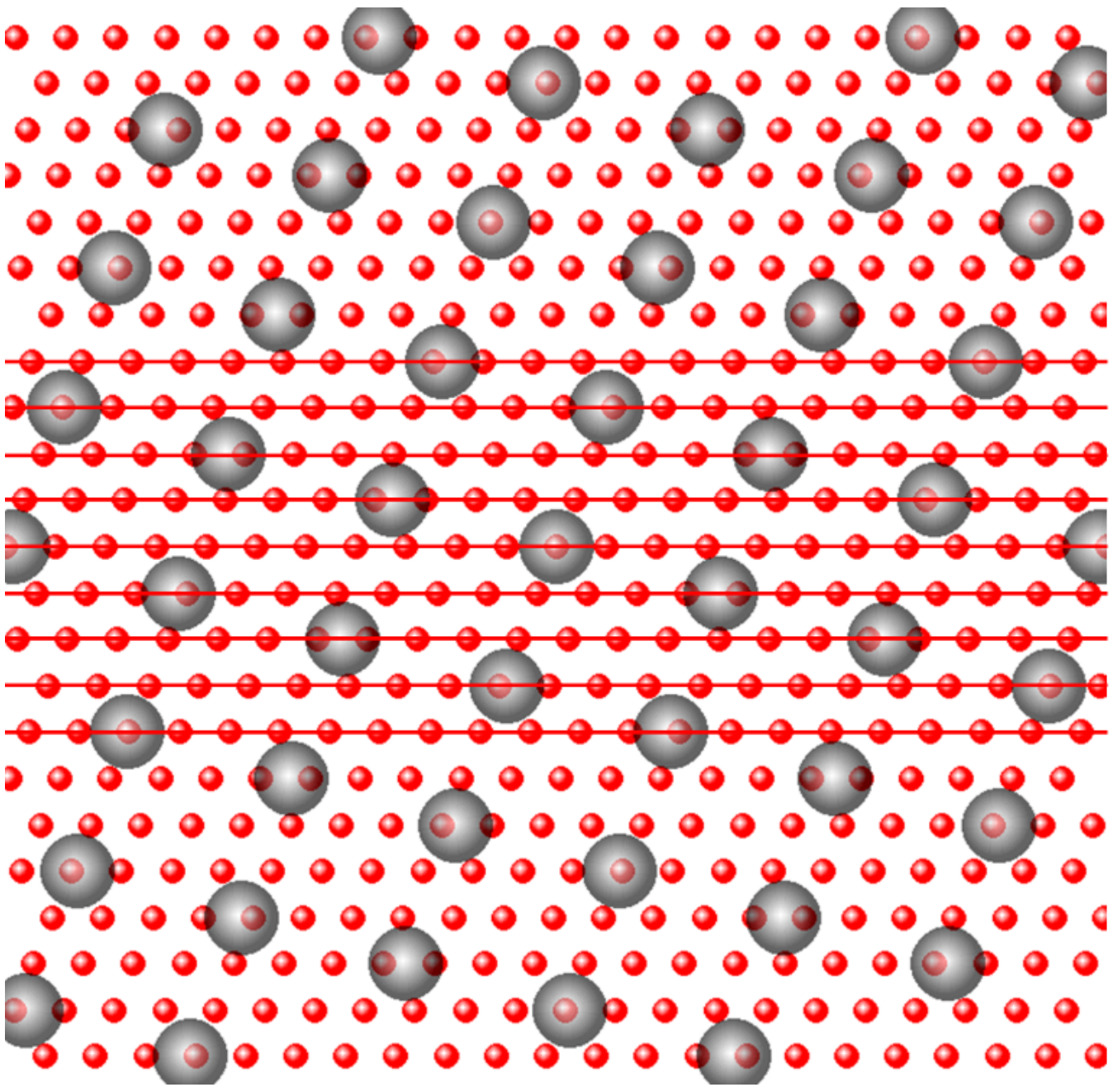

Supplementary Fig. 19 | Illustration of the epitaxial relationship between the SOS lattice and the M1 pre-assembly on the water surface. Schematic of the lattices of SOS (red circles) and the epitaxial grown M1 (gray circles). The red lines act as guides for the eyes to emphasize the point-on-line epitaxy of the two lattices.

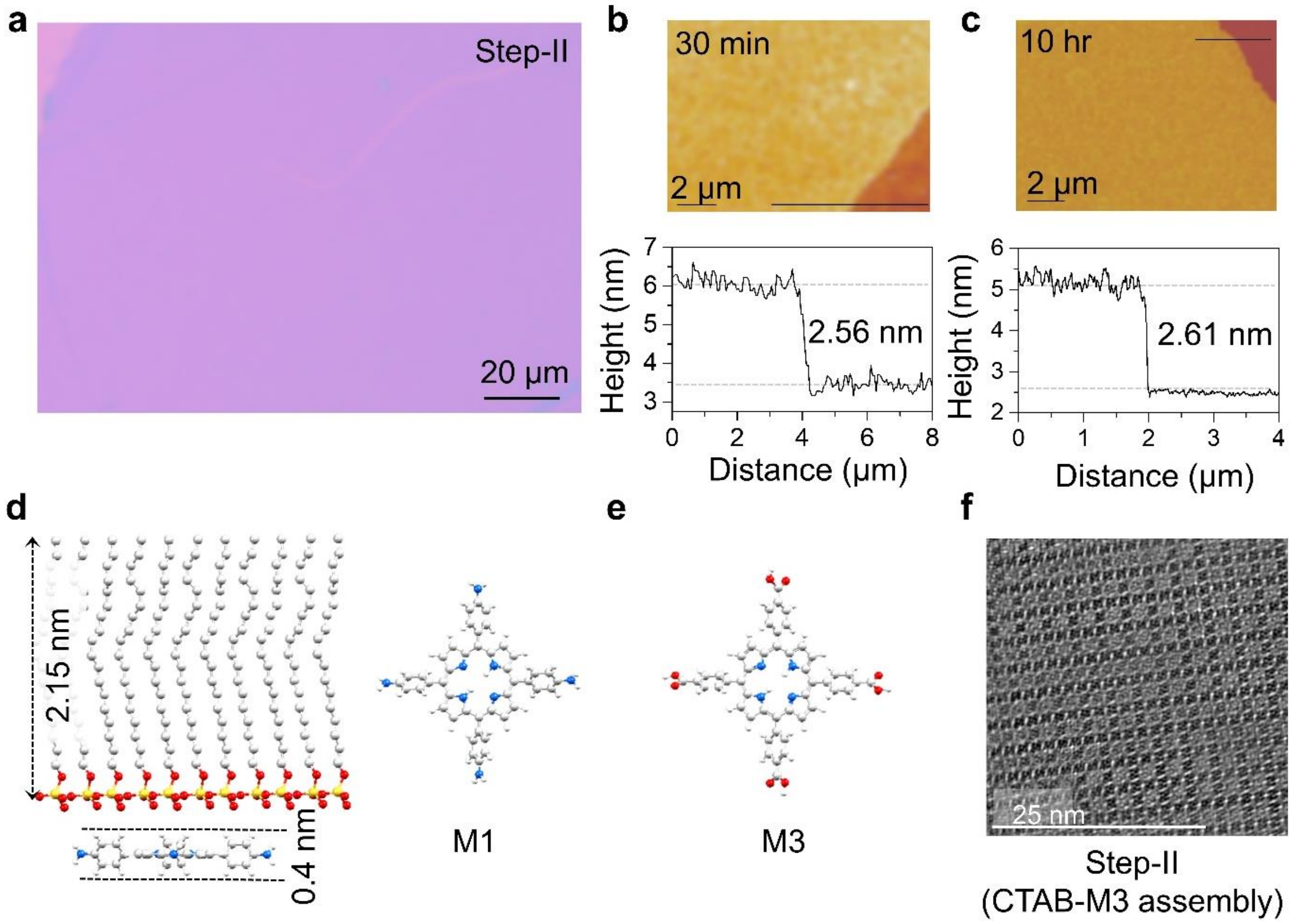

Supplementary Fig. 20 | Step-II monomer pre-assembly underneath charged surfactant monolayers. (a) Optical microscopy image of the SOS–M1 assembly formed in Step-II. (b,c) AFM images and height profiles of the SOS–M1 assembly after M1 adsorption for 30 min and 10 h, showing thicknesses of 2.56 and 2.61 nm, respectively. (d) Schematic thickness comparison of the SOS monolayer and M1/M3 molecules. (e) HRTEM image of the SOS–M1 assembly in Step-II, adapted from ref. 45 (Nat. Chem. 11, 994–1000, 2019). (f) HRTEM image of the CTAB–M3 assembly in Step-II.

The ring-like GIWAXS feature of the Step-II M1 assembly reflects an azimuthal distribution of many small crystalline domains over the macroscopic X-ray beam area, rather than molecular-level disorder within each domain. In GIWAXS, the diffraction signal is averaged over a large area on the water surface, typically on the scale of a few centimeters (~2 cm). In Step-II, the SOS–M1 assembly is mainly a monolayer/few-layer pre-assembly with relatively low scattering density. Therefore, even if each local domain is ordered, the presence of many differently oriented domains within the large GIWAXS footprint gives ring-like diffraction features.

In contrast, HRTEM probes local nanoscale regions. The transferred SOS–M1 (Nat. Chem. 11, 994–1000 (2019)) and CTAB–M3 assemblies show well-resolved square molecular lattices over local regions, confirming local crystalline ordering in Step-II. Therefore, the cartoons represent the local ordered monomer arrangement derived from HRTEM, whereas the GIWAXS rings reflect the macroscopic orientational distribution of such ordered domains over the large beam area. To further support Step-II pre-assembly, AFM measurements of the transferred SOS–M1 assembly show thicknesses of 2.56 nm after 30 min and 2.61 nm after 10 h. These values match the combined thickness

of the SOS monolayer and M1 molecule, supporting monolayer/few-layer M1 pre-assembly beneath the SOS surfactant monolayer.

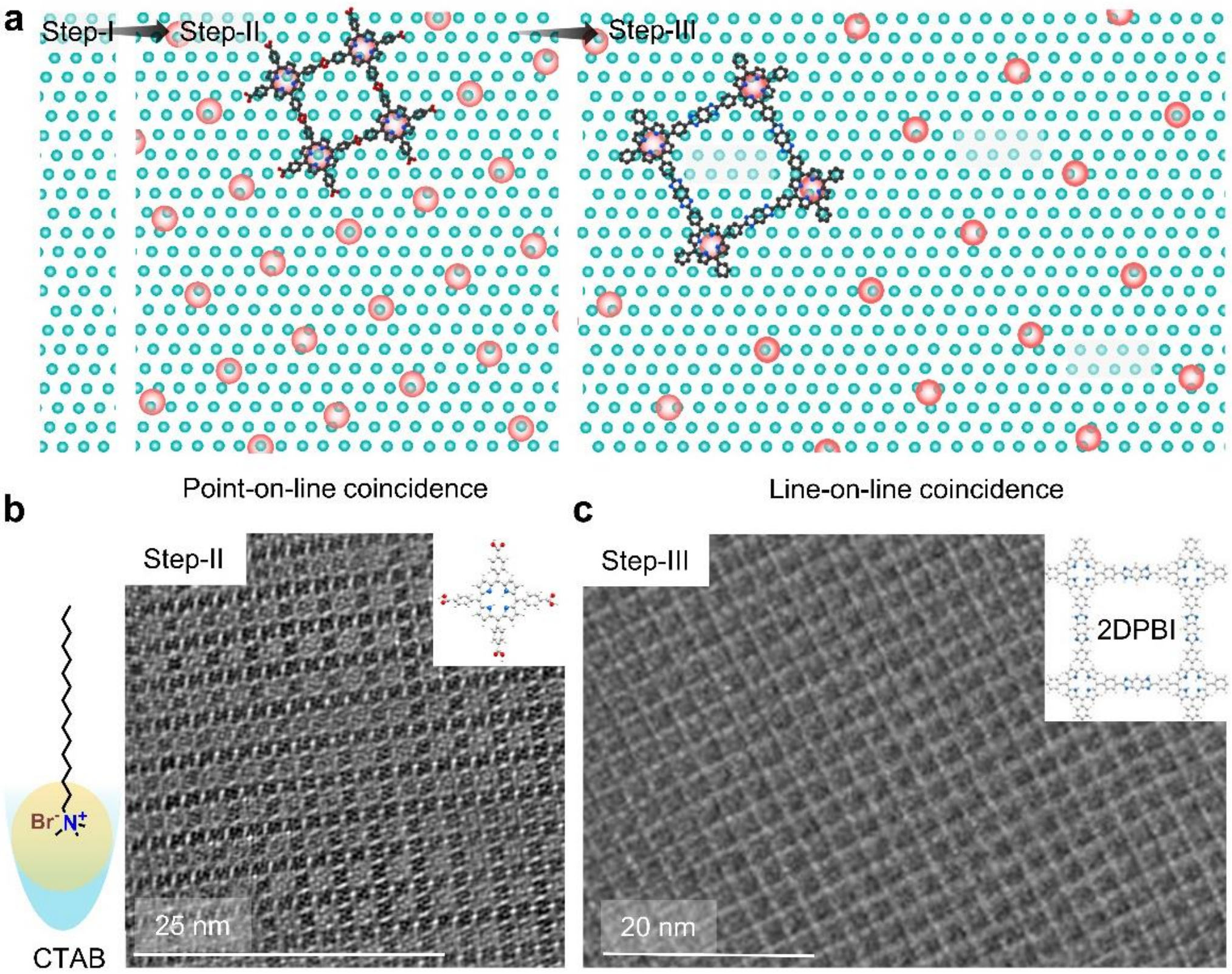

Supplementary Fig. 21 | Cationic surfactant CTAB-guided epitaxial pre-assembly of monomers and 2DPBI formation. (a) Schematic diagram of the CTAB-guided epitaxial growth process, showing the transition from point-on-line coincidence during M3 pre-assembly to line-on-line coincidence after 2DPBI formation. (b) HRTEM image of the pre-assembled CTAB–M3 layer (Step-II). (c) HRTEM image of the resulting 2DPBI film (Step-III).

The SOS surfactant contains an anionic sulfate headgroup, which promotes electrostatic adsorption of protonated M1 under acidic conditions. In addition, the crystalline SOS monolayer serves as an ordered template for the epitaxial pre-organization of M1. To further examine the role of surfactant charge, we performed additional GIWAXS experiments using the cationic surfactant CTAB. Under basic conditions, M3, 5,10,15,20-tetrakis(4-carboxyphenyl)porphyrin, carries negatively charged carboxylate groups and therefore interacts electrostatically with the cationic CTAB monolayer. CTAB forms a crystalline monolayer on the water surface with a = b = 4.49 Å and γ = 67.52°. Upon M3 addition, an ordered molecular assembly is observed with a = 17.53 Å, b = 17.49 Å, and γ = 89.40°. The epitaxial alignment corresponds to point-on-line epitaxy, in which M3 lattice points register along the [1,0] lattice lines of the CTAB monolayer. Upon addition of M4 (1,2,4,5-benzenetetramine), M4 reacts with the pre-assembled M3 layer to form 2DPBI. The resulting diffraction peaks correspond to a square 2D polymer lattice with a = b = 25.1 Å and γ = 90.00°. The epitaxial relationship then evolves into line-on-line epitaxy, in which extended lattice lines of 2DPBI along the [3,–6] direction coincide with the [1,1] crystallographic lines of the CTAB surfactant lattice. This charge-complementarity principle is also supported by our previous model reaction, in which the cationic surfactant CTAB was used to facilitate the adsorption of a negatively charged porphyrin reagent on the water surface, enabling the site-selective imidazole-bond-forming reaction. These results clearly demonstrate that the important factor for the

monomer assembly is the complementary charge on the surfactant compared to the M1 molecule and both anionic and cationic surfactants can be used for the successful synthesis of 2D polymers.

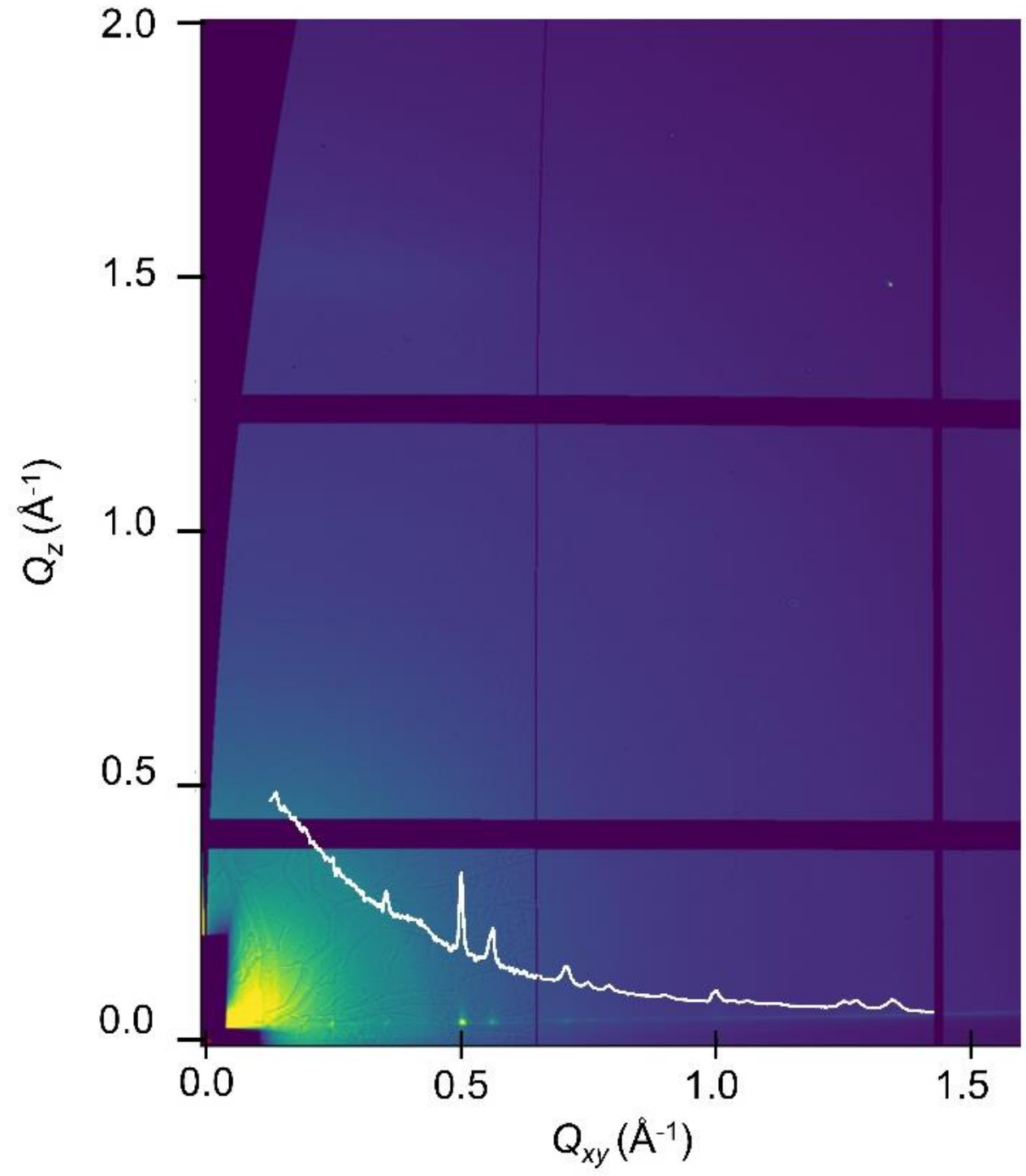

(hk)	Meas. peak [A^{-1}]	Fitted peak [A^{-1}]
(0,1)	0.248	0.250
(0,2)	0.503	0.500
(1,-2)	0.561	0.559
(2,-2)	0.707	0.707
(0,3)	0.752	0.750
(1,-3)	0.792	0.791
(0,4)	1.002	1.000
(2,-4)	1.120	1.118
(1,-5)	1.270	1.275
(2,-5)	1.347	1.346

Supplementary Fig. 22 | 2D polymerization in Step-III monitored by GIWAXS on the water surface. Following the addition of M2 to the water subphase, M2 reacts with the pre-assembled M1 layer on the water surface to drive 2D polymerization and form the 2DPI polymer. The resulting film was transferred on a silicon substrate and measured ex-situ; the GIWAXS pattern collected 18 h after M2 addition exhibits sharp Bragg reflections indexed to a square unit cell (a = b = 25.14 Å, γ = 90.00°). The table shows the measured and fitted peak positions together with the (hk) indices.

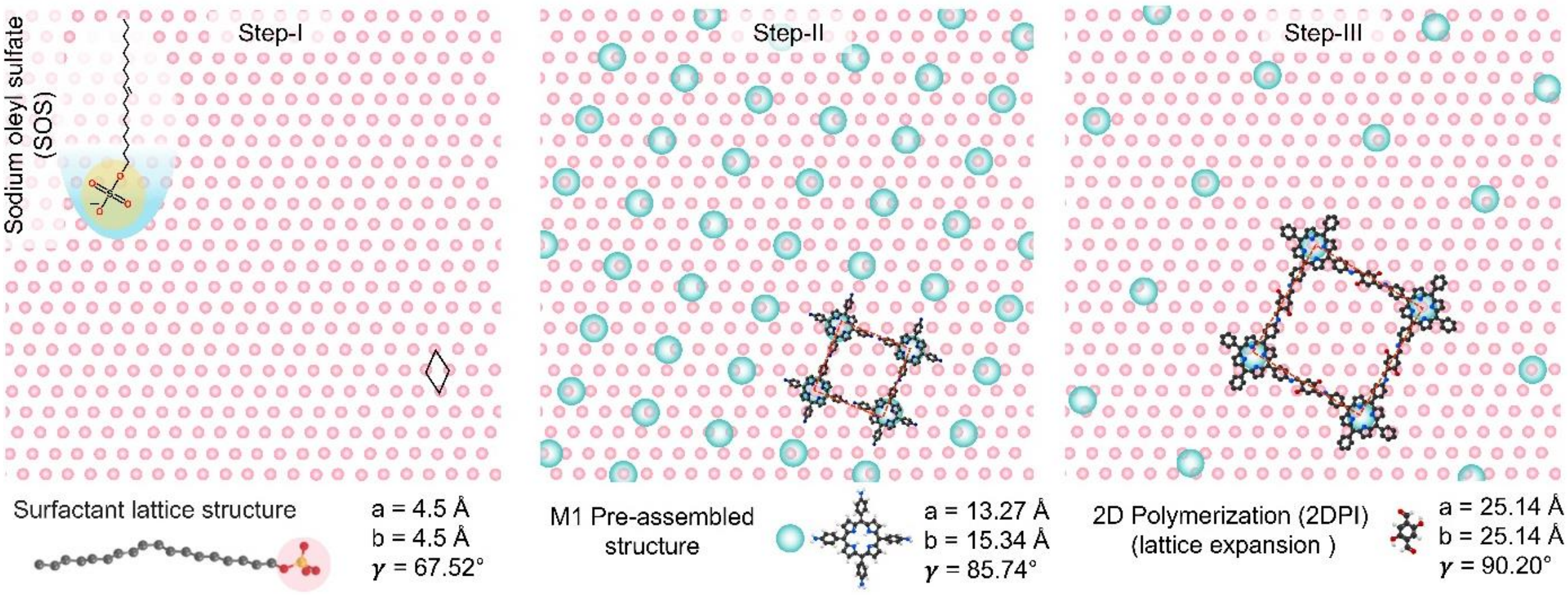

Supplementary Fig. 23 | Lattice expansion from M1 pre-assembly to the 2DPI polymer. Schematic of the stepwise structural evolution on the water surface. Step-I, formation of a crystalline SOS monolayer. Step-II, adsorption and pre-assembly of M1, yielding a 2D lattice with a = 13.27 Å, b = 15.34 Å, and γ = 85.74° (dashed unit cell). Step-III, following addition of M2 to the water subphase, M2 reacts with the pre-assembled M1 layer to drive 2D polymerization and form the 2DPI polymer with an expanded lattice (a = b = 25.14 Å, γ = 90.00°).

With the same approach as for the alignment between the surfactant SOS and M1 we calculated potential epitaxial relationships between SOS and the final polymer 2DPI. For the calculations we again used the SOS lattice (a = 4.5 Å, b = 4.5 Å, and γ = 67.52°) as substrate and the unit cell for 2DPI extracted from the in-situ GIXD and ex-situ GIWAXS measurements (a = 25.14 Å, b = 25.14 Å, and γ = 90°) as adsorbate lattice. For the calculations we once again allowed tolerances of 0.05 Å for the lattice vectors and 0.5° for γ. The solution with the highest confidence (1.0) was found for an epitaxy matrix of [[5.263 0.737],[-2.989 5.989]] and an azimuthal rotation of the 2DPI lattice in respect to the SOS lattice of 7°. The 2D unit cell for this 2DPI lattice was derived to be a = 25.14 Å, b = 25.10 Å, and γ = 90.2°, showing only a small deviation from a perfect square lattice. Figure S16 shows a schematic of the calculated epitaxial relationship. It can be clearly seen that the found configuration represents a line-on-line epitaxy, which is defined as a coincidence between nonprimitive reciprocal lattice vectors of the adsorbate (2DPI) and the substrate (SOS)[13]. In our case this corresponds to the (110) lattice lines of the surfactant and the (630) lines of the polymer. It has been shown in the literature that line-on-line epitaxy in organic-organic heterostructures represents an energetically favorably configuration with a minimum in the adsorbate-substrate interaction potential[13].

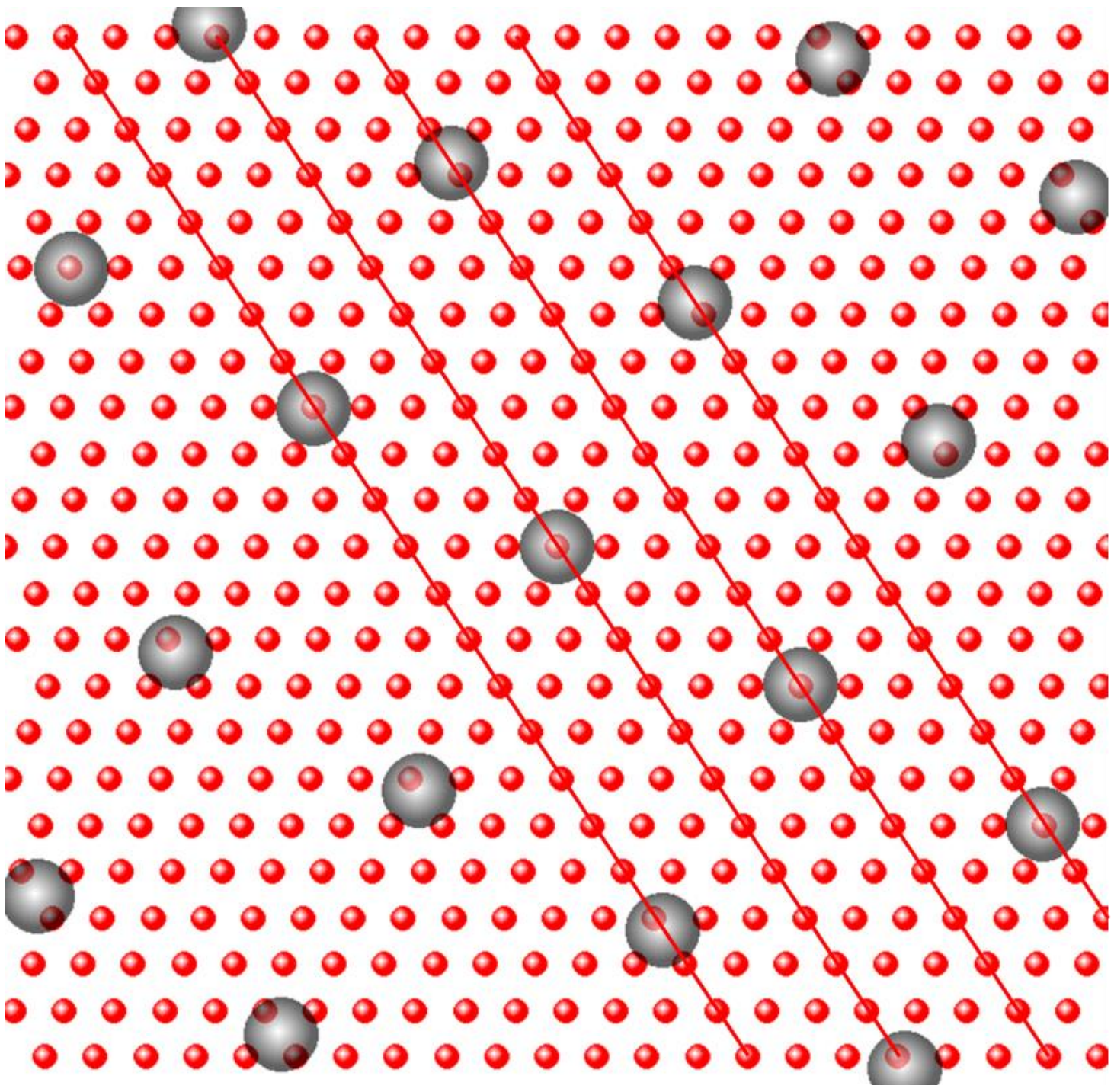

Supplementary Fig. 24 | Illustration of the epitaxial relationship between the SOS lattice and the 2DPI polymer. Schematic of the lattices of SOS (red circles) and the epitaxial grown 2DPI (gray circles). The red lines act as guides for the eyes to emphasize the line-on-line epitaxy of the two lattices.

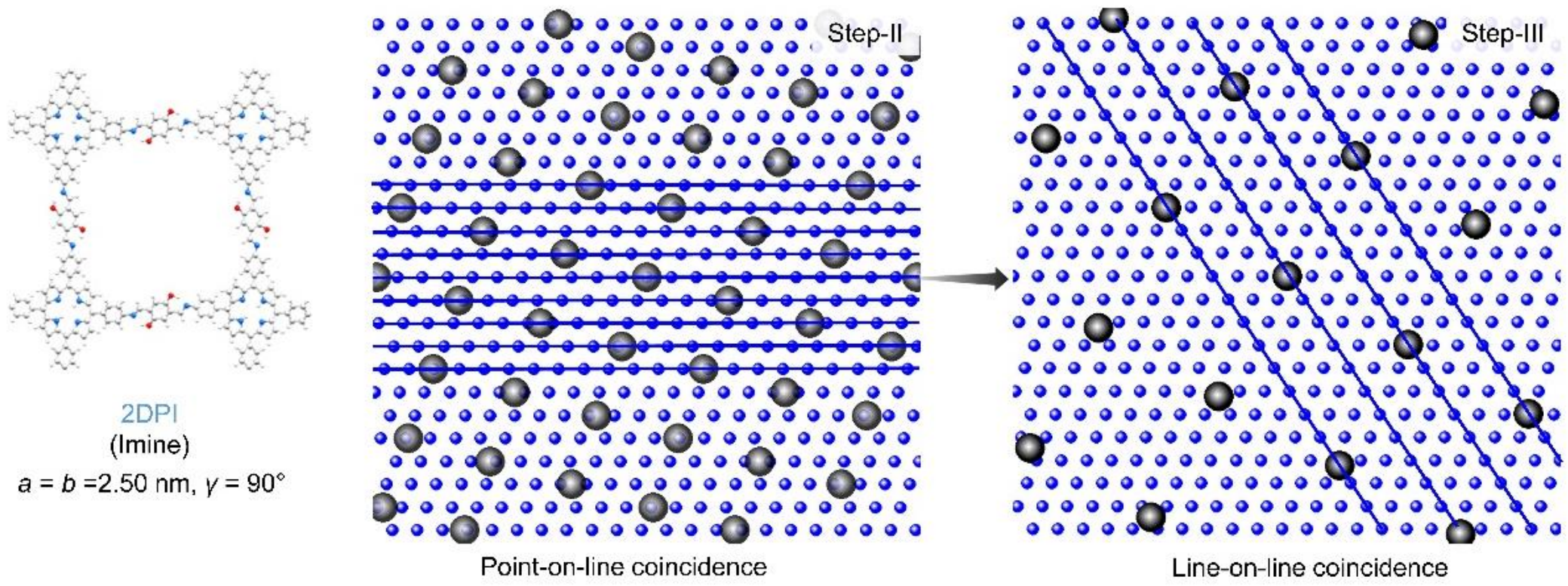

Supplementary Fig. 25 | Epitaxial transition in on-water surface synthesis of 2DPI. Schematic illustration of point-on-line coincidence during M1 pre-assembly in Step-II and line-on-line coincidence during 2DPI formation in Step-III.

The driving force for the transition from point-on-line to line-on-line epitaxy, we analyzed three representative square-lattice 2D polymer systems with different lattice parameters, using both anionic SOS and cationic CTAB surfactants.

Case-I: 2DPI formation with anionic SOS surfactant.

For 2DPI (a = b = 2.50 nm, γ = 90°), the Step-II epitaxial alignment corresponds to point-on-line epitaxy, in which M1 lattice points register along the (1,0) lattice lines of the SOS surfactant lattice. After M2 addition in Step-III, the lattice *expands* from the pre-assembled M1 structure (a = 13.22 Å, b = 15.29 Å, γ = 85.24°) to the square 2DPI lattice (a = b = 25.1 Å, γ = 90°). This lattice expansion changes the epitaxial relationship to line-on-line epitaxy (defined as coincidence of nonprimitive reciprocal lattice vectors of the adsorbate and the substrate), where the 2DPI [6,3] lattice lines coincide with the SOS [1,1] crystallographic lines.

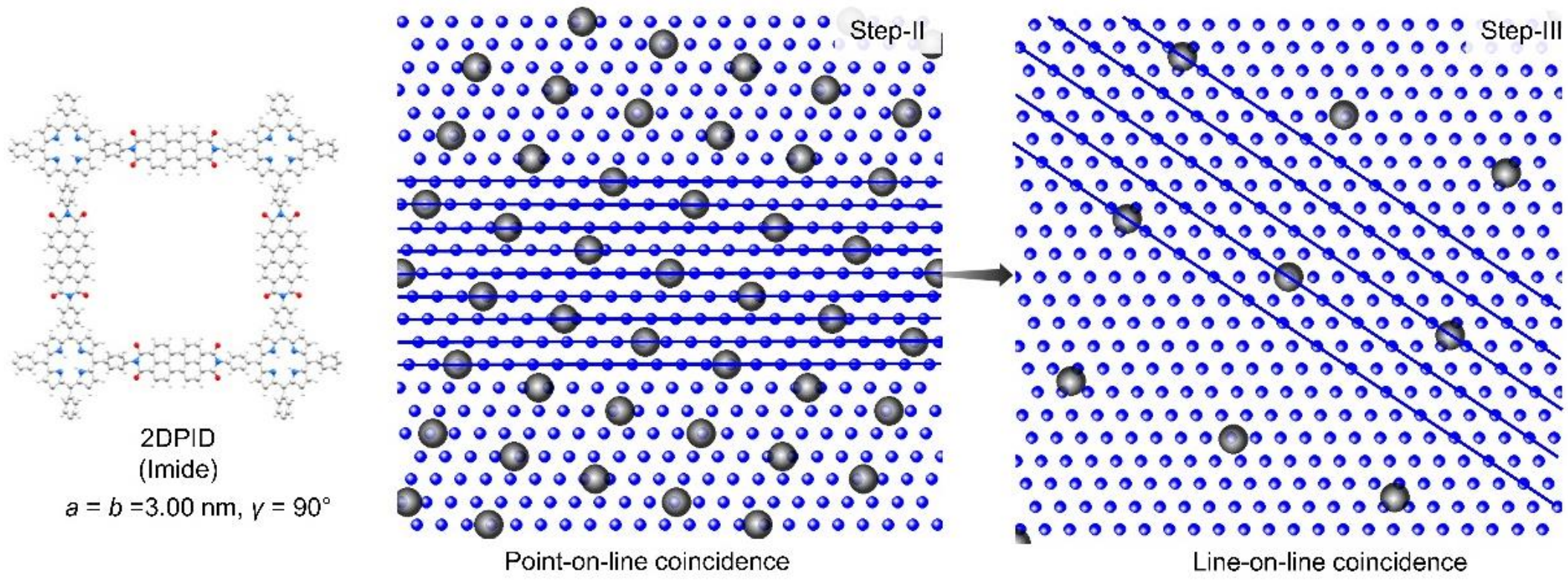

Supplementary Fig. 26 | Epitaxial transition in on-water surface synthesis of 2DPID. Schematic illustration of point-on-line coincidence during M3 pre-assembly in Step-II and line-on-line coincidence during 2DPBI formation in Step-III.

Case-II: 2DPID formation with anionic SOS surfactant.

For 2DPID (a = b = 3.00 nm, γ = 90°), the Step-II epitaxial alignment also corresponds to point-on-line epitaxy, in which M1 lattice points register along the (1,0) lattice lines of the SOS surfactant lattice. After the addition of M7 (perylene-3,4,9,10-tetracarboxylic dianhydride) in Step-III, the lattice expands from the pre-assembled M1 structure (a = 13.22 Å, b = 15.29 Å, γ = 85.24°) to the square 2DPID lattice (a = b ≈ 30.0 Å, γ = 90°). The epitaxial relationship then evolves into a line-on-line epitaxy, where extended 2DPID [-7,2] lattice lines coincide with the SOS [1,0] crystallographic lines.

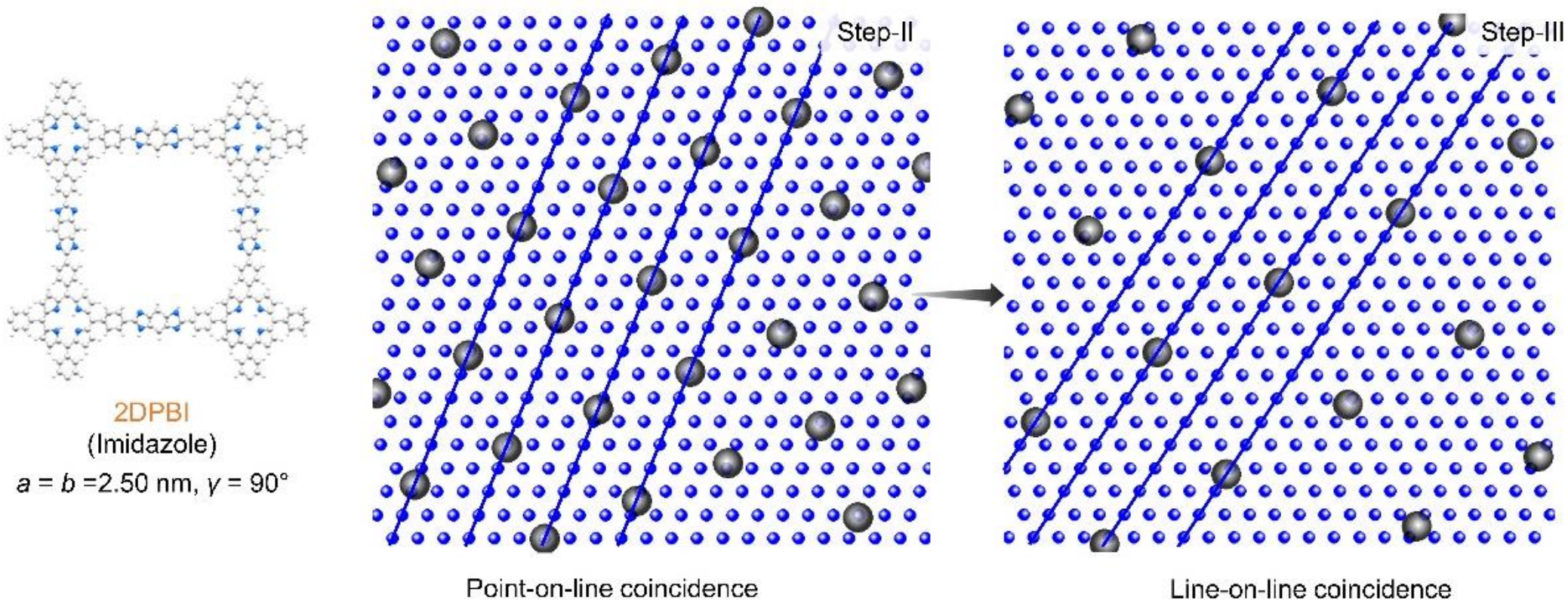

Supplementary Fig. 27 | Epitaxial transition in on-water surface synthesis of 2DPBI Schematic illustration of point-on-line coincidence during M1 pre-assembly in Step-II and line-on-line coincidence during 2DPID formation in Step-III.

Case-III: 2DPBI formation with cationic CTAB surfactant.

For 2DPBI (a = b = 2.50 nm, γ = 90°), the Step-II epitaxial alignment corresponds to point-on-line epitaxy, in which M3 lattice points register along the (0,1) lattice lines of the CTAB surfactant lattice. After M4 addition in Step-III, the lattice expands from the pre-assembled M3 structure (a = 17.53 Å, b = 17.49 Å, γ = 89.40°) to the square 2DPBI lattice (a = b = 25.1 Å, γ = 90.00°). The epitaxial relationship then evolves into line-on-line epitaxy, where extended 2DPBI [3,–6] lattice lines coincide with the CTAB [1,1] crystallographic lines.

Epitaxial growth is fundamentally driven by the minimization of total system energy, i.e. it depends on the balance between interfacial energy and the elastic strain required to match the interacting lattices. In our system, we would characterize the driving force for the transition from point-on-line to line-on-line epitaxy to be the dramatic lattice expansion that occurs during covalent polymerization (Step-III). During Step-II, the discrete monomer assemblies maintain a point-on-line registry with the charged crystalline surfactant. However, as the second monomer is added and covalent bonds form, the resulting 2D polymer lattice expands, and the much larger lattice will establish an epitaxial relationship that again minimizes energy (or none if none is available) – in our cases adopting line-on-line epitaxy in order to minimize the overall energy for the newly expanded lattice.

Since line-on-line epitaxy is the most general (every point-on-line lattice is thus also line-on-line), it therefore also represents the lowest degree of epitaxial matching, and it is possibly more common in systems where the elastic energy to distort the involved unit cells to higher degrees of epitaxy would exceed any potential gain in interface energy. However, because the epitaxial match in our systems involves soft crystals growing on soft templates, establishing a strict, quantitative energetic hierarchy

would require comprehensive energy calculations; however, the qualitative driving force is clearly the strain-induced relaxation of the expanded polymer.

Regarding universality, we would state that this mechanism cannot be considered a general rule for all monomers or lattice systems. The preferred epitaxial state depends sensitively on the exact balance of intra- and interlayer interactions—specifically, the elastic moduli of the two lattices and their interlayer interaction potentials. If, for instance, a different 2D polymer had a chemical structure and dimensions that by chance allowed a higher degree of lattice match (e.g., commensurate epitaxy) with minimal distortion, yielding a high gain in interfacial energy, one would likely observe commensurate epitaxy. While we consistently observe the transition to line-on-line epitaxy for all square-lattice 2D polymers examined in this study, the exact epitaxial evolution in other systems would thus remain highly dependent on monomer geometry, charge complementarity, surfactant lattice parameters, and the final 2D polymer structure.

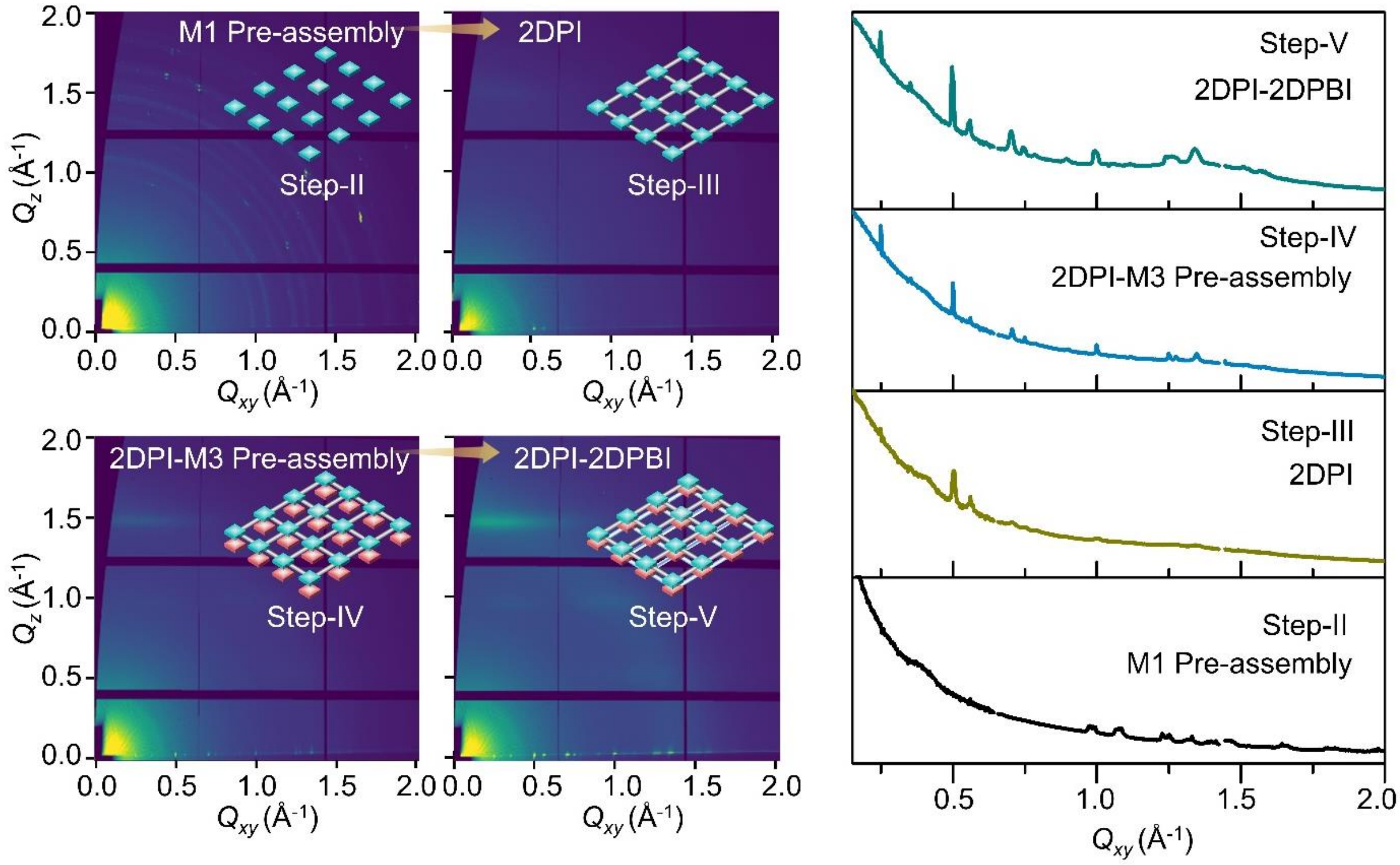

Supplementary Fig. 28 | Stepwise epitaxial assembly of a lattice-matched 2DPI–2DPBI vdW heterostructure. Ex-situ 2D GIWAXS patterns (films transferred on silicon) acquired after each step: Step-II, M1 pre-assembly; Step-III, 2DPI after M2-triggered 2D polymerization (18 h after M2 addition); Step-IV, pre-organization of M3 beneath 2DPI (6 h after M3 addition); Step-V, formation of the 2DPI–2DPBI heterostructure after M4-triggered polymerization. The comparison of the Bragg peak positions extracted from the images shows that the O_{xy} position of the reflections remains fixed while diffraction intensity increases from Step-III to Step-V, indicating that growth proceeds epitaxially on the pre-established line-on-line registry and preserves a commensurate, lattice-matched alignment of the two lattices along the (100) and (010) directions.

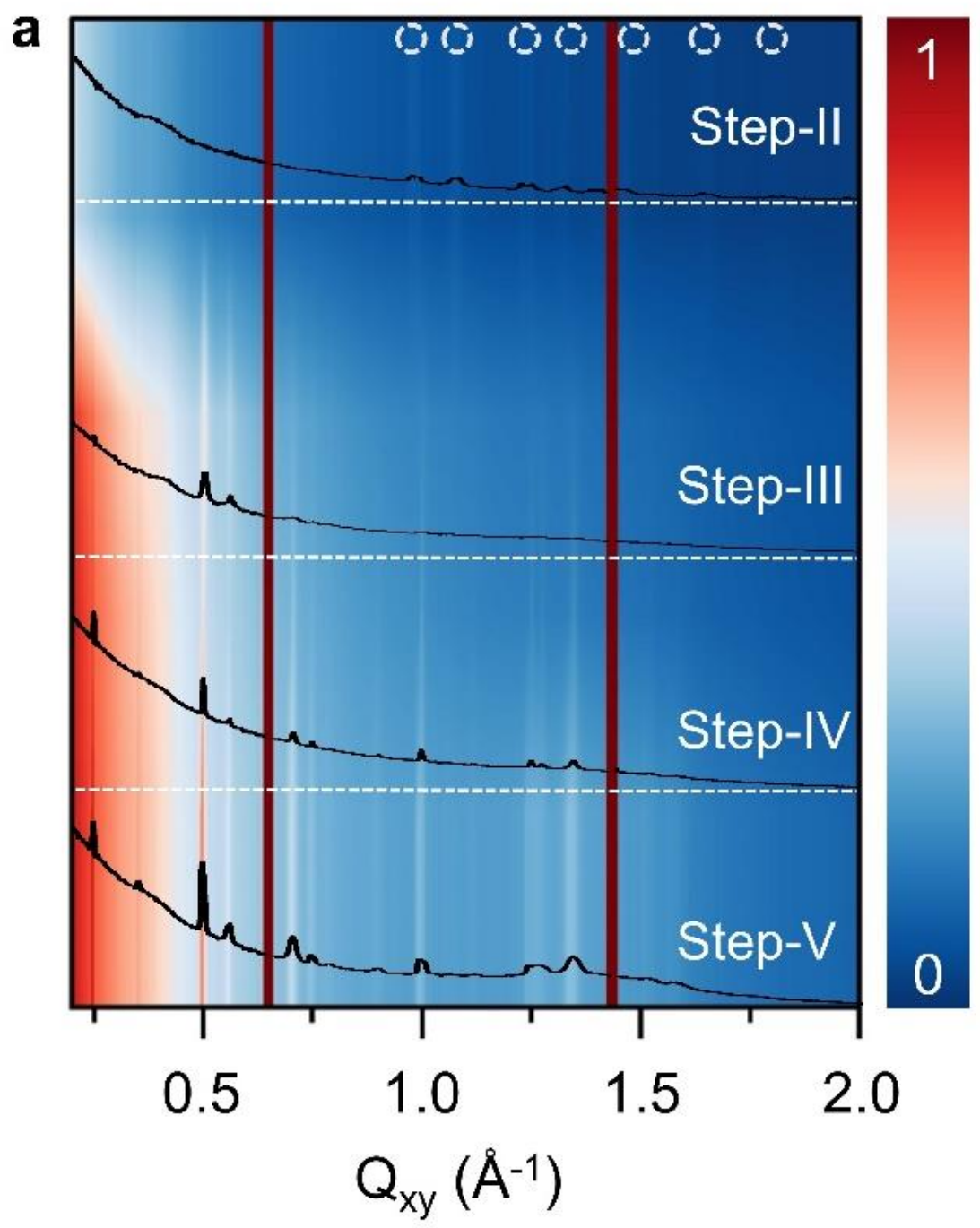

b

(hk)	Meas. peak (Å^{-1})	Fitted peak (Å^{-1})
(0, 1)	0.248	0.249
(1,-1)	0.352	0.352
(0, 2)	0.498	0.498
(1,-2)	0.557	0.557
(2,-2)	0.703	0.704
(0, 3)	0.746	0.747
(1,-3)	0.786	0.788
(2,-3)	0.896	0.898
(0, 4)	0.996	0.996
(3,-3)	1.058	1.057
(2,-4)	1.116	1.114
(0, 5)	1.256	1.246
(2,-5)	1.342	1.342
(1,-6)	1.515	1.515
(2,-6)	1.578	1.576

Supplementary Fig. 29 | Layer-by-layer growth analysis by ex-situ GIWAXS. Improved-contrast ex-situ GIWAXS patterns and corresponding 1D line-cut profiles from Step-III to Step-V, showing Bragg reflections at consistent Qxy positions with increasing intensity during sequential assembly.

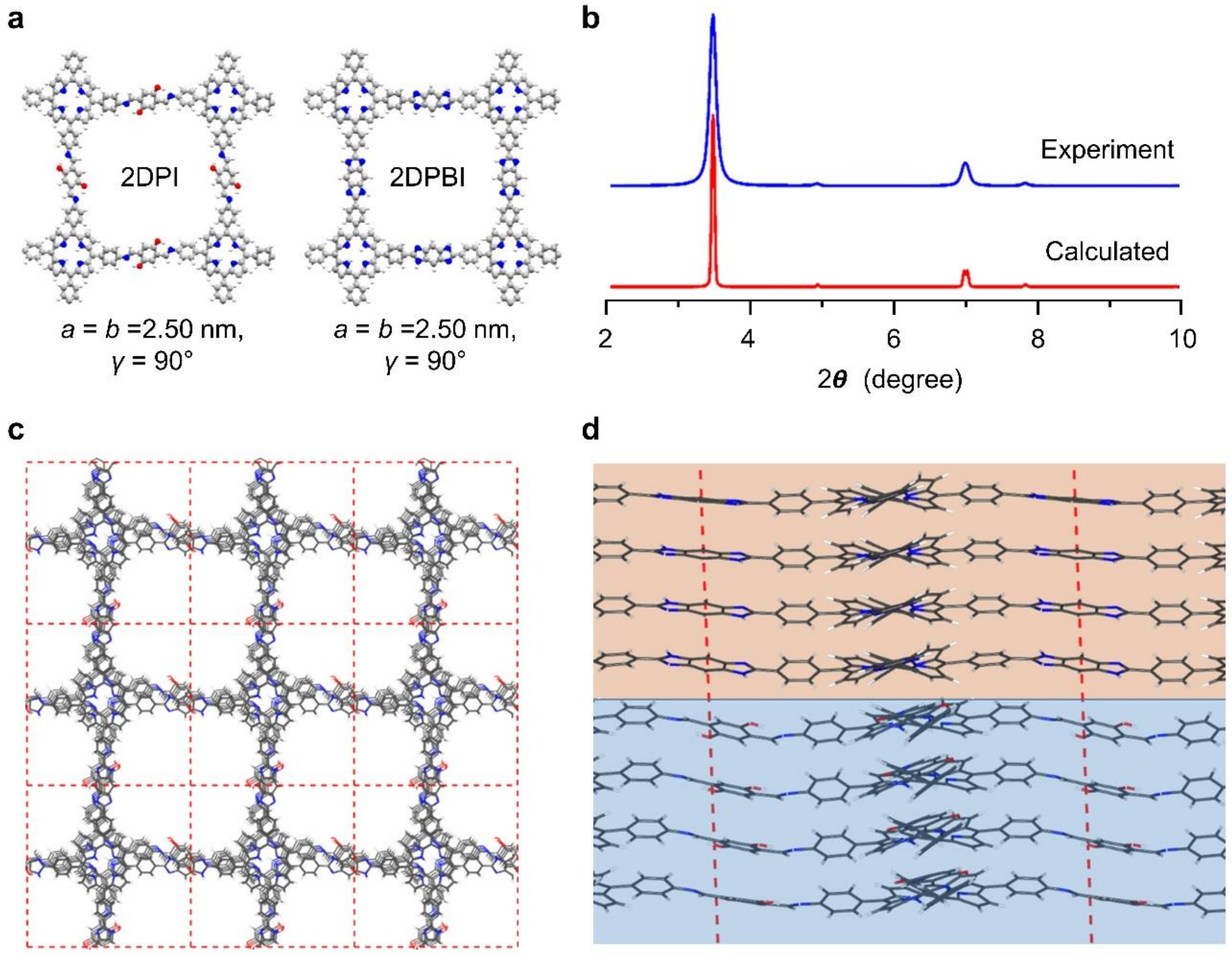

Supplementary Fig. 30 | Structural model and X-ray diffraction validation of the 2DPI–2DPBI vdW heterostructure. a, Parent 2D polymer lattices of 2D polyimine (2DPI) and 2D polybenzimidazole (2DPBI) (lattice parameter ≈ 2.5 nm). b, Experimental diffraction profile (blue) obtained from the in-plane GIWAXS signal of the heterostructure and converted from Q_{xy} to 2θ for direct comparison with the calculated pattern (red) simulated from the structural model. c,d, VESTA-rendered model of the heterostructure in top view (c) and side view (d), showing stacked 2DPI (blue) and 2DPBI (orange) layers.

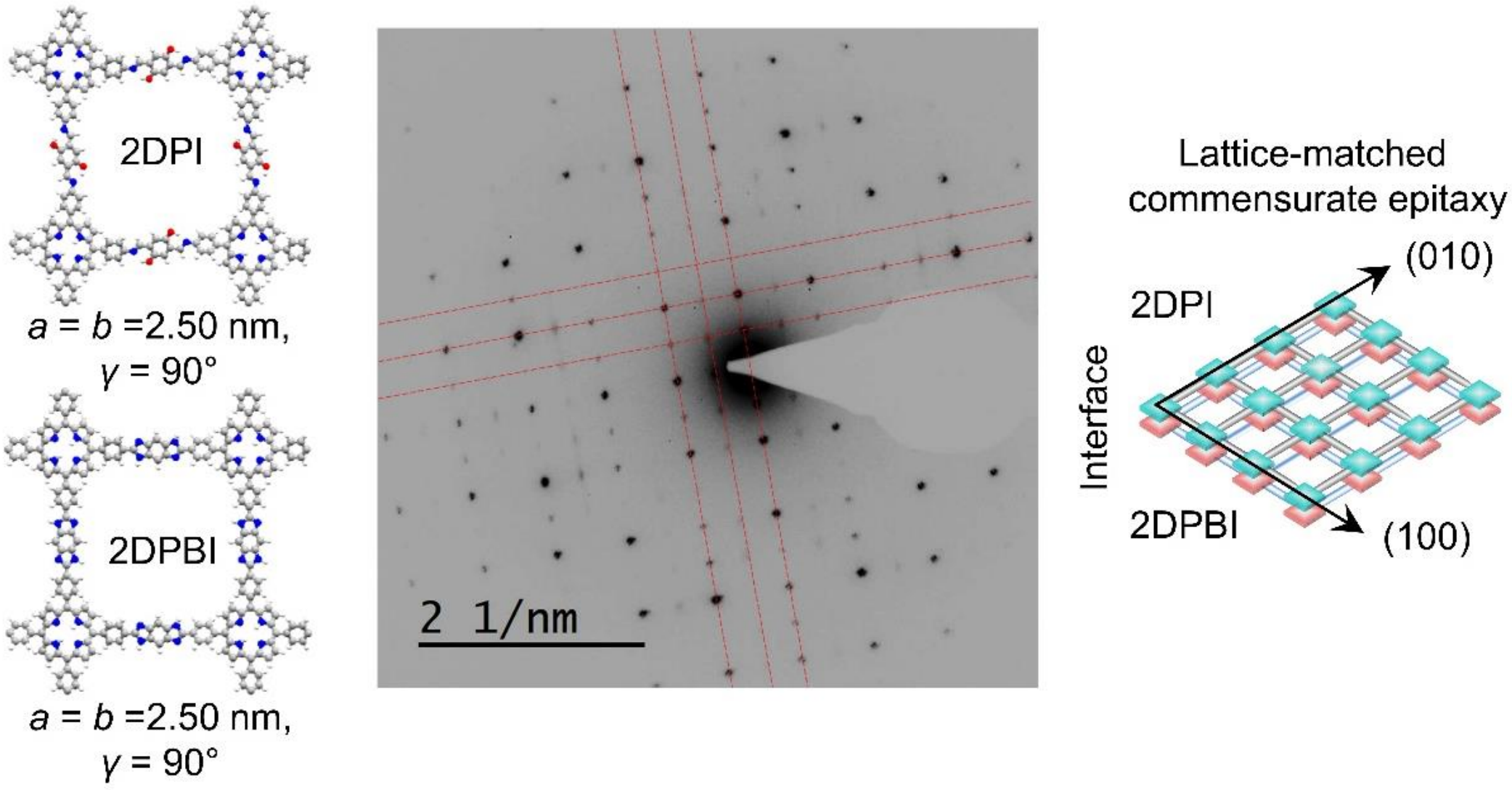

Supplementary Fig. 31 | Electron microscopy of the 2DPI–2DPBI heterostructure. Structural motifs of the constituent 2D polymers 2DPI (top) and 2DPBI (bottom). Selected-area electron diffraction (SAED) pattern from the 2DPI–2DPBI heterostructure showing a square reciprocal lattice; first-order reflections at 0.4 nm^{-1} correspond to a real-space lattice parameter of 2.5 nm, consistent with the unit cell derived from GIWAXS. Schematic of the lattice-matched, commensurate stacking of 2DPI and 2DPBI in the vdW heterostructure.

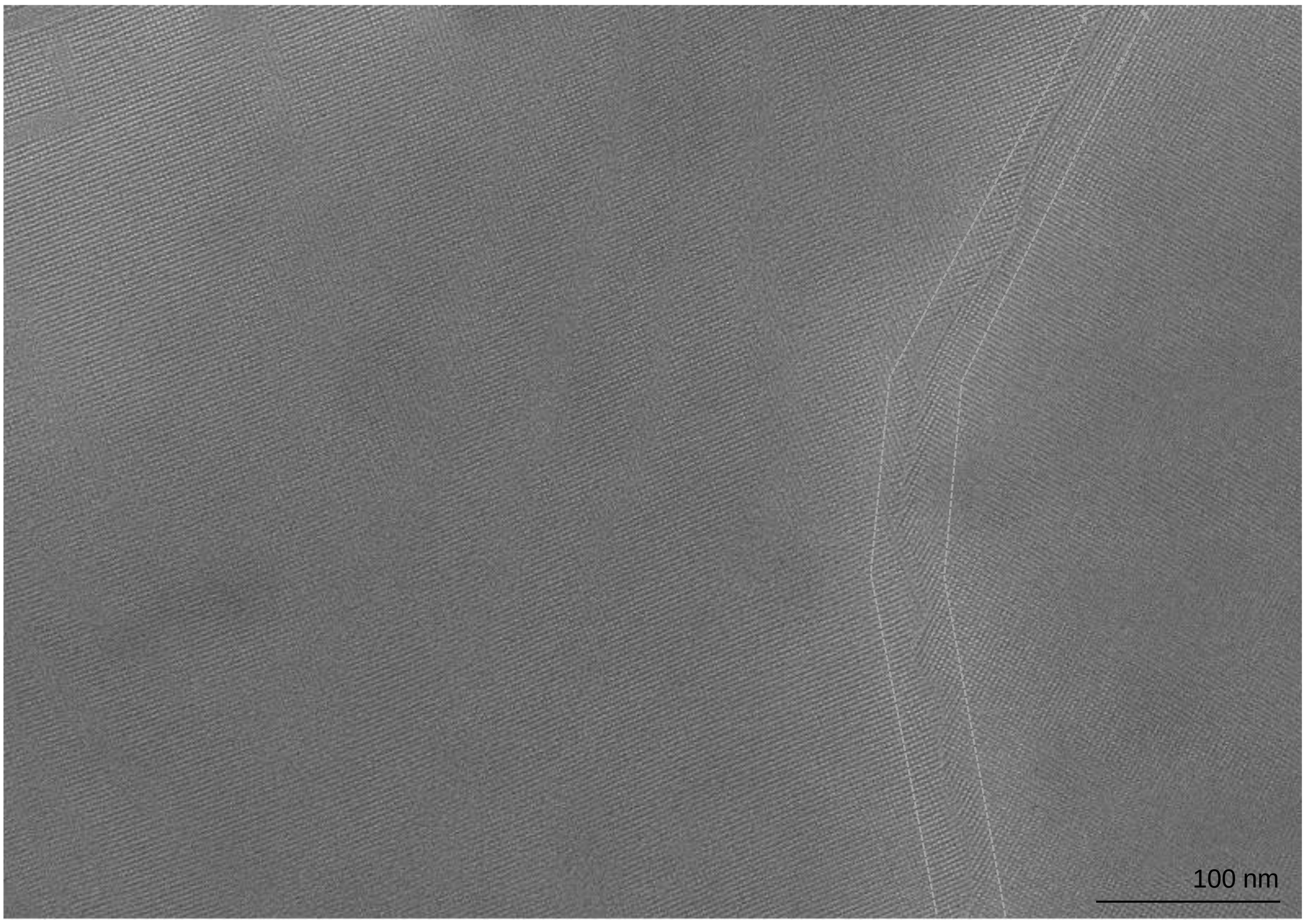

Supplementary Fig. 32 | Large-area HRTEM of the 2DPI–2DPBI vdW heterostructure. Large-area high-resolution TEM image showing uniform lattice contrast across the film. The absence of Moiré fringes supports a well-aligned, lattice-matched epitaxial relationship between the 2DPI and 2DPBI layers. Scale bar, 100 nm.

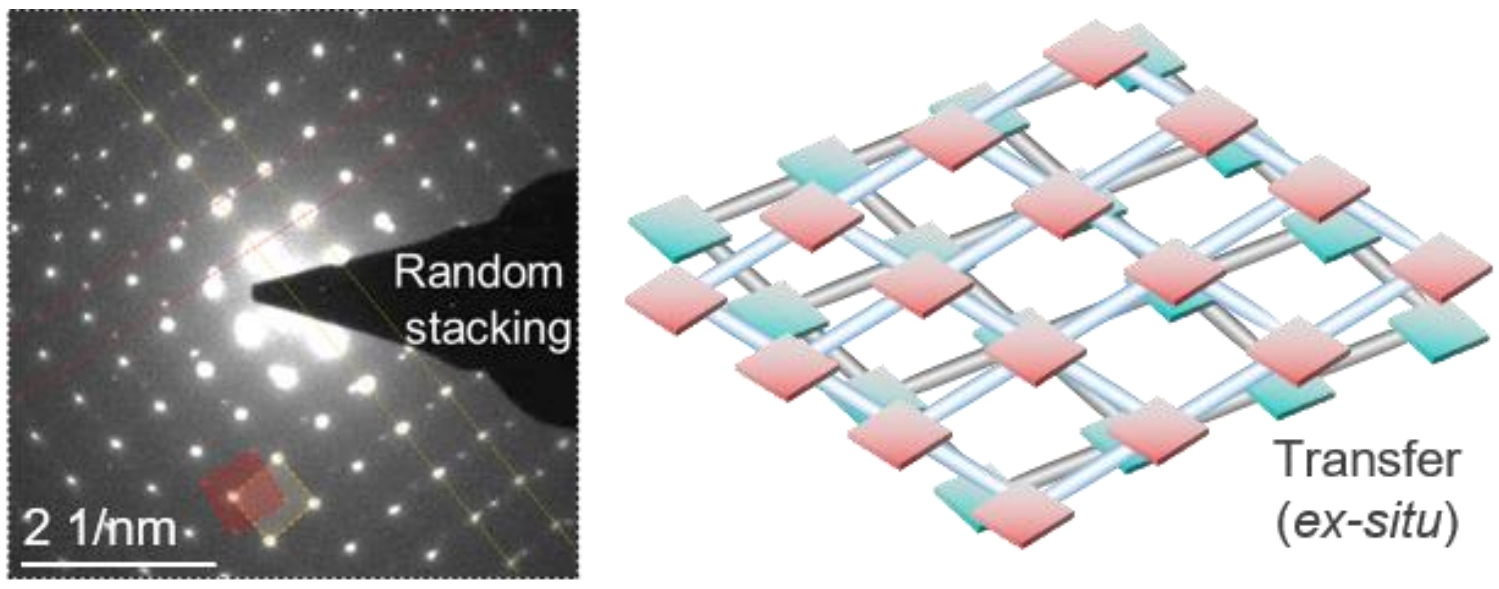

Supplementary Fig. 33 | Random rotational registry in mechanically assembled 2DPI–2DPBI vdW heterostructures. Selected-area electron diffraction (SAED) from an ex-situ mechanically assembled 2DPI–2DPBI vdW heterostructure shows multiple sets of discrete reflections at the same scattering radius but different azimuthal angles, indicating lattice-matched layers (common reciprocal-lattice magnitude, $|g| \approx 0.4\ nm^{-1}$) with random in-plane rotational registry. A schematic illustrates the ex-situ transfer/stacking configuration.

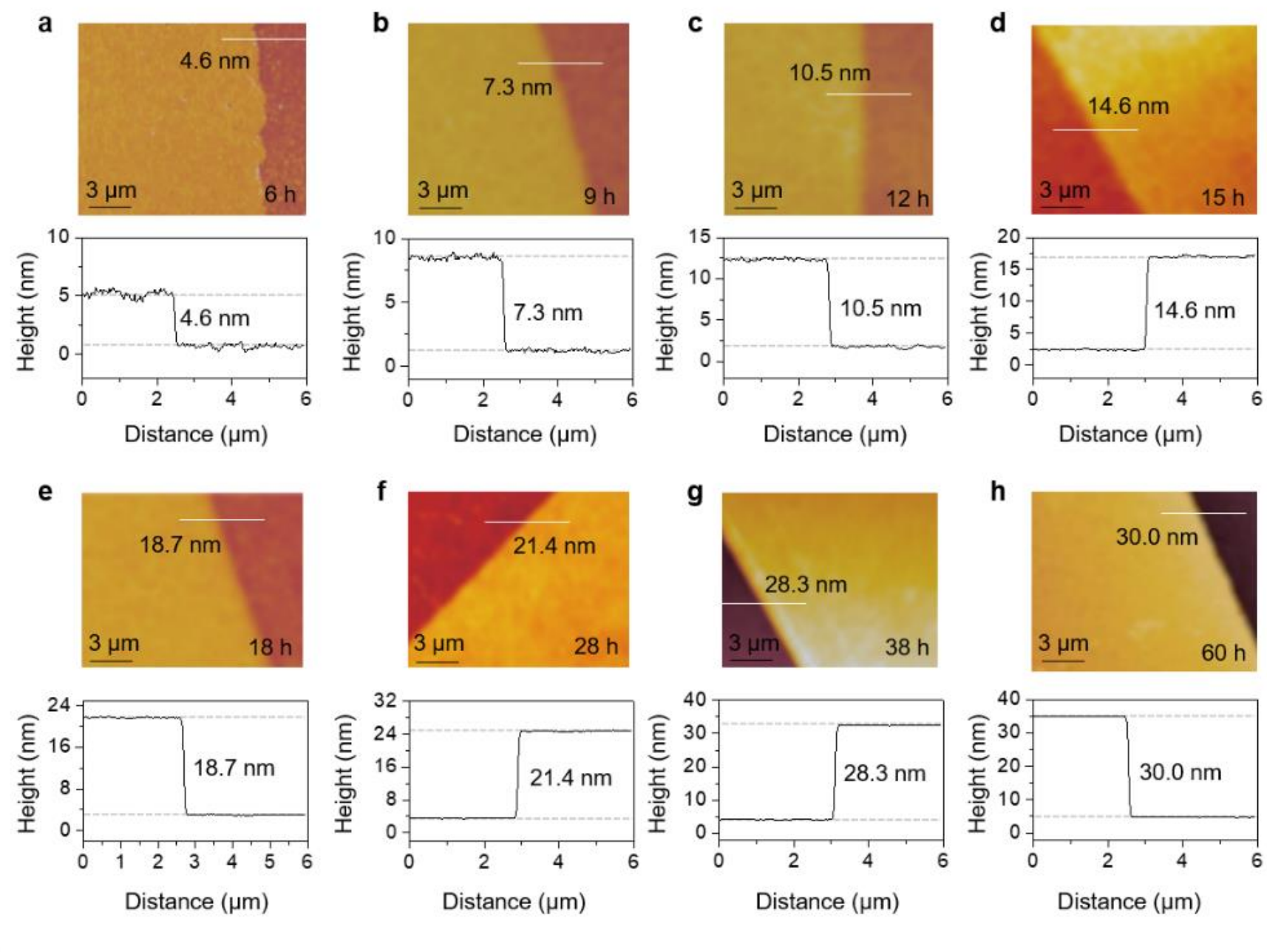

Supplementary Fig. 34 | Thickness control of 2D polymer vdW heterostructures. AFM images and corresponding height profiles showing the reaction-time-dependent thickness increase of the 2D polymer vdW heterostructures.

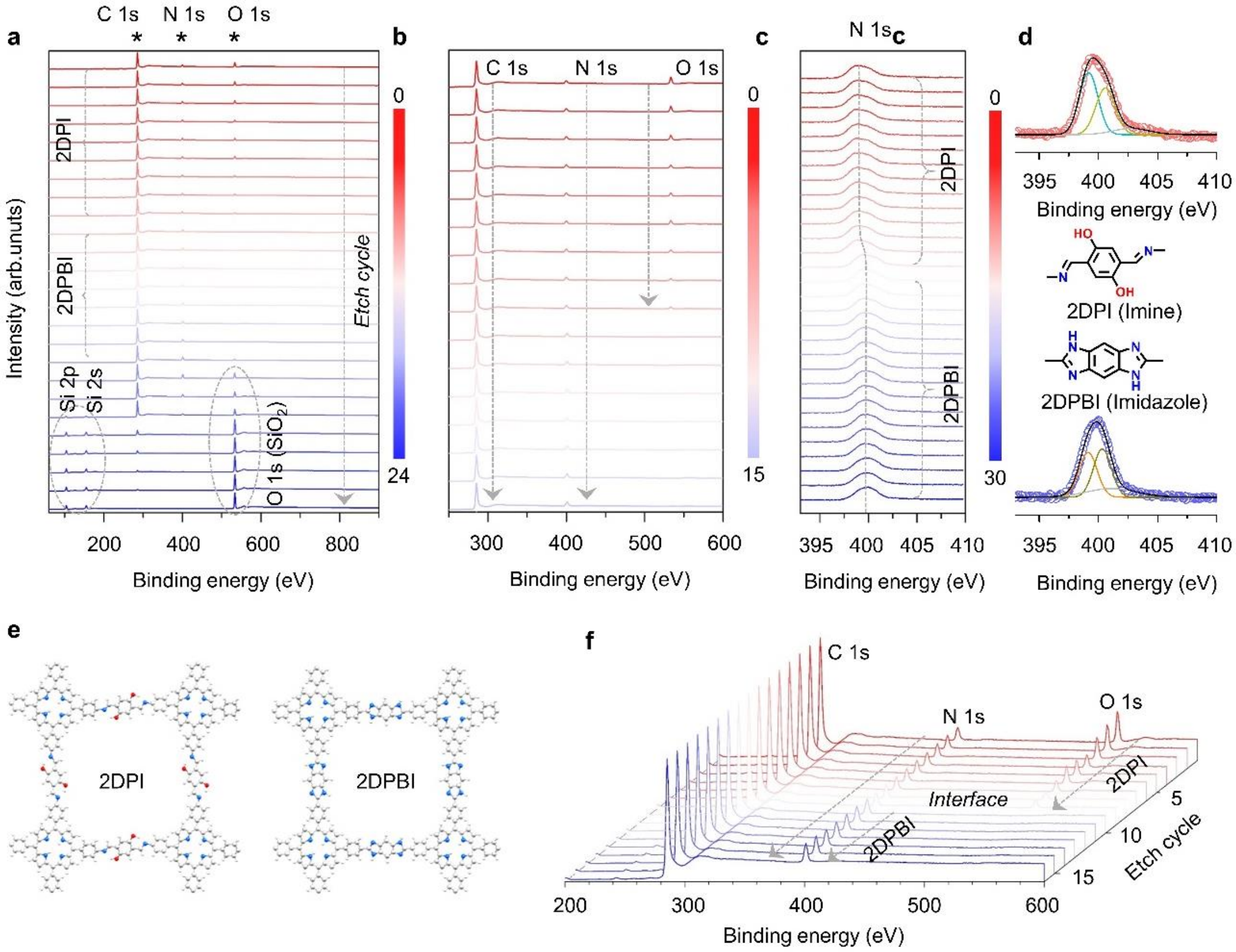

Supplementary Fig. 35 | XPS depth-profile analysis of the 2DPI–2DPBI vdW heterostructure. (a) Full XPS survey spectra collected along the etching depth. (b) Enlarged survey spectra showing the evolution of C 1s, N 1s, and O 1s signals. (c) High-resolution N 1s spectra along the depth profile. (d) Deconvoluted N 1s spectra from the top 2DPI region and bottom 2DPBI region. (e) Chemical structures of 2DPI and 2DPBI. (f) 3D representation of the survey spectra in (b), highlighting the transition across the 2DPI–2DPBI interface.

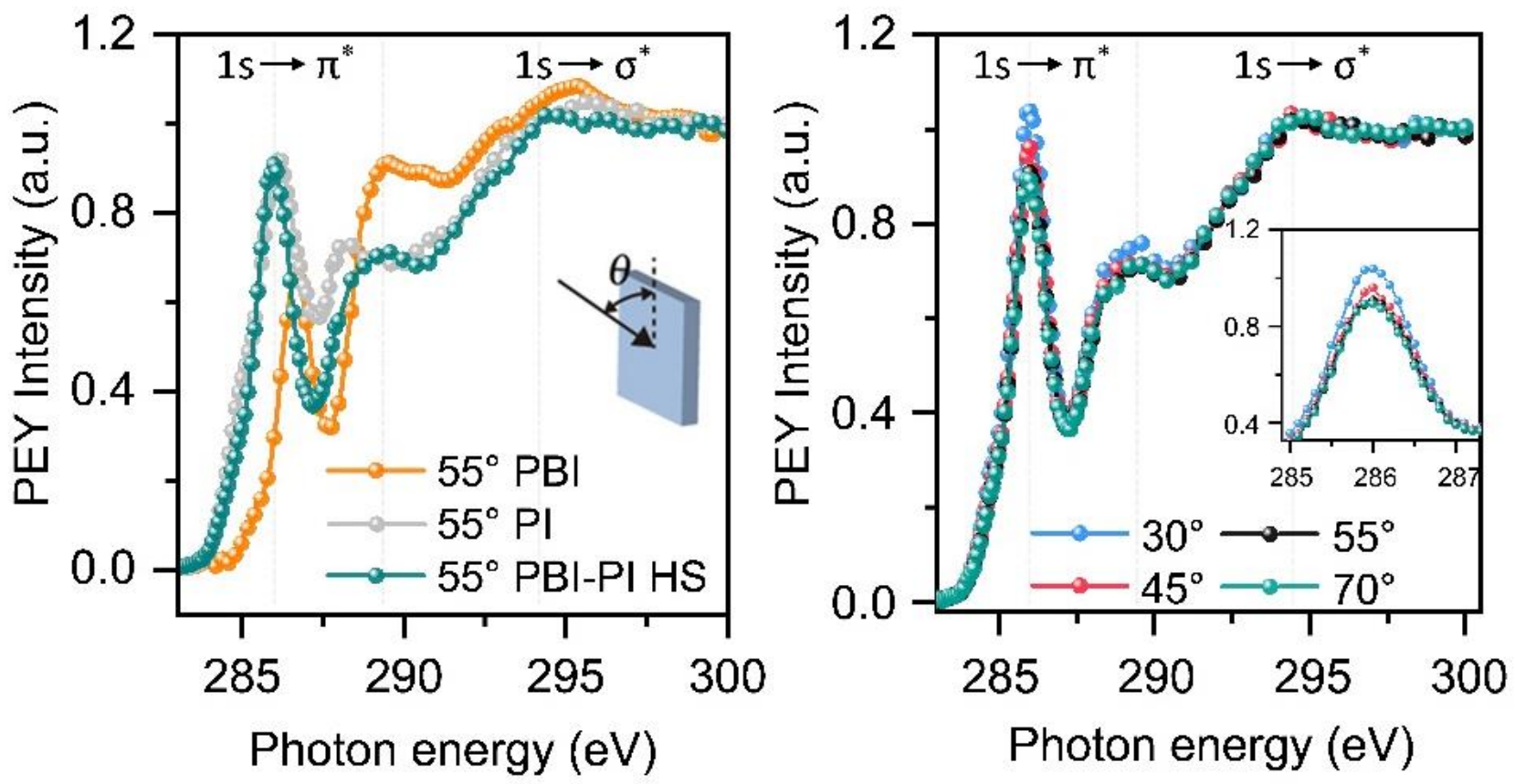

Supplementary Fig. 36 | Angle-dependent NEXAFS of the lattice-matched 2DPI–2DPBI vdW heterostructure. C K-edge NEXAFS spectra collected in partial electron yield (PEY) mode. Spectra of 2DPI, 2DPBI, and the 2DPI–2DPBI heterostructure measured at an incidence angle of 55°, showing the characteristic 1s→π* transition at ~286 eV and 1s→σ* transition at ~290 eV. Incidence-angle-dependent PEY spectra of the heterostructure (θ = 30°, 45°, 55°, and 70°; inset highlights the π* region), where the pronounced angular dependence indicates an ordered, predominantly in-plane orientation of the stacked layers.

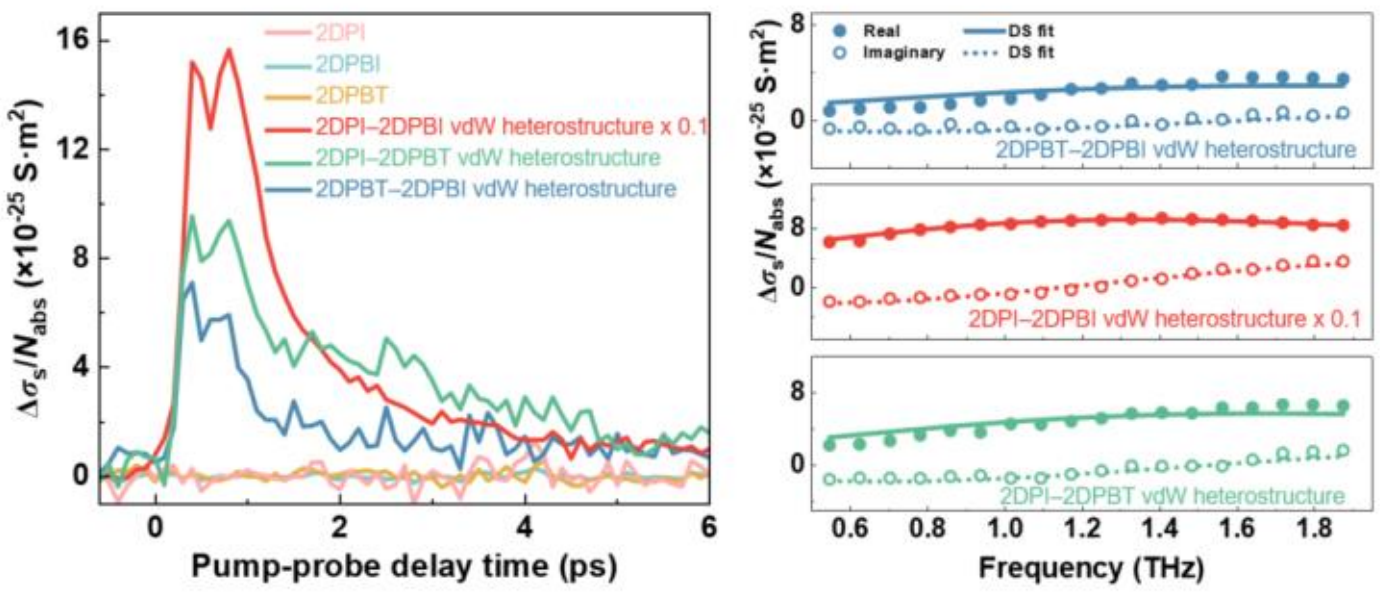

Supplementary Fig. 37 | Time- and frequency-resolved THz photoconductivity of lattice-matched 2D polymer vdW heterostructures. Time-resolved THz photoconductivity for the parent 2D polymers (2DPI, 2DPBI, 2DPBT) and the corresponding lattice-matched vdW heterostructures (2DPI–2DPBI, 2DPI–2DPBT, and 2DPBT–2DPBI), showing strongly enhanced photoresponse for the heterostructures. Complex, frequency-resolved THz photoconductivity (real and imaginary components) at ~1 ps after optical excitation, together with Drude–Smith (DS) model fits.

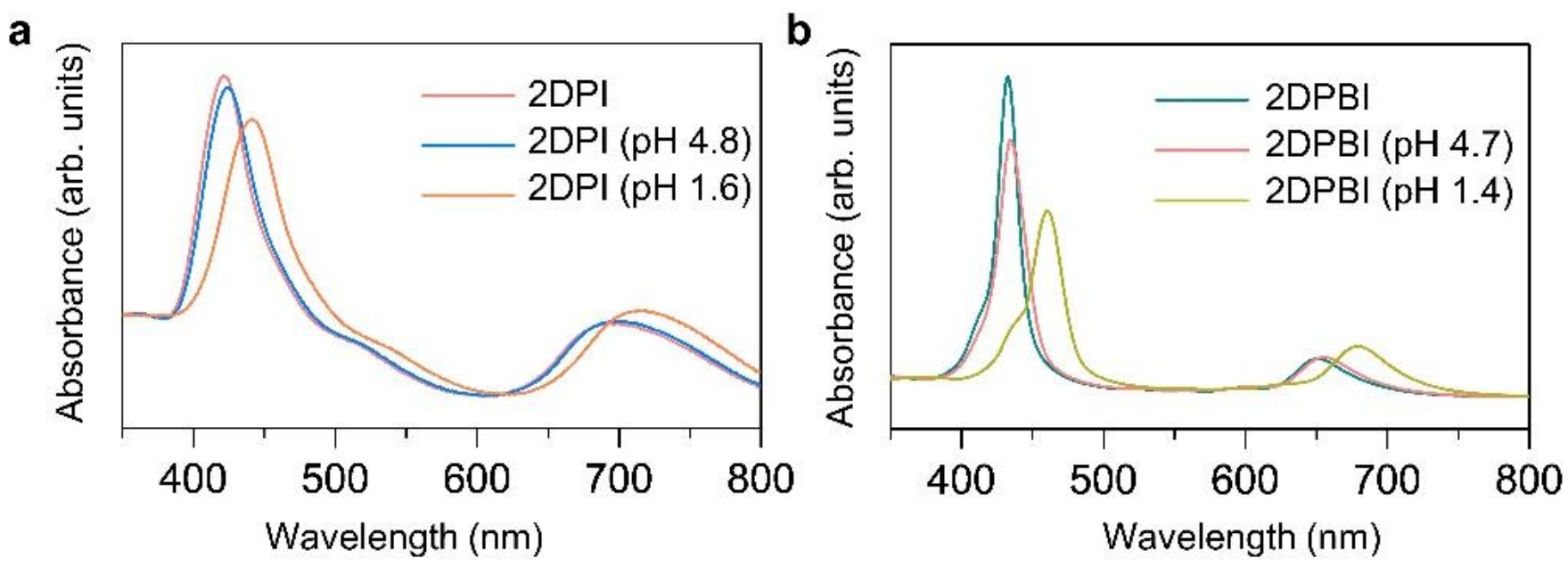

Supplementary Fig. 38 | pH-dependent UV–vis spectra of 2DPI and 2DPBI films. UV–vis absorption spectra of (a) 2DPI and (b) 2DPBI films before and after acid treatment, showing protonation-induced red shifts of the Soret and Q bands under acidic conditions.

To further check possible acid trapping/protonation, we performed pH-dependent UV–vis control experiments. Upon acid treatment of the as-synthesized films, protonation of the porphyrin core gives clear red shifts of the Soret and Q bands. For 2DPI, the Soret band shifts from 421.6 nm in the as-synthesized film to 423.8 nm at pH 4.8 and 442.15 nm at pH 1.6. For 2DPBI, the Soret band shifts from 431.5 nm in the as-synthesized film to 435 nm at pH 4.7 and 460 nm at pH 1.4. Similar red shifts are observed in the Q-band region. These changes provide clear spectroscopic signatures of porphyrin protonation. In contrast, the as-synthesized 2DPI and 2DPBI films do not show these protonation-induced red-shifted features. This is consistent with our synthesis protocol, in which excess monomers and acidic species are removed at each step and the subphase is exchanged with fresh Milli-Q water. These results support neutral free-base porphyrin cores in both 2DPI and 2DPBI and exclude significant acid trapping/protonation.

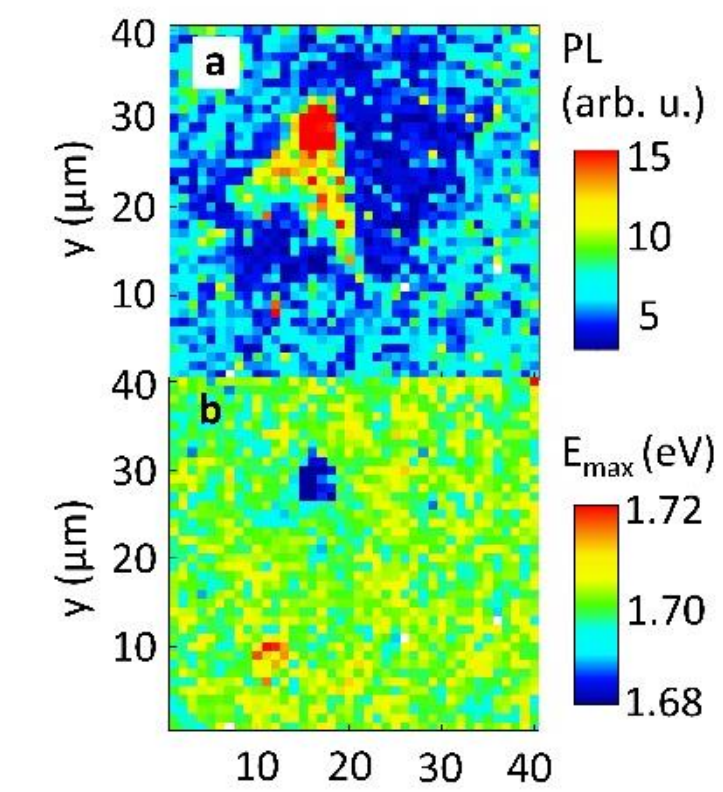

Supplementary Fig. 39 | Spatial photoluminescence (PL) mapping of the 2DPI–2DPBI vdW heterostructure. (a) PL intensity map and (b) PL peak-energy map acquired over a 40 × 40 μm^2 area with a 1 μm step size. The heterostructure exhibits largely homogeneous emission across the mapped region, with a consistent PL maximum of E_{max}=1.700 ± 0.004 eV.

Structural and electronic properties of parent 2D Polymer.

This section details the structural and electronic characterization of the parent 2DPs. It includes top-view representations of the 2D polymer structures, X-ray diffraction patterns confirming structural integrity, and electronic properties illustrated through band structure and density of states (DOS) diagrams. Planar-average potential graphs are also presented, with the potential adjusted relative to the vacuum level set to zero for uniform comparison across all structures.

2D Polybenzimidazole (2DPBI)

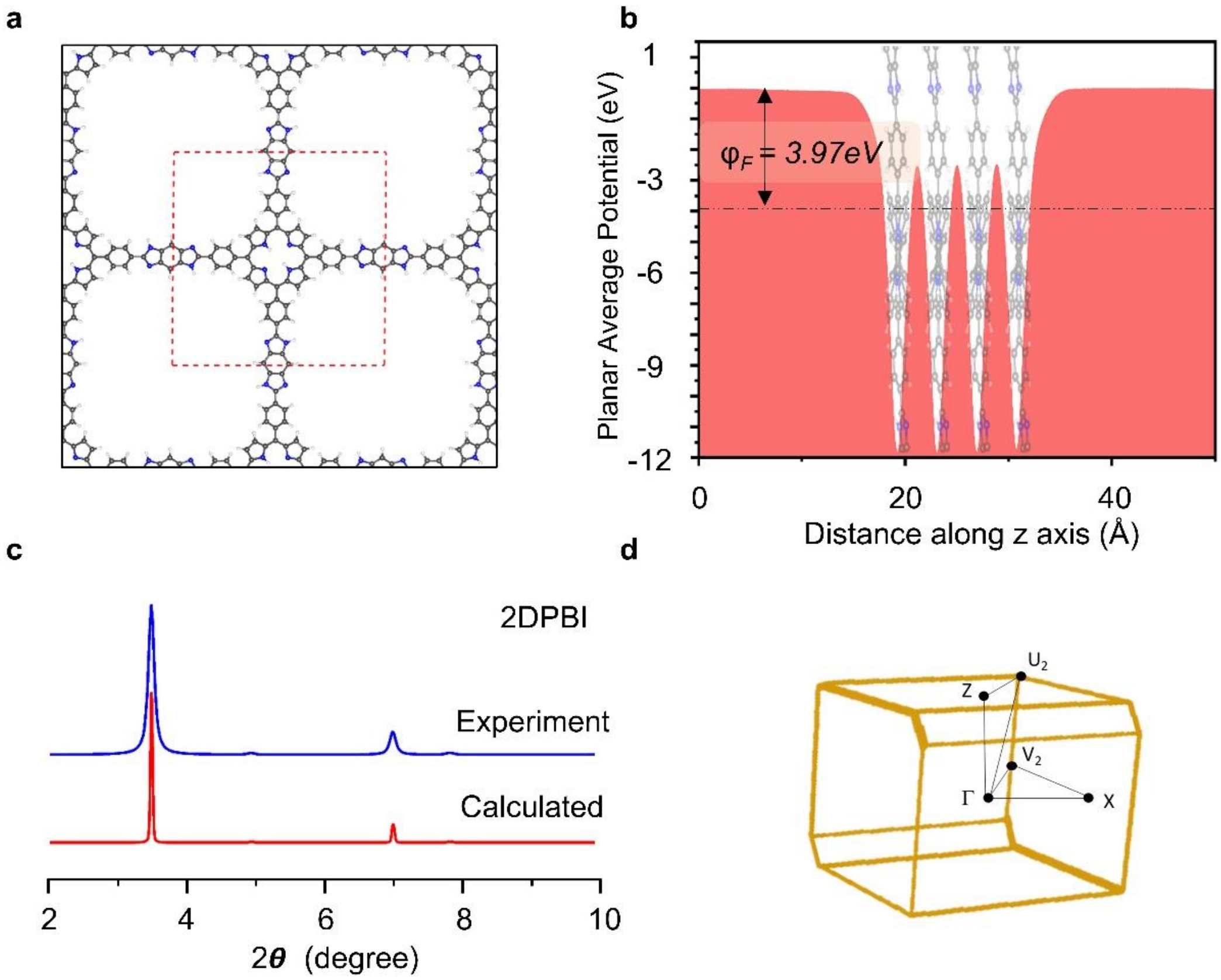

Supplementary Fig. 40 | 2D Polybenzimidazole (2DPBI): (a) Top view of the four-layer 2DPBI slab. (b) One-dimensional planar-averaged electrostatic potential along the z-direction. (c) Experimental diffraction profile (blue) obtained from the in-plane GIWAXS signal of the 2DPBI and converted from Q_{xy} to 2θ for direct comparison with the calculated pattern (red) simulated from the structural model. (d) Representation of the Brillouin zone.

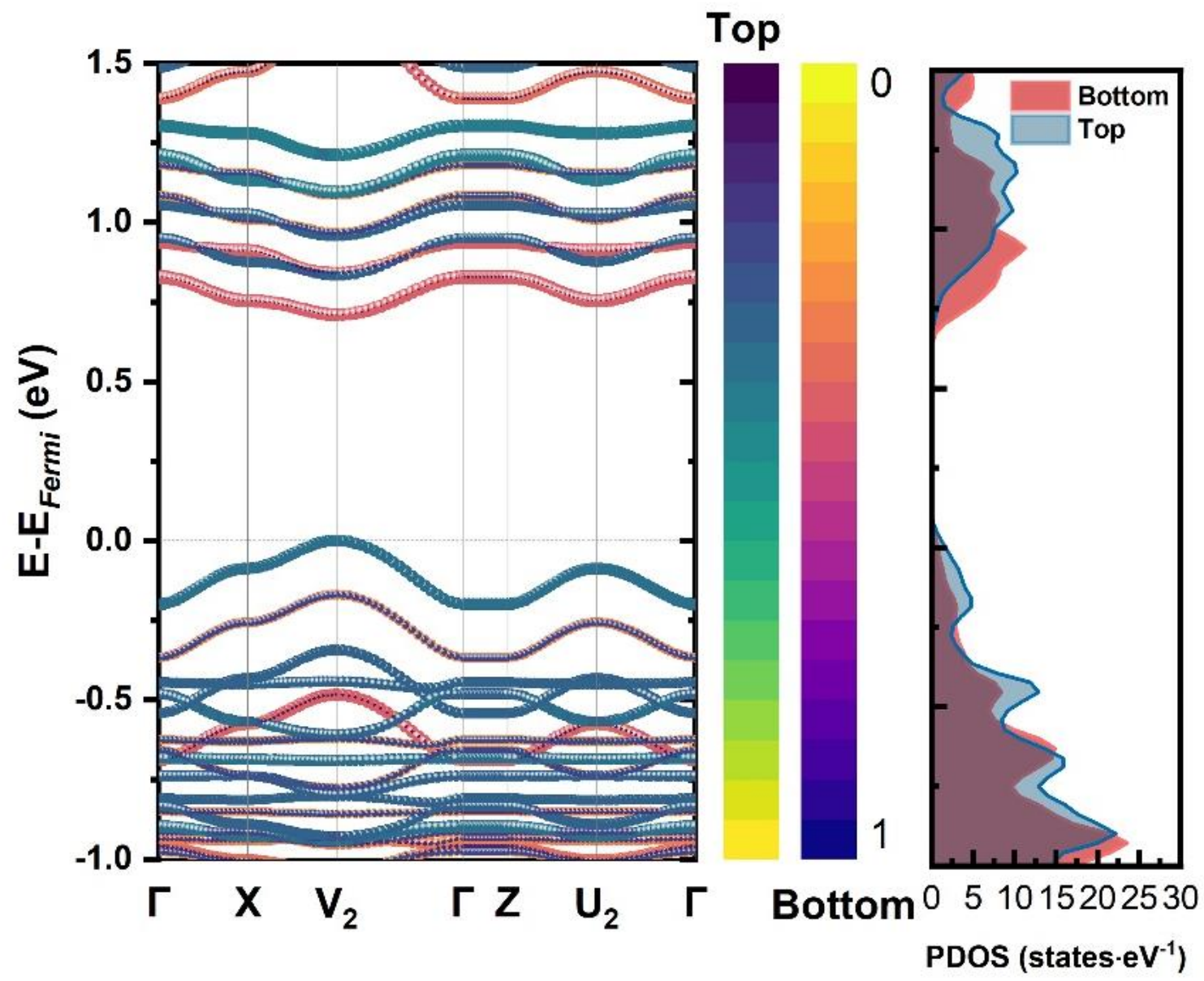

Supplementary Fig. 41 | 2D Polybenzimidazole (2DPBI). Band structure of the 2DPBI slab, separated into contributions from the top two and bottom two layers. (Right) Partial density of states (PDOS) of the 2DPBI slab**.**

2D Polybenzothiazole (2DPBT)

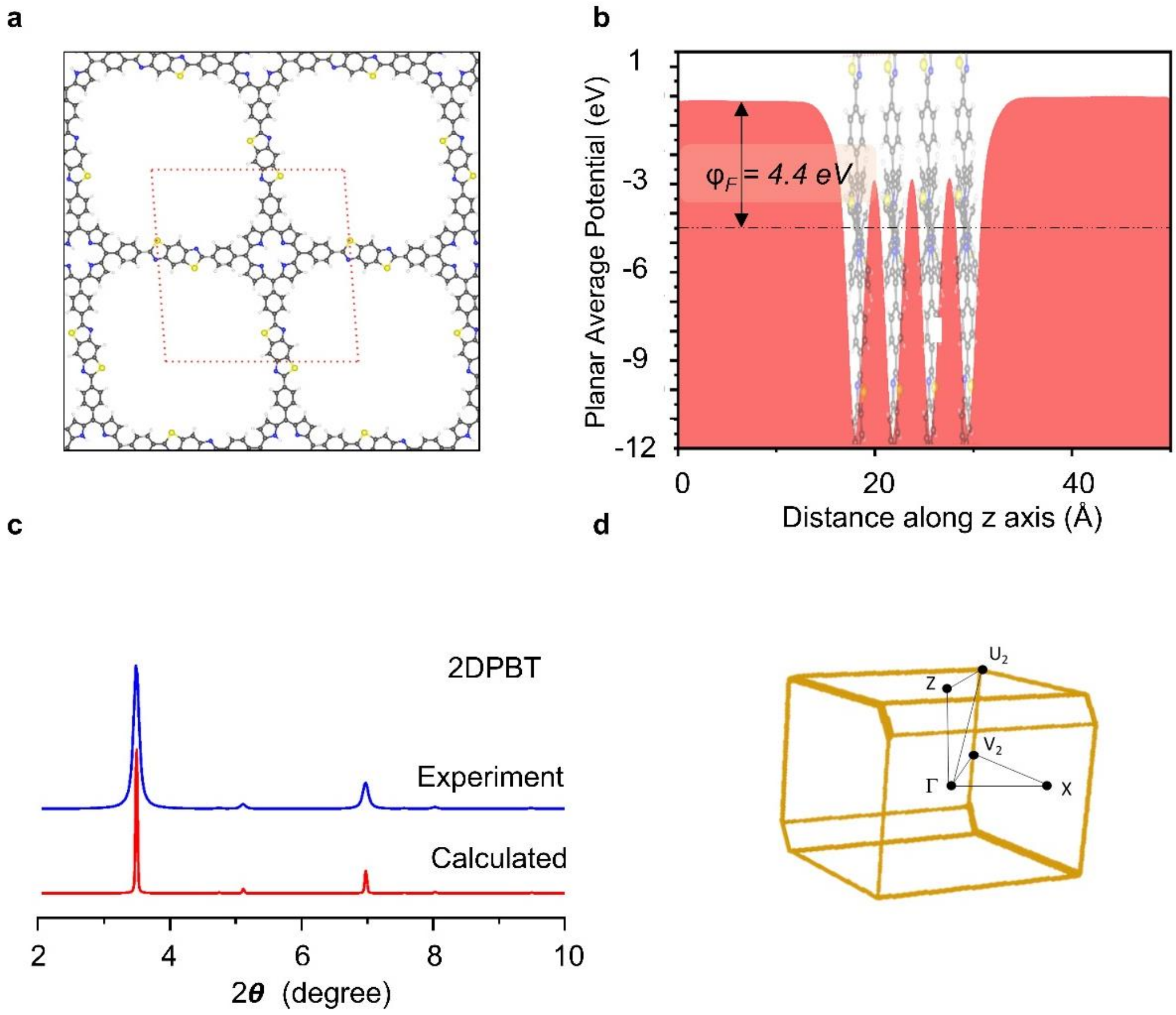

Supplementary Fig. 42 | 2D Polybenzothiazole (2DPBT) (a) Top view of the four-layer 2DPBT slab. (b) One-dimensional planar-averaged electrostatic potential along the z-direction (c) Experimental diffraction profile (blue) obtained from the in-plane GIWAXS signal of the 2DPBT and converted from Q_{xy} to 2θ for direct comparison with the calculated pattern (red) simulated from the structural model. (d) Representation of the Brillouin zone.

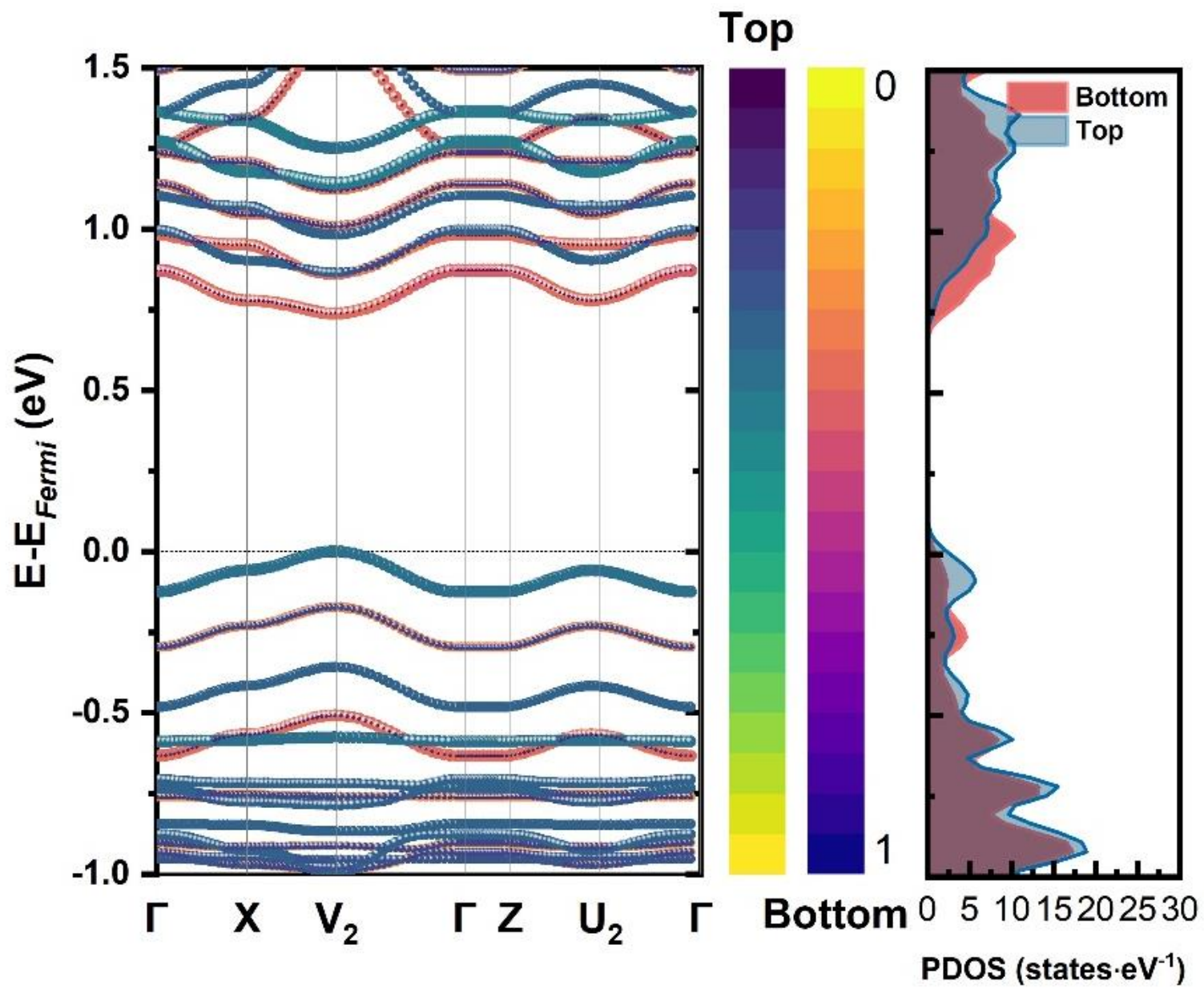

Supplementary Fig. 43 | 2D Polybenzothiazole (2DPBT). Band structure of the 2DPBT slab, separated into contributions from the top two and bottom two layers. (Right) Partial density of states (PDOS) of the 2DPBT slab**.**

2D Polyimine (2DPI)

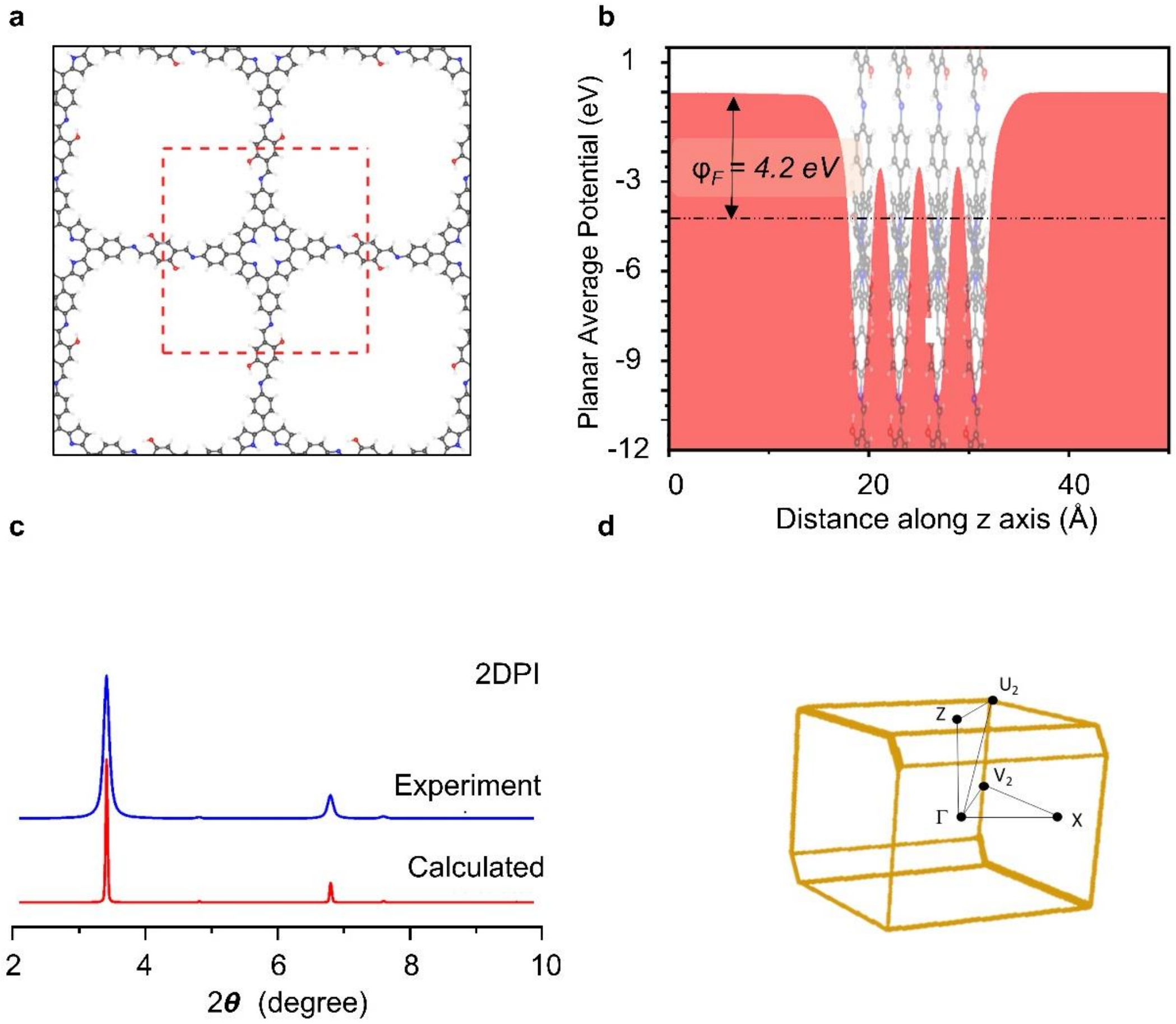

Supplementary Fig. 44 | 2D Polyimine (2DPI). (a) Top view of the four-layer 2DPI slab. (b) One-dimensional planar-averaged electrostatic potential along the z-direction. (c) Experimental diffraction profile (blue) obtained from the in-plane GIWAXS signal of the 2DPI and converted from Q_{xy} to 2θ for direct comparison with the calculated pattern (red) simulated from the structural model. (d) Representation of the Brillouin zone.

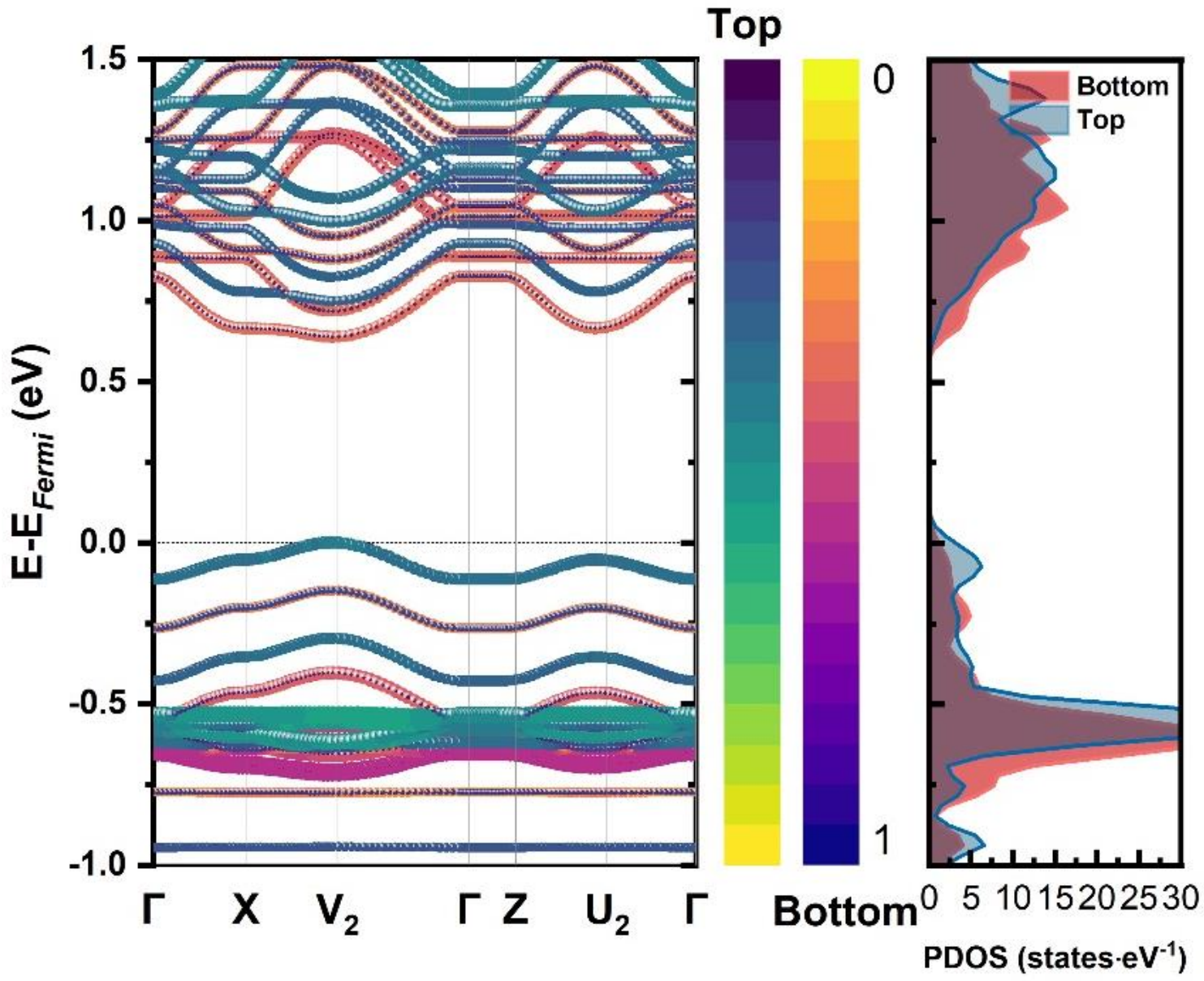

Supplementary Fig. 45 | 2D Polyimine (2DPI). Band structure of the 2DPI slab, separated into contributions from the top two and bottom two layers. (Right) Partial density of states (PDOS) of the 2DPI slab**.**

Structure and electronic property of lattice matched vdW heterostructure.

2DPI-2DPBI vdW Heterostructure

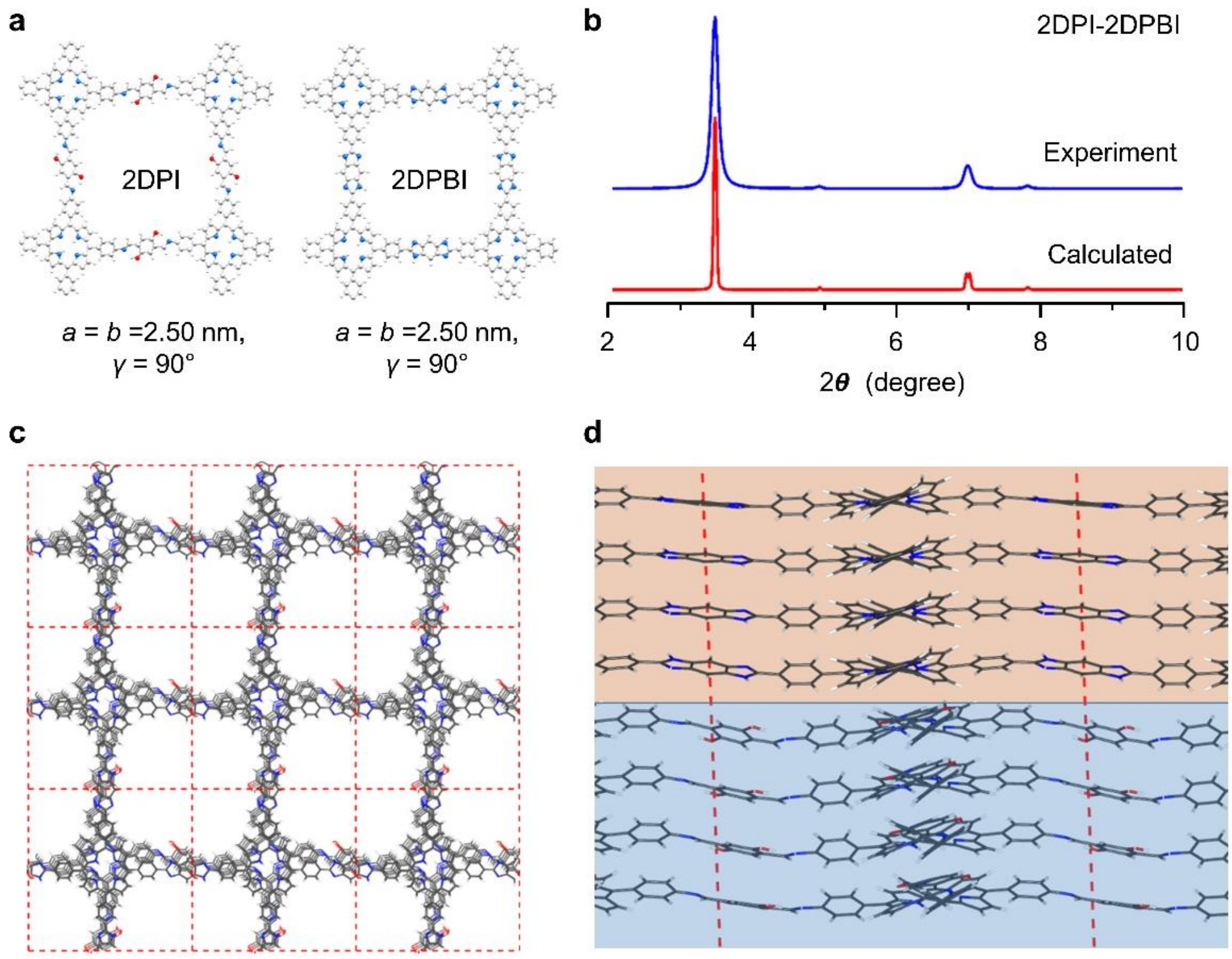

Supplementary Fig. 46 | 2DPI-2DPBI vdW heterostructure. (a) Parent 2DPs comprising 2D Polyimine (2DPI) and 2D Polybenzimidazole (2DPBI). (b) Experimental diffraction profile (blue) obtained from the in-plane GIWAXS signal of the 2DPI-2DPBI vdW heterostructure and converted from Q_{xy} to 2θ for direct comparison with the calculated pattern (red) simulated from the structural model. (c) Top view and (d) Side view (bottom, blue box: 2DPI; top, orange box: 2DPBI) of the 2DPI-2DPBI vdW heterostructure, as visualized using VESTA.

2DPI-2PBT vdW Heterostructure

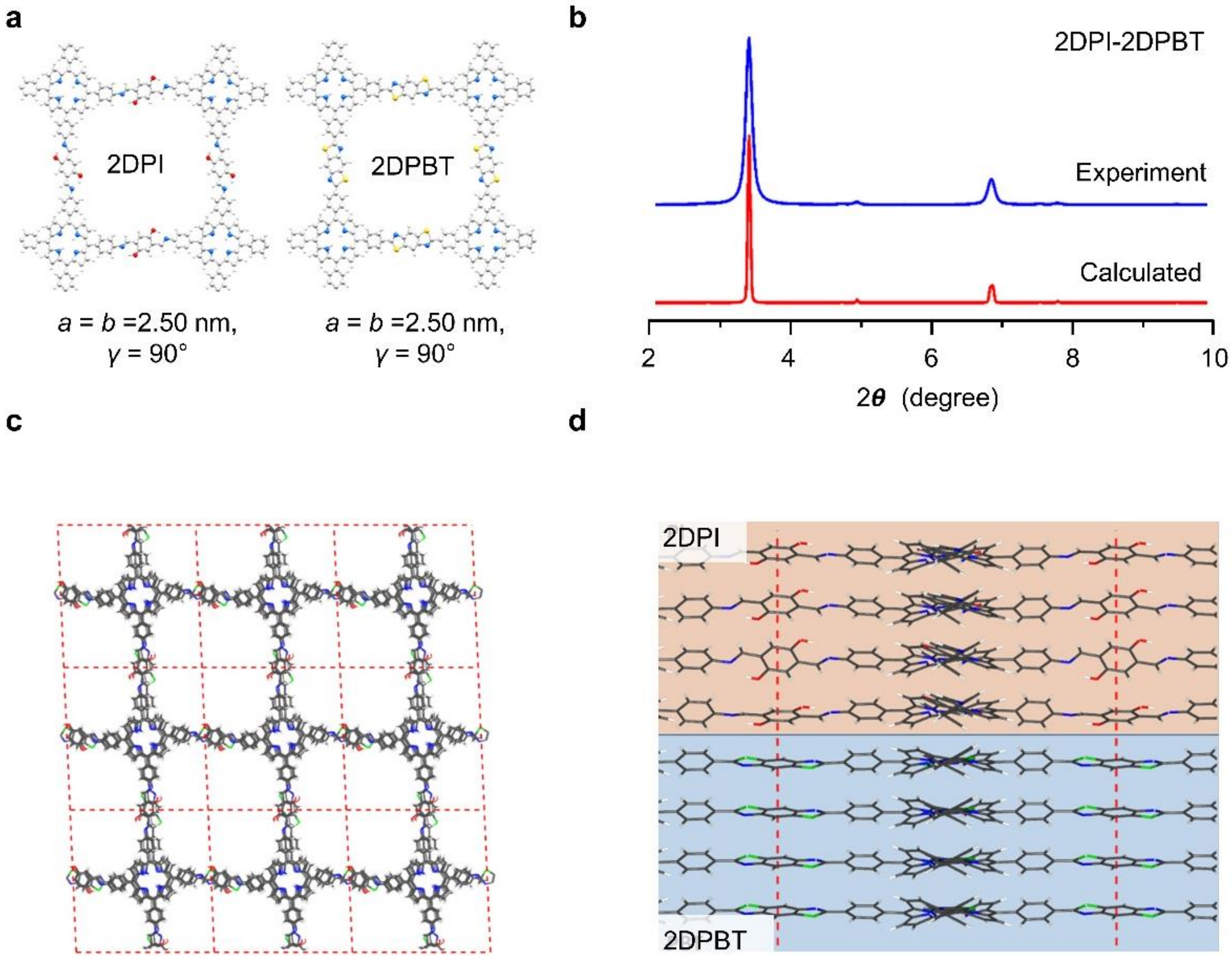

Supplementary Fig. 47 | 2DPI-2DPBT vdW heterostructure. (a) Parent 2DPs comprising 2D Polyimine (2DPI) and 2D Polybenzothiazole (2DPBT). (b) Experimental diffraction profile (blue) obtained from the in-plane GIWAXS signal of the 2DPI-2DPBT vdW heterostructure and converted from Q_{xy} to 2θ for direct comparison with the calculated pattern (red) simulated from the structural model. (c) Top view and (d) Side view (bottom, blue box: 2DPBT; top, orange box: 2DPI) of the 2DPI-2DPBT vdW heterostructure, as visualized using VESTA.

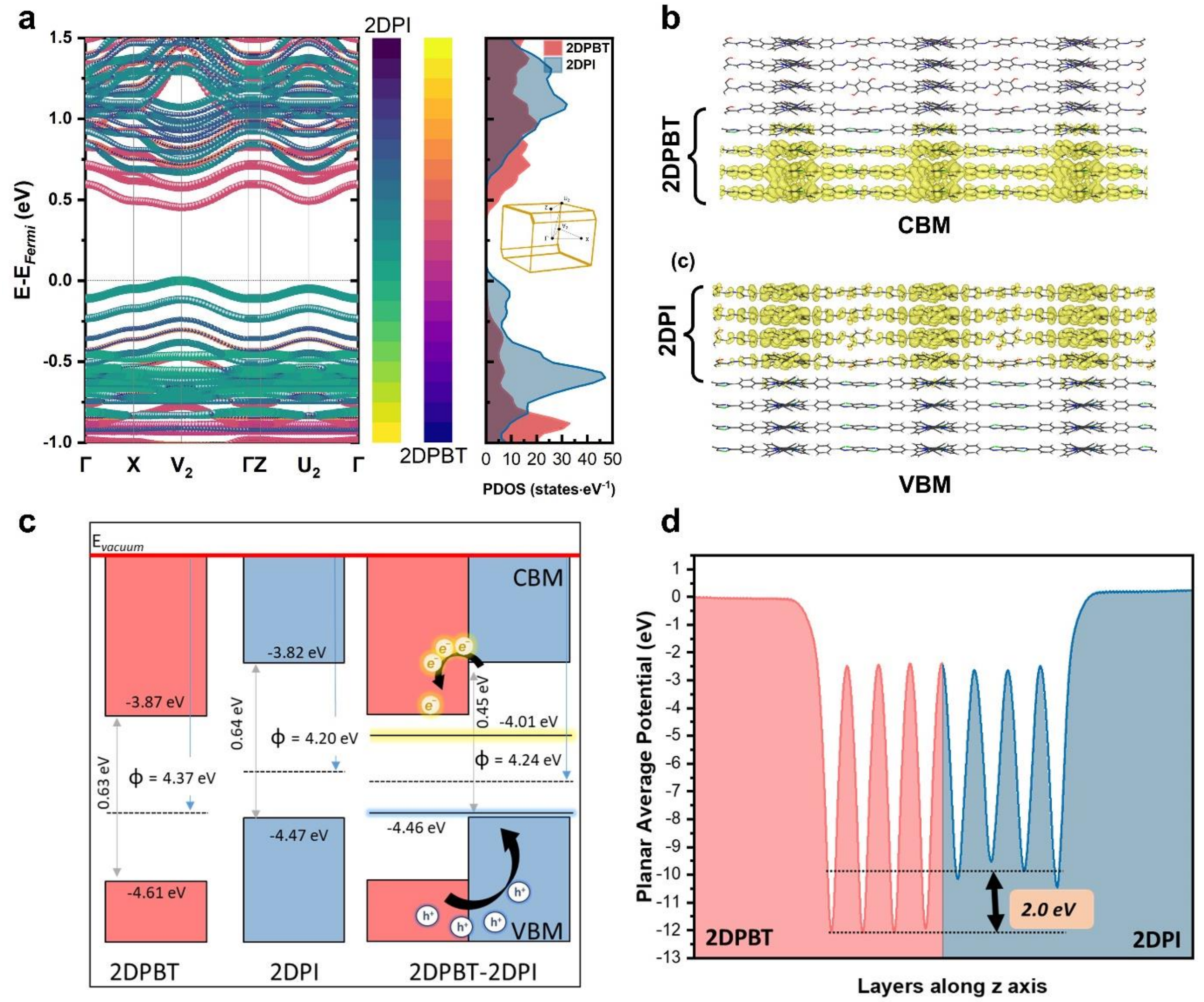

Supplementary Fig. 48 | 2DPI-2DPBT vdW heterostructure. (a) (left) Band structure (right) PDOS (inset) Brillouin zone. (b) band decomposed charge densities (Bottom) VBM localized at the 2DPI layer, (top) CBM localized at the 2DPBT layer with an isosurface value of 1.6×10^{-3}. (c) Schematic representation of band alignment at the 2DPI-2DPBT heterojunction, with Φ denoting the work function. (d) Planar-averaged electrostatic potential along the c-axis direction, highlighting the potential variation across the heterojunction where the 2DPBT is represented by red region and 2DPI is represented by blue region.

2DPBT-2PBI vdW Heterostructure

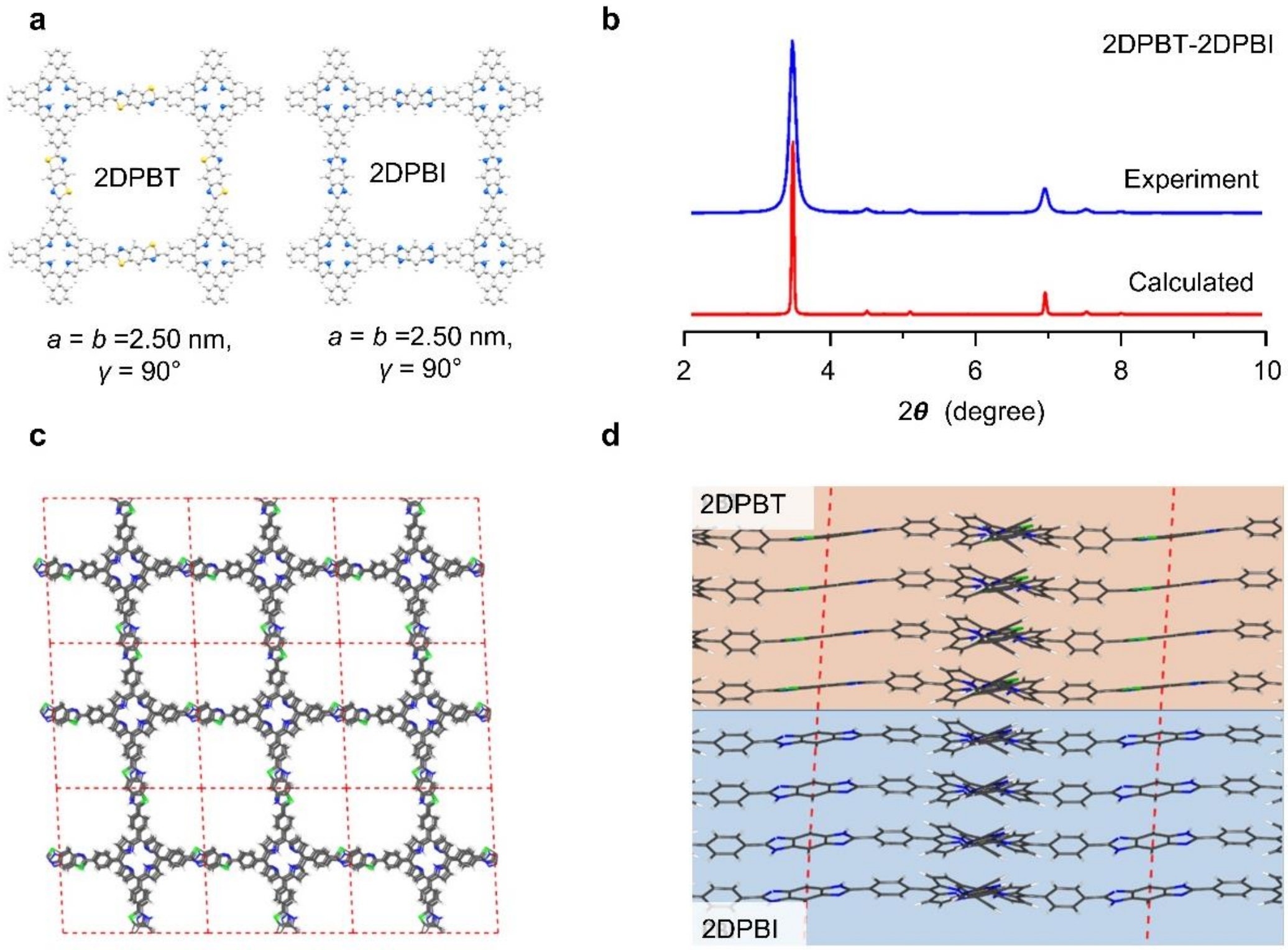

Supplementary Fig. 49 | 2DPBI-2DPBT vdW heterostructure. (a) Parent 2DPs comprising 2D Polybenzimidazole (2DPBI) and 2D Polybenzothiazole (2DPBI). (b) Experimental diffraction profile (blue) obtained from the in-plane GIWAXS signal of the 2DPBT-2DPBI vdW heterostructure and converted from Q_{xy} to 2θ for direct comparison with the calculated pattern (red) simulated from the structural model.. (c) Top view and (d) Side view (bottom, blue box: 2DPBI; top, orange box: 2DPBT) of the 2DPBT-2DPBI vdW heterostructure, as visualized using VESTA.

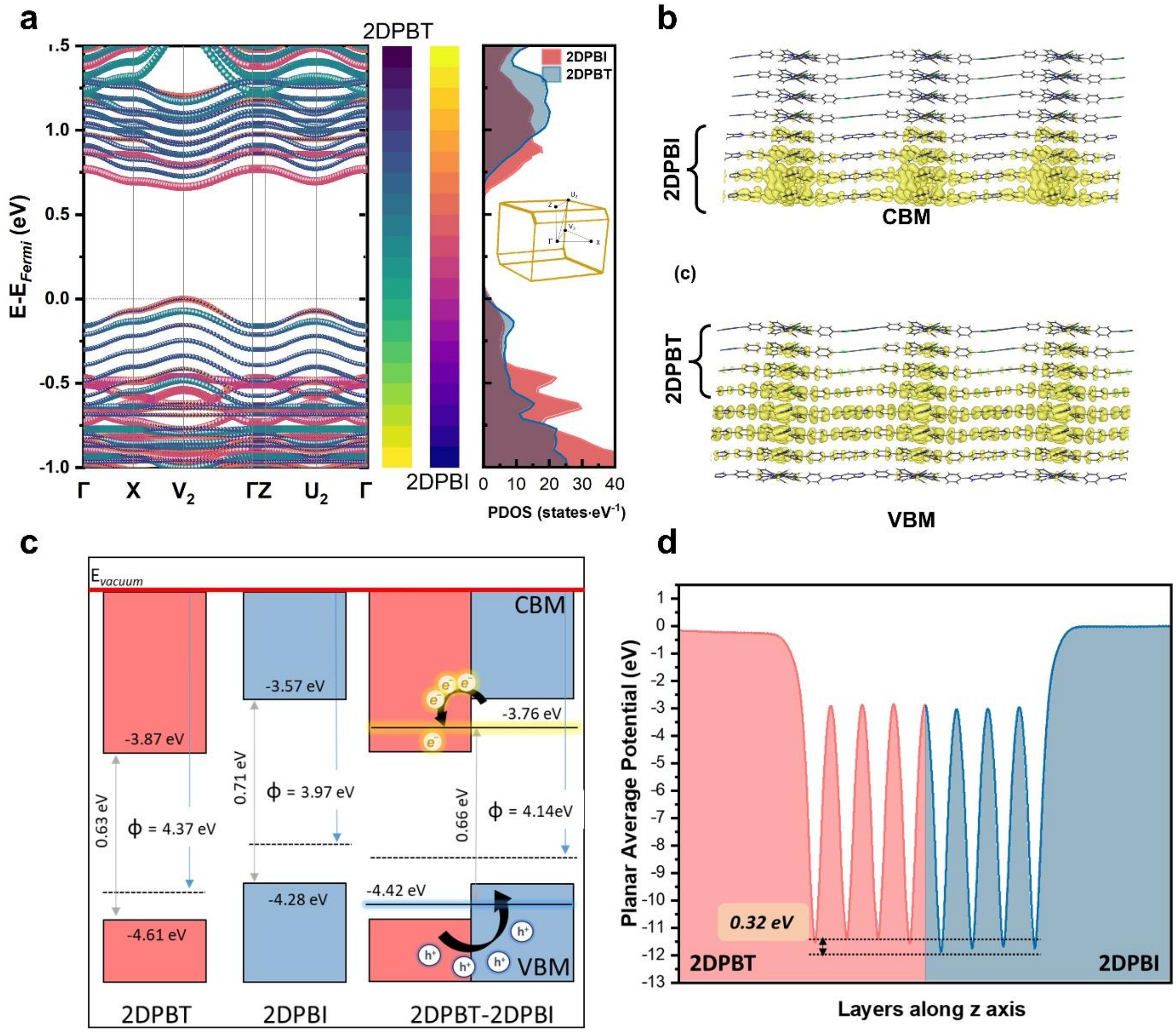

Supplementary Fig. 50 | 2DPBI-2DPBT vdW heterostructure. (a) (left) Band structure (right) PDOS (inset) Brillouin zone. (b) band decomposed charge densities (Bottom) VBM localized majorly at the 2DPBI layer and on 2DPBT layer, (top) CBM localized only at the 2DPBI layer with an isosurface value of 1.6×10^{-3}. (d) Planar-averaged electrostatic potential along the c-axis direction, highlighting the potential variation across the heterojunction where the 2DPBI is represented by red region and 2DPBT is represented by blue region.

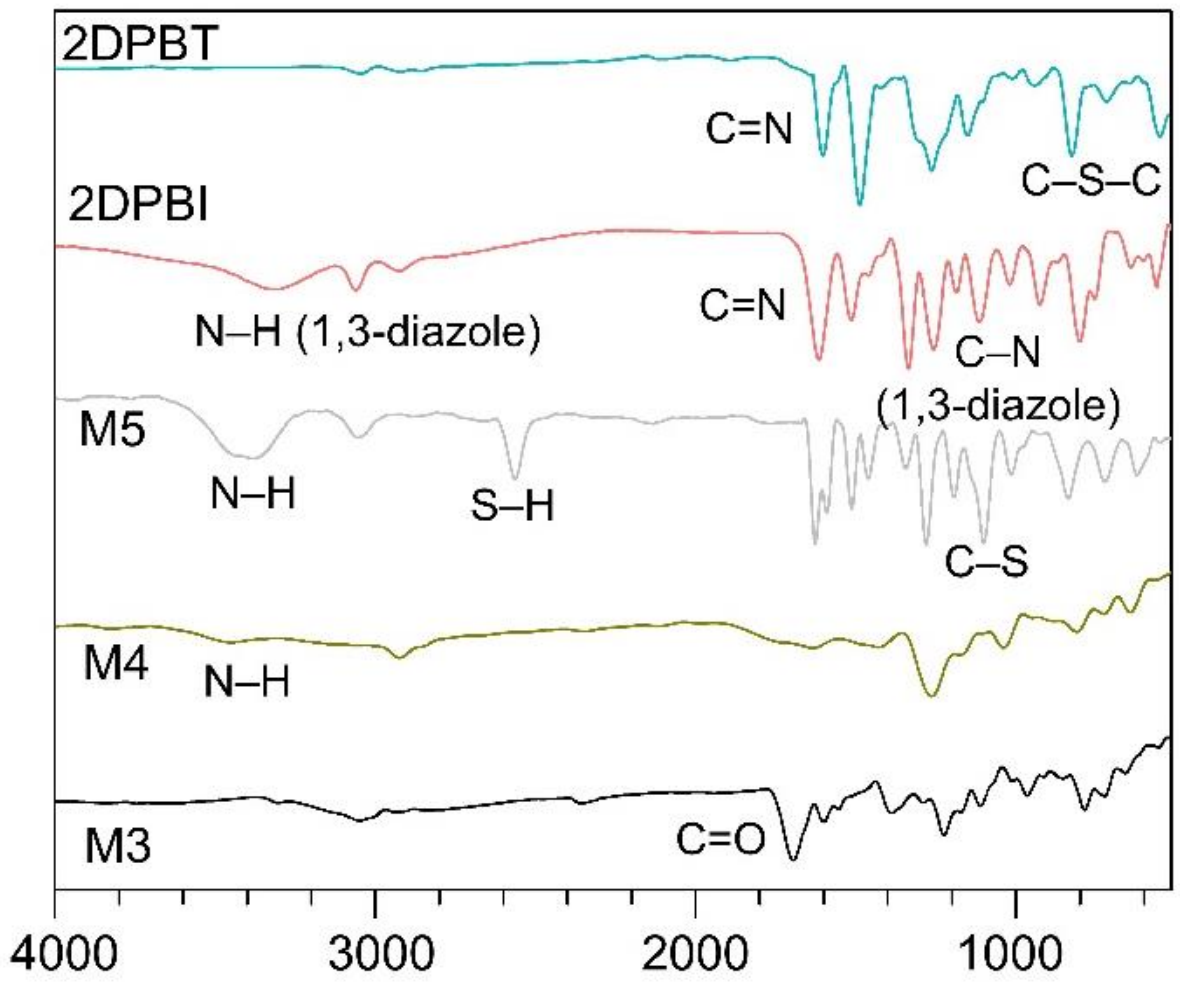

Supplementary Fig. 51 | FTIR characterization of 2DPBT and 2DPBI formation. FTIR spectra of precursor monomers M3–M5 and the corresponding 2D polymers.

For 2DPBI, comparison with the precursor monomers M3 and M4 shows that the N–H band of monomer M4 shifts from 3370 cm^{-1} to 3312 cm^{-1}, consistent with hydrogen-bonded N–H in the benzimidazole ring. A new band appears at 1622 cm^{-1}, assigned to C=N stretching in the benzimidazole linkage, while the C=O band of monomer M3 at 1708 cm^{-1} disappears, confirming formation of the benzimidazole-linked 2D polymer. For 2DPBT, comparison with monomers M3 and M5 shows the disappearance of the N–H band (3430 cm^{-1}) and the S–H band (2564 cm^{-1}). New bands appear around 1610 cm^{-1} (C=N) and 710 cm^{-1} (C–S–C), confirming benzothiazole linkage formation. Additionally, the C=O band from M3 at 1708 cm^{-1} disappears, confirming complete conversion to the benzothiazole-linked 2D polymer.

Azole and imide formation typically requires high temperature or activation in conventional bulk reactions. In our synthesis approach, however, the reactions occur through on-water surface synthesis, where the on-water surface effect and surfactant-confined pre-assembly play key roles in enhancing reactivity and selectivity. This has been supported by model reactions reported in our previous studies. For the imidazole model reaction, the reaction between R1 and R2 on the CTAB-confined water surface at room temperature gave the target benzimidazole product MC-1 with m/z = 730.48, as confirmed by MALDI–TOF MS, whereas the same reaction in water without surfactant gave only the starting reagents[45]. Similar behaviour was observed for imide formation, where imidization proceeded at room temperature on the water surface, while the same reaction in aqueous solution did not give the product[44].

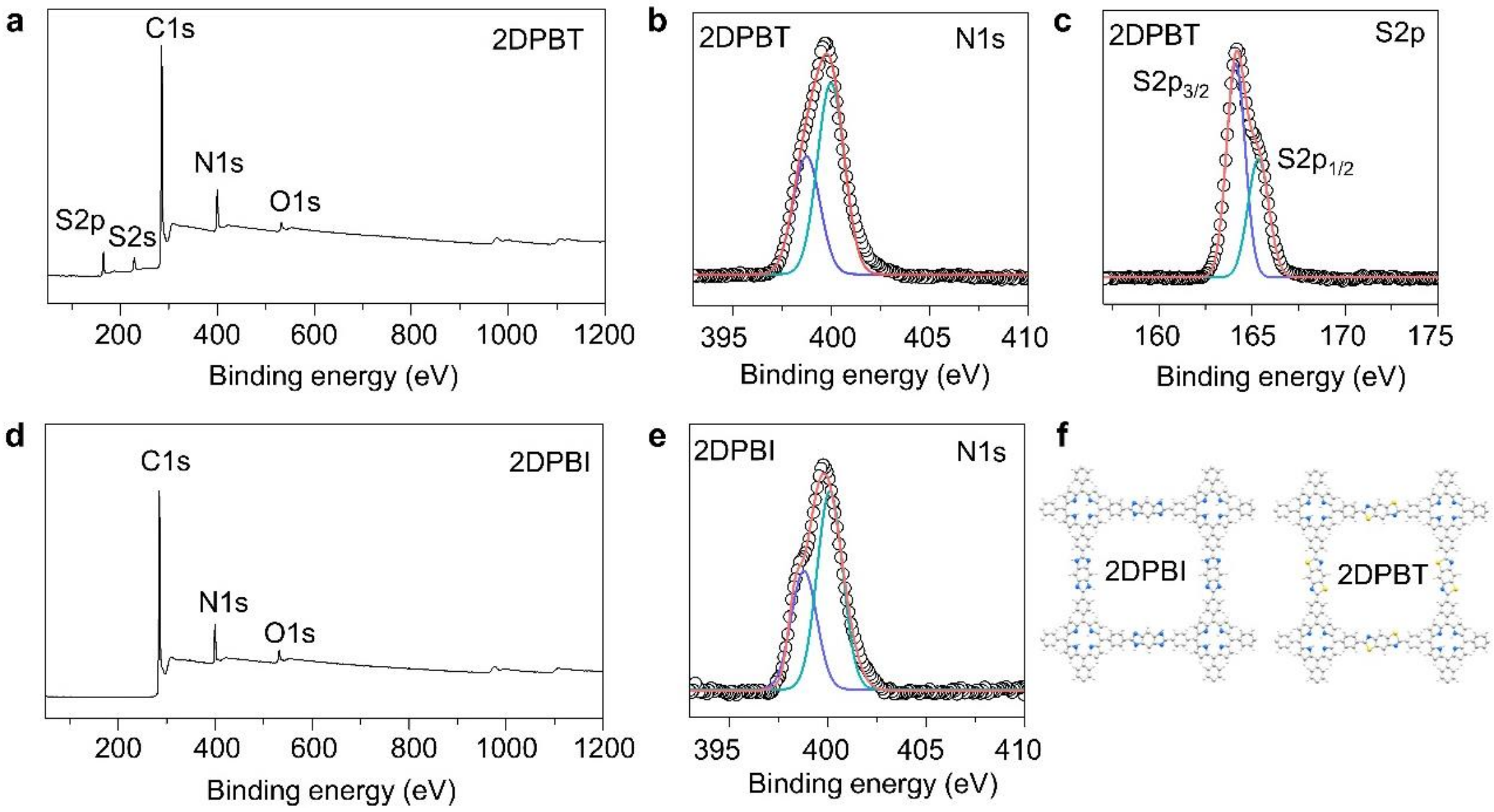

Supplementary Fig. 52 | XPS characterization of 2DPBT and 2DPBI. a–c, XPS survey, N 1s and S 2p spectra of 2DPBT, showing imine/amine nitrogen components and the characteristic S 2p doublet. **d,e,** XPS survey and N 1s spectra of 2DPBI, resolving imine- and amine-type nitrogen environments. **f,** Structural models of 2DPBI and 2DPBT.

XPS analysis further supports the formation of the diazole-linked 2D polymers. For 2DPBI, the N 1s spectrum was deconvoluted into two components at 398.5 and 400.1 eV, assigned to imine- and amine-type nitrogen atoms in the 1,3-diazole linkage, respectively. Similarly, 2DPBT showed N 1s components at 398.6 and 400.0 eV, corresponding to imine- and amine-type nitrogen environments in the thiazole–diazole linkage. The S 2p spectrum of 2DPBT displayed a characteristic spin–orbit doublet with S $2p_{3/2}$ and S $2p_{1/2}$ peaks at 164.1 and 165.3 eV, respectively, consistent with chemically bound sulfur in the polymer backbone.

Regarding the work function and Fermi level placement: When describing 2D periodic systems in a 3D periodic cell, the work function is formally defined as:

$$\varphi = V\_{vacuum} - E\_{Fermi}$$

The band alignment calculations with rigorous reference to the vacuum plateau extracted from the planar-averaged electrostatic potential (LOCPOT) following the standard VASP protocol (φ = V_vacuum − E_Fermi). The corrected work function of the 2DPI-2DPBI heterostructure is φ = 4.065 eV, with the Fermi level correctly placed at -4.065 eV with respect to vacuum, sitting within the bandgap above the VBM at -4.29 eV and below the CBM at -3.84 eV, consistent with Figure 4a. The individual layer work functions, obtained from separate independent calculations for each isolated layer, are φ_2DPI = 4.203 eV and φ_2DPBI = 3.973 eV. The work function offset of 0.23 eV between the two constituent layers drives Fermi-level equilibration upon contact, with electrons transferring from 2DPBI (lower φ) to 2DPI (higher φ), establishing the common Fermi level at -4.065 eV. This charge transfer is consistent with the charge density difference analysis (Figure 4d), which shows electron depletion from 2DPBI and accumulation on 2DPI, with a net transfer of approximately 0.05 e per unit cell. Figure 4a has been revised accordingly.

Regarding the two different work functions on opposite faces of the film:

The two faces of the heterostructure are not electrostatically equivalent. The work function of φ = 4.065 eV is a single bulk quantity computed for the heterostructure as a whole. The individual bulk work functions of φ_2DPI and φ_2DPBI are obtained from separate independent calculations and reflecting the intrinsic electronic structure of each polymer network. The interfacial dipole moment of 0.93 e·Å (surface dipole density: 1.45×10^{-3} e/Å, Figure 4e), oriented normal to the plane of the layers and determined from a single-point calculation with dipole correction applied (LDIPOL = T, DIPOL = 0.5 0.5 0.5), gives rise to a built-in potential step of 2.11 eV across the interface (Figure 4b). From the bulk work function, the interfacial dipole moment, and the built-in potential step, we conclude that the surface potential differs on the two faces of the heterostructure, consistent with the reviewer's observation. This asymmetry is a direct physical consequence of the interfacial dipole rather than an independently computed surface quantity. We further note that the built-in potential step of 2.11 eV significantly exceeds the work function difference of 0.23 eV between the isolated layers, indicating that the dominant contribution to the electrostatic asymmetry arises from the interfacial dipole alignment rather than from Fermi-level equilibration alone. This is well established in organic heterojunction systems where interfacial molecular dipoles routinely produce potential steps far exceeding the contact potential, and is the central physical result underpinning the charge transport properties reported. The calculated potential step of 2.11 eV was obtained from a 4+4 layer model,

which closely approximates the experimental film thickness. In the experimental structure, where the polymer films may extend beyond the thickness of the computational model, the macroscopic built-in potential experienced across the full device may be modulated by additional electrostatic screening from layers further from the interface.

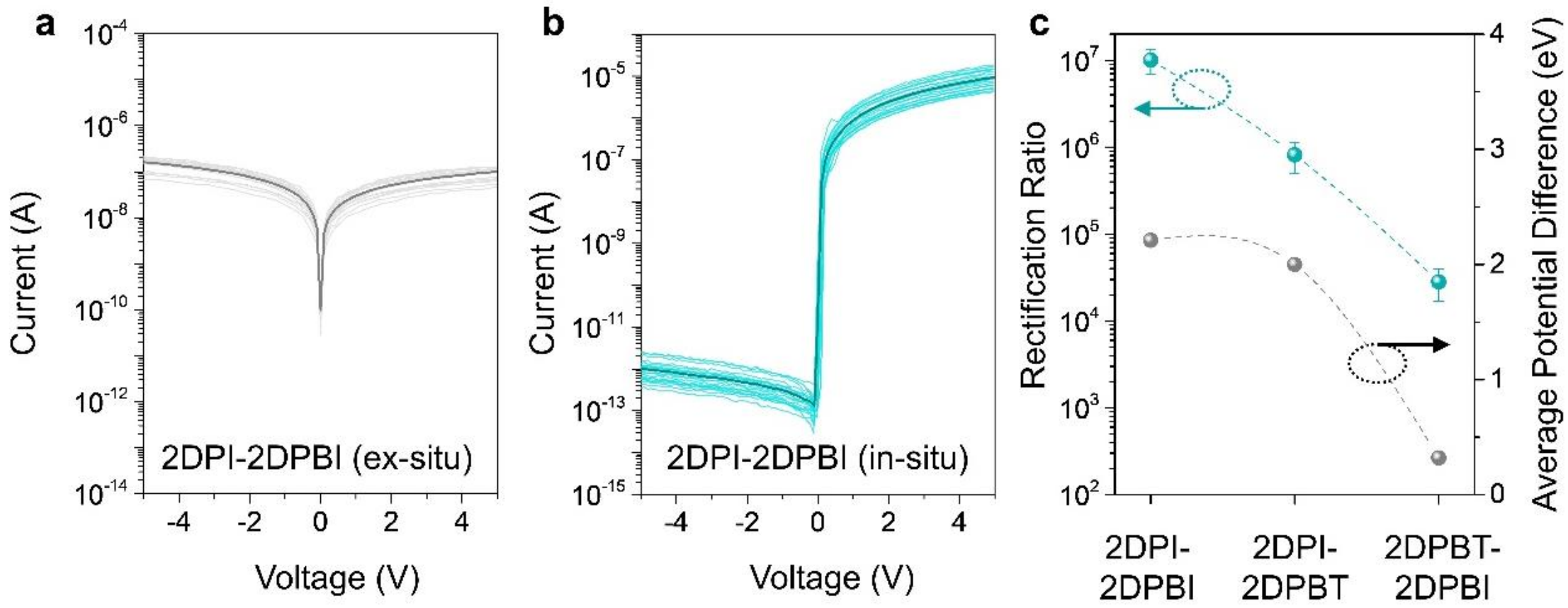

Supplementary Fig. 53 | Statistical transport analysis of vertical micro-devices. (a,b) I–V curves of fabricated vertical micro-devices based on ex-situ and in-situ 2DPI–2DPBI vdW heterostructures. (c) Statistical comparison of rectification ratio and average potential difference for different 2D polymer vdW heterostructure devices.

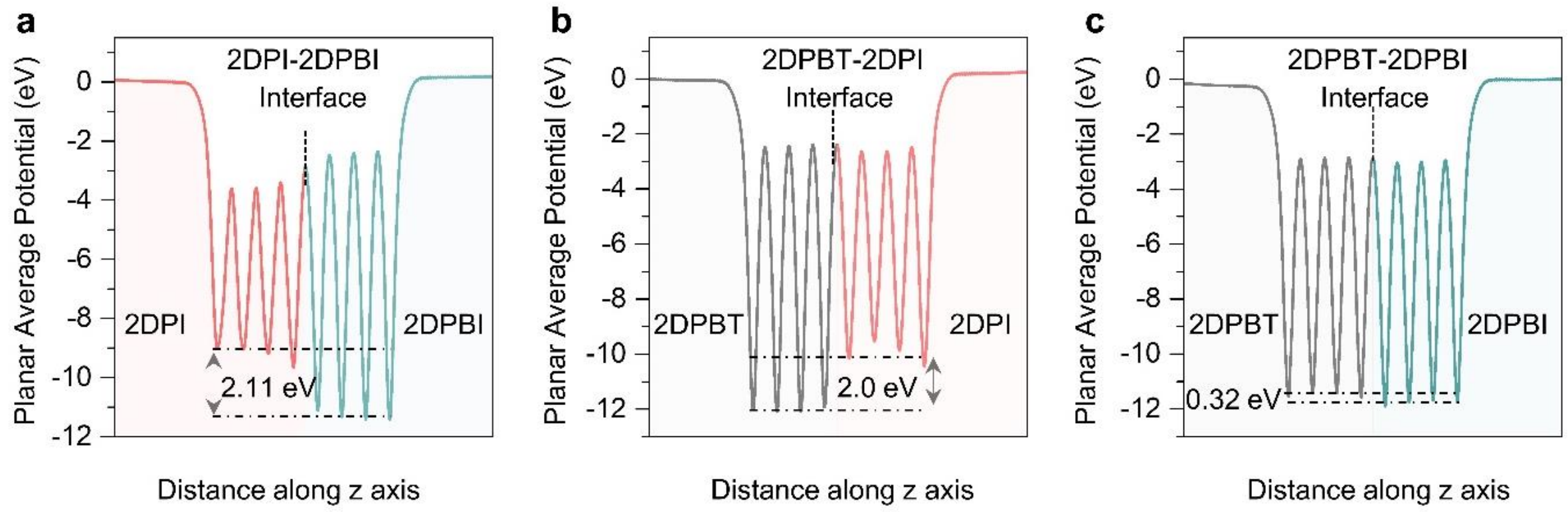

Supplementary Fig. 54 | Planar-averaged electrostatic potentials of lattice-matched 2D polymer vdW heterostructures. Planar-averaged electrostatic potential profiles along the stacking direction for (a) 2DPI–2DPBI, (b) 2DPBT–2DPI and (c) 2DPBT–2DPBI vdW heterostructures, showing built-in potential differences of 2.11, 2.0 and 0.32 eV, respectively.

The planar-averaged potential was calculated from the DFT electrostatic potential by averaging over the lateral **xy** plane along the stacking direction:

$$\bar{V}(z_0) = \frac{1}{A}\int_A V(x, y, z_0)\, dx\, dy$$

where **A** is the surface area of the unit cell. The average potential difference is defined as the potential step between the two sides of the heterostructure slab. This value reflects the built-in electrostatic asymmetry generated by the interfacial dipole.

We calculated this value for all three lattice-matched heterostructures: 2.11 eV for 2DPI–2DPBI, 2.0 eV for 2DPBT–2DPI, and 0.32 eV for 2DPBT–2DPBI. These theoretical values are plotted on the right y-axis of Fig. 5h and compared with the experimentally measured rectification ratios. The correlation shows that a larger interfacial potential difference is associated with stronger rectification.

This quantity is meaningful for heterostructures because it arises from the electrostatic asymmetry between two chemically distinct 2D polymers. For an individual 2D polymer, the two slab surfaces are equivalent and no interfacial potential step is expected.

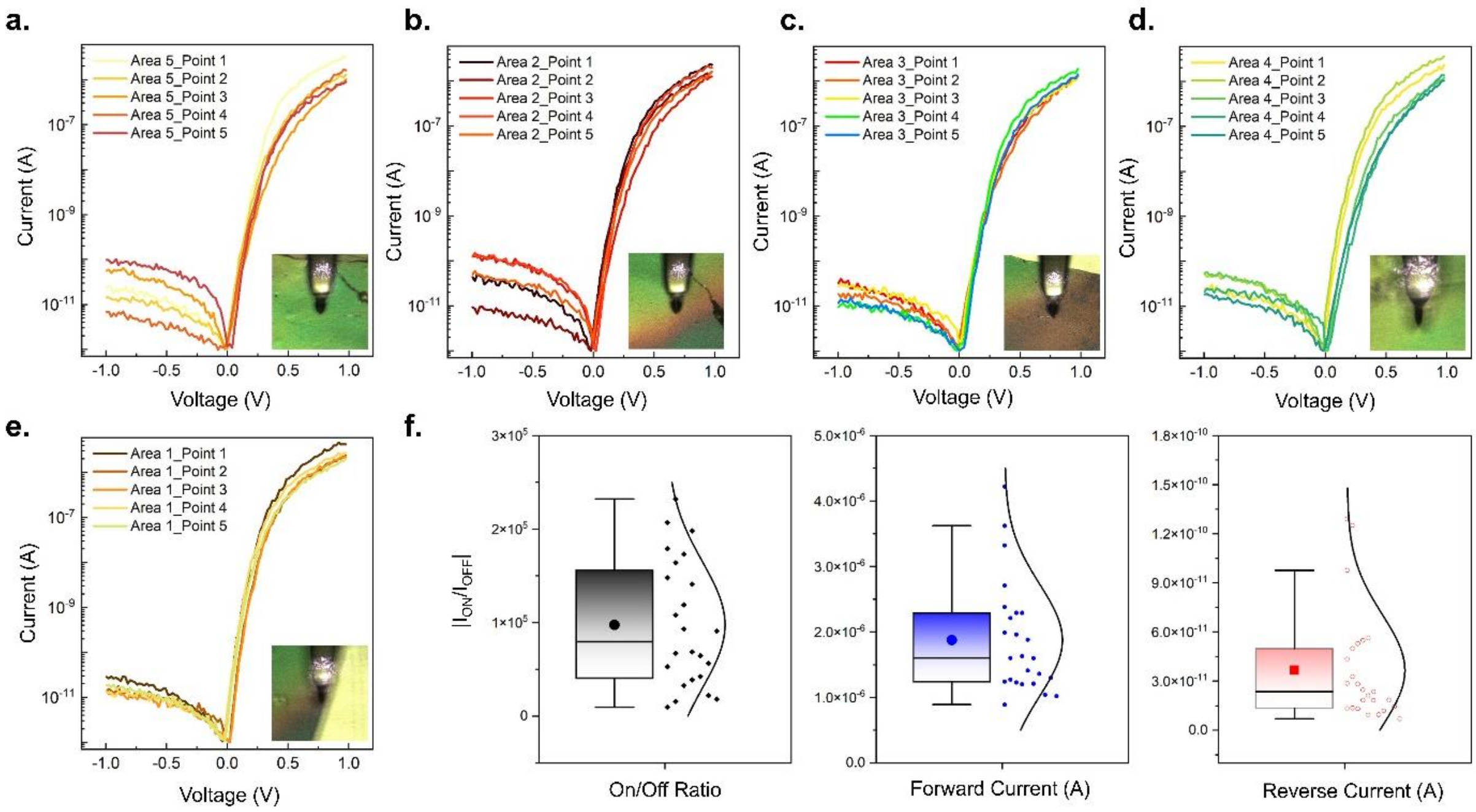

Supplementary Fig. 55 | Statistical c-AFM I–V characterization of 2DPI–2DPBI vdW heterostructures. (a–e) c-AFM I–V curves measured from five independent 2DPI–2DPBI vdW heterostructure samples, with five spatially distinct points collected from each sample. Insets show optical images of the cantilever approaching the sample surface. (f) Statistical analysis of the extracted transport parameters, including rectification ratio, forward current, and reverse current.

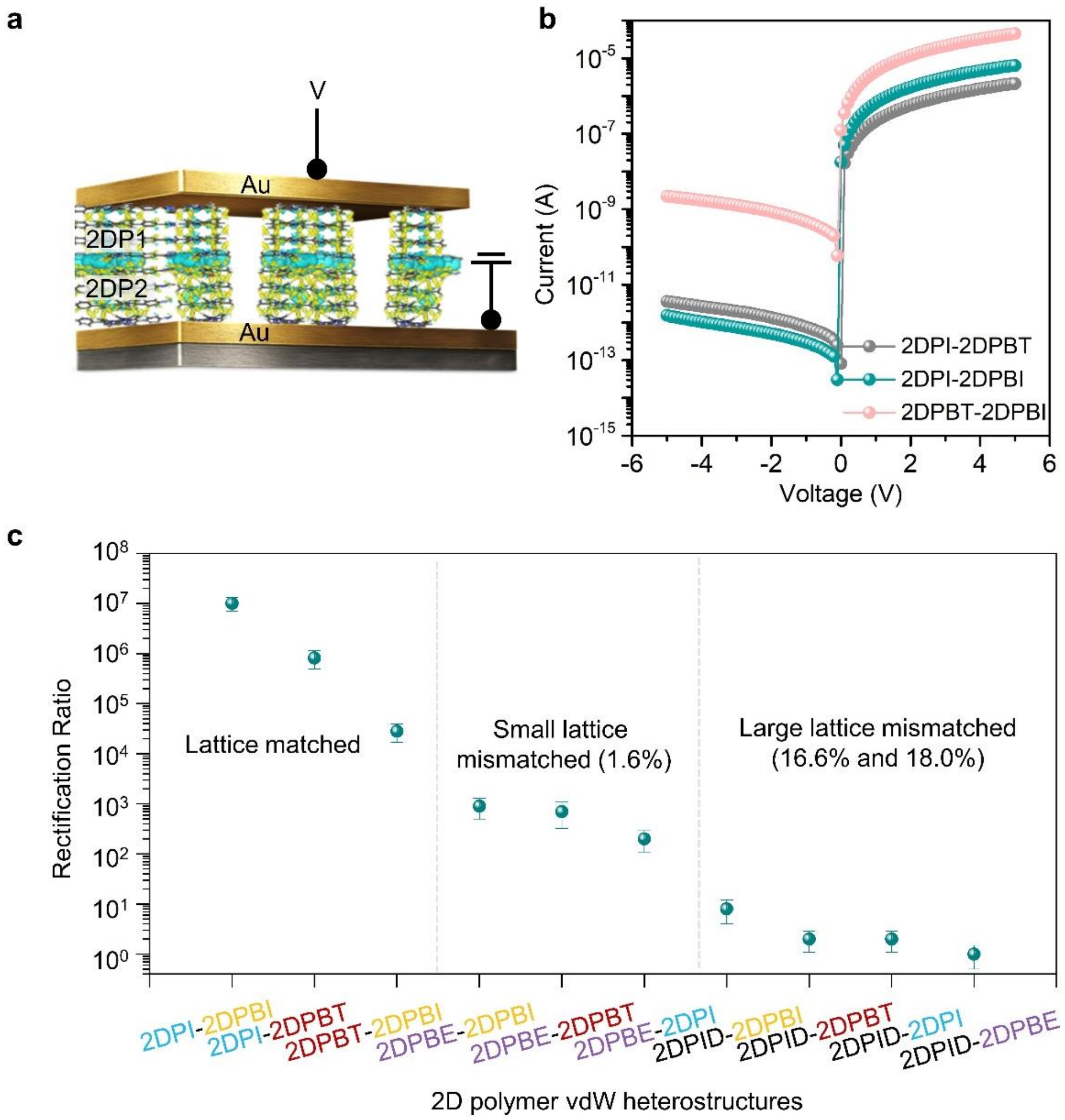

Supplementary Fig. 56 | Vertical charge transport and diode-like rectification in 2D polymer vdW heterostructures. (a) Schematic of the vertical heterojunction device geometry (Au/2DP1–2DP2/Au). (b) Current–voltage (*I*–*V*) characteristics of representative lattice-matched 2D polymer vdW heterostructures. (c) Rectification ratio summary for 2D polymer vdW heterostructures grouped by lattice mismatch: lattice-matched pairs exhibit rectification ratios of 10^7–10^4, small-mismatch pairs (1.6%) show reduced ratios of 10^3–10^2, and large-mismatch pairs (16.6% and 18.0%) approach ~10, indicating that coherent lattice alignment promotes efficient vertical charge transport and strong rectifying behavior.

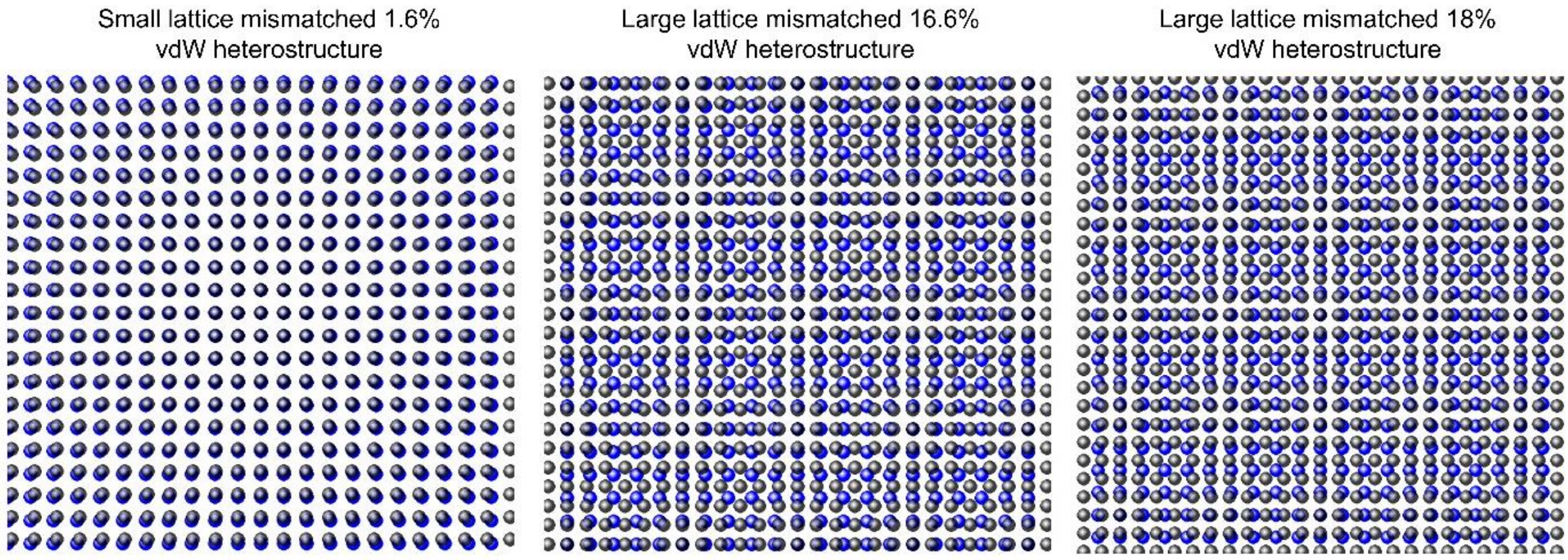

Supplementary Fig. 57 | Structural models of lattice-mismatched 2D polymer vdW heterostructures. Schematic models showing the interfacial lattice registry for small-mismatch and large-mismatch vdW heterostructures, illustrating the reduced density of closely matched interfacial sites with increasing lattice mismatch.

In the in-situ synthesized 2DPI–2DPBI vdW heterostructure, the commensurate epitaxial alignment enables directional interfacial charge redistribution between the two 2D polymer layers. The calculated built-in potential step of 2.11 eV and interfacial dipole moment of 0.93 e·Å, oriented normal to the layers, arise from this asymmetric charge transfer. Such a coherent interfacial dipole is favored when the two lattices are in epitaxial registry, because the frontier-orbital overlap across the interface is spatially well defined.

In contrast, in the ex-situ mechanically stacked 2DPI-2DPBI heterostructure, the two layers have random rotational orientations. This produces non-uniform local registry and disordered interfacial orbital overlap. As a result, local dipole contributions are not coherently aligned across the device area, strongly reducing the macroscopic built-in electrostatic asymmetry. This explains the nearly symmetric conductance observed for the ex-situ heterostructure.

The vertical charge-transport measurements of the lattice-mismatched heterostructures further support the role of lattice registry. The rectification ratio is highest for lattice-matched heterostructures, reaching 10^7–10^4, decreases to 10^3–10^2 for the small-mismatch heterostructure, and approaches only ~10 for large-mismatch heterostructures. In the small-mismatch case, partial local registry is still preserved, allowing some interfacial orbital overlap. In contrast, for 16.6% and 18.0% lattice-mismatched heterostructures, the relative registry changes strongly across the Moiré pattern, reducing the density of closely matched interfacial sites. This weakens coherent interfacial charge redistribution and the built-in electrostatic asymmetry, leading to much weaker rectification.

References

1. Elstner, M. & Seifert, G. Density functional tight binding. *Philosophical Transactions of the Royal Society A: Mathematical, Physical and Engineering Sciences* **372** (2014).
2. Te Velde, G. t. *et al.* Chemistry with ADF. *Journal of Computational chemistry* **22**, 931-967 (2001).
3. Baerends, T. *et al. ADF, ADF2019, SCM, Theoretical Chemistry, Vrije Universiteit, Amsterdam, The Netherlands, 2016*
4. Gaus, M., Cui, Q. & Elstner, M. DFTB3: Extension of the self-consistent-charge density-functional tight-binding method (SCC-DFTB). *Journal of chemical theory and computation* **7**, 931-948 (2011).
5. Kresse, G. & Hafner, J. Norm-conserving and ultrasoft pseudopotentials for first-row and transition elements. *Journal of Physics: Condensed Matter* **6**, 8245-8257 (1994).
6. Sun, G. *et al.* Performance of the Vienna ab initio simulation package (VASP) in chemical applications. *Journal of Molecular Structure: THEOCHEM* **624**, 37-45 (2003).
7. Perdew, J. P., Burke, K. & Ernzerhof, M. Generalized gradient approximation made simple. *Physical review letters* **77**, 3865 (1996).
8. Blöchl, P. E. Projector augmented-wave method. *Physical review B* **50**, 17953 (1994).
9. Momma, K. & Izumi, F. VESTA: a three-dimensional visualization system for electronic and structural analysis. *Applied Crystallography* **41**, 653-658 (2008).
10. Prasoon, A. *et al.* Site-selective chemical reactions by on-water surface sequential assembly. *Nat. Commun.* **14**, 8313 (2023).
11. Mannsfeld, S. C. B. & Fritz, T. Advanced modelling of epitaxial ordering of organic layers on crystalline surfaces. *Modern Physics Letters B* **20**, 585-605 (2006).
12. Mannsfeld, S. B. & Fritz, T. Analysis of the substrate influence on the ordering of epitaxial molecular layers: The special case of point-on-line coincidence. *Physical Review B* **69**, 075416 (2004).
13. Mannsfeld, S. C. B., Leo, K. & Fritz, T. Line-on-line coincidence: a new type of epitaxy found in organic-organic heterolayers. *Physical review letters* **94**, 056104 (2005).
