# Supplementary material for: Organic two-dimensional van der Waals heterostructures
Source: Nature. 2026 Sep 23;658(8135):388–97. doi: 10.1038/s41586-026-11074-6 (PMC13645628; doi:10.1038/s41586-026-11074-6)
Supplement: Supplementary file 2 [file 41586_2026_11074_MOESM2_ESM.pdf]

nature portfolio

Peer Review File

Programmable Lattice Engineering of Organic Two-Dimensional van der Waals Heterostructures

Corresponding Author: Professor Xinliang Feng

Any redactions in this file are there to maintain patient confidentiality, the confidentiality of unpublished data, or to remove third-party material.

This file contains all reviewer reports in order by version, followed by all author rebuttals in order by version.

Version 0:

Reviewer comments:

Referee #1

(Remarks to the Author)
This manuscript reports an interesting sequential interfacial assembly strategy for preparing vdW heterostructures from different 2D polymers with varying lattice-match degrees. The authors demonstrated unique properties like charge separation, photoluminescence quenching, and ultrafast carrier dynamics for the lattice-matched heterostructures. Diode-like rectification with high ON/OFF ratios is also demonstrated for the lattice-matched ones. While the concept of lattice-matched and mismatched heterostructures is compelling, the experimental evidence largely focuses on the lattice-matched 2DPI-2DPBI heterostructure. Structural characterizations for the lattice-mismatched heterostructures, such as GIWAXS, GIXD, and HRTEM, are not provided. The lack of experimental confirmation raises major concerns about whether the proposed large-mismatch heterostructures can be formed as proposed. In addition, key aspects of sequential interfacial assembly remain unclear. For instance, it is not verified whether the first layer is fully dense and continuous, or whether potential defects and large pore sizes of the first layer could allow the second layer to form on the same plane instead of forming a bilayered heterostructure. Overall, this manuscript could be considered for publication only if all the major and minor concerns below can be well addressed:
Major concerns:
1. The concept of lattice-matched (0%) and mismatched (16.6%, 18.0%) heterostructures is based on theoretical structures of the 2D polymers used in this work. However, only the lattice-matched (0%) 2DPI-2DPBI vdW heterostructure is experimentally investigated with the results of GIXD, GIWAXS, and HRTEM. The lattice-matched heterostructure might be the easiest one to demonstrate. The experimental evidence for the mismatched heterostructures, particularly their GIWAXS and HRTEM data, are also of great significance to support the concept of this manuscript.
2. The surfactant monolayer plays a central role in guiding the interfacial assembly and epitaxial growth of the 2D polymer films. However, the rationale behind the use of this specifical surfactant is not fully discussed. In addition to the anionic surfactant used in this work, is it possible to use cationic surfactants? How will their charge properties affect the interfacial assembly of 2D polymer films?
3. The HRTEM images in Figure 2b and Supplementary Figure 18 show grain boundaries and discontinuities within the 2D polymer film. Will such structural defects affect interfacial electronic coupling and out-of-plane electrical transport? Moreover, the observation of such grain boundaries may indicate disordered adsorption of monomers during Sept-II. Could this structural nonuniformity be avoided by optimizing the synthesis conditions?
4. The manuscript proposes a transition from point-on-line to line-on-line epitaxy during sequential assembly. However, the driving force for this evolution remains insufficiently clarified. Moreover, is this mechanism universal for different monomers, or does it rely on specific lattice systems?
5. For large lattice-mismatched 2D polymer vdW heterostructures (16.6% and 18.0% mismatch), it is unclear how interfacial strain and structure are accommodated. Did the authors observe any moire fringes for these cases given their significant lattice mismatch degrees?
6. In layer-on-layer heterostructures, diffraction contributions from individual layers may overlap. The authors should clarify how overlapping GIWAXS and GIXD signals are assigned to each layer, particularly for those heterostructures with large lattice-mismatch degrees.
7. Thickness control over the developed heterostructures from 4.6 to 30 nm is discussed. However, there are no experimental results to support this discussion.

8. How can the authors experimentally confirm that the first layer is fully dense and continuous? Could the monomers for the second layer diffuse through the relatively large pores of the first layer and polymerize on top of the first layer, rather than exclusively beneath it? Could potential defects in the first layer cause the second layer to nucleate and grow on the same plane, rather than forming a layer-on-layer heterostructure?
9. The authors claimed that "Bragg reflections were observed at the same Qxy and Qz positions in the GIWAXS diffraction, with further intensity enhancement, confirming that the epitaxial alignment was preserved throughout." However, based on the current GIWAXS images, it is challenging to conclude the above results as the scattering signals are relatively weak and unclear, possibly due to the low thickness of the heterostructure. Can the authors increase the film thickness and provide better GIWAXS data for clearer comparison?
10. The bandgap of 2DPI-2DPBI vdW heterostructure was calculated to be 0.45 eV, which is smaller than that of the individual 2DPI and 2DPBI. The authors attributed this reduction to strong electronic coupling across the interface. How can this electronic coupling be formed without directional bonding between two layers?
Minor issues:
1. The authors reported the use of five 2DPs, including polyimine, polyimide, polybenzimidazole, polybenzothiazole, and polyboronate ester. However, the framework of polyboronate ester polymers is often chemically unstable, especially in moisture, aqueous solutions, and air. Will the polyboronate ester-based film be decomposed after synthesis? How about its chemically stability?
2. In the synthesis of polymer films, is the SOS surfactant directly spreading on water surface? Or is it dissolved in organic solvents like chloroform as described in their previous studies (Nat Commun 2023, 14, 8313)?
3. To further confirm the absence of amorphous domains in the prepared heterostructures, it is suggested to provide additional HRTEM images from different film regions.

Referee #2

(Remarks to the Author)
This is a tour de force work in controlling the structure of 2D materials at a molecular level. Combining their signature "on-water" polymerization with a full toolbox of state-of-the-art characterizations, Xinliang Feng and coauthors report the synthesis of planar heterostructures of 2D semiconducting polymers and demonstrate record-large current rectification, which appears to depend on the lattice matching between the two semiconductor films. In my opinion, the work is very significant and certainly highly original (first of its kind in 2D polymer materials). I have a number of questions and comments (below), but I expect the revised manuscript to be publishable in Nature.
1) One of the apparent limitations of this 'on-water' polymerization (compared to polymerization on crystalline surfaces) is the inability to produce polymer monolayers. What limits the lower end of the polymer thickness (reported 4.5 nm, ~10 layers), i.e., what happens at lower monomer concentration? Are there any step-edges observed by AFM? (also, the AFMs, eg Fig.S21, should be accompanied by the line height profile)
2) The XPS depth profiling is used to support the atomically sharp interface in the heterostructure (Fig. S22). Such a significant shift seems a bit surprising given that the structure contains eight/twelve (DPI/PBI) nitrogen atoms per unit cell, and only 4 of them would be shifted to higher BE, going from DPI to DPBI. Can the protonation (acid trapping) in the lower layer be excluded (especially given the relatively high pKa of imidazole)? Fig. S22 should be supplemented with 1D spectra of the top and bottom of the film, deconvoluted. Also, it would be highly desirable to see full XPS spectra and the elemental (N/O) ratio along the film depth.
3) I am confused by the discussion of the (calculated) interfacial band alignment. The manuscript suggests a work function of 4.81 eV, which should place the Fermi level below the VBM =-4.29 eV (although Fig. 4 shows it above the VBM??). I suppose such lowering of the Fermi level is possible due to charge transfer, but it also highlights that the structure should have two different workfunctions on the opposite sides of the film.
4) The transport measurements are performed using conductive AFM. The big advantage of this approach is the ability to rapidly acquire a significant amount of data on a given sample, but only one curve is shown per structure/condition. A statistical analysis of the current and rectification ratio should be reported (and the uncertainties included in Fig. 5h). A major limitation of c-AFM measurements is that the junction characteristics depend strongly on the tip-surface separation, and an asymmetric conductance (although probably not 10^7) can be observed under certain conditions even in a perfectly symmetric film. The set point of the measurement should be reported (also, the tip diameter).
5) A remarkable and somewhat unexpected (to me at least) finding of the work is that the rectification depends on the epitaxy between the layers, which is supported by the symmetric conductance of the 'ex-situ' prepared DPI/DPBI heterostructure with a random layer orientation. However, I did not find any explanation for this behavior. The manuscript (line 360) implies that random orientation leads to "absence of interfacial dipole," but it's not clear to me why this would be the case (or how the authors established this point). Also, the measurements of "lattice MIS-matched" films are not discussed in the manuscript; Fig. S37 is important but is 'hidden' in the SI.

Further points:
-- The facile formation of benzimidazole from carboxylic acid and phenylenediamine at RT would be surprising for many chemists (it usually requires high temperatures and activation). I don't dispute the results (WAXS and the MALDI-TOF of the molecular product in ref. 46 seem convincing); however, the manuscript would benefit from some explanation of this unusually facile reaction (in the SI). Also, if the DPDBI (and DPBT) polymers/COFs prepared under THESE conditions have not been reported before, it would be great to include spectral evidence for the reaction (e.g., FTIR, XPS).
--why the assembled M1 shows rings in GIWAXS? It implies a disordered crystal orientation, which seems to contradict the presented cartoons of the assembly.
-- Line 255: "This behavior is likely due to the ultrafast formation of highly localized excitonic states, which exhibit negligible

conductivity." The phrasing is confusing, as the localized exciton should form with a similar rate in the homogenous films as well (followed by possible charge separation). Overall, the stated lack of photoconductivity may need further explanation/context. The photoinduced formation of charge carriers is commonly observed in single-phase organic materials (likely facilitated by defects), and, if I am not mistaken, has been widely employed to probe charge mobility of similar homogenous Schiff base COFs. What's different in this case?
-- Fig. 3d seems important but is not discussed. Are the differences between the absorption profiles of 2DPI and 2DPBI due to different stacking (it seems unlikely that these are due to different in-plane conjugation)? Can the authors exclude the possible difference in the porphyrin protonation state (given the reaction performed in HCl solution…)?
--What data is the "average potential difference" in Fig. 5h based on? Depth profiling UPS? (it's only shown for one heterostructure, what about the other two?)
--The SI needs more details on sample preparation, e.g. volume of water, amount of SOS (and method of spreading – in some solvent?) in step I; approximate # of aqueous layer exchanges between steps? Exact conditions (reaction time) for the other 9 heterostructures?
-- Fig. S9 caption gives 2D unit cell of the self-assembled monomer with reference to "indexed reflections", but no indexing is shown on the figure, and I am not sure what it refers to (the peaks observed in Fig. S9d seem to represent the SOS layer along).
-- Rated to the above: pls, complement the figure with 1D diffraction patterns as it's often impossible to see the discussed diffraction peaks on the 2D GIWAXS images (Figs. 2, S12)

Referee #3

(Remarks to the Author)
The manuscript presents an extensive and carefully executed body of experimental work on organic vdW heterostructures based on 2D polymers. The combination of sequential on-water-surface assembly and structural characterization is impressive, and the authors clearly demonstrate a high level of control over layer stacking, lattice registry, and interfacial properties.

However, despite the breadth and technical sophistication of the study, it remains unclear whether the proposed strategy constitutes a fundamentally new conceptual advance with the potential to significantly reshape the field. In particular, the bottom-up assembly approach on air-water interfaces seems to function primarily as an enabling technique rather than offering new mechanistic insight into interlayer interactions or emergent phenomena unique to organic 2D systems.

Experimental approach and key analytic methods are already published by the same authors in "On-water surface synthesis of electronically coupled 2D polyimide-MoS2 van der Waals heterostructure. Commun Chem 6, 280 (2023)" and "On-water surface synthesis of crystalline, few-layer two-dimensional polymers assisted by surfactant monolayers, Nat. Chem. 11, 994–1000 (2019)". In particular, surfactant-monolayer-assisted interfacial synthesis (SMAIS) use a water surface to pre-organize monomers, creating well-defined 2D polymers with large crystalline domains has been established and the current approach is largely expected.

The authors synthesized and performed in situ structural analysis of the 2D polymers sequentially (Step I to Step V) on the water surface. This reviewer assumes that thermal annealing was carried out prior to device measurements of final organic vdW heterostructures to remove residual or trapped water, which is not described in either the main text or the Supporting Information. What is the effect of water molecules bound to the polymer backbones on lattice parameters and the degree of lattice mismatch in these 2D polymer assemblies?

The authors identify three commensurate, lattice-matched pairs (2DPI–2DPBI, 2DPI–2DPBT, and 2DPBT–2DPBI), which raises some concerns. Polybenzimidazole (PBI) is known to be hygroscopic, whereas polybenzothiazole (PBT) is comparatively hydrophobic, and both are grown on polyimine (PI), which exhibits intermediate water affinity. It is therefore unclear how epitaxial growth on a water surface can proceed in a commensurate manner despite such differences in water uptake behavior. How can the in situ GIWAXS data (wet state) be in good agreement with the HR-TEM results obtained in the dry state? Residual water could act as a dopant, enhance charge transfer, or induce swelling, all of which may significantly alter the intrinsic electronic or semiconducting properties of these materials.

As shown in Supplementary Fig. 9, the in situ GIWAXS profiles are fully isotropic, indicating that the pre-assembled M1 layer is randomly oriented and only becomes ordered upon polymerization. This appears inconsistent with the schematic illustrations throughout the main text and Supplementary Figs. In fact, Figure 2d suggest that the M1 pre-assembly is already organized via point-on-line coincidence, with polymerization represented simply by adding connecting lines without altering the monomer arrangement. This results in a discrepancy between the experimental observations and the schematic representations. Specifically, it is unclear how a randomly pre-assembled M1 layer can transform into an ordered M2 array through polymerization. This is not related to a commensurate epitaxial interface, as the high glass transition temperature of conjugated polymers severely limits chain mobility, making structural rearrangement extremely slow.

In summary, while the study constitutes a technical achievement and presents a comprehensive dataset, the authors should more clearly articulate its broader conceptual impact on the field.

Version 1:

Reviewer comments:

Referee #1

(Remarks to the Author)
The authors have carried out extensive additional experiments and provided satisfactory responses to all of my previous concerns. The revised manuscript has been significantly improved and now presents a compelling study of organic vdW heterostructures. I therefore recommend its publication in Nature.

Referee #2

(Remarks to the Author)
The authors have amde a major effort in revising the manuscript. All significant issues raised in the original set of reviews have been well-addressed. I recommend accepting the revised manuscript in its current form. Congratulations to the authors on this significant breakthrough in the field.

Referee #3

(Remarks to the Author)
The manuscript presents an extensive and carefully executed body of experimental work on organic van der Waals (vdW) heterostructures based on 2D polymers. The combination of sequential on-water surface assembly and structural characterization is highly compelling, and the authors demonstrate a remarkable level of control over layer stacking, lattice registry, and interfacial properties.

In particular, the study advances the development of fully organic 2D polymer–2D polymer vdW heterostructures under surfactant-guided epitaxial registry. This approach provides programmable control over lattice matching and mismatching across a wide range of organic 2D polymer systems.

This reviewer appreciates the authors' thoughtful point-by-point responses to the comments raised and finds that both the quality and clarity of the manuscript have been significantly improved. Accordingly, this reviewer supports its publication in Nature.

Open Access This Peer Review File is licensed under a Creative Commons Attribution 4.0 International License, which permits use, sharing, adaptation, distribution and reproduction in any medium or format, as long as you give appropriate credit to the original author(s) and the source, provide a link to the Creative Commons license, and indicate if changes were made.
In cases where reviewers are anonymous, credit should be given to 'Anonymous Referee' and the source.
The images or other third party material in this Peer Review File are included in the article's Creative Commons license, unless indicated otherwise in a credit line to the material. If material is not included in the article's Creative Commons license and your intended use is not permitted by statutory regulation or exceeds the permitted use, you will need to obtain permission directly from the copyright holder.
To view a copy of this license, visit https://creativecommons.org/licenses/by/4.0/

Point-to-point response to the comments from the reviewers

Referee #1 (Remarks to the Author):

General comment: *This manuscript reports an interesting sequential interfacial assembly strategy for preparing vdW heterostructures from different 2D polymers with varying lattice-match degrees. The authors demonstrated unique properties like charge separation, photoluminescence quenching, and ultrafast carrier dynamics for the lattice-matched heterostructures. Diode-like rectification with high ON/OFF ratios is also demonstrated for the lattice-matched ones. While the concept of lattice-matched and mismatched heterostructures is compelling, the experimental evidence largely focuses on the lattice-matched 2DPI-2DPBI heterostructure. Structural characterizations for the lattice-mismatched heterostructures, such as GIWAXS, GIXD, and HRTEM, are not provided. The lack of experimental confirmation raises major concerns about whether the proposed large-mismatch heterostructures can be formed as proposed. In addition, key aspects of sequential interfacial assembly remain unclear. For instance, it is not verified whether the first layer is fully dense and continuous, or whether potential defects and large pore sizes of the first layer could allow the second layer to form on the same plane instead of forming a bilayered heterostructure. Overall, this manuscript could be considered for publication only if all the major and minor concerns below can be well addressed:*

Response: We greatly appreciate the reviewer's positive and insightful comments, which have been invaluable in revising our manuscript. We have carefully addressed all concerns raised by the reviewer, and have provided point-by-point responses below. We hope that the additional efforts made in the revised manuscript appropriately address the reviewer's concerns.

Major concerns:

1. *The concept of lattice-matched (0%) and mismatched (16.6%, 18.0%) heterostructures is based on theoretical structures of the 2D polymers used in this work. However, only the lattice-matched (0%) 2DPI-2DPBI vdW heterostructure is experimentally investigated with the results of GIXD, GIWAXS, and HRTEM. The lattice-matched heterostructure might be the easiest one to demonstrate. The experimental evidence for the mismatched heterostructures, particularly their GIWAXS and HRTEM data, are also of great significance to support the concept of this manuscript.*

Response: We sincerely appreciate the reviewer's valuable and insightful comment. Following the reviewer's suggestion, we have added new GIWAXS, SAED, and HRTEM data for both the small-mismatch 1.6% and large-mismatch 18.0% 2D polymer vdW heterostructures.

We classify the 2D polymer vdW heterostructures into three types: lattice-matched 0%, small-mismatch 1.6%, and large-mismatch 18.0%. The lattice-matched 0% 2DPI–2DPBI vdW

heterostructure was already characterized in the original manuscript and serves as the reference system. Both 2DPI and 2DPBI have identical square lattice parameters of a = b = 2.50 nm, γ = 90°. Therefore, their diffraction peaks overlap. The SAED pattern shows a single set of bright diffraction spots corresponding to the 2.50 nm lattice periodicity, and GIWAXS/GIXD shows one set of in-plane Bragg peaks at the same Q_{xy} positions, without peak splitting. These results confirm coherent lattice matching in the 0% 2DPI–2DPBI vdW heterostructure.

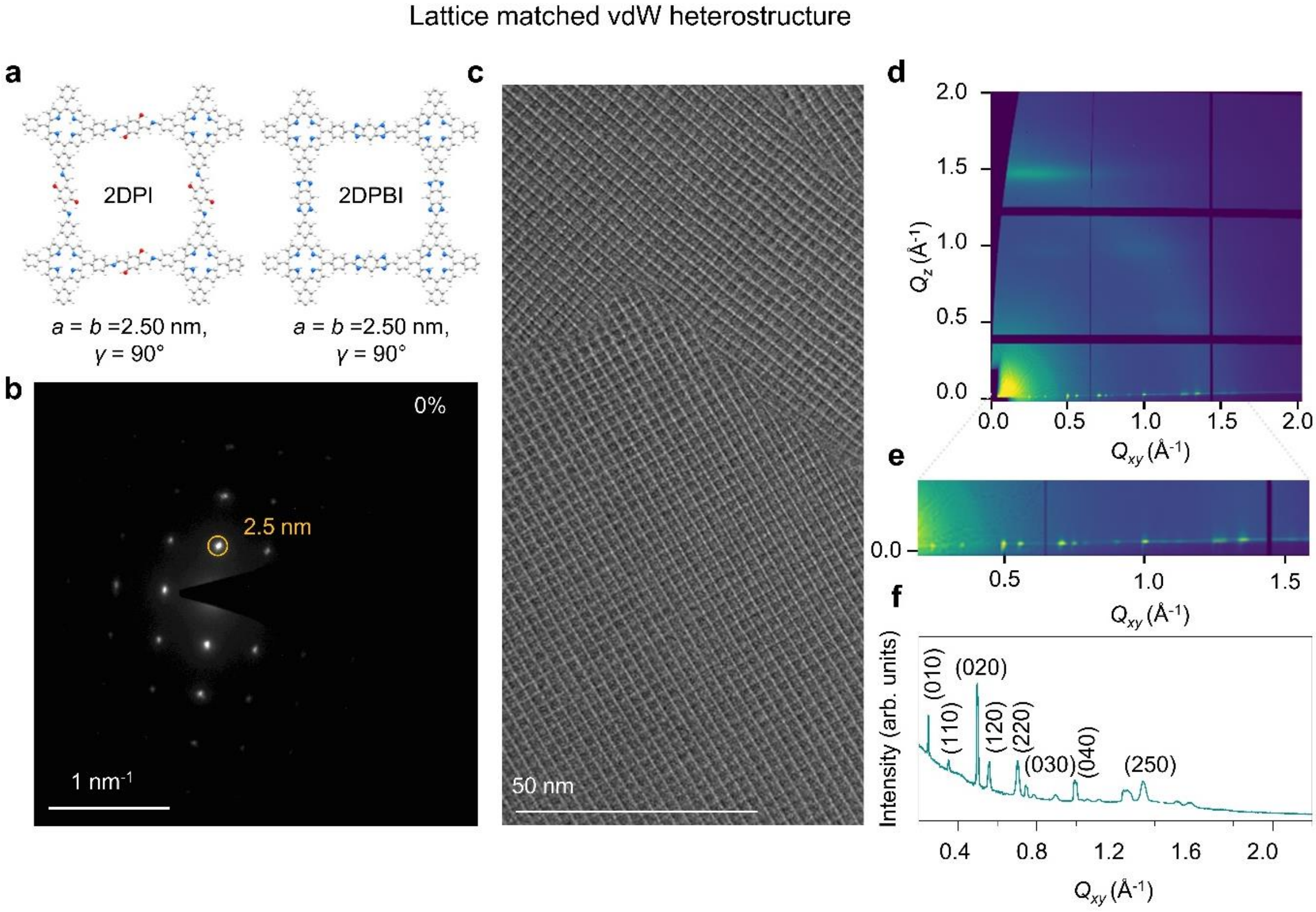

Figure R1 | Structural characterization of the lattice-matched 0% 2DPI–2DPBI 2D polymer vdW heterostructure. (a) Molecular structures of 2DPI and 2DPBI. (b) SAED pattern showing a single set of diffraction spots corresponding to 2.5 nm periodicity. (c) HRTEM image showing ordered lattice domains. (d,e) GIWAXS pattern and enlarged in-plane region. (f) Integrated in-plane GIWAXS profile near $Q_z = 0$, showing the indexed Bragg reflections.

For the small-mismatch 1.6% 2DPI–2DPBE vdW heterostructure, the two 2D polymers have slightly different lattice parameters: a = b = 2.50 nm for 2DPI and a = b = 2.46 nm for 2DPBE. Because of this small lattice difference, the diffraction peaks split into two closely spaced peaks rather than appearing as a single peak. The GIWAXS image (Fig. R2c) shows two distinct peaks at Q_{xy} = 0.251 and 0.255 $Å^{-1}$, corresponding to the (100) lattice periodicities with spacings of 2.50 and 2.46 nm, respectively. The same peak separation with double the distance is therefore also observed for the (200) reflections at $Q_{xy} \approx 0.503$ and 0.511 $Å^{-1}$. Consistently, SAED shows closely spaced double spots corresponding to 2.50 and 2.46 nm, confirming the coexistence of both lattices in the small-mismatch 1.6% vdW heterostructure (Figure R2). HRTEM further shows continuous ordered crystalline domains without

pronounced warping of the 2D polymer layers. A few isolated knot-like defects are observed, as marked by the yellow arrows, while large-area HRTEM images show no amorphous regions at the domain boundaries (Figure R3).

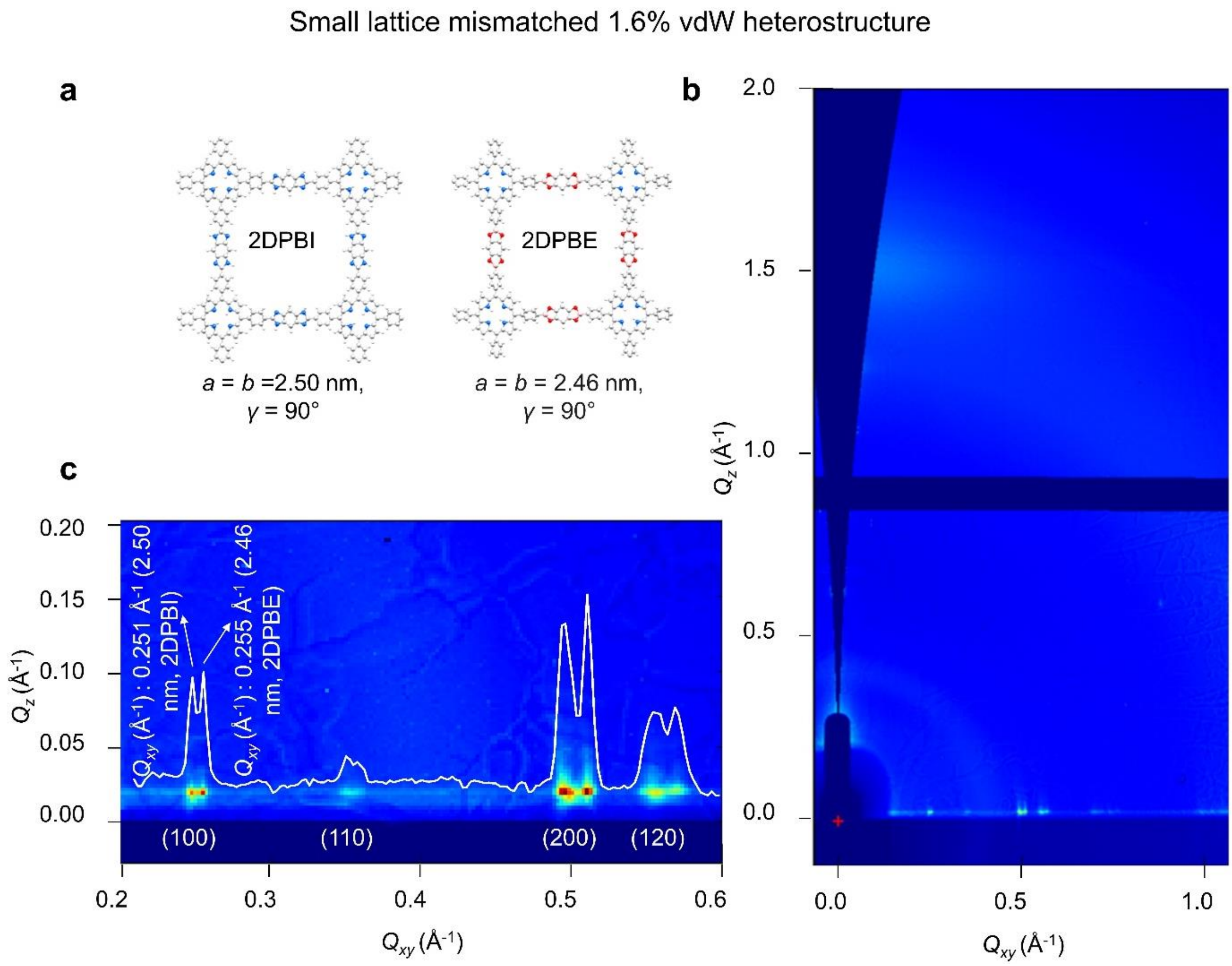

Figure R2 | GIWAXS characterization of the small-mismatch 1.6% 2DPBI–2DPBE 2D polymer vdW heterostructure. (a) Molecular structures of 2DPBI (a = b = 2.50 nm, γ = 90°) and 2DPBE (a = b = 2.46 nm, γ = 90°). (b) 2D GIWAXS pattern. (c) Enlarged in-plane GIWAXS region near $Q_z = 0$, showing split (100) peaks at $Q_{xy} = 0.251$ and 0.255 $Å^{-1}$, corresponding to lattice periodicities of 2.50 and 2.46 nm, respectively, with the splitting retained at higher-order reflections.

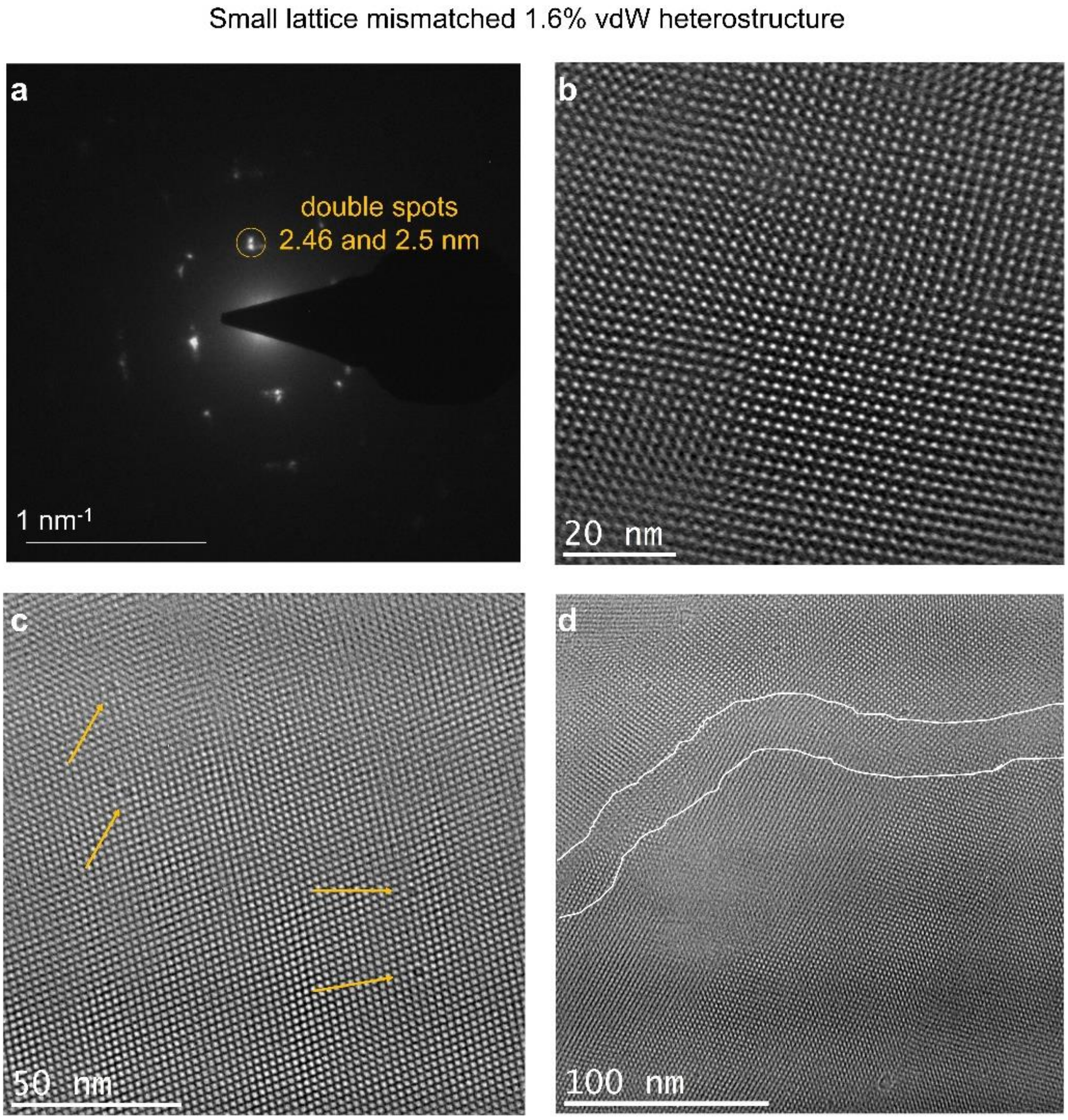

Figure R3 | SAED and HRTEM characterization of the small-mismatch 1.6% 2DPBI–2DPBE 2D polymer vdW heterostructure. (a) SAED pattern showing closely spaced double spots corresponding to 2.50 and 2.46 nm lattice periodicities. (b) HRTEM image showing continuous ordered crystalline domains without pronounced warping of the 2D polymer layers. (c) HRTEM image showing a few isolated knot-like defects, marked by yellow arrows. (d) Large-area HRTEM image showing continuous crystalline domains without amorphous regions at the domain boundaries.

For the large-mismatch 18.0% 2DPID–2DPBE vdW heterostructure, the two 2D polymers have distinct lattice parameters: a = b = 3.00 nm for 2DPID and a = b = 2.46 nm for 2DPBE. SAED shows a characteristic two sets of diffraction pattern with reflections from both constituent 2D polymers, corresponding to lattice periodicities of approximately 3.00 and 2.46 nm. Distinct Moiré reflections are also observed, providing direct evidence of long-range interference between the mismatched lattices. GIWAXS further shows two in-plane reflections at $Q_{xy} \approx 0.20$ Å^{-1} and $Q_{xy} \approx 0.25$ Å^{-1}, assigned to 2DPID and 2DPBE, respectively.

Large-area HRTEM images show distinct crystalline domains, with Moiré contrast observed in all of them, as highlighted in yellow. This confirms long-range interference between the two stacked 2D polymer lattices. Higher-magnification HRTEM images reveal wrinkling/warping of the 2D polymer layers, indicating out-of-plane deformation. These deformations provide a strain-relief pathway, allowing the stacked 2D polymer lattices to release interfacial stress through local curvature. In addition, long-range edge dislocations are observed, further indicating dissipation of the mechanical mismatch within the heterostructure. Together, the reciprocal-space signatures from SAED/GIWAXS and the real-space HRTEM features suggest that strain in the larger-mismatch 18.0% 2D polymer vdW heterostructure is relaxed through a combination of local lattice distortion, dislocation formation, and out-of-plane deformation.

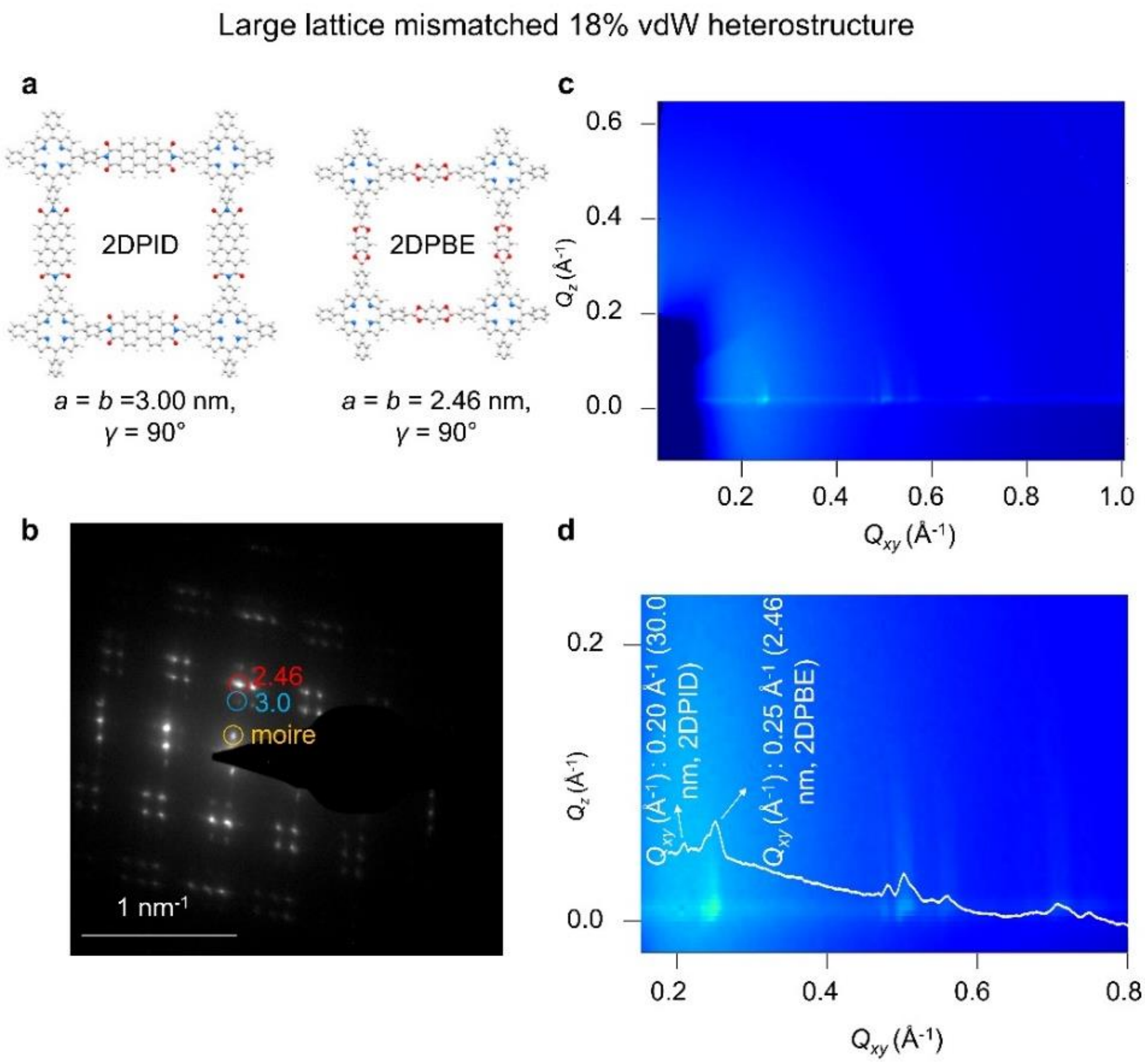

Figure R4 | SAED and GIWAXS characterization of the larger-mismatch 18.0% 2DPID–2DPBE 2D polymer vdW heterostructure. (a) Molecular structures of 2DPID (a = b = 3.00 nm, γ = 90°) and 2DPBE (a = b = 2.46 nm, γ = 90°). (b) SAED pattern showing double spots corresponding to 3.00 and 2.46 nm lattice periodicities, together with distinct Moiré reflections. (c) 2D GIWAXS pattern. (d) Enlarged in-plane GIWAXS region near $Q_z = 0$, showing reflections at $Q_{xy} \approx 0.20$ $Å^{-1}$ and 0.25 $Å^{-1}$, assigned to 2DPID and 2DPBE, respectively.

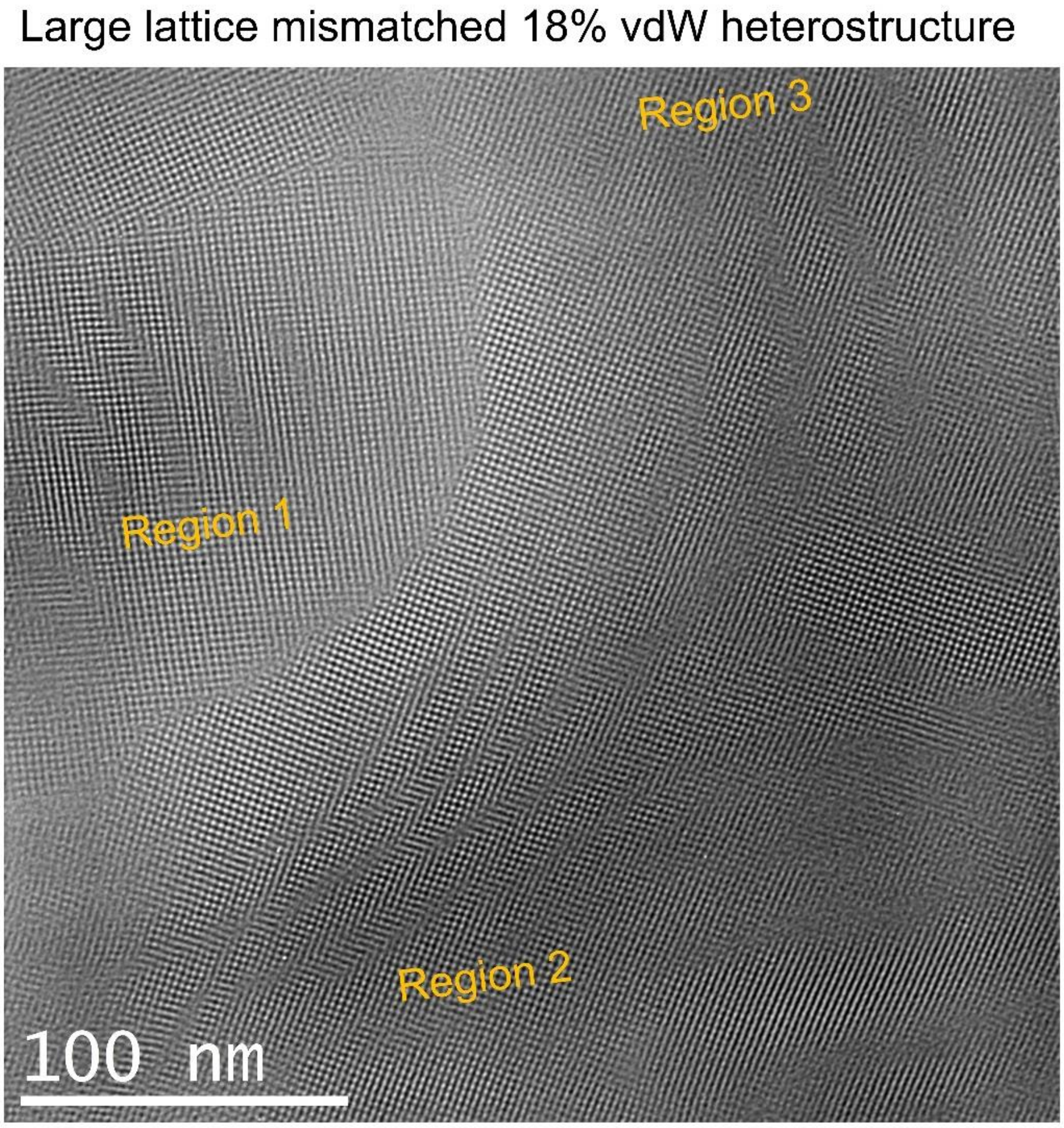

Figure R5 | HRTEM characterization of the larger-mismatch 18.0% 2DPID–2DPBE 2D polymer vdW heterostructure. Large-area HRTEM image showing different crystalline domains (regions 1–3), with Moiré contrast observed in all domains, confirming long-range interference between the two stacked 2D polymer lattices.

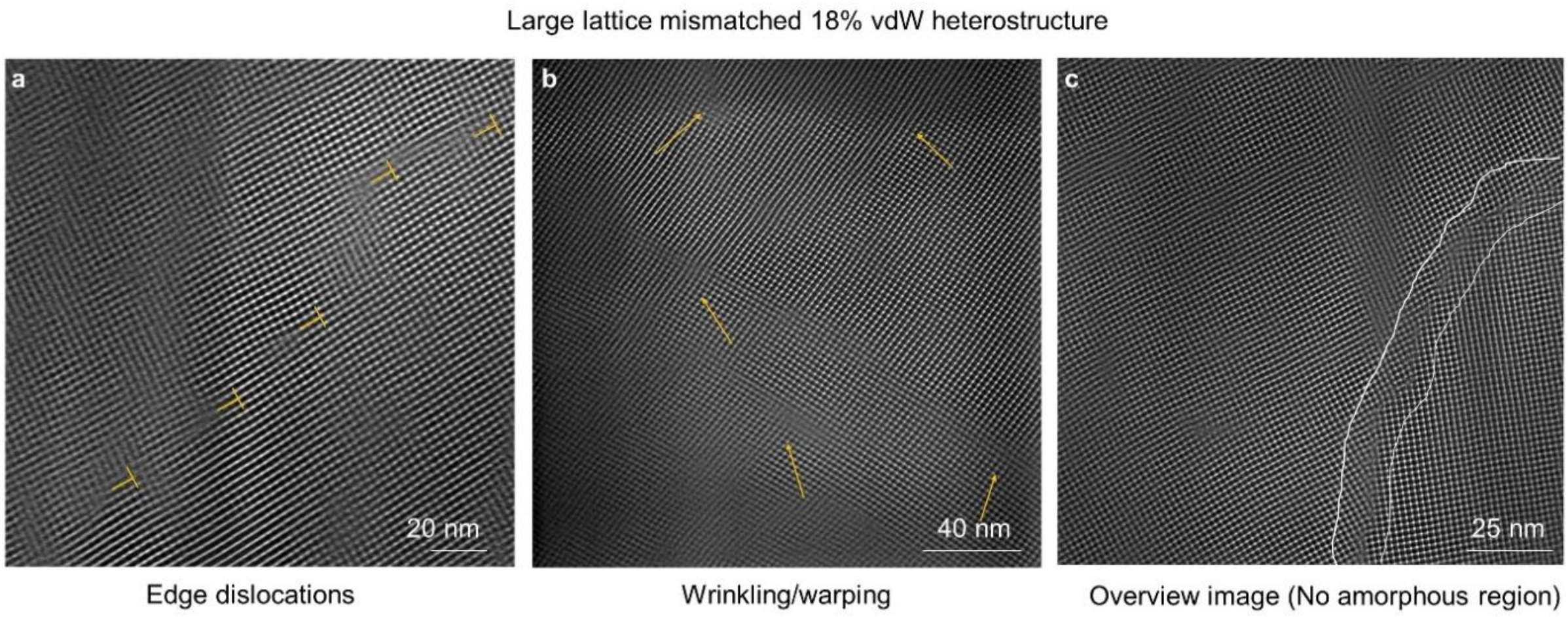

Figure R6 | HRTEM characterization of the larger-mismatch 18.0% 2DPID–2DPBE 2D polymer vdW heterostructure. (a) HRTEM image showing long-range edge dislocations. (b) HRTEM image showing wrinkling/warping of the 2D polymer layers. (c) Large-area HRTEM overview image showing continuous crystalline domains without amorphous regions at the domain boundaries.

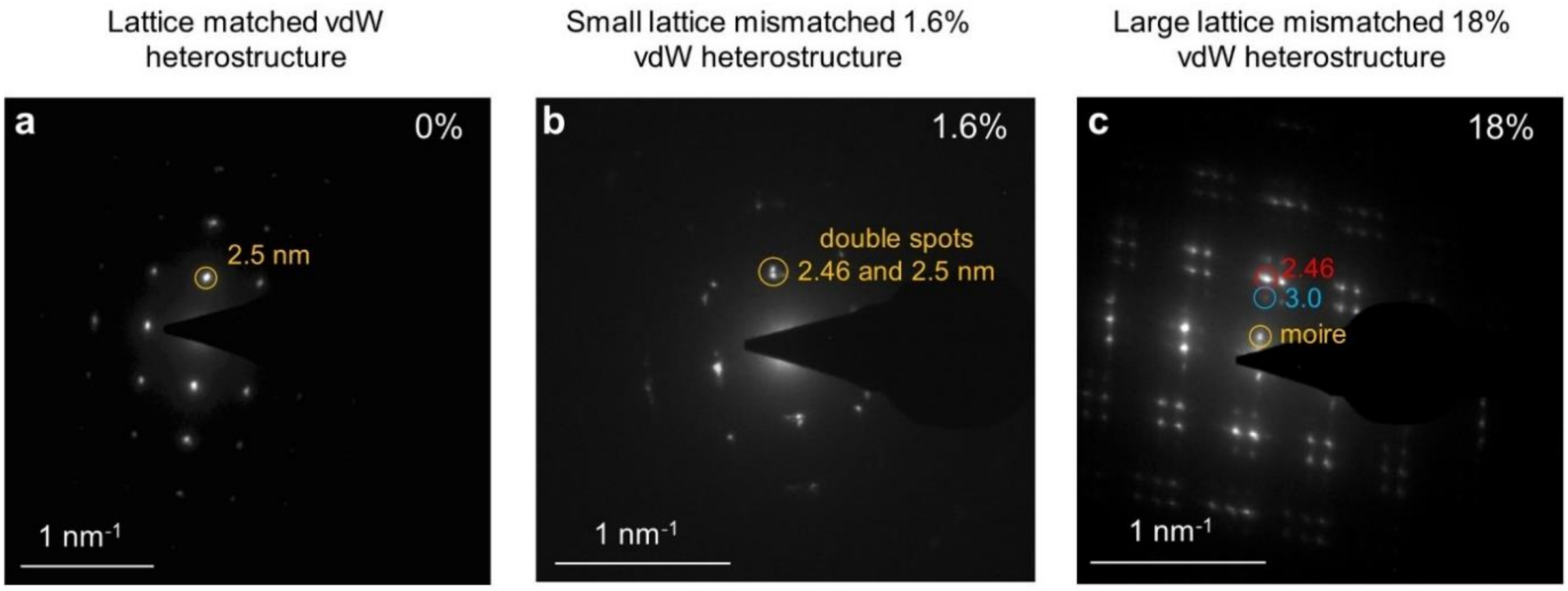

Figure R7 | SAED comparison of lattice-matched and lattice-mismatched 2D polymer vdW heterostructures. (a) Lattice-matched 0% vdW heterostructure showing a single set of diffraction spots corresponding to 2.50 nm periodicity. (b) Small-mismatch 1.6% vdW heterostructure showing closely spaced double spots corresponding to 2.46 and 2.50 nm periodicities. (c) Larger-mismatch 18.0% vdW heterostructure showing clearly separated diffraction spots corresponding to 2.46 and 3.00 nm periodicities, together with distinct Moiré reflections.

Action: Figures R1–R7 have been incorporated into the revised Supplementary Information as Supplementary Figs. 8–14. On page 10 of the revised main text, the following discussion has been added. A detailed discussion is also provided in the Supplementary Information, see Supplementary Figs. 8–14.

Text added to the main text:

Furthermore, for the small-mismatch 1.6% **2DPBI–2DPBE** vdW heterostructure, GIWAXS resolves two closely spaced in-plane reflection series, with (100) peaks at Q_{xy} = 0.251 and 0.255 $Å^{-1}$, corresponding to lattice parameters of a = b = 2.50 nm for **2DPBI** and a = b = 2.46 nm for **2DPBE**, respectively. SAED further shows closely spaced superimposed diffraction lattices corresponding to both lattice periodicities, while HRTEM confirms continuous ordered crystalline domains without pronounced warping of the 2D polymer layers. For the large-mismatch 18.0% **2DPID–2DPBE** vdW heterostructure, GIWAXS shows two clearly separated in-plane reflections at $Q_{xy} \approx 0.20$ and 0.25 $Å^{-1}$, assigned to **2DPID** and **2DPBE**, respectively. In addition, SAED shows two sets of diffraction spots from the constituent 2D polymer lattices, corresponding to lattice parameters of a = b = 3.00 nm for 2DPID and a = b = 2.46 nm for 2DPBE, along with distinct Moiré reflections. Large-area HRTEM images show different crystalline domains with Moiré contrast, while higher-magnification HRTEM reveals wrinkling/warping and long-range edge dislocations. Together, these reciprocal-space and real-space observations suggest that strain relaxation in the large-mismatch heterostructure occurs through a combination of local lattice distortion, dislocation formation, and out-of-plane deformation (Supplementary Figs. 30–40).

2. *The surfactant monolayer plays a central role in guiding the interfacial assembly and epitaxial growth of the 2D polymer films. However, the rationale behind the use of this specifical surfactant is not fully discussed. In addition to the anionic surfactant used in this work, is it possible to use cationic surfactants? How will their charge properties affect the interfacial assembly of 2D polymer films?*

Response: We sincerely appreciate the reviewer's valuable and insightful comment. The sequential assembly approach relies on two key design criteria: (i) charge complementarity between the surfactant and the molecular building block, and (ii) the ability of the surfactant monolayer to form an ordered crystalline surface that guides monomer pre-assembly.

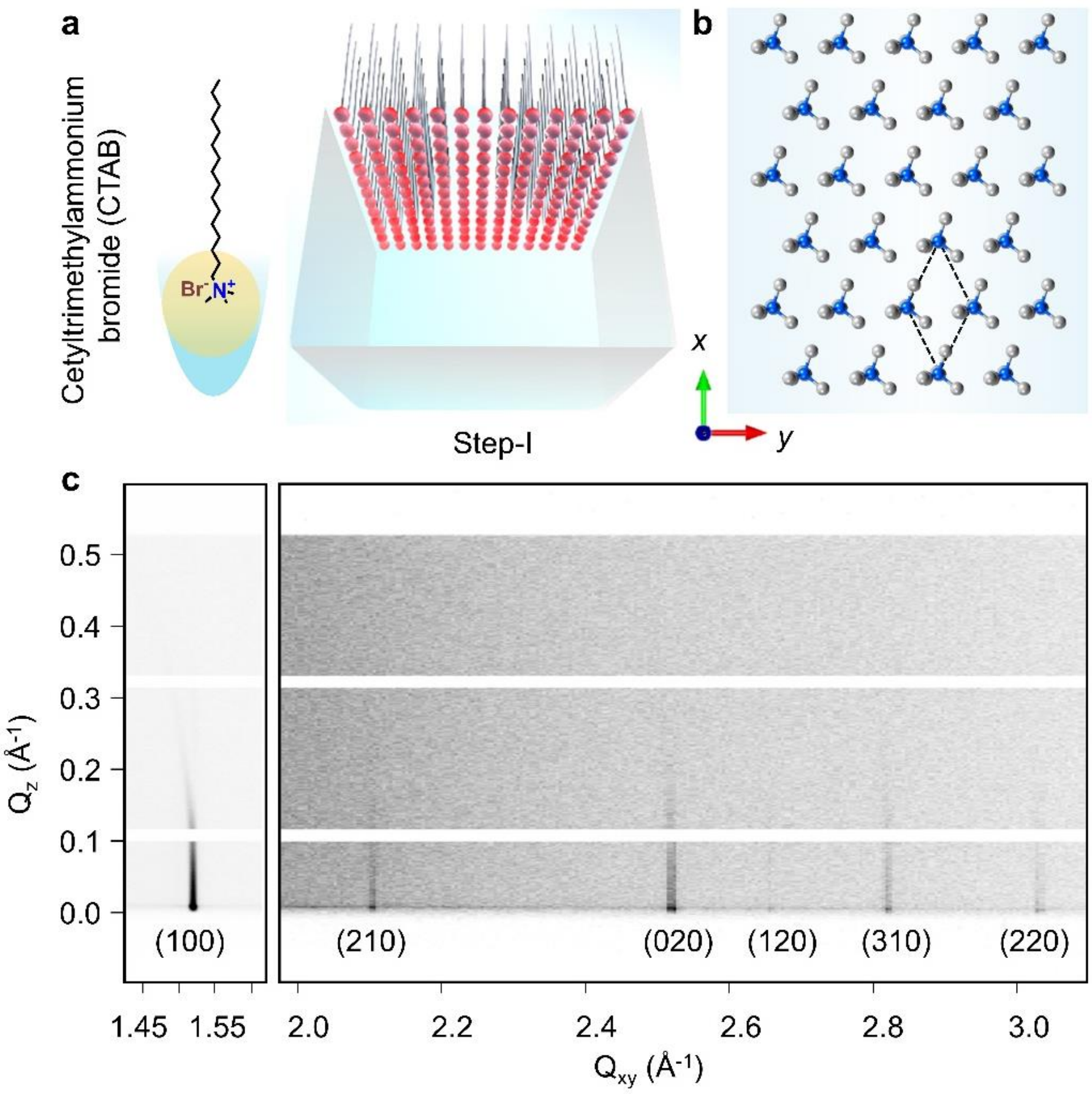

Figure R8 | GIXD characterization of the CTAB surfactant monolayer. (a) Molecular structure and schematic of the cationic CTAB monolayer on the water surface. (b) Molecular structure of the CTAB monolayer. (c) In-situ GIXD pattern showing sharp diffraction peaks from the crystalline CTAB monolayer.

The SOS surfactant contains an anionic sulfate headgroup, which promotes electrostatic adsorption of protonated M1 under acidic conditions. In addition, the crystalline SOS monolayer serves as an ordered template for the epitaxial pre-organization of M1. To further examine the role of surfactant charge, we performed additional GIWAXS experiments using the cationic surfactant CTAB. Under basic conditions, M3, 5,10,15,20-tetrakis(4-carboxyphenyl)porphyrin, carries negatively charged

carboxylate groups and therefore interacts electrostatically with the cationic CTAB monolayer. CTAB forms a crystalline monolayer on the water surface with a = b = 4.49 Å and γ = 67.52°. Upon M3 addition, an ordered molecular assembly is observed with a = 17.53 Å, b = 17.49 Å, and γ = 89.40°. The epitaxial alignment corresponds to point-on-line epitaxy, in which M3 lattice points register along the [1,0] lattice lines of the CTAB monolayer. Upon addition of M4 (1,2,4,5-benzenetetramine), M4 reacts with the pre-assembled M3 layer to form 2DPBI. The resulting diffraction peaks correspond to a square 2D polymer lattice with a = b = 25.1 Å and γ = 90.00°. The epitaxial relationship then evolves into line-on-line epitaxy, in which extended lattice lines of 2DPBI along the [3,–6] direction coincide with the [1,1] crystallographic lines of the CTAB surfactant lattice. This charge-complementarity principle is also supported by our previous model reaction, in which the cationic surfactant CTAB was used to facilitate the adsorption of a negatively charged porphyrin reagent on the water surface, enabling the site-selective imidazole-bond-forming reaction (*Nat. Commun. 14, 8313, 2023*). These results clearly demonstrate that the important factor for the monomer assembly is the complementary charge on the surfactant compared to the M1 molecule and both anionic and cationic surfactants can be used for the successful synthesis of 2D polymers.

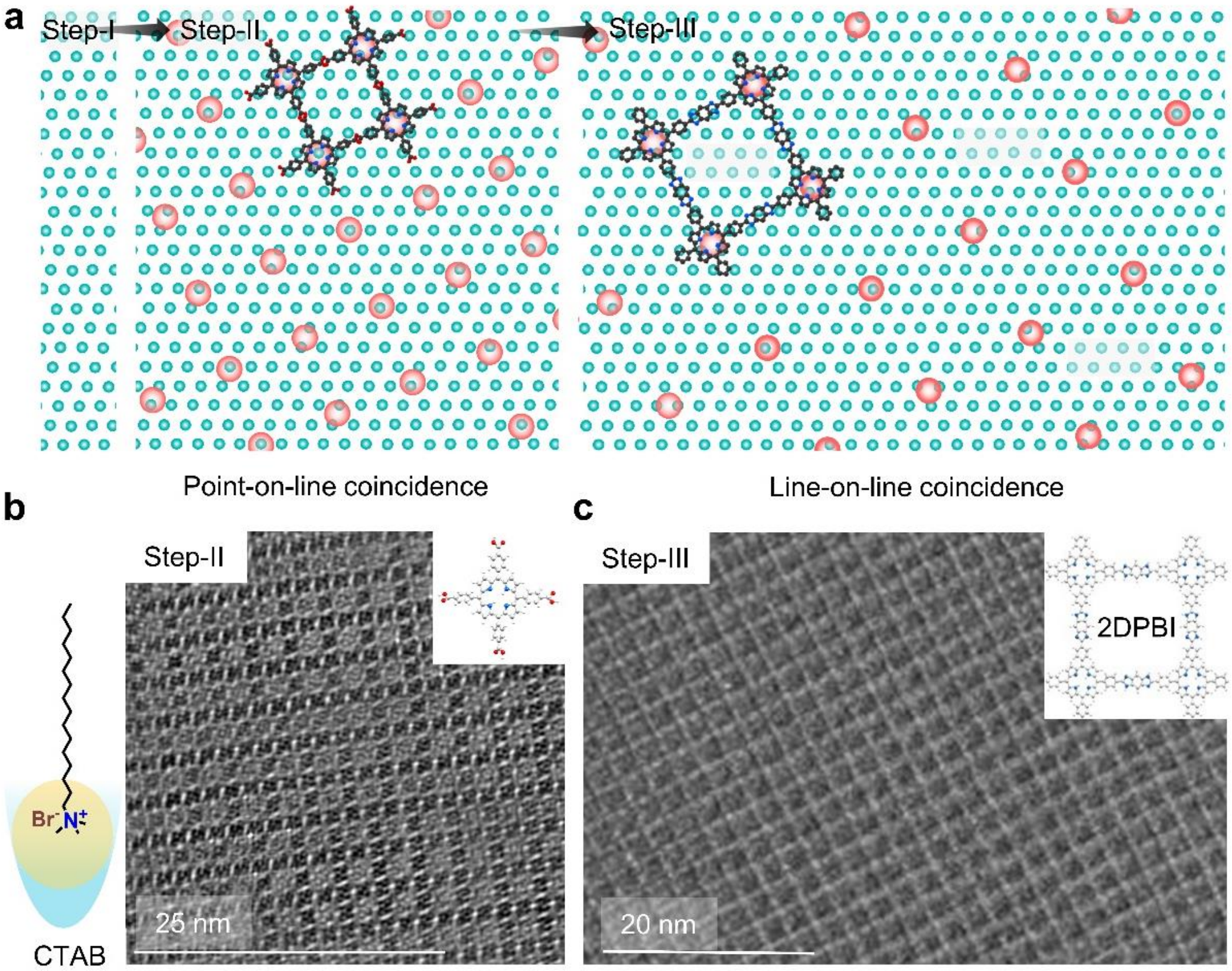

Figure R9 | Cationic surfactant CTAB-guided epitaxial pre-assembly of monomers and 2DPBI formation. (a) Schematic diagram of the CTAB-guided epitaxial growth process, showing the transition from point-on-line coincidence during M3 pre-assembly to line-on-line coincidence after 2DPBI formation. (b) HRTEM image of the pre-assembled CTAB–M3 layer (Step-II). (c) HRTEM image of the resulting 2DPBI film (Step-III).

More broadly, the lattice structure of the surfactant monolayer is a key parameter for 2D polymer growth, as it affects both monomer adsorption and azimuthal pre-organization on the water surface. As shown for both SOS and CTAB, charged surfactant monolayers can guide monomer ordering through epitaxial alignment. However, such ordering requires a suitable epitaxial match between the surfactant lattice and the size/symmetry of the adsorbed monomers. We therefore consider surfactant-lattice design to be an important direction for extending this strategy to other monomer systems. [redacted]

[REDACTED]

[redacted]

Action: Figure R9 have been incorporated into the revised Supplementary Information as Supplementary Fig. 21. We have added the corresponding discussion to the Supplementary Information, on the role of surfactant charge, CTAB-guided M3 pre-assembly, and subsequent 2DPBI formation see Supplementary Figs. 20-21.

.**3.** *The HRTEM images in Figure 2b and Supplementary Figure 18 show grain boundaries and discontinuities within the 2D polymer film. Will such structural defects affect interfacial electronic coupling and out-of-plane electrical transport? Moreover, the observation of such grain boundaries may indicate disordered adsorption of monomers during Sept-II. Could this structural nonuniformity be avoided by optimizing the synthesis conditions?*

Response: We appreciate the reviewer's valuable and insightful comment. To assess the influence of grain boundaries on out-of-plane transport, we performed nanoscale conductive AFM (c-AFM) I–V measurements on the lattice-matched 2DPI–2DPBI vdW heterostructure. The measurements were collected from five different samples, with five spatially distinct points measured on each sample. The effects of local defects and grain boundaries are reflected in a one-order-of-magnitude variation in the rectification ratio; however, all values at ±1 V remain within the range of 10^4–10^5. A similar one-order-of-magnitude variation is also observed in the vertical micro-device measurements at the same bias window of ±1 V, where the rectification ratios likewise remain within the range of 10^4–10^5. This consistency between nanoscale c-AFM and vertical micro-device measurements indicates that grain boundaries and local discontinuities may influence the local current transport response, but do not necessarily dominate the overall out-of-plane diode behavior of the vertical 2DPI–2DPBI vdW heterostructure.

Consistently, a recent study on 2DPANI showed that transport in the out-of-plane direction measured by c-AFM is less affected by structural defects, whereas transport in the in-plane direction is more sensitive to structural defects, energetic disorder, and trap states (*Nature 638, 411–417, 2025*). This behavior is mainly associated with the distinct device length scales: in the in-plane direction, devices are typically fabricated on the micrometre scale, whereas in the out-of-plane direction, charge transport occurs across the much thinner (i.e *π-π* direction), typically only tens of nanometres. Therefore, in the present vertical vdW heterostructure geometry, grain boundaries have only a limited influence on the measured out-of-plane transport. In contrast, such defects would be more critical in lateral heterostructure devices, where charge transport proceeds across extended in-plane grain boundaries.

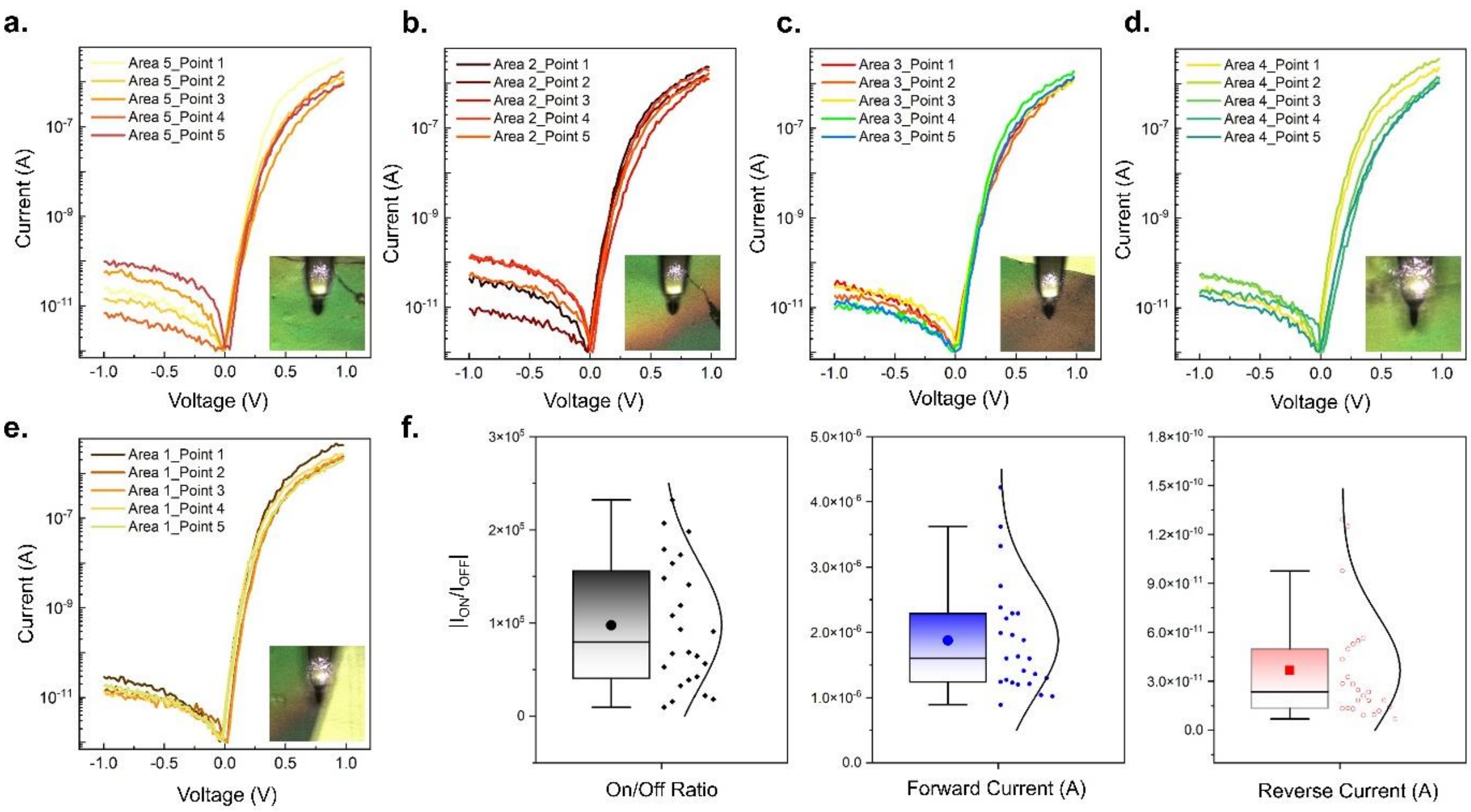

Figure R11 | Statistical c-AFM I–V characterization of 2DPI–2DPBI vdW heterostructures. (a–e) c-AFM I–V curves measured from five independent 2DPI–2DPBI vdW heterostructure samples, with five spatially distinct points collected from each sample. Insets show optical images of the cantilever approaching the sample surface. (f) Statistical analysis of the extracted transport parameters, including rectification ratio, forward current, and reverse current.

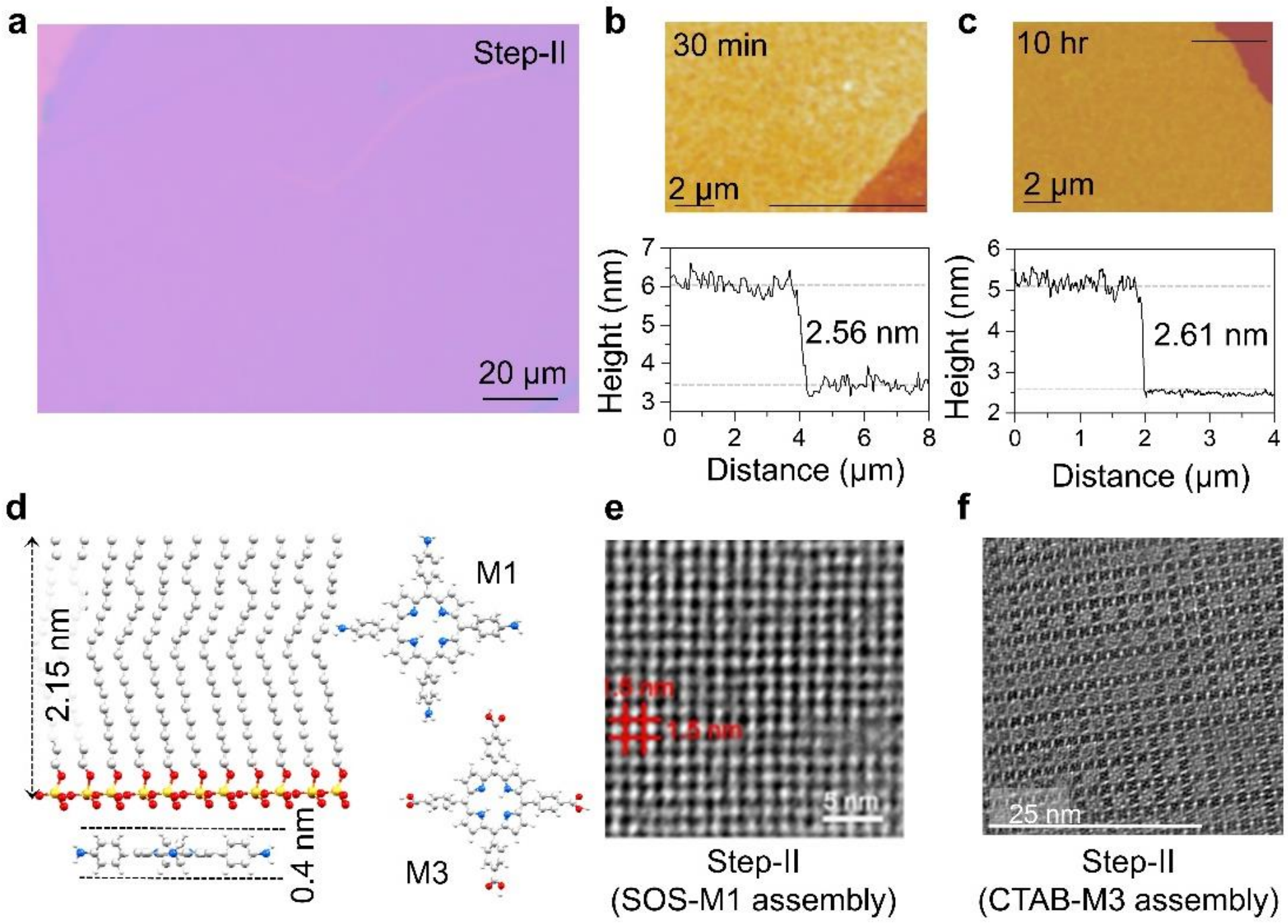

Figure R12 | Step-II monomer pre-assembly underneath the charged surfactant monolayers. (a) Optical microscopy image of the SOS–M1 assembly formed in Step-II. (b,c) AFM images and height profiles of the SOS–M1 assembly after M1 adsorption for 30 min and 10 h, showing thicknesses of 2.56 and 2.61 nm, respectively. (d) Schematic thickness comparison of the SOS monolayer and M1/M3 molecules. (e) HRTEM image of the SOS–M1 assembly in Step-II, adapted from ref. 45 (Nat. Chem. 11, 994–1000, 2019). (f) HRTEM image of the CTAB–M3 assembly in Step-II.

To examine the Step-II pre-assembly process, we performed AFM measurements on the SOS–M1 assembly transferred onto SiO_2/Si by the Langmuir–Schaefer method. After 30 min of M1 adsorption, the transferred SOS–M1 assembly shows a thickness of 2.56 nm. Extending Step-II to 10 h gives a similar thickness of 2.61 nm. These values closely match the combined thickness of the SOS monolayer (~2.15 nm) and the thickness of M1 molecules (~0.4 nm), suggesting that Step-II mainly involves monolayer/few-layer M1 pre-assembly beneath the SOS surfactant monolayer. HRTEM images further show crystalline SOS–M1 and CTAB–M3 assemblies, supporting ordered monomer pre-organization in Step-II. This behavior is similar to the previously reported model reaction for on-water surface synthesis (*Nat Commun 14, 8313, 2023*). For the model reaction, porphyrin adsorption beneath the SOS monolayer screened the surfactant charge within ~30 min, establishing a pre-assembled porphyrin layer. After the addition of the second reactant the reaction kinetics became faster, and bond formation regenerated a dynamic charged interface that promoted further interfacial growth. Similarly, during 2D

polymerization, Step-II mainly corresponds to M1 pre-assembly, while the more dynamic growth process starts after M2 addition in Step-III.

Regarding the possible origin of grain boundaries, on water surface synthesis is inherently delicate. The crystalline surfactant monolayer is first formed on the water surface, and subsequent monomer injection/addition can disturb the order of the monolayer and interface. In the 2D polymerization process, such a disturbances can occur at two points: first during monomer pre-assembly after M1 addition (Step-II), and then during polymerization after M2 addition (Step-III). Together with monomer diffusion and interfacial changes, these perturbations can locally disturb the SOS surfactant structure (e.g. cracks, folds), generating a few crystalline domains in the SOS monolayer, which ultimately influence the size of the resulting 2D polymer film's crystalline domains..

Action: Figures R11 and R12 have been incorporated into the revised Supplementary Information as Supplementary Figs. 55 and 20, respectively.

4. *The manuscript proposes a transition from point-on-line to line-on-line epitaxy during sequential assembly. However, the driving force for this evolution remains insufficiently clarified. Moreover, is this mechanism universal for different monomers, or does it rely on specific lattice systems?*

Response: We sincerely appreciate the reviewer's valuable and insightful comment. To clarify the driving force for the transition from point-on-line to line-on-line epitaxy, we analyzed three representative square-lattice 2D polymer systems with different lattice parameters, using both anionic SOS and cationic CTAB surfactants.

Case-I: 2DPI formation with anionic SOS surfactant.

For 2DPI (a = b = 2.50 nm, γ = 90°), the Step-II epitaxial alignment corresponds to point-on-line epitaxy, in which M1 lattice points register along the (1,0) lattice lines of the SOS surfactant lattice. After M2 addition in Step-III, the lattice *expands* from the pre-assembled M1 structure (a = 13.22 Å, b = 15.29 Å, γ = 85.24°) to the square 2DPI lattice (a = b = 25.1 Å, γ = 90°). This lattice expansion changes the epitaxial relationship to line-on-line epitaxy (defined as coincidence of nonprimitive reciprocal lattice vectors of the adsorbate and the substrate), where the 2DPI [6,3] lattice lines coincide with the SOS [1,1] crystallographic lines.

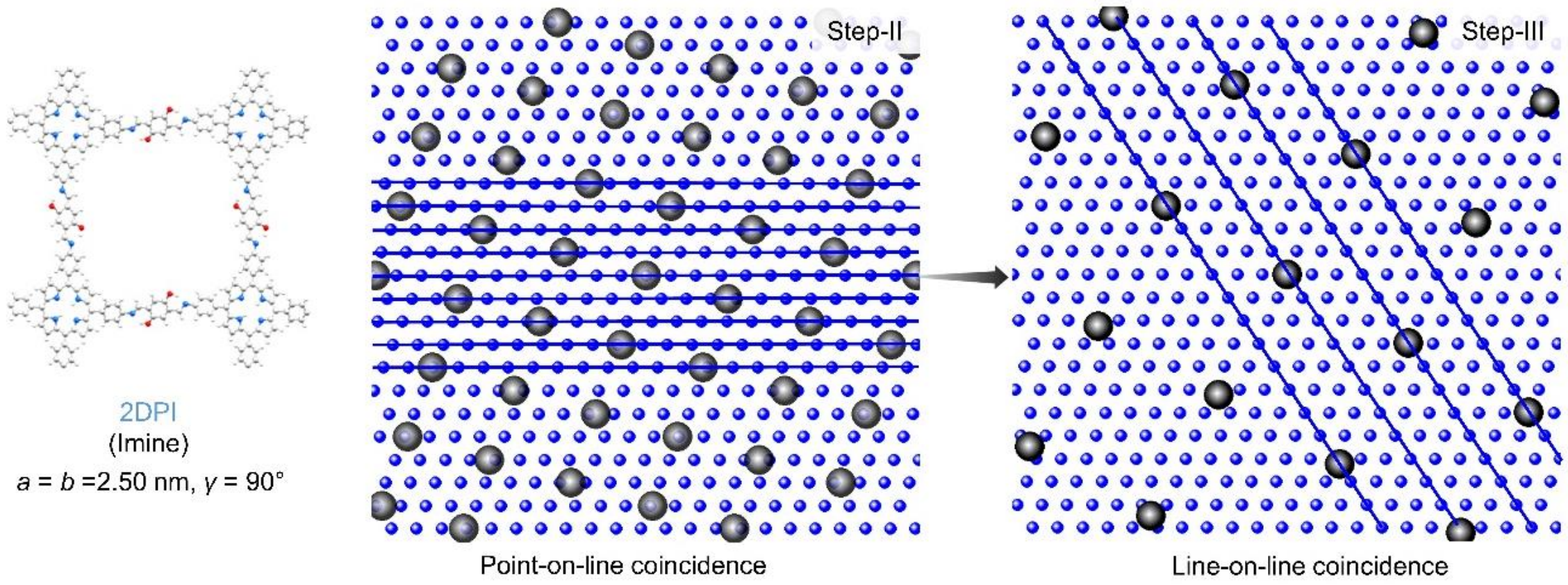

Figure 13 | Epitaxial transition in on-water surface synthesis of 2DPI. Schematic illustration of point-on-line coincidence during M1 pre-assembly in Step-II and line-on-line coincidence during 2DPI formation in Step-III.

Case-II: 2DPID formation with anionic SOS surfactant.

For 2DPID (a = b = 3.00 nm, γ = 90°), the Step-II epitaxial alignment also corresponds to point-on-line epitaxy, in which M1 lattice points register along the (1,0) lattice lines of the SOS surfactant lattice. After the addition of M7 (perylene-3,4,9,10-tetracarboxylic dianhydride) in Step-III, the lattice expands from the pre-assembled M1 structure (a = 13.22 Å, b = 15.29 Å, γ = 85.24°) to the square 2DPID lattice (a = b ≈ 30.0 Å, γ = 90°). The epitaxial relationship then evolves into a line-on-line epitaxy, where extended 2DPID [-7,2] lattice lines coincide with the SOS [1,0] crystallographic lines.

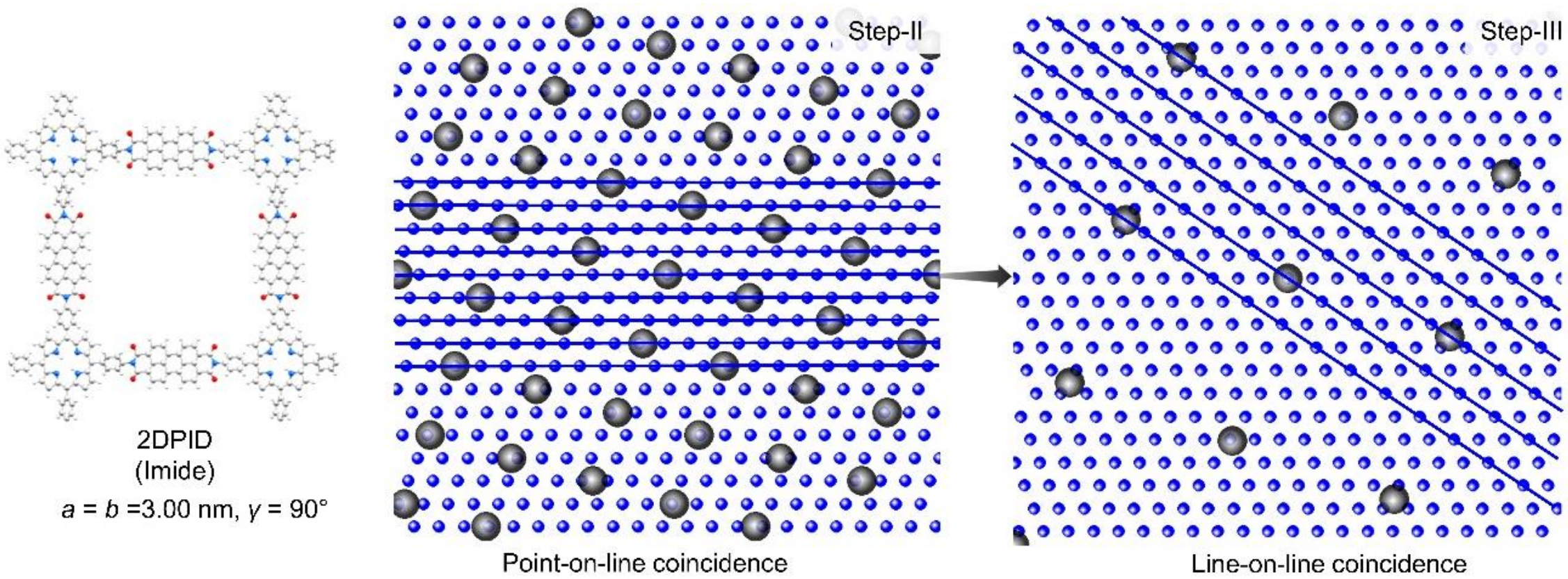

Figure 14 | Epitaxial transition in on-water surface synthesis of 2DPID. Schematic illustration of point-on-line coincidence during M3 pre-assembly in Step-II and line-on-line coincidence during 2DPBI formation in Step-III.

Case-III: 2DPBI formation with cationic CTAB surfactant.

For 2DPBI (a = b = 2.50 nm, γ = 90°), the Step-II epitaxial alignment corresponds to point-on-line epitaxy, in which M3 lattice points register along the (0,1) lattice lines of the CTAB surfactant lattice. After M4 addition in Step-III, the lattice expands from the pre-assembled M3 structure (a = 17.53 Å,

b = 17.49 Å, γ = 89.40°) to the square 2DPBI lattice (a = b = 25.1 Å, γ = 90.00°). The epitaxial relationship then evolves into line-on-line epitaxy, where extended 2DPBI [3,–6] lattice lines coincide with the CTAB [1,1] crystallographic lines.

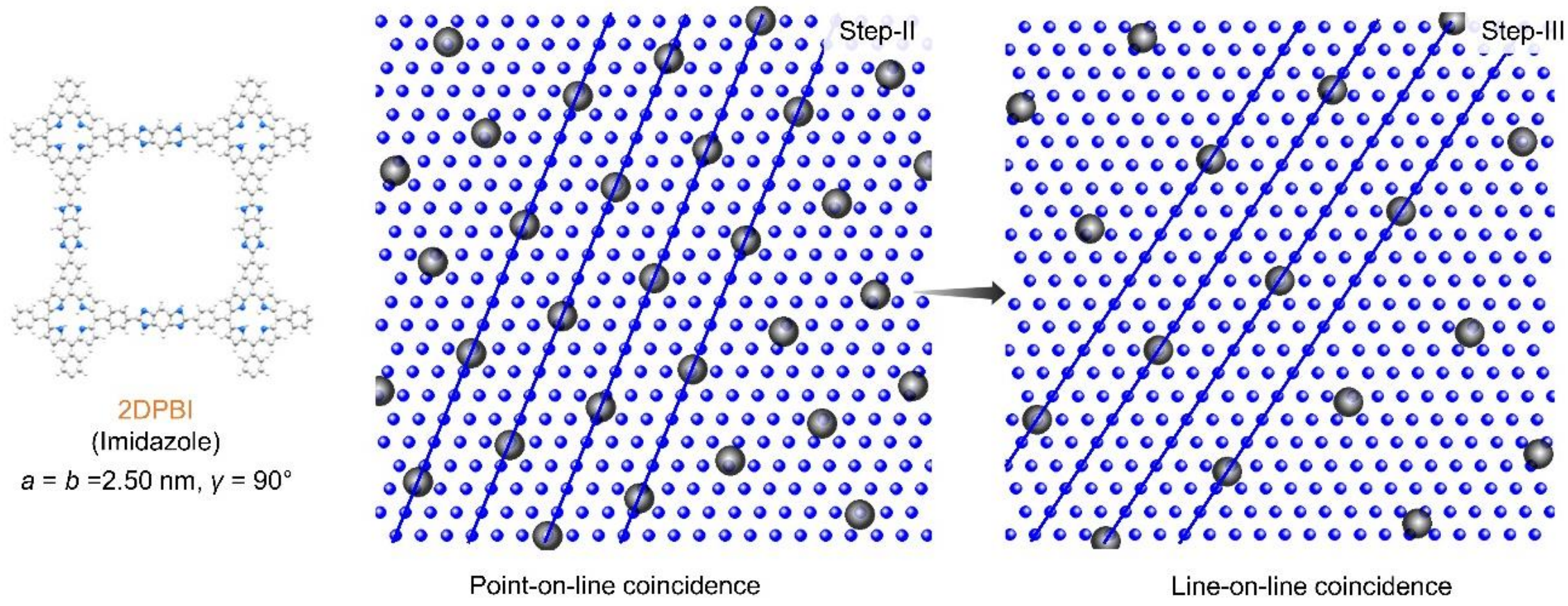

Figure 15 | Epitaxial transition in on-water surface synthesis of 2DPBI Schematic illustration of point-on-line coincidence during M1 pre-assembly in Step-II and line-on-line coincidence during 2DPID formation in Step-III.

Epitaxial growth is fundamentally driven by the minimization of total system energy, i.e. it depends on the balance between interfacial energy and the elastic strain required to match the interacting lattices. In our system, we would characterize the driving force for the transition from point-on-line to line-on-line epitaxy to be the dramatic lattice expansion that occurs during covalent polymerization (Step-III). During Step-II, the discrete monomer assemblies maintain a point-on-line registry with the charged crystalline surfactant. However, as the second monomer is added and covalent bonds form, the resulting 2D polymer lattice expands, and the much larger lattice will establish an epitaxial relationship that again minimizes energy (or none if none is available) – in our cases adopting line-on-line epitaxy in order to minimize the overall energy for the newly expanded lattice.

Since line-on-line epitaxy is the most general (every point-on-line lattice is thus also line-on-line), it therefore also represents the lowest degree of epitaxial matching, and it is possibly more common in systems where the elastic energy to distort the involved unit cells to higher degrees of epitaxy would exceed any potential gain in interface energy. However, because the epitaxial match in our systems involves soft crystals growing on soft templates, establishing a strict, quantitative energetic hierarchy would require comprehensive energy calculations; however, the qualitative driving force is clearly the strain-induced relaxation of the expanded polymer.

Regarding universality, we would state that this mechanism cannot be considered a general rule for all monomers or lattice systems. The preferred epitaxial state depends sensitively on the exact balance of intra- and interlayer interactions—specifically, the elastic moduli of the two lattices and their interlayer interaction potentials. If, for instance, a different 2D polymer had a chemical structure and dimensions

that by chance allowed a higher degree of lattice match (e.g., commensurate epitaxy) with minimal distortion, yielding a high gain in interfacial energy, one would likely observe commensurate epitaxy. While we consistently observe the transition to line-on-line epitaxy for all square-lattice 2D polymers examined in this study, the exact epitaxial evolution in other systems would thus remain highly dependent on monomer geometry, charge complementarity, surfactant lattice parameters, and the final 2D polymer structure.

Action: Figures R13–R15 have been incorporated into the revised Supplementary Information as Supplementary Figs. 25–27. The corresponding discussion on the transition from point-on-line to line-on-line epitaxy for the 2DPI, 2DPID, and 2DPBI square-lattice systems has been added to the Supplementary Information. Detailed discussion is provided in Supplementary Figs. 25–27.

5. *For large lattice-mismatched 2D polymer vdW heterostructures (16.6% and 18.0% mismatch), it is unclear how interfacial strain and structure are accommodated. Did the authors observe any moire fringes for these cases given their significant lattice mismatch degrees?*

Response: We appreciate the reviewer's valuable comment. Yes, Moiré features are observed in the large-mismatch 2D polymer vdW heterostructures, as shown in Figures. R4–R7. For these systems, SAED shows superimposed diffraction from the two stacked 2D polymer lattices. For the 18.0% 2DPID–2DPBE vdW heterostructure, SAED shows characteristic two sets of diffraction spots from both constituent lattices, together with distinct Moiré reflections. Large-area HRTEM images show different crystalline domains, with Moiré contrast observed within the domains. Higher-magnification HRTEM further reveals wrinkling/warping of the 2D polymer layers, as well as long-range edge-dislocation features. These observations indicate that the large lattice mismatch is relaxed through Moiré formation, local lattice distortion, and out-of-plane deformation.

6. *In layer-on-layer heterostructures, diffraction contributions from individual layers may overlap. The authors should clarify how overlapping GIWAXS and GIXD signals are assigned to each layer, particularly for those heterostructures with large lattice-mismatch degrees.*

Response: We appreciate the reviewer's valuable comment. We assigned the GIWAXS/GIXD signals by first identifying the individual diffraction peaks of each parent 2D polymer. The first-order in-plane reflection, assigned to the (100)/(010) family, is the most characteristic peak of the square 2D lattice and directly defines the lattice parameter through $a = 2\pi/Q(100)$.

For the lattice-matched 2DPI–2DPBI vdW heterostructure (Figure R1), both 2D polymers have the same square lattice parameter ($a = b = 2.50$ nm, $\gamma = 90°$). Therefore, the (100)/(010) and higher-order reflections of both layers appear at the exact same Q_{xy} position and should therefore overlap. This overlap is assigned to coincident reflections from both stacked 2D polymer layers. For the small-

mismatch 1.6% 2DPI–2DPBE vdW heterostructure (Figure R2), the first-order reflections are clearly split into two resolvable peaks. GIWAXS shows two (100) peaks at Qxy = 0.251 Å^{-1} and 0.255 Å^{-1}, corresponding to a = b = 2.50 nm for 2DPI and a = b = 2.46 nm for 2DPBE, respectively. For example, in the 18.0% 2DPID–2DPBE vdW heterostructure, the (100) reflections from 2DPID (a = b = 3.00 nm) and 2DPBE (a = b = 2.46 nm) appear at distinct Q_{xy} positions, allowing direct assignment of the individual 2D polymer contributions.

7. *Thickness control over the developed heterostructures from 4.6 to 30 nm is discussed. However, there are no experimental results to support this discussion.*

Response: We appreciate the reviewer's valuable comment. We have added experimental AFM data to support the thickness control of the 2D polymer vdW heterostructures. The heterostructure thickness can be tuned from 4.6 to 30.0 nm by varying the reaction time from 6 to 60 h.

Action: Figure R16 has been incorporated into the revised Supplementary Information as Supplementary Fig. 34.

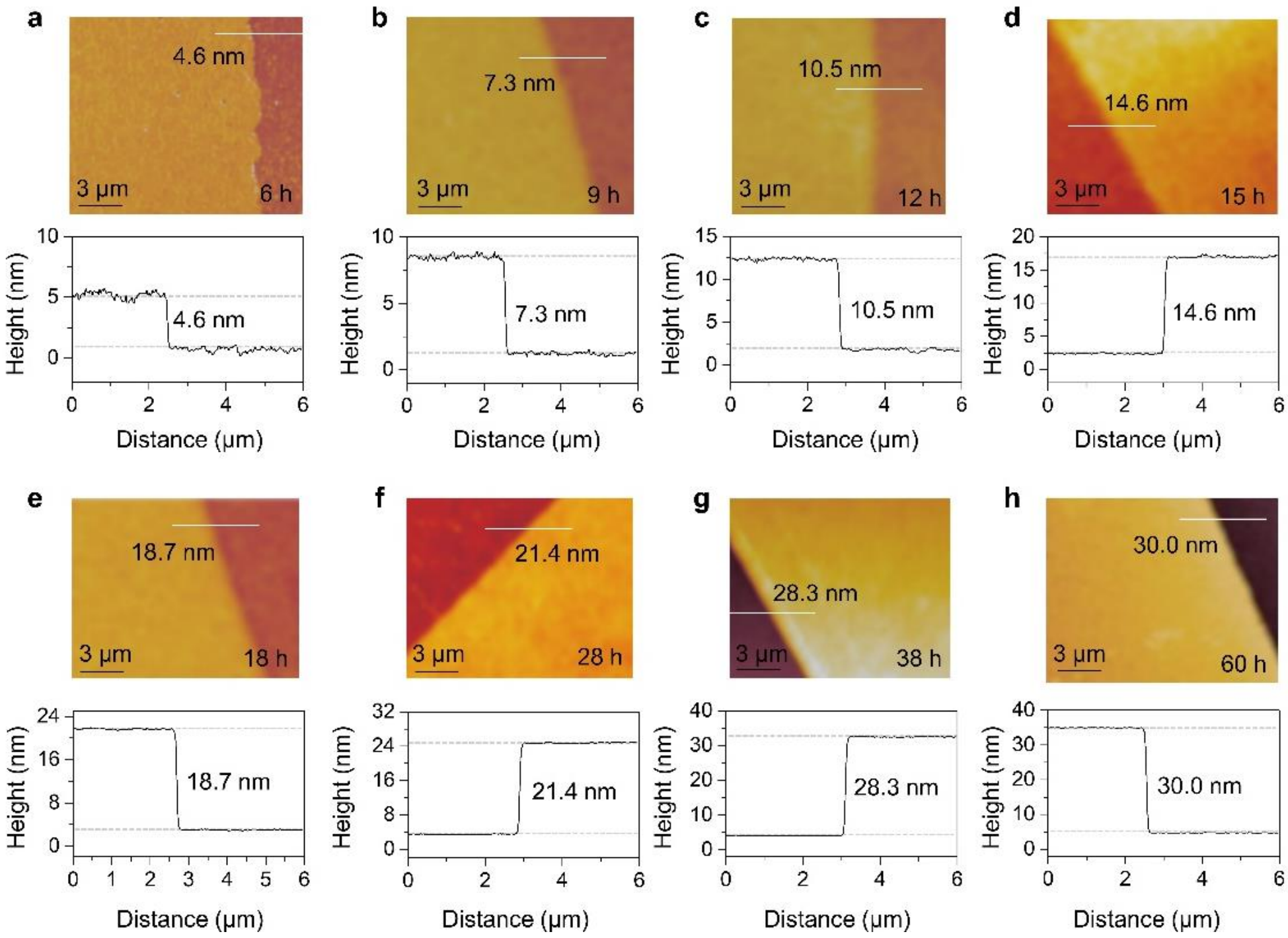

Figure R16 | Thickness control of 2D polymer vdW heterostructures. AFM images and corresponding height profiles showing the reaction-time-dependent thickness increase of the 2D polymer vdW heterostructures.

8. *How can the authors experimentally confirm that the first layer is fully dense and continuous? Could the monomers for the second layer diffuse through the relatively large pores of the first layer and polymerize on top of the first layer, rather than exclusively beneath it? Could potential defects in the*

first layer cause the second layer to nucleate and grow on the same plane, rather than forming a layer-on-layer heterostructure?

Response: We sincerely appreciate the reviewer's important comment. To examine the continuity of the first 2DPI layer, we performed Brewster angle microscopy (BAM) directly on the water surface. A blank water surface was used as the control. At the Brewster angle of water (~53°), p-polarized light from a clean water surface gives nearly zero reflection, resulting in a black background. In Step-III, the 2DPI film shows clear grey/bright contrast against the black water background, confirming continuous film formation on the water surface. We further transferred the 2DPI film onto SiO_2/Si and copper TEM grids. Optical microscopy shows a uniform film with clearly visible, sharp edges over large areas. The 2DPI film can also suspend over large grid openings with edge lengths of ~60 μm, indicating good mechanical stability and large-area continuity. Some film edges can break during transfer, which we attribute to transfer-induced mechanical damage. HRTEM shows only a few isolated knot-like defects, as discussed above (Figures R3–R6), rather than extended holes or large discontinuities. The lateral size of these knot-like defects is within ~1.5 nm, corresponding to the molecular scale. Therefore, these small and isolated defects are not expected to allow effective monomer diffusion through the first 2D polymer layer and are not sufficient to induce dominant same-plane growth of the second 2D polymer.

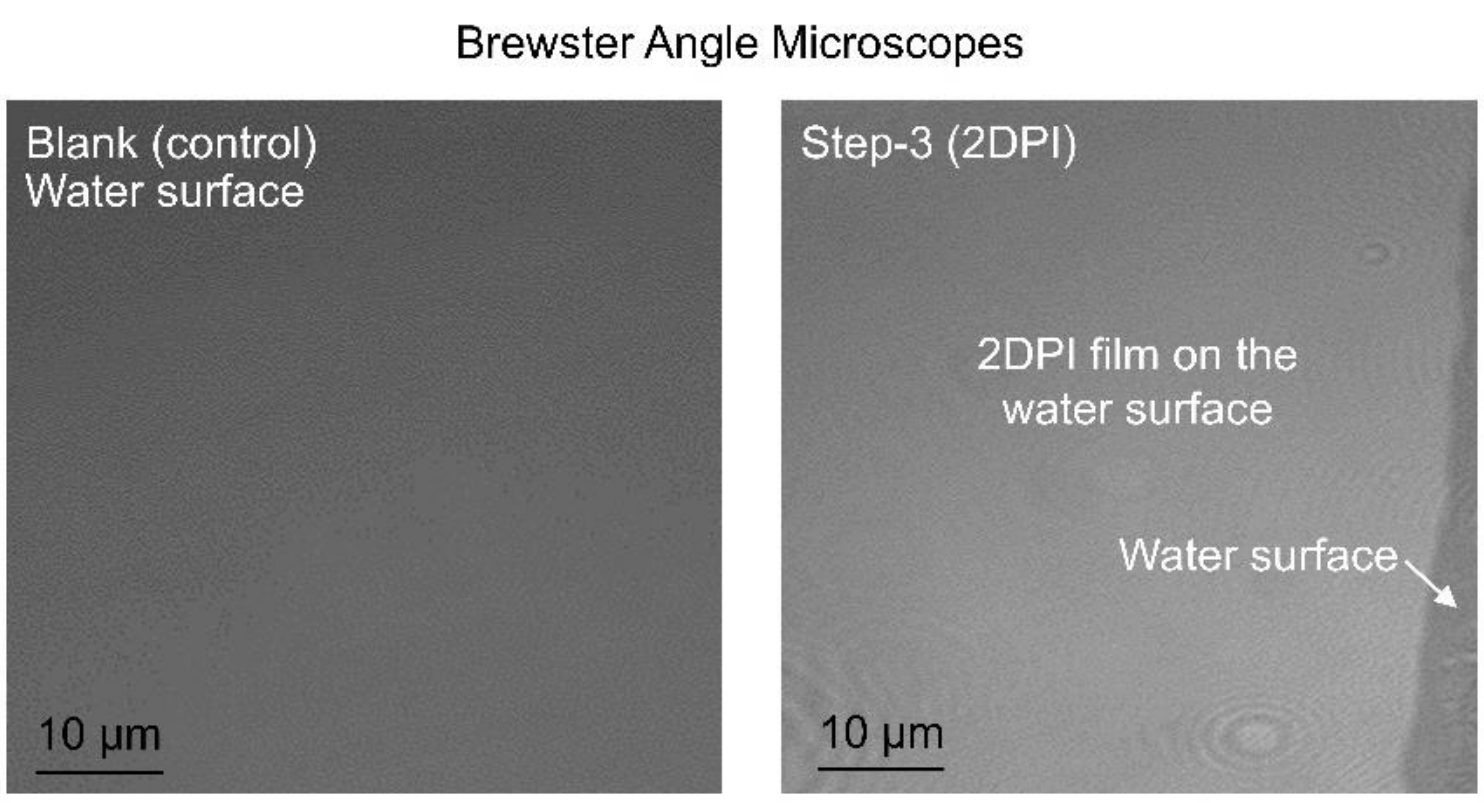

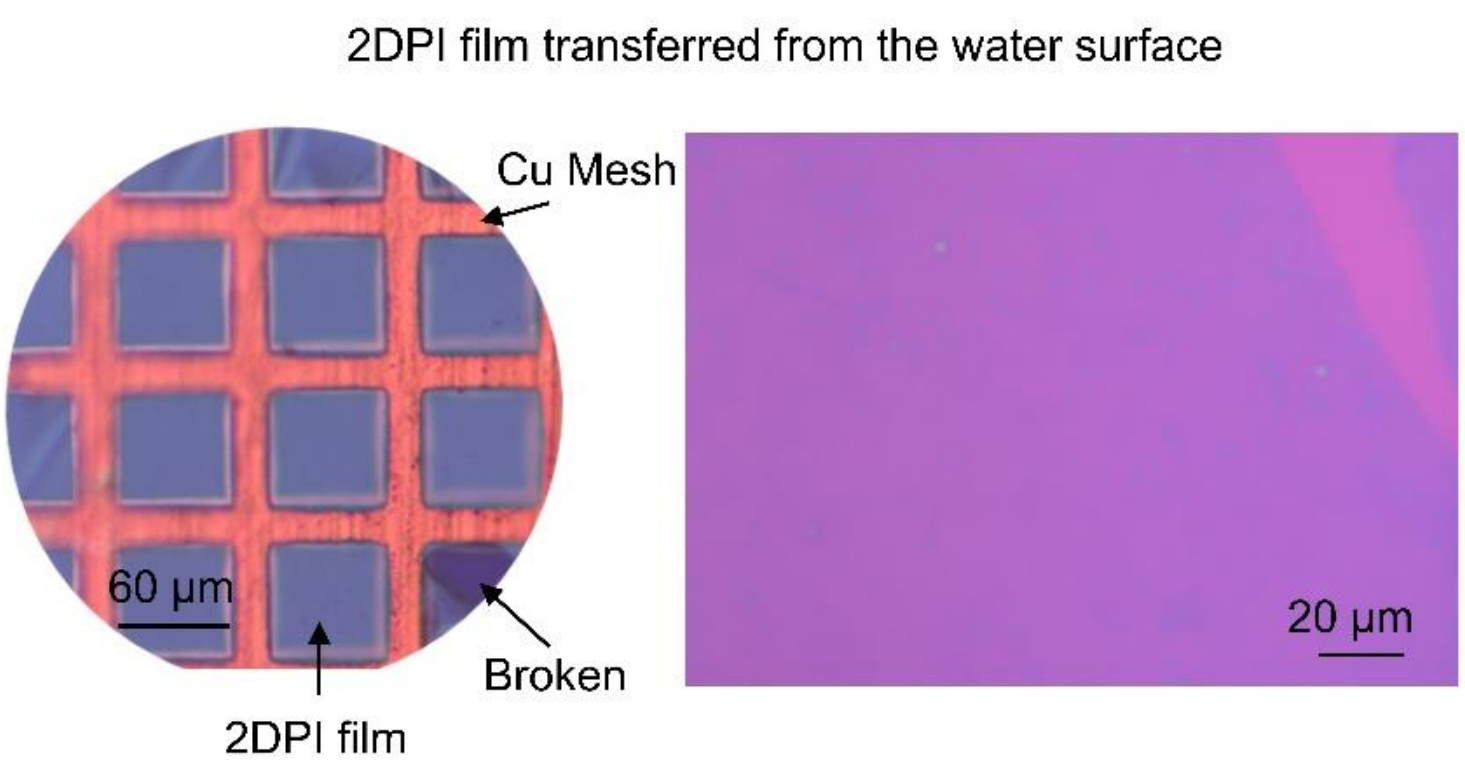

Figure R17 | Continuity of the 2DPI film on the water surface. Brewster angle microscopy images of a blank water surface and the 2DPI film in Step-III, showing clear film contrast on the water surface. Optical microscopy images of the transferred 2DPI film on a Cu mesh and SiO2/Si substrate, showing large-area film continuity with visible film edges.

Action: Figure R17 has been incorporated into the revised Supplementary Information as Supplementary Fig. 1. The corresponding discussion has been added to page 5 of the main text.

9. *The authors claimed that "Bragg reflections were observed at the same Qxy and Qz positions in the GIWAXS diffraction, with further intensity enhancement, confirming that the epitaxial alignment was preserved throughout." However, based on the current GIWAXS images, it is challenging to conclude the above results as the scattering signals are relatively weak and unclear, possibly due to the low thickness of the heterostructure. Can the authors increase the film thickness and provide better GIWAXS data for clearer comparison?*

Response: We sincerely appreciate the reviewer's comment. We agree that the original GIWAXS images did not show the weak Bragg reflections clearly because of the contrast scaling. We have therefore provided revised GIWAXS plots with improved contrast using a different color map, together with 1D line-cut profiles for clearer comparison.

The 1D profiles clearly show the characteristic Bragg reflections from Step-III to Step-V. These reflections remain at the same Q_{xy} positions, while their intensity increases with each sequential assembly step. This confirms that the in-plane epitaxial alignment is preserved during layer-by-layer growth. The measured peak positions are also consistent with the fitted peak positions, as summarized in the added table.

a

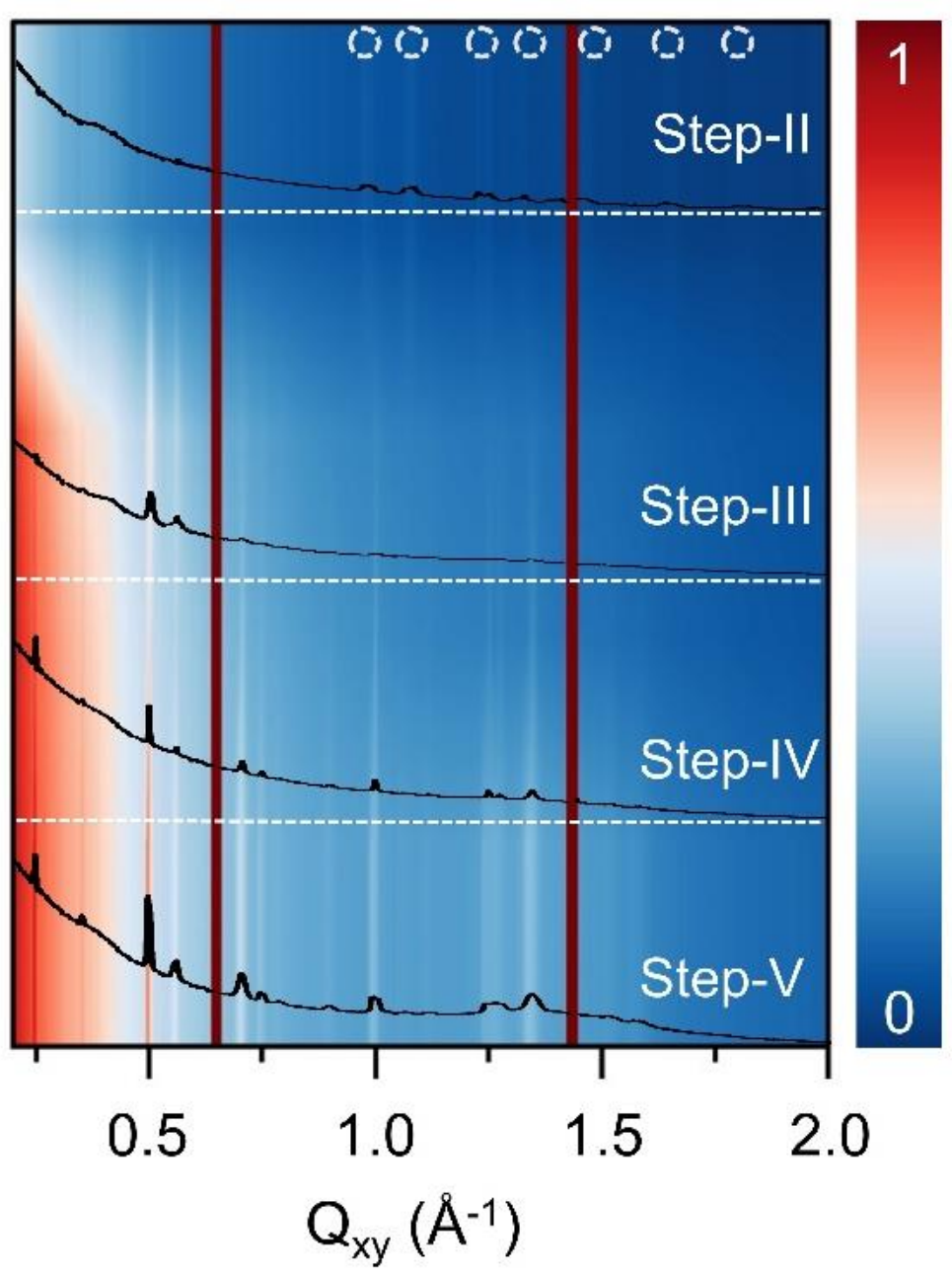

b

(hk)	Meas. peak (Å^{-1})	Fitted peak (Å^{-1})
(0, 1)	0.248	0.249
(1,-1)	0.352	0.352
(0, 2)	0.498	0.498
(1,-2)	0.557	0.557
(2,-2)	0.703	0.704
(0, 3)	0.746	0.747
(1,-3)	0.786	0.788
(2,-3)	0.896	0.898
(0, 4)	0.996	0.996
(3,-3)	1.058	1.057
(2,-4)	1.116	1.114
(0, 5)	1.256	1.246
(2,-5)	1.342	1.342
(1,-6)	1.515	1.515
(2,-6)	1.578	1.576

Figure R18 | Layer-by-layer growth analysis by ex-situ GIWAXS. Improved-contrast ex-situ GIWAXS patterns and corresponding 1D line-cut profiles from Step-III to Step-V, showing Bragg reflections at consistent Qxy positions with increasing intensity during sequential assembly.

Action: Figure R18 has been incorporated into the revised Supplementary Information as Supplementary Fig. 29 and into the main text as Fig. 2c.

10. *The bandgap of 2DPI-2DPBI vdW heterostructure was calculated to be 0.45 eV, which is smaller than that of the individual 2DPI and 2DPBI. The authors attributed this reduction to strong electronic coupling across the interface. How can this electronic coupling be formed without directional bonding between two layers?*

Response: We sincerely appreciate the reviewer's comment. The 2DPI–2DPBI vdW heterostructure shows an interlayer distance of 0.36–0.39 nm, which is slightly larger than that of graphene and comparable to many layered 2D materials. Such stacking-dependent proximity effects are well known in layered π-electron systems, including few-layer 2D polymers, 2D COFs, and 2D MOFs, where vdW stacking can modify the electronic structure and reduce the bandgap without forming interlayer covalent bonds. The influence of interlayer proximity on the electronic structure has been investigated in detail, for example, in *Adv. Funct. Mater. 30, 1908004 (2020)*, *Nat Commun 13, 7240 (2022)* and *J. Am. Chem. Soc. 2024, 146, 4, 2574–2582.*

Action: On page 12 of the revised main text, the following sentence has been added, "This reduction indicates strong electronic coupling across the interface, arising from a stacking-dependent proximity effect between the two layers[49], despite the absence of directional interlayer bonding".

Minor issues:

1. *The authors reported the use of five 2DPs, including polyimine, polyimide, polybenzimidazole, polybenzothiazole, and polyboronate ester. However, the framework of polyboronate ester polymers is often chemically unstable, especially in moisture, aqueous solutions, and air. Will the polyboronate ester-based film be decomposed after synthesis? How about its chemically stability?*

Response: We appreciate the reviewer's valuable comment. The polyboronate ester-based 2D polymer film does not show decomposition after synthesis. The film remains transferable and shows clear GIWAXS, SAED, and HRTEM signatures of an ordered 2D polymer lattice. This is consistent with reported boronate ester 2D polymer thin films prepared by the same surfactant-monolayer-assisted on-water surface strategy (*Angew. Chem. Int. Ed. 2020, 59, 8218–8224*), where the films showed sufficient stability for transfer and operation in silicon-nanowire FET-based memory devices. We note that the long-term hydrolytic stability of polyboronate ester films is expected to be lower than that of the other 2D polymer chemistries used here, such as imine, imide, imidazole, and thiazole.

2. *In the synthesis of polymer films, is the SOS surfactant directly spreading on water surface? Or is it dissolved in organic solvents like chloroform as described in their previous studies (Nat Commun 2023, 14, 8313)?*

Response: We sincerely appreciate the valuable reviewer's comment. Yes, the SOS surfactant was first dissolved in chloroform and then spread onto the water surface, following the procedure used in our previous studies (*Nat Commun 2023, 14, 8313*). After evaporation of chloroform, the SOS molecules form a surfactant monolayer on the water surface. The monomers were then introduced into the water subphase for surfactant-guided interfacial assembly and polymerization.

Action: We have added more details to the Experimental Section.

3. *To further confirm the absence of amorphous domains in the prepared heterostructures, it is suggested to provide additional HRTEM images from different film regions.*

Response: We appreciate the reviewer's valuable comment. We have added additional large-area and higher-magnification HRTEM images from different film regions to further examine structural uniformity. As shown in Figs. R3, R5 and R6, the heterostructure films show ordered crystalline domains across different regions, without clear amorphous regions at the domain boundaries. A few isolated single knot-like defects or local lattice distortions are observed.

Action: Figures R1–R7 have been incorporated into the revised Supplementary Information as Supplementary Figs. 8–14. On page 10 of the revised main text, the following discussion has been added. A detailed discussion is also provided in the Supplementary Information, see Supplementary Figs. 8–14.

Referee #2 (Remarks to the Author):

General comment: *This is a tour de force work in controlling the structure of 2D materials at a molecular level. Combining their signature "on-water" polymerization with a full toolbox of state-of-the-art characterizations, Xinliang Feng and coauthors report the synthesis of planar heterostructures of 2D semiconducting polymers and demonstrate record-large current rectification, which appears to depend on the lattice matching between the two semiconductor films. In my opinion, the work is very significant and certainly highly original (first of its kind in 2D polymer materials). I have a number of questions and comments (below), but I expect the revised manuscript to be publishable in Nature.*

Response: We greatly appreciate the reviewer's positive and insightful comments, as well as the recommendation for publication. The reviewer's suggestions have been invaluable in revising and improving our manuscript. We have carefully addressed all concerns raised by the reviewer, and point-by-point responses are provided below. We hope that the additional efforts made in the revised manuscript appropriately address the reviewer's concerns.

1. *One of the apparent limitations of this 'on-water' polymerization (compared to polymerization on crystalline surfaces) is the inability to produce polymer monolayers. What limits the lower end of the polymer thickness (reported 4.5 nm, ~10 layers), i.e., what happens at lower monomer concentration? Are there any step-edges observed by AFM? (also, the AFMs, eg Fig.S21, should be accompanied by the line height profile).*

Response: We sincerely appreciate the reviewer's valuable comment. We fully acknowledge the reviewer's point that obtaining a fully crystalline monolayer 2D polymer film by on-water surface 2D polymerization remains challenging. We attempted to further reduce the film thickness by decreasing the monomer concentration, including conditions estimated for monolayer coverage on the water surface. However, at lower concentrations, the films showed incomplete/inhomogeneous coverage and were often fragmented. We did not observe well-defined monolayer-like terraces or discrete step edges that could be assigned to individual polymer layers. Therefore, the reported lower thickness limit of ~4.5 nm does not represent the thinnest 2DPI–2DPBI vdW heterostructure film that can form, but rather the thinnest continuous and homogeneous film for which we can reproducibly resolve the crystalline structure and unit-cell parameters by GIWAXS and HRTEM.

AFM reveals a clear step edge for the 2DPI–2DPBI vdW heterostructure, corresponding to the total film thickness. Separate step edges from the individual 2D polymers are not resolved. This could arise from the commensurate epitaxial stacking between the lattice-matched 2DPI and 2DPBI lattices, in which the two 2D polymers form a continuous layer-by-layer heterostructure rather than distinguishable height terraces from of each individual component. The progressive increase in

thickness over reaction time supports controlled growth, with the heterostructure thickness tunable from 4.6 to 30 nm by adjusting the monomer adsorption/reaction time during in situ assembly.

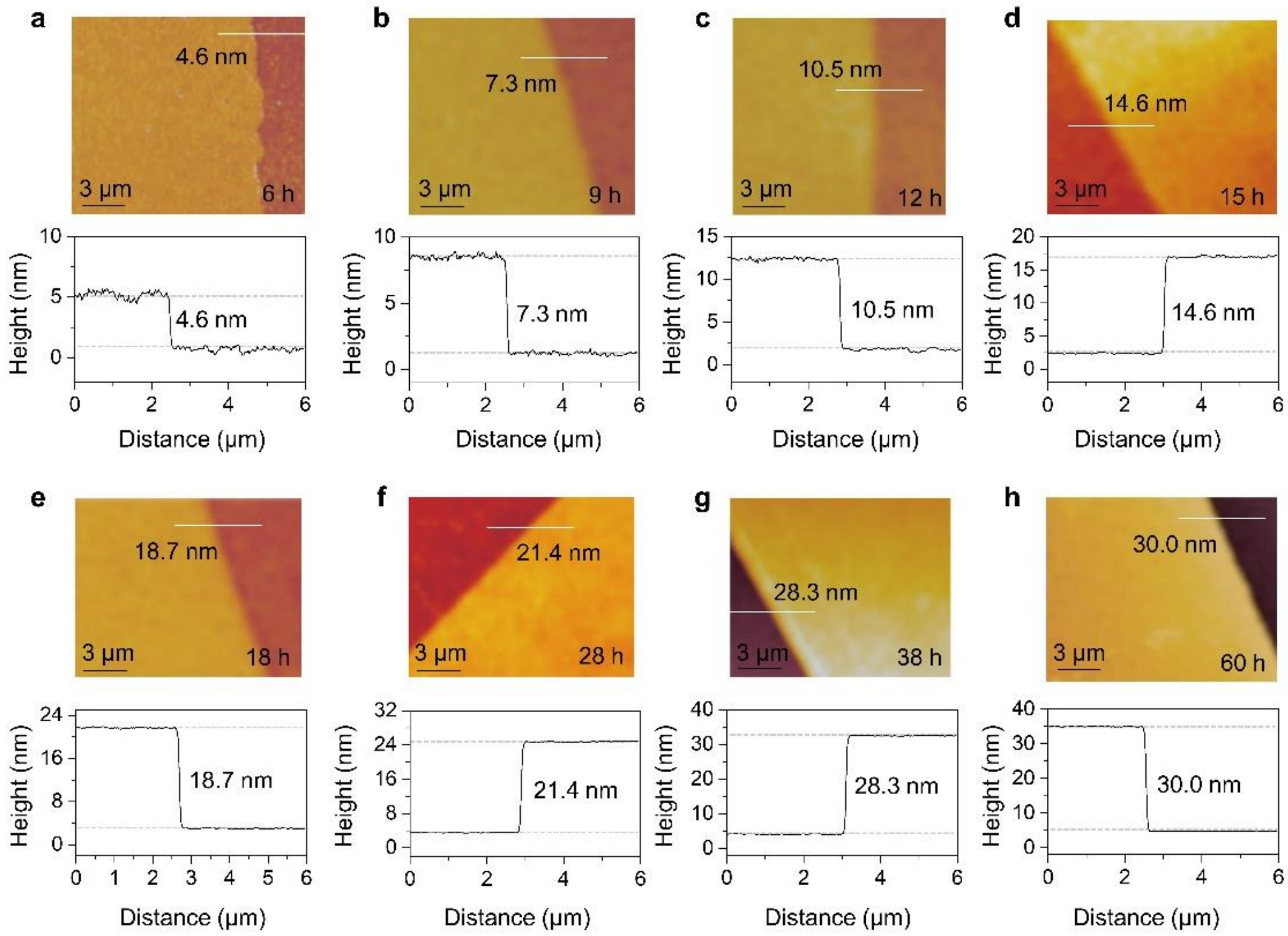

Figure R16 | Thickness control of 2D polymer vdW heterostructures. AFM images and corresponding height profiles showing the reaction-time-dependent thickness increase of the 2D polymer vdW heterostructures.

Action: Figure R16 has been incorporated into the revised Supplementary Information as Supplementary Fig. 34.

2. *The XPS depth profiling is used to support the atomically sharp interface in the heterostructure (Fig. S22). Such a significant shift seems a bit surprising given that the structure contains eight/twelve (DPI/PBI) nitrogen atoms per unit cell, and only 4 of them would be shifted to higher BE, going from DPI to DPBI. Can the protonation (acid trapping) in the lower layer be excluded (especially given the relatively high pKa of imidazole)? Fig. S22 should be supplemented with 1D spectra of the top and bottom of the film, deconvoluted. Also, it would be highly desirable to see full XPS spectra and the elemental (N/O) ratio along the film depth.*

Response: We sincerely thank the reviewer for the insightful and detailed comments. The N 1s position used previously in the depth-profile analysis was estimated from survey spectra with limited energy resolution. The high-resolution N 1s spectra show a peak centered at 399.71 eV for the top 2DPI region and 400.01 eV for the bottom 2DPBI region, corresponding to a binding-energy difference of 0.30 eV. The deconvoluted N 1s spectra are consistent with the expected nitrogen environments. For 2DPI, the component at 398.97 eV is assigned to imine nitrogen, while the component at 400.65 eV corresponds

to N–H nitrogen in the porphyrin macrocycle. For 2DPBI, the component at 399.14 eV is assigned to C=N nitrogen in benzimidazole, while the component at 400.43 eV corresponds to N–H nitrogen from benzimidazole and the porphyrin macrocycle. In the full XPS survey spectra, the top 2DPI region shows C 1s, N 1s and O 1s signals. After etching into the 2DPBI region, the O 1s signal strongly decreases, while C 1s and N 1s remain, consistent with the oxygen-free 2DPBI structure. At deeper etching cycles, O 1s, Si 2p and Si 2s signals appear from the SiO_2/Si substrate. The N/O depth profile shows a clear transition from oxygen-containing 2DPI to oxygen-free 2DPBI, supporting a sharp chemical interface.

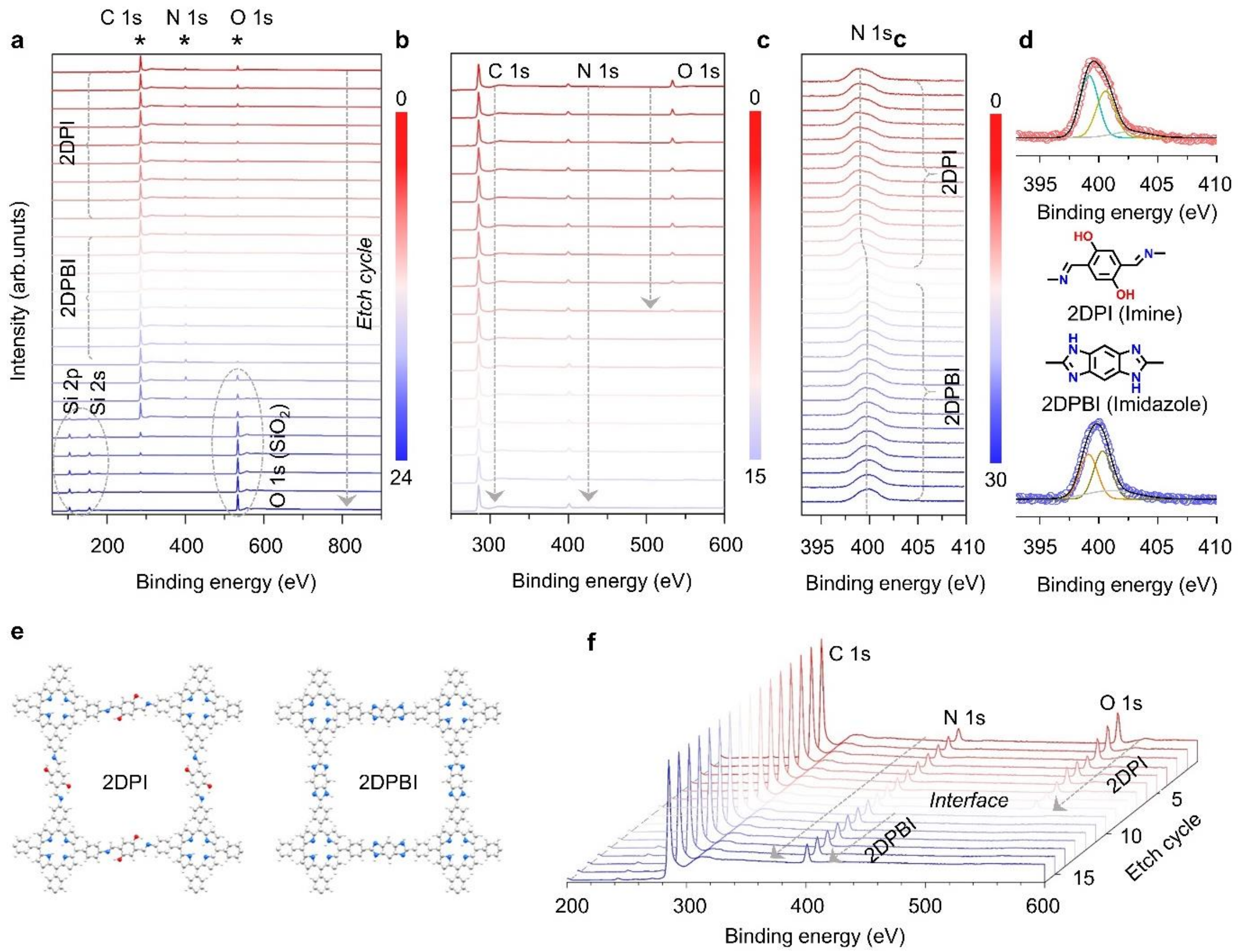

Figure R19 | XPS depth-profile analysis of the 2DPI–2DPBI vdW heterostructure. (a) Full XPS survey spectra collected along the etching depth. (b) Enlarged survey spectra showing the evolution of C 1s, N 1s, and O 1s signals. (c) High-resolution N 1s spectra along the depth profile. (d) Deconvoluted N 1s spectra from the top 2DPI region and bottom 2DPBI region. (e) Chemical structures of 2DPI and 2DPBI. (f) 3D representation of the survey spectra in (b), highlighting the transition across the 2DPI–2DPBI interface.

To further check possible acid trapping/protonation, we performed pH-dependent UV–vis control experiments. Upon acid treatment of the as-synthesized films, protonation of the porphyrin core gives clear red shifts of the Soret and Q bands. For 2DPI, the Soret band shifts from 421.6 nm in the as-synthesized film to 423.8 nm at pH 4.8 and 442.15 nm at pH 1.6. For 2DPBI, the Soret band shifts

from 431.5 nm in the as-synthesized film to 435 nm at pH 4.7 and 460 nm at pH 1.4. Similar red shifts are observed in the Q-band region. These changes provide clear spectroscopic signatures of porphyrin protonation. In contrast, the as-synthesized 2DPI and 2DPBI films do not show these protonation-induced red-shifted features. This is consistent with our synthesis protocol, in which excess monomers and acidic species are removed at each step and the subphase is exchanged with fresh Milli-Q water. These results support neutral free-base porphyrin cores in both 2DPI and 2DPBI and exclude significant acid trapping/protonation.

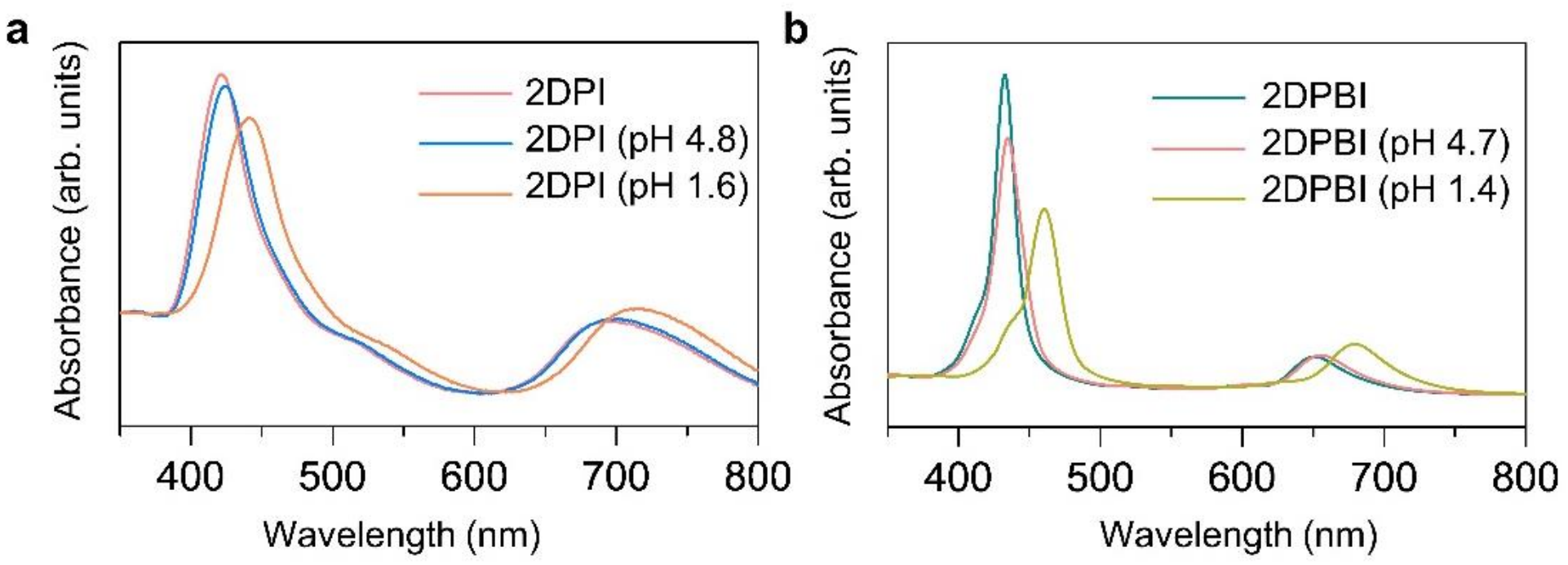

Figure R20 | pH-dependent UV–vis spectra of 2DPI and 2DPBI films. UV–vis absorption spectra of (a) 2DPI and (b) 2DPBI films before and after acid treatment, showing protonation-induced red shifts of the Soret and Q bands under acidic conditions.

Action: Figures R19 and R20 have been added to the revised Supplementary Information as Supplementary Figs. 35 and 38, respectively. The corresponding discussion has also been added in relation to Supplementary Figs. 35 and 38.

3. *I am confused by the discussion of the (calculated) interfacial band alignment. The manuscript suggests a work function of 4.81 eV, which should place the Fermi level below the VBM =-4.29 eV (although Fig. 4 shows it above the VBM??). I suppose such lowering of the Fermi level is possible due to charge transfer, but it also highlights that the structure should have two different work functions on the opposite sides of the film.*

Response: We thank the reviewer for this detailed observation.

Regarding the work function and Fermi level placement:

When describing 2D periodic systems in a 3D periodic cell, the work function is formally defined as:

$$\varphi = V_{\_vacuum} - E_{\_Fermi}$$

We have carefully re-examined the band alignment calculations with rigorous reference to the vacuum plateau extracted from the planar-averaged electrostatic potential (LOCPOT) following the standard VASP protocol (φ = V_vacuum − E_Fermi). The corrected work function of the 2DPI-2DPBI

heterostructure is φ = 4.065 eV, with the Fermi level correctly placed at -4.065 eV with respect to vacuum, sitting within the bandgap above the VBM at -4.29 eV and below the CBM at -3.84 eV, consistent with Figure 4a. The individual layer work functions, obtained from separate independent calculations for each isolated layer, are φ_2DPI = 4.203 eV and φ_2DPBI = 3.973 eV. The work function offset of 0.23 eV between the two constituent layers drives Fermi-level equilibration upon contact, with electrons transferring from 2DPBI (lower φ) to 2DPI (higher φ), establishing the common Fermi level at -4.065 eV. This charge transfer is consistent with the charge density difference analysis (Figure 4d), which shows electron depletion from 2DPBI and accumulation on 2DPI, with a net transfer of approximately 0.05 e per unit cell. Figure 4a has been revised accordingly.

Regarding the two different work functions on opposite faces of the film: The reviewer correctly identifies that the two faces of the heterostructure are not electrostatically equivalent. The work function of φ = 4.065 eV is a single bulk quantity computed for the heterostructure as a whole. The individual bulk work functions of φ_2DPI and φ_2DPBI are obtained from separate independent calculations and reflecting the intrinsic electronic structure of each polymer network. The interfacial dipole moment of 0.93 e·Å (surface dipole density: 1.45×10^{-3} e/Å, Figure 4e), oriented normal to the plane of the layers and determined from a single-point calculation with dipole correction applied (LDIPOL = T, DIPOL = 0.5 0.5 0.5), gives rise to a built-in potential step of 2.11 eV across the interface (Figure 4b). From the bulk work function, the interfacial dipole moment, and the built-in potential step, we conclude that the surface potential differs on the two faces of the heterostructure, consistent with the reviewer's observation. This asymmetry is a direct physical consequence of the interfacial dipole rather than an independently computed surface quantity.

We further note that the built-in potential step of 2.11 eV significantly exceeds the work function difference of 0.23 eV between the isolated layers, indicating that the dominant contribution to the electrostatic asymmetry arises from the interfacial dipole alignment rather than from Fermi-level equilibration alone. This is well established in organic heterojunction systems where interfacial molecular dipoles routinely produce potential steps far exceeding the contact potential, and is the central physical result underpinning the charge transport properties reported. The calculated potential step of 2.11 eV was obtained from a 4+4 layer model, which closely approximates the experimental film thickness. In the experimental structure, where the polymer films may extend beyond the thickness of the computational model, the macroscopic built-in potential experienced across the full device may be modulated by additional electrostatic screening from layers further from the interface.

Action: On page 12 of the revised main text, the following sentence has been added, "Normalized by the unit cell area, this corresponds to a surface dipole density of 1.45×10^{-3} e/Å. Additionally, the planar-averaged electrostatic potential shows a built-in potential step of approximately 2.11 eV across the interface (Fig. 4b). Therefore, from the bulk work function, the interfacial dipole moment, and the

built-in potential step, we conclude that the surface potential differs on the two faces of the heterostructure, which is a direct physical consequence of the interfacial dipole.” The corresponding detailed discussion has also been added, see Supplementary Information Page 66.

4. *The transport measurements are performed using conductive AFM. The big advantage of this approach is the ability to rapidly acquire a significant amount of data on a given sample, but only one curve is shown per structure/condition. A statistical analysis of the current and rectification ratio should be reported (and the uncertainties included in Fig. 5h). A major limitation of c-AFM measurements is that the junction characteristics depend strongly on the tip-surface separation, and an asymmetric conductance (although probably not 10^7) can be observed under certain conditions even in a perfectly symmetric film. The set point of the measurement should be reported (also, the tip diameter).*

Response: We sincerely thank the reviewer for the constructive suggestion. We clarify that the transport data in Fig. 5 were obtained from fabricated vertical micro-devices, rather than from c-AFM measurements. We have now added statistical analysis from multiple micro-devices. The revised Fig. 5h includes multiple I–V curves, extracted rectification ratios, and error bars showing the statistical uncertainty. We also performed new nanoscale c-AFM I–V measurements on the 2DPI–2DPBI vdW heterostructure. Measurements were collected from five independent samples, with five spatially distinct points measured on each sample. The c-AFM data show reproducible diode-like behavior, with rectification ratios at ±1 V in the range of 10^4–10^5, consistent with the vertical micro-device measurements at the same bias window.

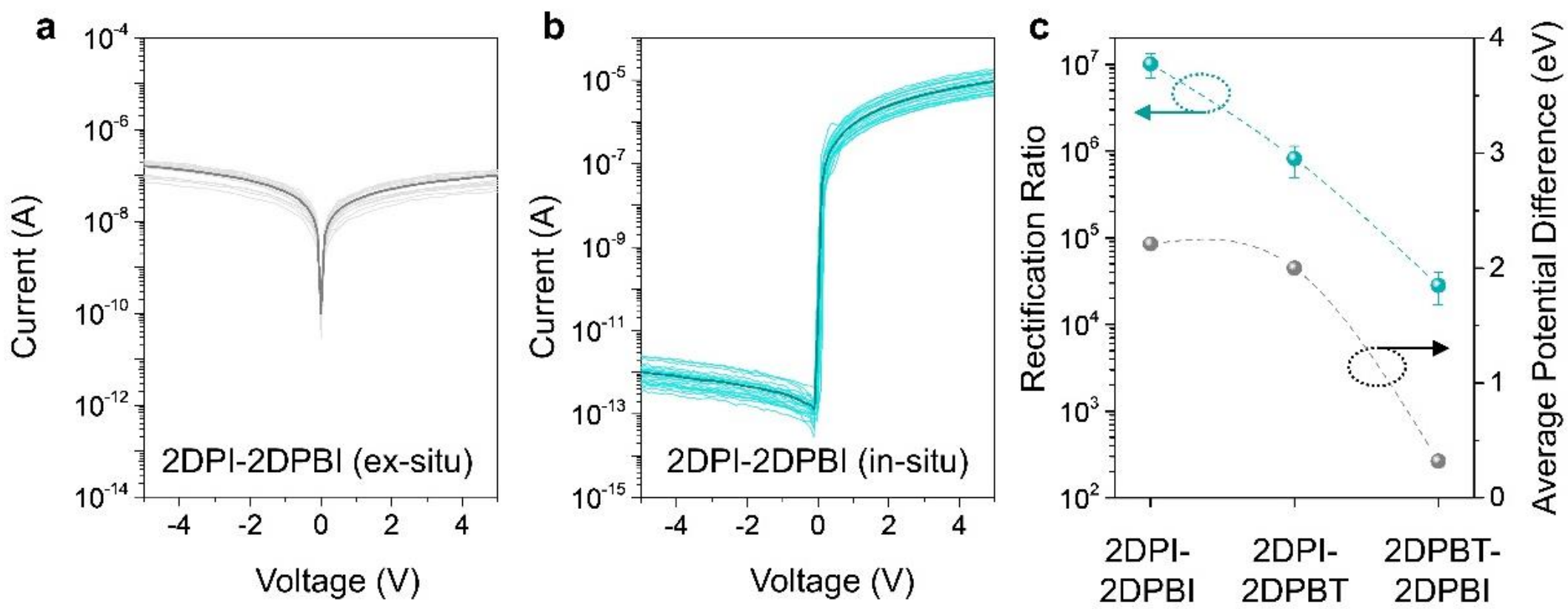

Figure R21 | Statistical transport analysis of vertical micro-devices. (a,b) I–V curves of fabricated vertical micro-devices based on ex-situ and in-situ 2DPI–2DPBI vdW heterostructures. (c) Statistical comparison of rectification ratio and average potential difference for different 2D polymer vdW heterostructure devices.

We conducted electrical characterization of the 2DPI–2DPBI vdW heterostructures utilizing c-AFM equipped with cantilevered solid platinum tips (25Pt300B-10, Park Systems). According to the

manufacturer, these tips possess a nominal apex radius of 10 nm. For sample preparation, the heterostructure layers were mechanically and wet-transferred onto a 100 nm Cr-Au/n++ Si substrate. This substrate was subsequently mounted onto a stainless-steel AFM chuck using silver paste, which was also applied to the sample corners to ensure robust electrical contact between the substrate and the chuck. To establish consistent mechanical contact while preventing physical degradation of the 2DPI–2DPBI vdW heterostructure film, the tip pressing force was maintained between 400 and 550 nN; the sample surface was concurrently monitored for damage using an integrated optical microscope. Measurements were performed point-by-point by applying a DC bias voltage between the AFM cantilever and the stainless-steel chuck to collect the resulting current.

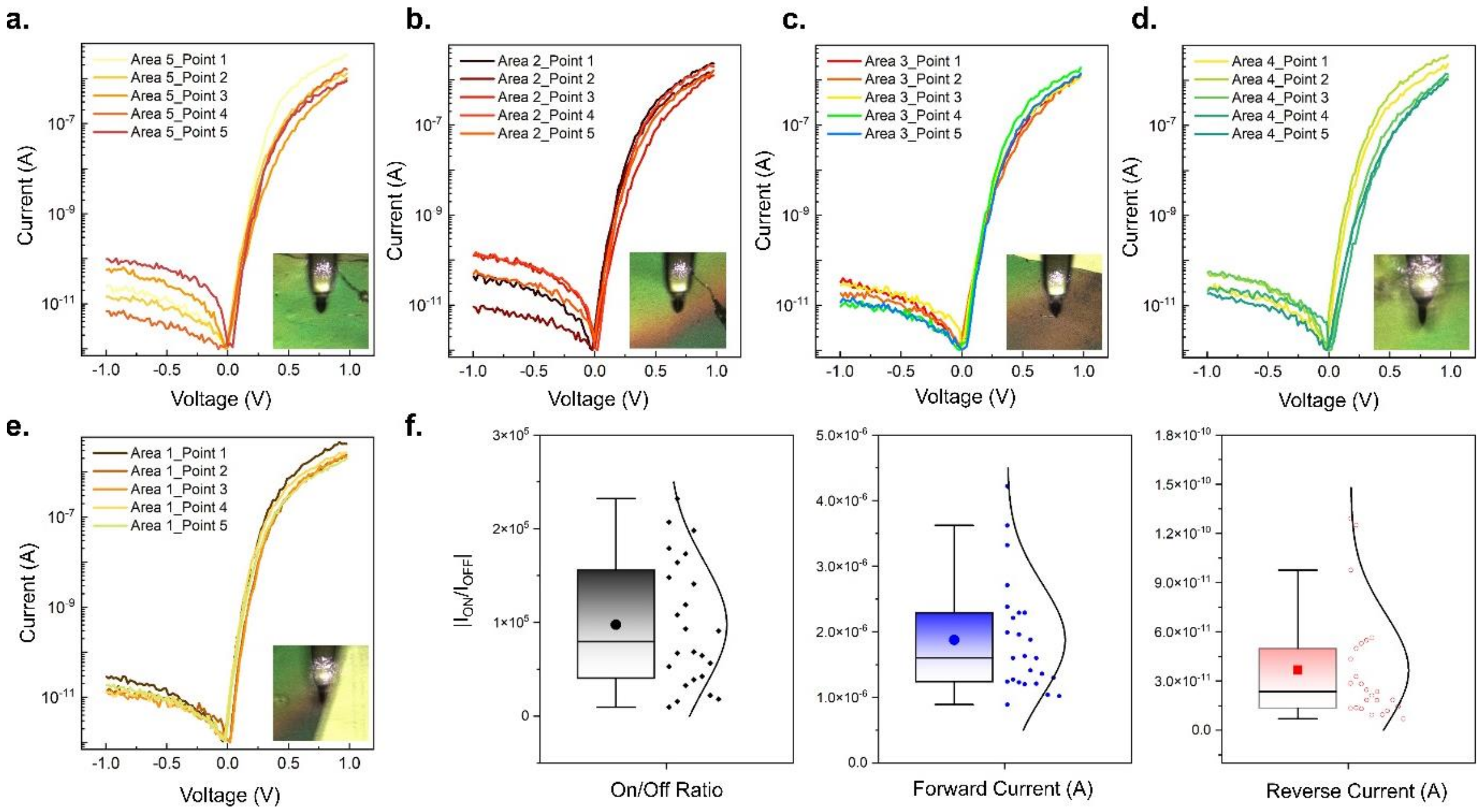

Figure R11 | Statistical c-AFM I–V characterization of 2DPI–2DPBI vdW heterostructures. (a–e) c-AFM I–V curves measured from five independent 2DPI–2DPBI vdW heterostructure samples, with five spatially distinct points collected from each sample. Insets show optical images of the cantilever approaching the sample surface. (f) Statistical analysis of the extracted transport parameters, including rectification ratio, forward current and reverse current.

Action: Figures R21 and R11 have been added to the revised Supplementary Information as Supplementary Figs. 53 and 55, respectively. The corresponding discussion has also been added.

5. *A remarkable and somewhat unexpected (to me at least) finding of the work is that the rectification depends on the epitaxy between the layers, which is supported by the symmetric conductance of the 'ex-situ' prepared DPI/DPBI heterostructure with a random layer orientation. However, I did not find any explanation for this behavior. The manuscript (line 360) implies that random orientation leads to "absence of interfacial dipole," but it's not clear to me why this would be the case (or how the authors*

established this point). Also, the measurements of "lattice MIS-matched" films are not discussed in the manuscript; Fig. S37 is important but is 'hidden' in the SI.

Response: We sincerely thank the reviewer for this important comment. We have revised the discussion to clarify why rectification depends on epitaxial registry.

In the in-situ synthesized 2DPI–2DPBI vdW heterostructure, the commensurate epitaxial alignment enables directional interfacial charge redistribution between the two 2D polymer layers. The calculated built-in potential step of 2.11 eV and interfacial dipole moment of 0.93 e·Å, oriented normal to the layers, arise from this asymmetric charge transfer. Such a coherent interfacial dipole is favored when the two lattices are in epitaxial registry, because the frontier-orbital overlap across the interface is spatially well defined.

In contrast, in the ex-situ mechanically stacked 2DPI-2DPBI heterostructure, the two layers have random rotational orientations. This produces non-uniform local registry and disordered interfacial orbital overlap. As a result, local dipole contributions are not coherently aligned across the device area, strongly reducing the macroscopic built-in electrostatic asymmetry. This explains the nearly symmetric conductance observed for the ex-situ heterostructure.

The vertical charge-transport measurements of the lattice-mismatched heterostructures further support the role of lattice registry. The rectification ratio is highest for lattice-matched heterostructures, reaching 10^7–10^4, decreases to 10^3–10^2 for the small-mismatch heterostructure, and approaches only ~10 for large-mismatch heterostructures. In the small-mismatch case, partial local registry is still preserved, allowing some interfacial orbital overlap. In contrast, for 16.6% and 18.0% lattice-mismatched heterostructures, the relative registry changes strongly across the Moiré pattern, reducing the density of closely matched interfacial sites. This weakens coherent interfacial charge redistribution and the built-in electrostatic asymmetry, leading to much weaker rectification.

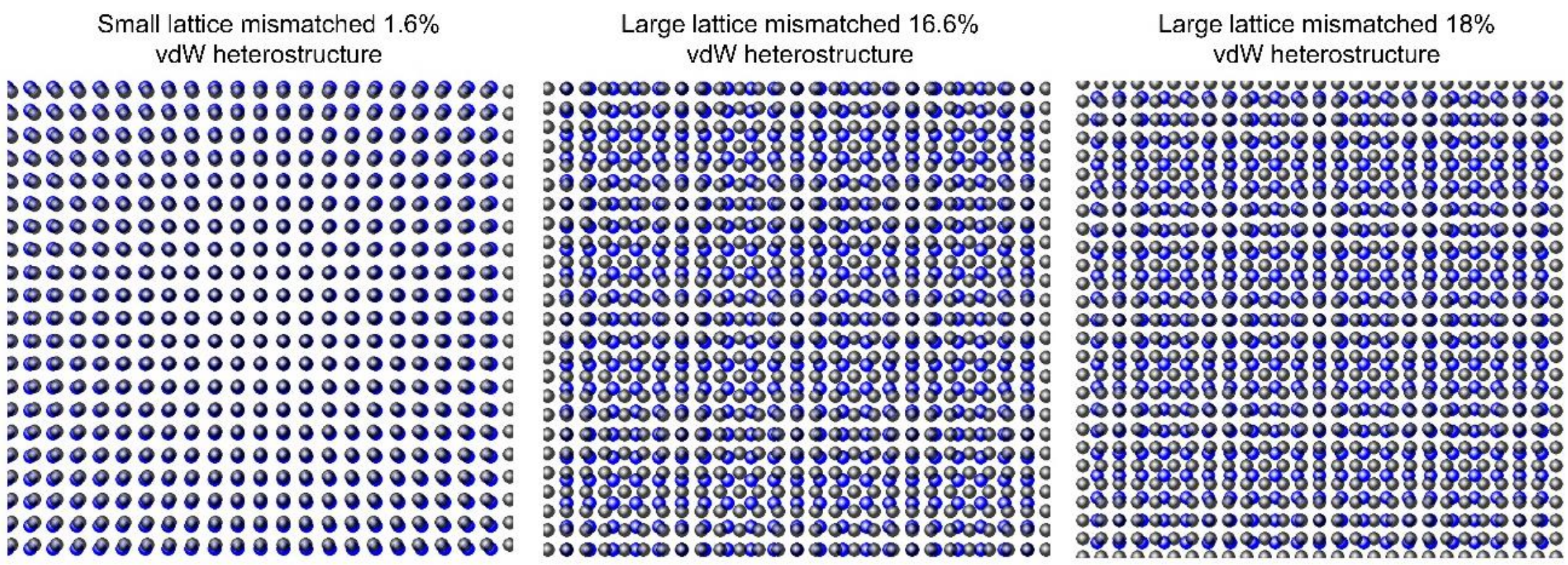

Figure R22 | Structural models of lattice-mismatched 2D polymer vdW heterostructures. Schematic models showing the interfacial lattice registry for small-mismatch and large-mismatch vdW

heterostructures, illustrating the reduced density of closely matched interfacial sites with increasing lattice mismatch.

Action: We thank the reviewer for this valuable suggestion. Accordingly, we have moved the key data previously shown in Supplementary Fig. 37 into the main text as Fig. 5h. The measurements and discussion of the lattice-mismatched films have also been expanded in the revised Supplementary Information; Supplementary Figs. 53–57.

Minor issues:

1. *The facile formation of benzimidazole from carboxylic acid and phenylenediamine at RT would be surprising for many chemists (it usually requires high temperatures and activation). I don't dispute the results (WAXS and the MALDI-TOF of the molecular product in ref. 46 seem convincing); however, the manuscript would benefit from some explanation of this unusually facile reaction (in the SI). Also, if the DPDBI (and DPBT) polymers/COFs prepared under THESE conditions have not been reported before, it would be great to include spectral evidence for the reaction (e.g., FTIR, XPS).*

Response: We sincerely appreciate the reviewer for this important comment. We agree that azole and imide formation typically requires high temperature or activation in conventional bulk reactions. In our synthesis approach, however, the reactions occur through on-water surface synthesis, where the on-water surface effect and surfactant-confined pre-assembly play key roles in enhancing reactivity and selectivity. This has been supported by model reactions reported in our previous studies. For the imidazole model reaction, the reaction between R1 and R2 on the CTAB-confined water surface at room temperature gave the target benzimidazole product MC-1 with m/z = 730.48, as confirmed by MALDI–TOF MS, whereas the same reaction in water without surfactant gave only the starting reagents. Similar behaviour was observed for imide formation, where imidization proceeded at room temperature on the water surface, while the same reaction in aqueous solution did not give the product (*Nat. Chem.* 11, 994–1000, 2019; *Nat. Commun. 14, 8313, 2023*).

Following the reviewer's suggestion, we have added FTIR and XPS characterization of 2DPBI and 2DPBT prepared under the present conditions. For 2DPBI, comparison with the precursor monomers M3 and M4 shows that the N–H band of monomer M4 shifts from 3370 cm^{-1} to 3312 cm^{-1}, consistent with hydrogen-bonded N–H in the benzimidazole ring. A new band appears at 1622 cm^{-1}, assigned to C=N stretching in the benzimidazole linkage, while the C=O band of monomer M3 at 1708 cm^{-1} disappears, confirming formation of the benzimidazole-linked 2D polymer. For 2DPBT, comparison with monomers M3 and M5 shows the disappearance of the N–H band (3430 cm^{-1}) and the S–H band (2564 cm^{-1}). New bands appear around 1610 cm^{-1} (C=N) and 710 cm^{-1} (C–S–C), confirming benzothiazole linkage formation. Additionally, the C=O band from M3 at 1708 cm^{-1} disappears, confirming complete conversion to the benzothiazole-linked 2D polymer.

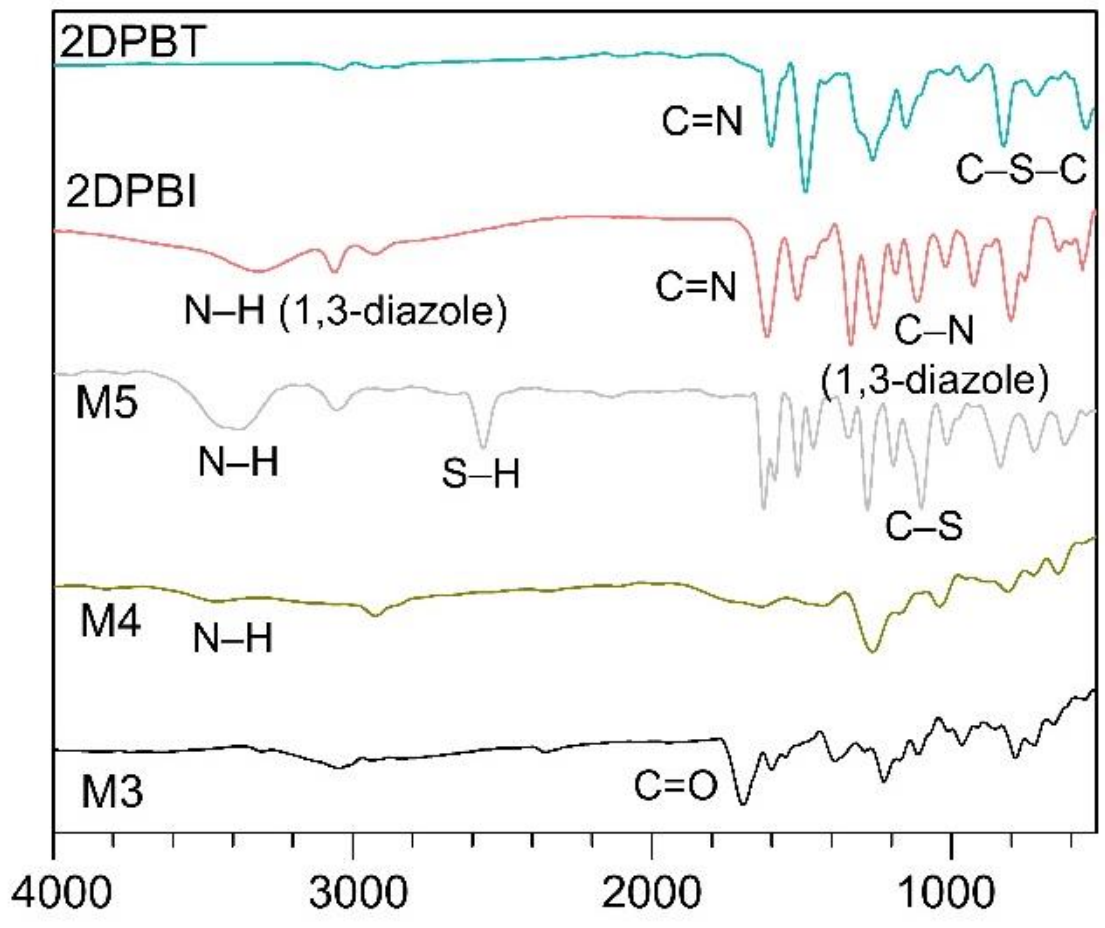

Figure R23 | FTIR characterization of 2DPBT and 2DPBI formation. FTIR spectra of precursor monomers M3–M5 and the corresponding 2D polymers.

XPS analysis further supports the formation of the diazole-linked 2D polymers. For 2DPBI, the N 1s spectrum was deconvoluted into two components at 398.5 and 400.1 eV, assigned to imine- and amine-type nitrogen atoms in the 1,3-diazole linkage, respectively. Similarly, 2DPBT showed N 1s components at 398.6 and 400.0 eV, corresponding to imine- and amine-type nitrogen environments in the thiazole–diazole linkage. The S 2p spectrum of 2DPBT displayed a characteristic spin–orbit doublet with S $2p_{3/2}$ and S $2p_{1/2}$ peaks at 164.1 and 165.3 eV, respectively, consistent with chemically bound sulfur in the polymer backbone.

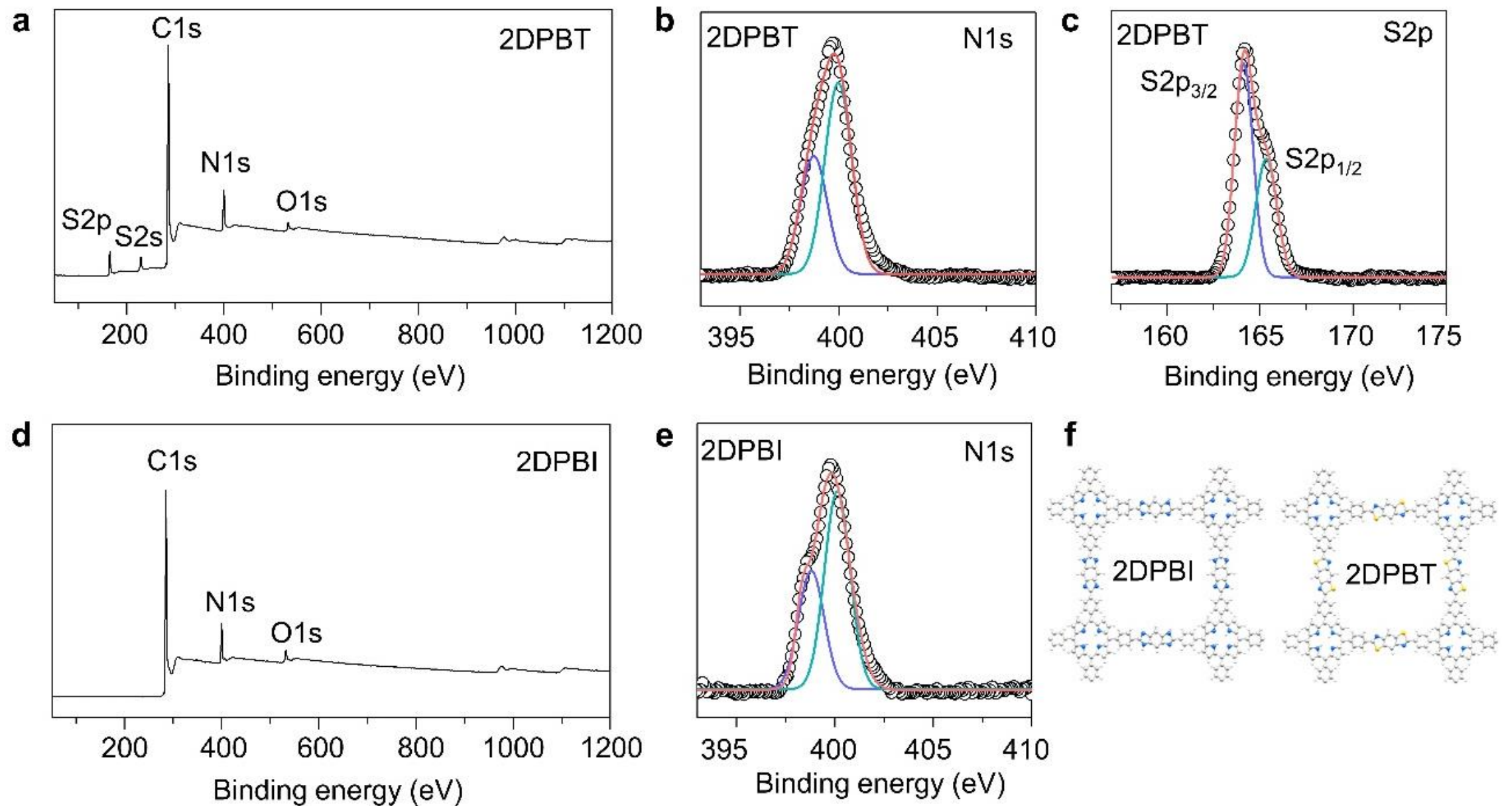

Figure R24 | XPS characterization of 2DPBT and 2DPBI. a–c, XPS survey, N 1s and S 2p spectra of 2DPBT, showing imine/amine nitrogen components and the characteristic S 2p doublet. **d,e,** XPS survey and N 1s spectra of 2DPBI, resolving imine- and amine-type nitrogen environments. **f,** Structural models of 2DPBI and 2DPBT.

Action: Figures R23 and R24 have been added to the revised Supplementary Information as Supplementary Figs. 51 and 52, respectively.

2. *Why the assembled M1 shows rings in GIWAXS? It implies a disordered crystal orientation, which seems to contradict the presented cartoons of the assembly.*

Response: We sincerely thank the reviewer for this important comment. The ring-like GIWAXS feature of the Step-II M1 assembly reflects an azimuthal distribution of many small crystalline domains over the macroscopic X-ray beam area, rather than molecular-level disorder within each domain. In GIWAXS, the diffraction signal is averaged over a large area on the water surface, typically on the scale of a few centimeters (~2 cm). In Step-II, the SOS–M1 assembly is mainly a monolayer/few-layer pre-assembly with relatively low scattering density. Therefore, even if each local domain is ordered, the presence of many differently oriented domains within the large GIWAXS footprint gives ring-like diffraction features.

In contrast, HRTEM probes local nanoscale regions. The transferred SOS–M1 and CTAB–M3 assemblies show well-resolved square molecular lattices over local regions, confirming local crystalline ordering in Step-II. Therefore, the cartoons represent the local ordered monomer arrangement derived from HRTEM, whereas the GIWAXS rings reflect the macroscopic orientational distribution of such ordered domains over the large beam area. To further support Step-II pre-assembly, AFM measurements of the transferred SOS–M1 assembly show thicknesses of 2.56 nm after 30 min and 2.61 nm after 10 h. These values match the combined thickness of the SOS monolayer and M1 molecule, supporting monolayer/few-layer M1 pre-assembly beneath the SOS surfactant monolayer.

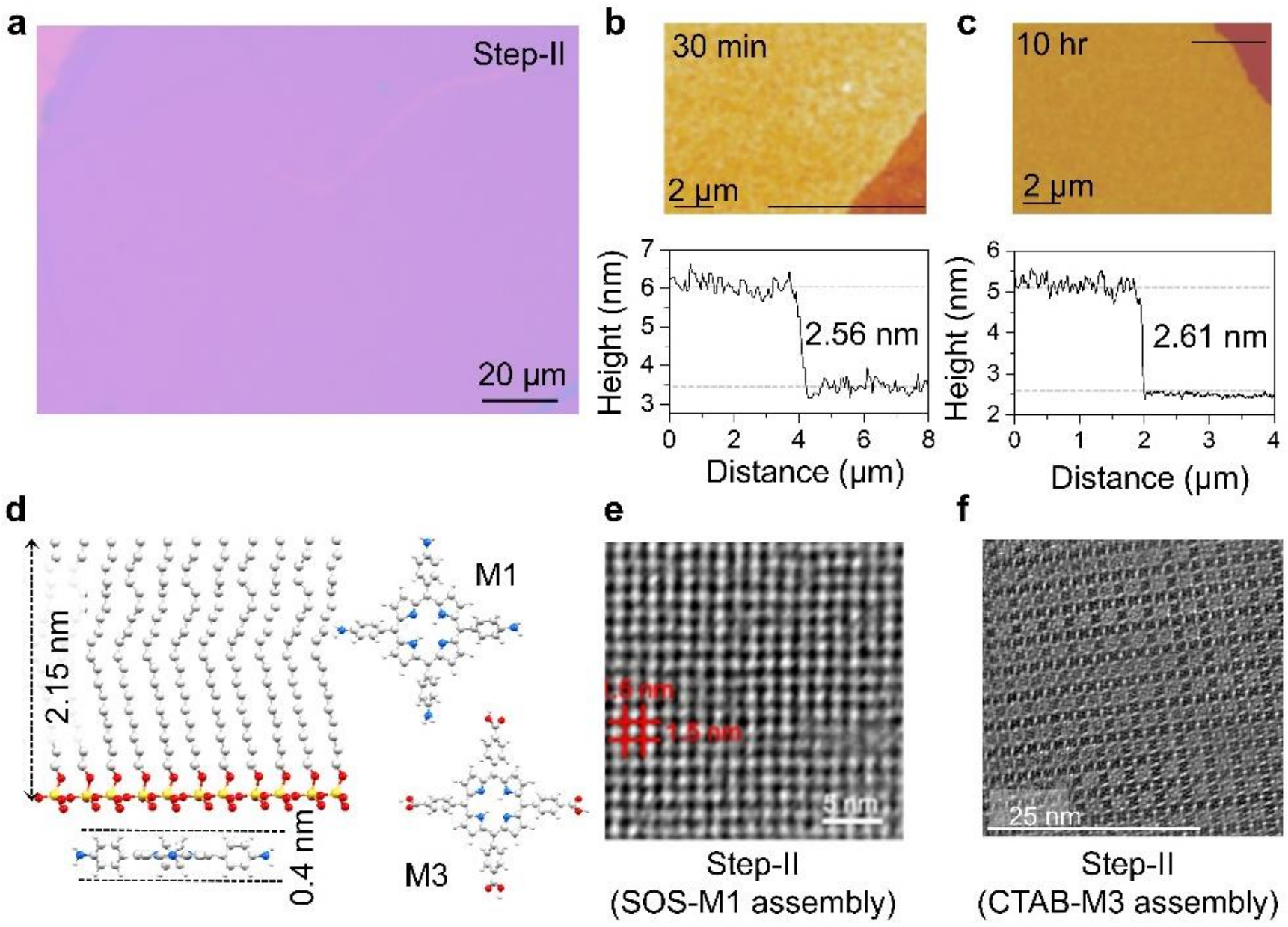

Figure R12 | Step-II monomer pre-assembly underneath charged surfactant monolayers. (a) Optical microscopy image of the SOS–M1 assembly formed in Step-II. (b,c) AFM images and height profiles of the SOS–M1 assembly after M1 adsorption for 30 min and 10 h, showing thicknesses of 2.56 and 2.61 nm, respectively. (d) Schematic thickness comparison of the SOS monolayer and M1/M3 molecules. (e) HRTEM image of the SOS–M1 assembly in Step-II, adapted from ref. 45 (Nat. Chem. 11, 994–1000, 2019). (f) HRTEM image of the CTAB–M3 assembly in Step-II.

Action: Figure R12 has been added to the revised Supplementary Information. The corresponding discussion clarifying the GIWAXS ring features and local HRTEM-resolved ordered assemblies has also been added, see Supplementary Fig.20.

3. *Line 255: "This behavior is likely due to the ultrafast formation of highly localized excitonic states, which exhibit negligible conductivity." The phrasing is confusing, as the localized exciton should form with a similar rate in the homogenous films as well (followed by possible charge separation). Overall, the stated lack of photoconductivity may need further explanation/context. The photoinduced formation of charge carriers is commonly observed in single-phase organic materials (likely facilitated by defects), and, if I am not mistaken, has been widely employed to probe charge mobility of similar homogenous Schiff base COFs. What's different in this case?*

Response: We thank the reviewer for this insightful comment and agree that the original phrasing was not sufficiently precise. We have revised the text to clarify both the nature of the photoexcited states and the absence of measurable photoconductivity in the individual films.

For (1), we agree that exciton formation is expected to occur on ultrafast timescales also in the homogeneous films. Our intention was not to suggest a fundamentally different formation rate, but rather to emphasize the fate of the photoexcited states. In the revised manuscript, we clarify that: Photoexcitation in the individual 2DPI and 2DPBI films does generate excited states, as independently confirmed by time-resolved photoluminescence measurements, which show clear emission and decay dynamics. However, on the same time scale, the absence of a detectable real *and* imaginary photoconductivity signal in the THz measurements indicates that these excitations do not evolve into mobile or even weakly polarizable charge carriers. This behavior is most consistent with the formation of strongly localized excitonic states with very low polarizability. While carrier trapping could, in principle, also suppress conductivity, the observed photoluminescence following the OPTP decay time demonstrates that complete trapping into dark, non-radiative states is unlikely. All these results suggest that the excitons are highly localized ("compact") with low polarizability, limiting their contribution to the THz response.

For (2), we appreciate the reviewer's point that photoinduced charge generation is commonly observed in single-phase organic semiconductors, including COFs, often facilitated by defects or energetic disorder. In this context, we now clarify that the behavior observed here differs in several important aspects: The studied 2D polymer films exhibit a high degree of crystallinity and structural order, as

evidenced by GIWAXS and HRTEM, which likely reduces the density of defect states that could assist exciton dissociation into free carriers. In the absence of a driving force for charge separation (e.g., energetic offsets or built-in electric fields), excitons remain bound and recombine rather than dissociate. In contrast, in the lattice-matched heterostructure, the type-II band alignment and built-in interfacial electric field provide an efficient pathway for exciton dissociation into spatially separated free carriers, which is directly reflected in the emergence of a finite photoconductivity signal. We therefore attribute the key difference not to the absence of initial charge generation, but to the lack of efficient exciton dissociation channels in the individual films.

Action: We have revised the corresponding passage in the manuscript to reflect this more precise interpretation and to better distinguish between exciton formation, localization, and dissociation on page 10 as follows, "On the other hand, time-resolved photoluminescence measurements (Figure 3e,f) show clear emission and decay dynamics up to ~200 ps, with substantially reduced PL for the heterostructure. The absence of photoconductivity and strong PL in the individual layers is consistent with the ultrafast formation of highly localized excitonic states, which exhibit negligible conductivity and polarizability in the THz frequency. In contrast, the finite photoconductivity in 2DPI–2DPBI vdW heterostructure testifies to the generation of free carriers resulting from efficient charge separation across the interface, with electrons localizing in 2DPI and holes accumulating in 2DPBI (see simulated interfacial energetics in later section)."

4. *Fig. 3d seems important but is not discussed. Are the differences between the absorption profiles of 2DPI and 2DPBI due to different stacking (it seems unlikely that these are due to different in-plane conjugation)? Can the authors exclude the possible difference in the porphyrin protonation state (given the reaction performed in HCl solution...)?*

Response: We sincerely thank the reviewer for this important comment. To check possible acid trapping/protonation, we performed pH-dependent UV–vis control experiments. Upon acid treatment, protonation of the porphyrin core gives clear red shifts of the Soret and Q bands. For 2DPI, the Soret band shifts from 421.6 nm in the as-synthesized film to 423.8 nm at pH 4.8 and 442.15 nm at pH 1.6. For 2DPBI, the Soret band shifts from 431.5 nm in the as-synthesized film to 435 nm at pH 4.7 and 460 nm at pH 1.4. Similar red shifts are also observed in the Q-band region. These changes provide clear spectroscopic signatures of porphyrin protonation.

In contrast, the as-synthesized 2DPI and 2DPBI films do not show these protonation-induced red-shifted features. This is consistent with our synthesis protocol, in which excess monomers and acidic species are removed at each step and the subphase is exchanged with fresh Milli-Q water. These results support neutral free-base porphyrin cores in both 2DPI and 2DPBI and exclude significant acid trapping/protonation.

Action: Figure R20 has been added to the revised Supplementary Information as Supplementary Fig. 38. The corresponding discussion has also been added to page 11 of the main text and to the Supplementary Information; see Supplementary Fig. 38.

5. *What data is the "average potential difference" in Fig. 5h based on? Depth profiling UPS? (it's only shown for one heterostructure, what about the other two?)*

Response: We thank the reviewer for this question. The "average potential difference" in Fig. 5h is not obtained from depth-profiling UPS. It is a theoretical value calculated from the planar-averaged electrostatic potential of each lattice-matched heterostructure.

The planar-averaged potential was calculated from the DFT electrostatic potential by averaging over the lateral **xy** plane along the stacking direction:

$$\bar{V}(z_0) = \frac{1}{A}\int_A V(x, y, z_0)\, dx\, dy$$

where **A** is the surface area of the unit cell. The average potential difference is defined as the potential step between the two sides of the heterostructure slab. This value reflects the built-in electrostatic asymmetry generated by the interfacial dipole.

We calculated this value for all three lattice-matched heterostructures: 2.11 eV for 2DPI–2DPBI, 2.0 eV for 2DPBT–2DPI, and 0.32 eV for 2DPBT–2DPBI. These theoretical values are plotted on the right y-axis of Fig. 5h and compared with the experimentally measured rectification ratios. The correlation shows that a larger interfacial potential difference is associated with stronger rectification.

This quantity is meaningful for heterostructures because it arises from the electrostatic asymmetry between two chemically distinct 2D polymers. For an individual 2D polymer, the two slab surfaces are equivalent and no interfacial potential step is expected.

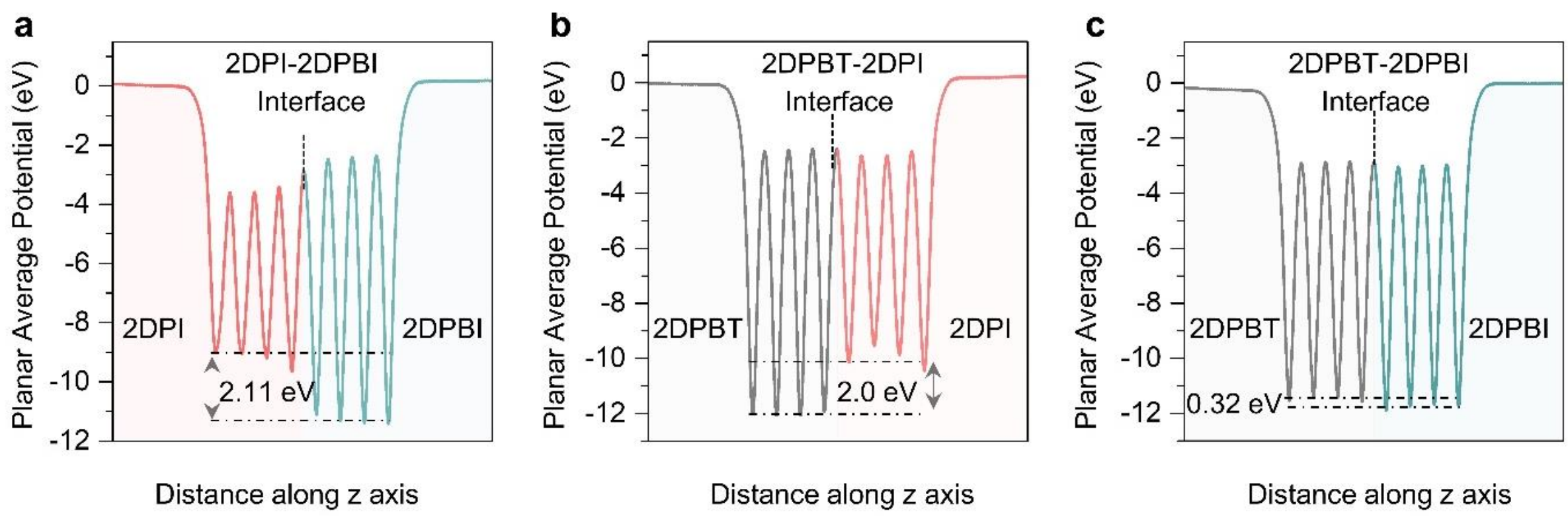

Figure R25 | Planar-averaged electrostatic potentials of lattice-matched 2D polymer vdW heterostructures. Planar-averaged electrostatic potential profiles along the stacking direction for (a)

2DPI–2DPBI, (b) 2DPBT–2DPI and (c) 2DPBT–2DPBI vdW heterostructures, showing built-in potential differences of 2.11, 2.0 and 0.32 eV, respectively.

Action: The definition and calculation method of the average potential difference have been clarified in the revised manuscript. The values for all three lattice-matched heterostructures are now explicitly discussed in Supplementary Fig. 54.

6. *The SI needs more details on sample preparation, e.g. volume of water, amount of SOS (and method of spreading – in some solvent?) in step I; approximate # of aqueous layer exchanges between steps? Exact conditions (reaction time) for the other 9 heterostructures?*

Response: We sincerely thank the reviewer for this helpful comment. We have now expanded the sample-preparation details in the Supplementary Information for all ten 2D polymer vdW heterostructures. The revised section includes the water volume, surfactant amount, solvent and spreading method used in Step-I, the subphase-exchange procedure between each step, and the exact monomer conditions and reaction times for all heterostructures.

7. *Fig. S9 caption gives 2D unit cell of the self-assembled monomer with reference to "indexed reflections", but no indexing is shown on the figure, and I am not sure what it refers to (the peaks observed in Fig. S9d seem to represent the SOS layer along).*

Response: We sincerely thank the reviewer for pointing this out. The SOS surfactant lattice itself is shown separately in Supplementary Fig. S8, consistent with our previous study (Nat. Commun. 14, 8313, 2023). The indexed reflections in Fig. S9 refer to the SOS–M1 pre-assembly after M1 addition. Fig. S9-d compares the in-situ GIXD pattern measured directly on the water surface after M1 addition with the ex-situ GIWAXS pattern of the transferred SOS–M1 thin film. The peak positions from both measurements agree well and are indexed to the M1 pre-assembled structure, giving a 2D unit cell of a = 13.22 Å, b = 15.29 Å and γ = 85.24°.

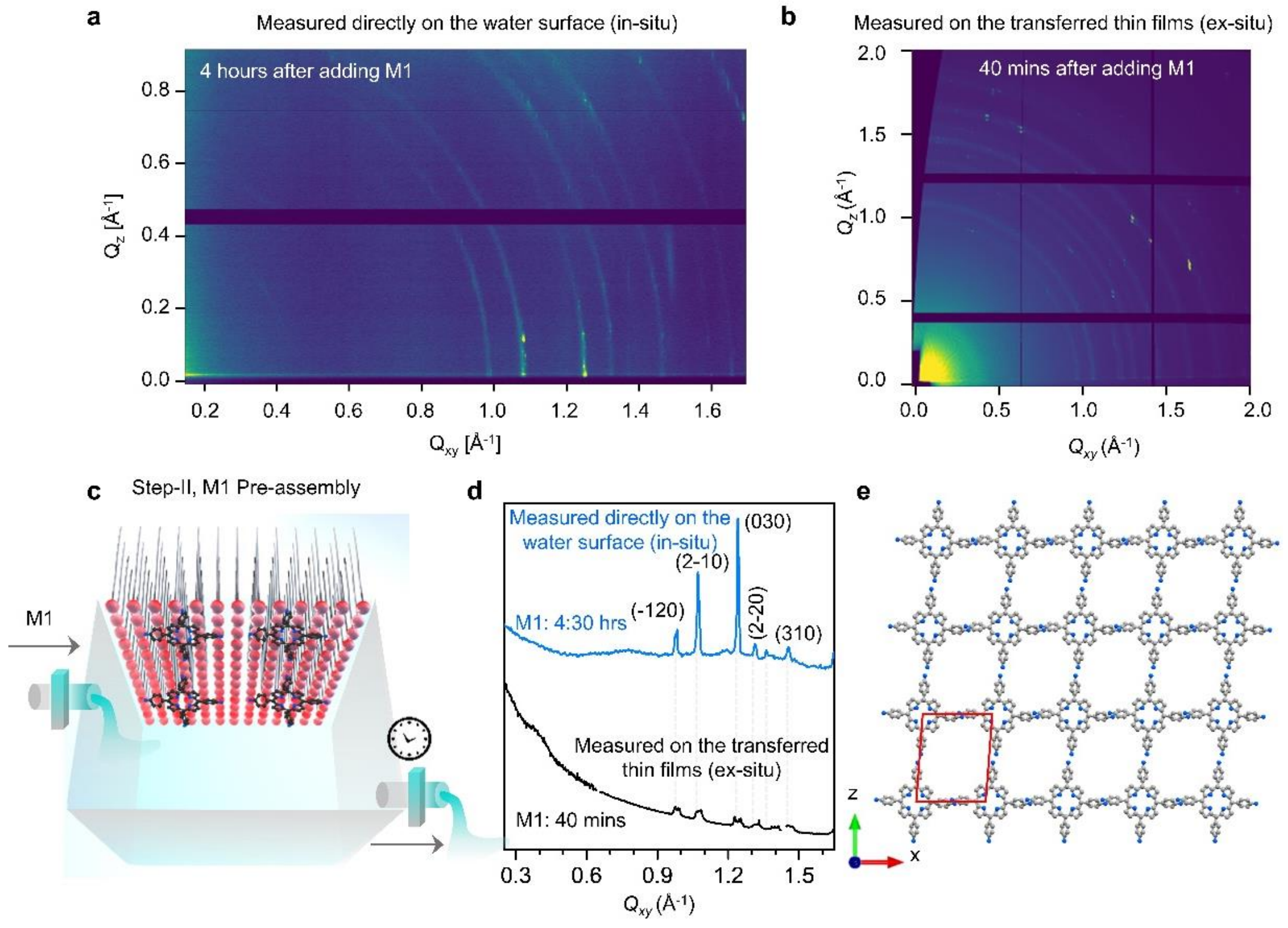

Figure R26 | Pre-organization of M1 during Step-II resolved by in-situ GIXD and ex-situ GIWAXS.

8. Rated to the above: pls, complement the figure with 1D diffraction patterns as it's often impossible to see the discussed diffraction peaks on the 2D GIWAXS images (Figs. 2, S12).

Response: We sincerely appreciate the reviewer's comment. We agree that the original GIWAXS images did not show the weak Bragg reflections clearly because of the contrast scaling. We have therefore provided revised GIWAXS plots with improved contrast, together with 1D line-cut profiles for clearer comparison.

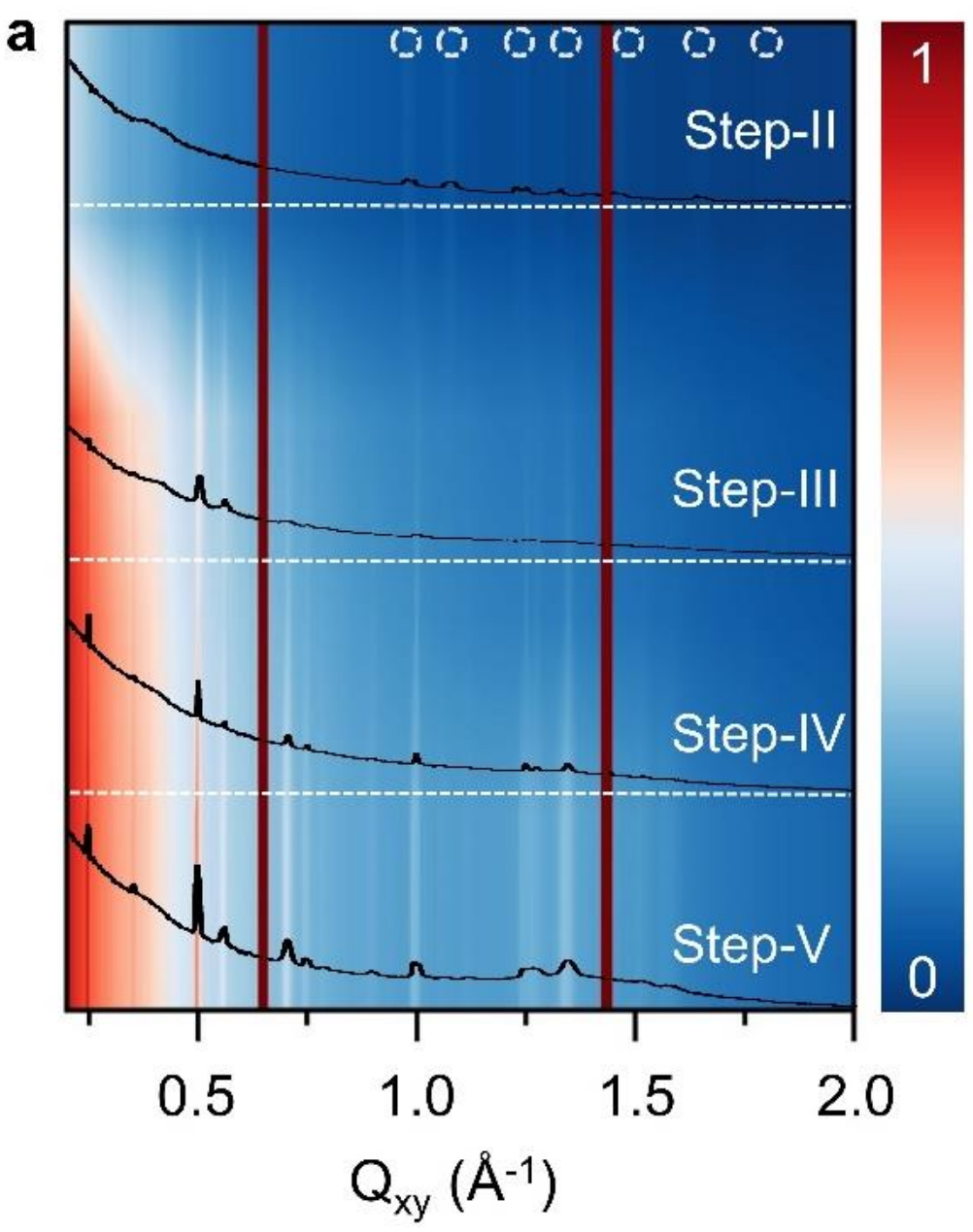

b

(hk)	Meas. peak (Å-1)	Fitted peak (Å-1)
(0, 1)	0.248	0.249
(1,-1)	0.352	0.352
(0, 2)	0.498	0.498
(1,-2)	0.557	0.557
(2,-2)	0.703	0.704
(0, 3)	0.746	0.747
(1,-3)	0.786	0.788
(2,-3)	0.896	0.898
(0, 4)	0.996	0.996
(3,-3)	1.058	1.057
(2,-4)	1.116	1.114
(0, 5)	1.256	1.246
(2,-5)	1.342	1.342
(1,-6)	1.515	1.515
(2,-6)	1.578	1.576

Figure R18 | Layer-by-layer growth analysis by ex-situ GIWAXS. Improved-contrast ex-situ GIWAXS patterns and corresponding 1D line-cut profiles from Step-III to Step-V, showing Bragg reflections at consistent Qxy positions with increasing intensity during sequential assembly.

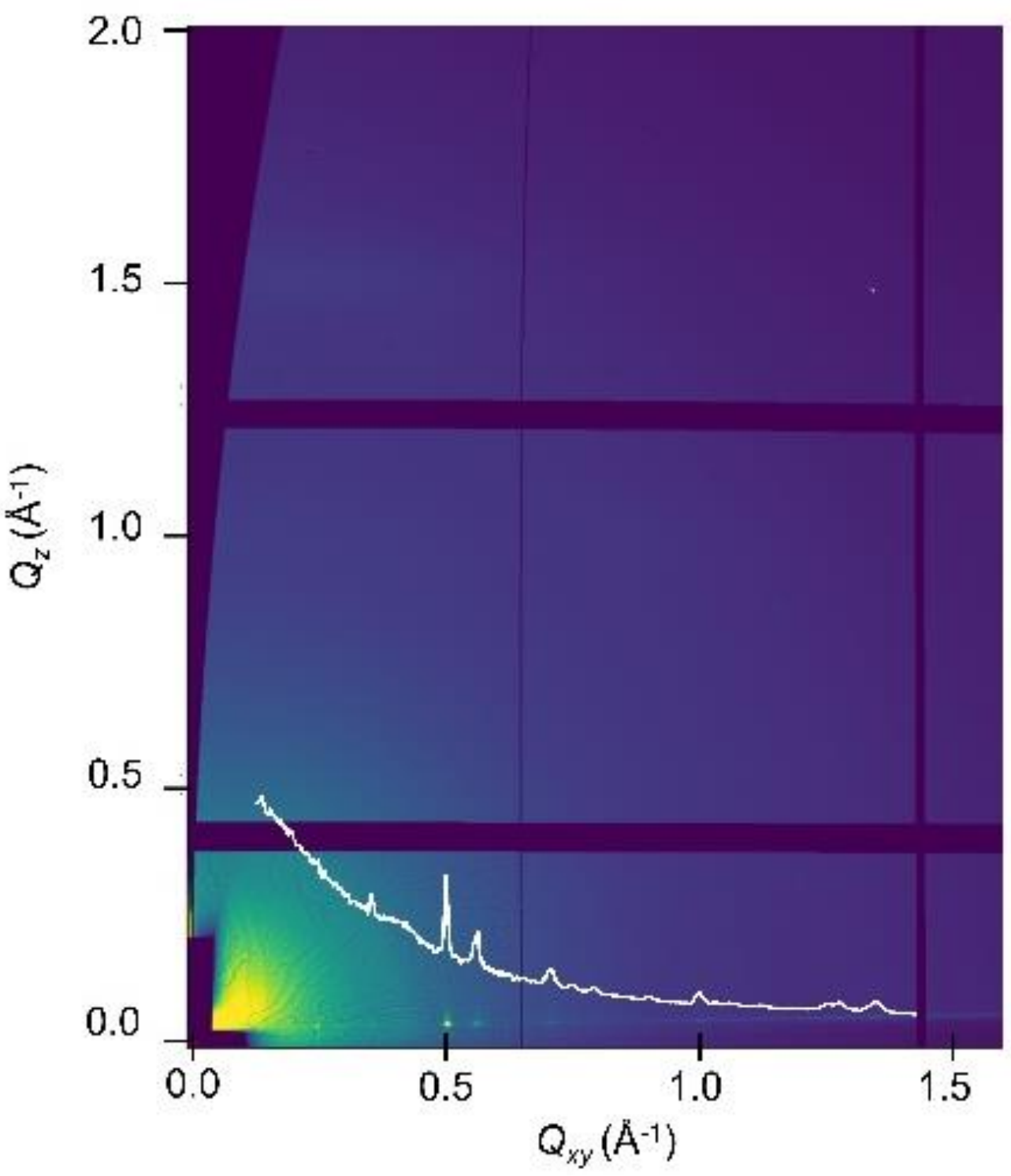

(hk)	Meas. peak [A-1]	Fitted peak [A-1]
(0,1)	0.248	0.250
(0,2)	0.503	0.500
(1,-2)	0.561	0.559
(2,-2)	0.707	0.707
(0,3)	0.752	0.750
(1,-3)	0.792	0.791
(0,4)	1.002	1.000
(2,-4)	1.120	1.118
(1,-5)	1.270	1.275
(2,-5)	1.347	1.346

Figure R18 | GIWAXS analysis of Step-III 2DPI formation. Ex-situ GIWAXS pattern and 1D diffraction profile of the film after M2 addition, showing indexed Bragg reflections of the square 2DPI lattice with a = b = 25.14 Å and γ = 90.00°.

Referee #3 (Remarks to the Author):

General comment: *The manuscript presents an extensive and carefully executed body of experimental work on organic vdW heterostructures based on 2D polymers. The combination of sequential on-water-surface assembly and structural characterization is impressive, and the authors clearly demonstrate a high level of control over layer stacking, lattice registry, and interfacial properties.*

Response: We greatly appreciate the reviewer's positive and insightful comments, which have been invaluable in revising our manuscript. We have carefully addressed all concerns raised by the reviewer, and point-by-point responses are provided below. We hope that the additional efforts made in the revised manuscript appropriately address the reviewer's concerns.

However, despite the breadth and technical sophistication of the study, it remains unclear whether the proposed strategy constitutes a fundamentally new conceptual advance with the potential to significantly reshape the field. In particular, the bottom-up assembly approach on air-water interfaces seems to function primarily as an enabling technique rather than offering new mechanistic insight into interlayer interactions or emergent phenomena unique to organic 2D systems.

Response: We sincerely thank the reviewer for this thoughtful comment. In our approach, the water surface is designed as a dynamic reaction interface. The crystalline surfactant lattice guides monomer pre-assembly, and the subsequent polymerization steps proceed under epitaxial registry. This enables a controlled structural evolution from monomer assembly to 2D polymer formation and finally to layer-by-layer vdW heterostructures with defined lattice matching or mismatching. This control is particularly important for organic 2D crystals, where achieving lattice-matched vdW interfaces at or near zero twist is extremely challenging because the interlayer interactions are weak and non-directional. The present strategy uses surfactant-guided epitaxy to direct the growth of chemically distinct 2D polymer lattices and thereby enabling programmable control over lattice matching and mismatch in 2D polymer vdW heterostructures.

Importantly, this represents the first example of 2D polymer vdW heterostructures with programmable control over lattice matching and mismatching, as also highlighted by Referee #2, who described the work as "first of its kind in 2D polymer materials". The resulting registry control directly affects the interfacial properties. Lattice-matched heterostructures show coherent interfacial charge redistribution, built-in electrostatic potential steps, and strong out-of-plane rectification, whereas no clear rectification is observed in the randomly oriented ex-situ mechanically transferred sample. Thus, the device response arises from programmed interlayer registry rather than from the individual 2D polymers alone.

Our findings therefore establish programmable lattice engineering as a general principle for constructing organic vdW heterostructures, where controlled lattice registry and mismatch provide access to interfacial coupling, band alignment, dipoles, and out-of-plane device functionality that have so far remained largely inaccessible in organic vdW platforms. This level of control positions organic vdW heterostructures as a versatile platform for electronics and future interfacial spin and quantum phenomena.

Experimental approach and key analytic methods are already published by the same authors in "On-water surface synthesis of electronically coupled 2D polyimide-MoS_2 van der Waals heterostructure. Commun Chem 6, 280 (2023)" and "On-water surface synthesis of crystalline, few-layer two-dimensional polymers assisted by surfactant monolayers, Nat. Chem. 11, 994–1000 (2019)". In particular, surfactant-monolayer-assisted interfacial synthesis (SMAIS) use a water surface to pre-organize monomers, creating well-defined 2D polymers with large crystalline domains has been established and the current approach is largely expected.

Response: We sincerely thank the reviewer for this important comment. We agree that our previous work established surfactant-monolayer-assisted interfacial synthesis (SMAIS) for preparing crystalline few-layer 2D polymers on the water surface (Nat. Chem. 11, 994–1000, 2019). However, that work focused on individual 2D polymer films and did not address vdW heterostructure formation. Similarly, in the reported 2DPI–MoS_2 system (Commun. Chem. 6, 280, 2023), the 2D polymer was synthesized on the water surface under Langmuir–Blodgett trough conditions and then transferred onto pre-existing MoS_2 flakes. This produced a 2D polymer–inorganic 2D material heterostructure with random lattice orientation.

In contrast, the present work develops fully organic 2D polymer–2D polymer vdW heterostructures. Here, the water surface is designed as a dynamic interface, where the interfacial charge density is controlled by pH and sequential monomer addition, allowing each monomer-assembly and polymerization step to proceed under surfactant-guided epitaxial registry. This enables programmable control over lattice matching and mismatching across a broad range of organic 2D polymer lattices. Therefore, the key advance of the present work is not the use of SMAIS alone, but its deployment for programmable lattice engineering of all-organic 2D polymer vdW heterostructures. Controlled lattice-matched and lattice-mismatched organic 2D polymer vdW heterostructures have not been established so far in the literature. In contrast to randomly oriented vdW heterostructures, such registry-controlled interfaces exhibit distinct interfacial coupling, built-in dipoles, and out-of-plane device functionality.

Action: To better clarify the key groundbreaking features of our study, we have added the following sentences to the Introduction of the main text, page 4:

“Here, we introduce a bottom-up route to organic van der Waals heterostructures in which chemically distinct crystalline 2D polymers are stacked with defined lattice registry and near-zero twist, and where the lattice mismatch between layers (0%, 1.6%, 16.6%, and 18.0%) is deliberately programmed at the molecular level. In contrast to our previous SMAIS studies that focused on single 2D polymer layers[44] or organic–inorganic hybrids[45], the present work realizes all-organic 2D–2D heterostructures with tunable lattice mismatch and directly correlates lattice registry with interfacial band alignment and vertical diode behavior.”

The authors synthesized and performed in situ structural analysis of the 2D polymers sequentially (Step I to Step V) on the water surface. This reviewer assumes that thermal annealing was carried out prior to device measurements of final organic vdW heterostructures to remove residual or trapped water, which is not described in either the main text or the Supporting Information. What is the effect of water molecules bound to the polymer backbones on lattice parameters and the degree of lattice mismatch in these 2D polymer assemblies?

Response: We sincerely thank the reviewer for this important comment. We have now clarified the sample-drying procedure in the revised Supporting Information. Before device fabrication and electrical measurements, the transferred heterostructure films were thermally annealed in a vacuum oven at 80 °C for 6 h to remove residual surface or trapped water. We note that the structural evolution was first monitored directly on the water surface by in-situ GIXD and the extracted in-plane lattice parameters are consistent with those obtained later on via GIWAXS measurements after film transfer and drying. This indicates that the water surface does not measurably change the covalent in-plane lattice parameters. This is further supported by depth-profile XPS. During etching from the top 2DPI region to the bottom 2DPBI region, the O 1s signal disappears in the 2DPBI region, while C 1s and N 1s remain. At deeper etching cycles, O 1s reappears together with Si 2p/Si 2s, arising from the SiO_2/Si substrate. This elemental evolution supports the absence of trapped oxygen-containing water species at the interface and confirms a chemically sharp 2DPI–2DPBI vdW heterostructures interface.

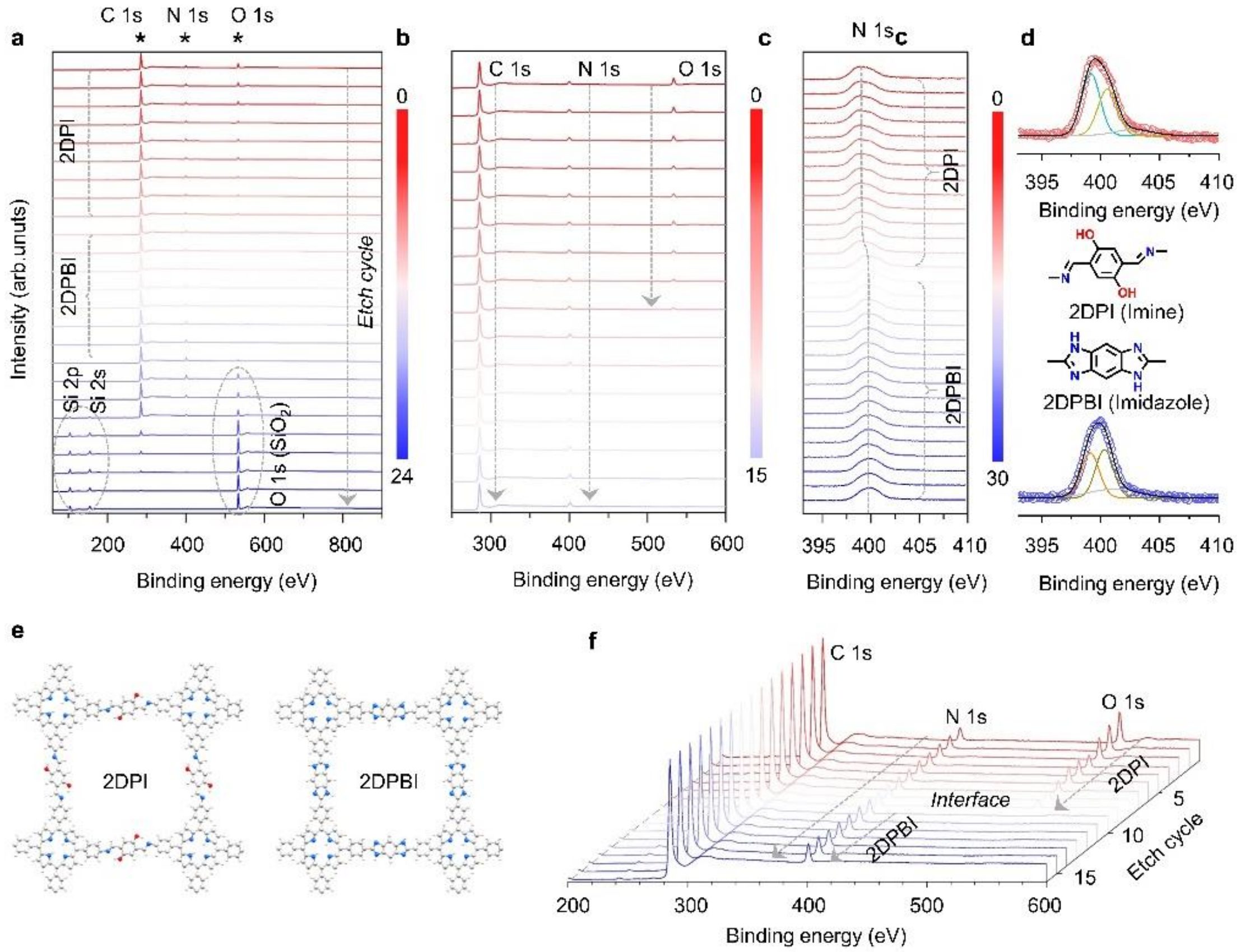

Figure R19 | XPS depth-profile analysis of the 2DPI–2DPBI vdW heterostructure. (a) Full XPS survey spectra collected along the etching depth. (b) Enlarged survey spectra showing the evolution of C 1s, N 1s and O 1s signals. (c) High-resolution N 1s spectra along the depth profile. (d) Deconvoluted N 1s spectra from the top 2DPI region and bottom 2DPBI region. (e) Chemical structures of 2DPI and 2DPBI. (f) 3D representation of the survey spectra in (b), highlighting the transition across the 2DPI–2DPBI interface.

Action: The vacuum thermal-annealing condition used prior to device measurements (80 °C for 6 h) has been included in the Methods section. Figure R19 has been added to the revised Supplementary Information as Supplementary Fig. 35.

The authors identify three commensurate, lattice-matched pairs (2DPI–2DPBI, 2DPI–2DPBT, and 2DPBT–2DPBI), which raises some concerns. Polybenzimidazole (PBI) is known to be hygroscopic, whereas polybenzothiazole (PBT) is comparatively hydrophobic, and both are grown on polyimine (PI), which exhibits intermediate water affinity. It is therefore unclear how epitaxial growth on a water surface can proceed in a commensurate manner despite such differences in water uptake behavior. How can the in situ GIWAXS data (wet state) be in good agreement with the HR-TEM results obtained in the dry state? Residual water could act as a dopant, enhance charge transfer, or induce swelling, all of which may significantly alter the intrinsic electronic or semiconducting properties of these materials.

Response: We sincerely thank the reviewer for this important comment. We would like to clarify that the heterostructures are not formed by stacking pre-existing hydrophilic or hydrophobic polymer films on the water surface. Instead, all heterostructures are synthesized by a bottom-up in-situ sequential assembly process. In each step, the corresponding monomer is dissolved in the aqueous subphase under suitable acid/base conditions, adsorbs at the charged surfactant/interface, and assembles according to the epitaxial relationship imposed by the surfactant head group or the previously formed 2D polymer lattice, as illustrated in Fig. 1b, steps I–V. Therefore, the commensurate growth is governed primarily by interfacial charge-assisted monomer assembly and lattice registry, rather than by the macroscopic water affinity of the final polymer backbone. The use of anionic SOS and cationic CTAB allows different monomers to be selectively guided at the water surface through electrostatic interaction and surfactant-confined pre-assembly. If water uptake or hydrophilicity dominated the process, we would expect significant lattice distortion or loss of registry. Instead, the in-situ GIXD/GIWAXS data measured directly on the water surface and the HR-TEM/SAED data obtained after transfer and drying yield consistent in-plane lattice parameters.

As shown in Supplementary Fig. 9, the in situ GIWAXS profiles are fully isotropic, indicating that the pre-assembled M1 layer is randomly oriented and only becomes ordered upon polymerization. This appears inconsistent with the schematic illustrations throughout the main text and Supplementary Figs. In fact, Figure 2d suggest that the M1 pre-assembly is already organized via point-on-line coincidence, with polymerization represented simply by adding connecting lines without altering the monomer arrangement. This results in a discrepancy between the experimental observations and the schematic representations. Specifically, it is unclear how a randomly pre-assembled M1 layer can transform into an ordered M2 array through polymerization. This is not related to a commensurate epitaxial interface, as the high glass transition temperature of conjugated polymers severely limits chain mobility, making structural rearrangement extremely slow.

Response: We appreciate the reviewer for this important comment. The ring-like GIWAXS feature of the Step-II M1 assembly actually reflects an azimuthal distribution of many small crystalline domains over the macroscopic X-ray beam area, rather than molecular-level disorder within each domain. In GIWAXS as employed here, the diffraction signal is averaged over a large area on the water surface, typically on the scale of a few centimeters (~2 cm). In Step-II, the SOS–M1 assembly is mainly a monolayer/few-layer pre-assembly with relatively low scattering density. Therefore, even if each local domain is ordered, the presence of many differently oriented domains within the large GIWAXS footprint can produce ring-like diffraction features. Specifically, we believe that on the macroscopic scale (μm - mm) irregularities in the M1 sub-phase film are mechanically introduced during to the injection of M1 and vibrations, lead to many wrinkles or folds across the cm-long beam footprint.

In contrast, HRTEM probes local nanoscale regions. The transferred SOS–M1 and CTAB–M3 assemblies show well-resolved square molecular lattices over local regions, confirming local crystalline ordering in Step-II. Therefore, the cartoons represent the local ordered monomer arrangement derived from HRTEM, whereas the GIWAXS rings reflect the macroscopic orientational distribution of such ordered domains over the large beam area. To further support Step-II pre-assembly, AFM measurements of the transferred SOS–M1 assembly show thicknesses of 2.56 nm after 30 min and 2.61 nm after 10 h. These values match the combined thickness of the SOS monolayer and M1 molecule, supporting monolayer/few-layer M1 pre-assembly beneath the SOS surfactant monolayer.

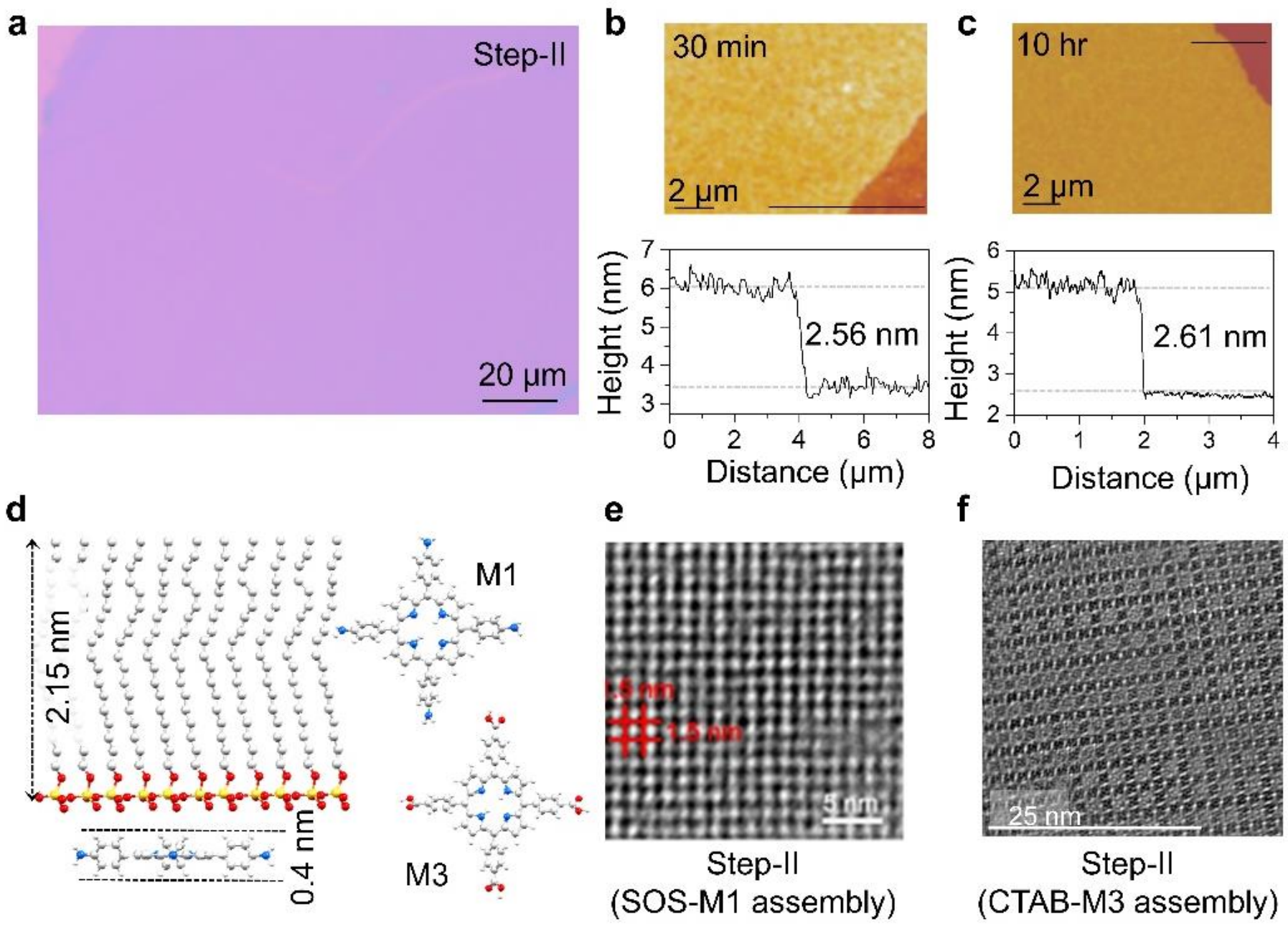

Figure R12 | Step-II monomer pre-assembly underneath charged surfactant monolayers. (a) Optical microscopy image of the SOS–M1 assembly formed in Step-II. (b,c) AFM images and height profiles of the SOS–M1 assembly after M1 adsorption for 30 min and 10 h, showing thicknesses of 2.56 and 2.61 nm, respectively. (d) Schematic thickness comparison of the SOS monolayer and M1/M3 molecules. (e) HRTEM image of the SOS–M1 assembly in Step-II, adapted from ref. 45 (Nat. Chem. 11, 994–1000, 2019). (f) HRTEM image of the CTAB–M3 assembly in Step-II.

To clarify the driving force for the transition from point-on-line to line-on-line epitaxy, we analyzed three representative square-lattice 2D polymer systems with different lattice parameters, using both anionic SOS and cationic CTAB surfactants.

Case-I: 2DPI formation with anionic SOS surfactant.

For 2DPI (a = b = 2.50 nm, γ = 90°), the Step-II epitaxial alignment corresponds to point-on-line epitaxy, in which M1 lattice points register along the (1,0) lattice lines of the SOS surfactant lattice. After M2 addition in Step-III, the lattice *expands* from the pre-assembled M1 structure (a = 13.22 Å, b = 15.29 Å, γ = 85.24°) to the square 2DPI lattice (a = b = 25.1 Å, γ = 90°). This lattice expansion

changes the epitaxial relationship to line-on-line epitaxy (defined as coincidence of nonprimitive reciprocal lattice vectors of the adsorbate and the substrate), where the 2DPI [6,3] lattice lines coincide with the SOS [1,1] crystallographic lines.

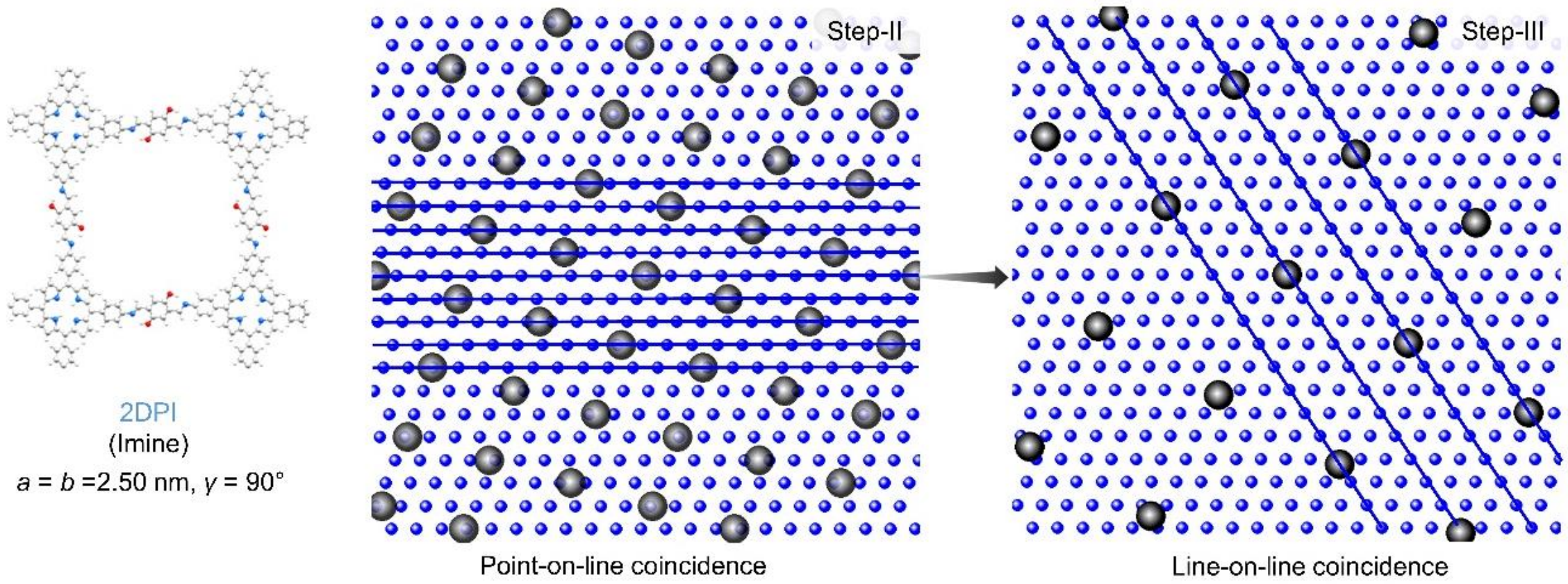

Figure 13 | Epitaxial transition in on-water surface synthesis of 2DPI. Schematic illustration of point-on-line coincidence during M1 pre-assembly in Step-II and line-on-line coincidence during 2DPI formation in Step-III.

Case-II: 2DPID formation with anionic SOS surfactant.

For 2DPID (a = b = 3.00 nm, γ = 90°), the Step-II epitaxial alignment also corresponds to point-on-line epitaxy, in which M1 lattice points register along the (1,0) lattice lines of the SOS surfactant lattice. After addition of M7 (perylene-3,4,9,10-tetracarboxylic dianhydride) in Step-III, the lattice expands from the pre-assembled M1 structure (a = 13.22 Å, b = 15.29 Å, γ = 85.24°) to the square 2DPID lattice (a = b ≈ 30.0 Å, γ = 90°). The epitaxial relationship then evolves into a line-on-line epitaxy, where extended 2DPID [-7,2] lattice lines coincide with the SOS [1,0] crystallographic lines.

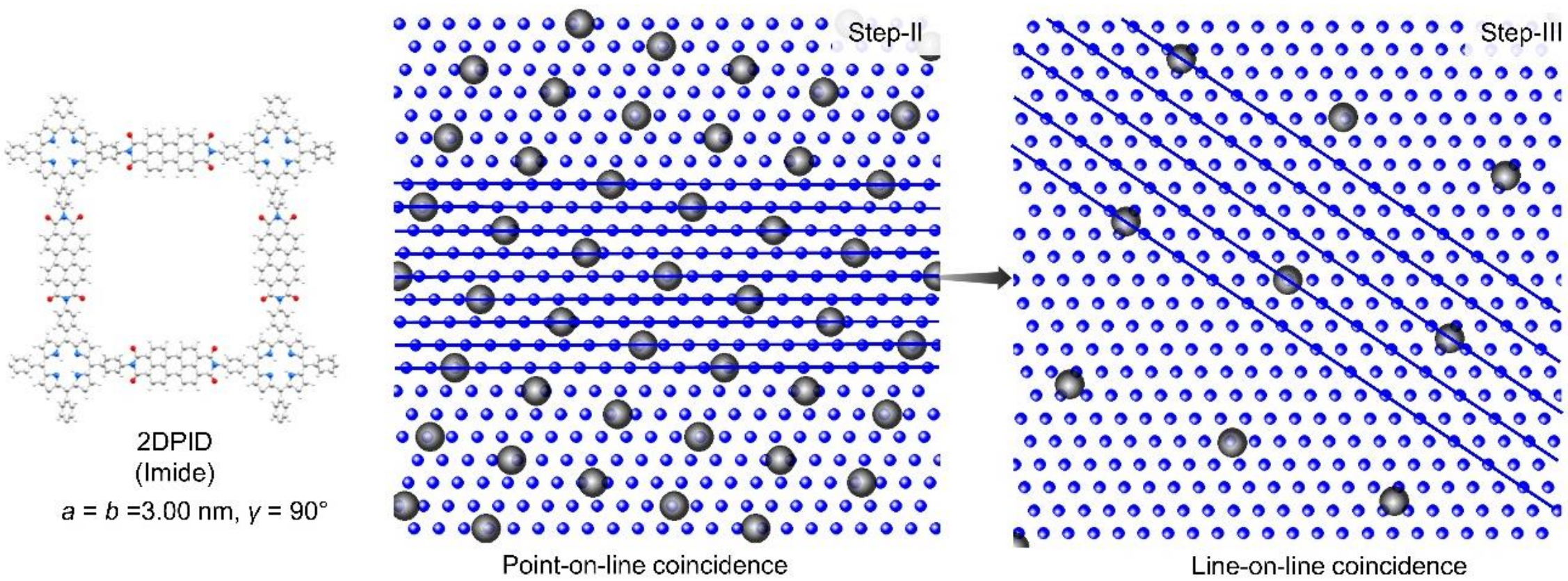

Figure 14 | Epitaxial transition in on-water surface synthesis of 2DPID. Schematic illustration of point-on-line coincidence during M3 pre-assembly in Step-II and line-on-line coincidence during 2DPBI formation in Step-III.

Case-III: 2DPBI formation with cationic CTAB surfactant.

For 2DPBI (a = b = 2.50 nm, γ = 90°), the Step-II epitaxial alignment corresponds to point-on-line epitaxy, in which M3 lattice points register along the (0,1) lattice lines of the CTAB surfactant lattice. After M4 addition in Step-III, the lattice expands from the pre-assembled M3 structure (a = 17.53 Å, b = 17.49 Å, γ = 89.40°) to the square 2DPBI lattice (a = b = 25.1 Å, γ = 90.00°). The epitaxial relationship then evolves into line-on-line epitaxy, where extended 2DPBI [3,–6] lattice lines coincide with the CTAB [1,1] crystallographic lines.

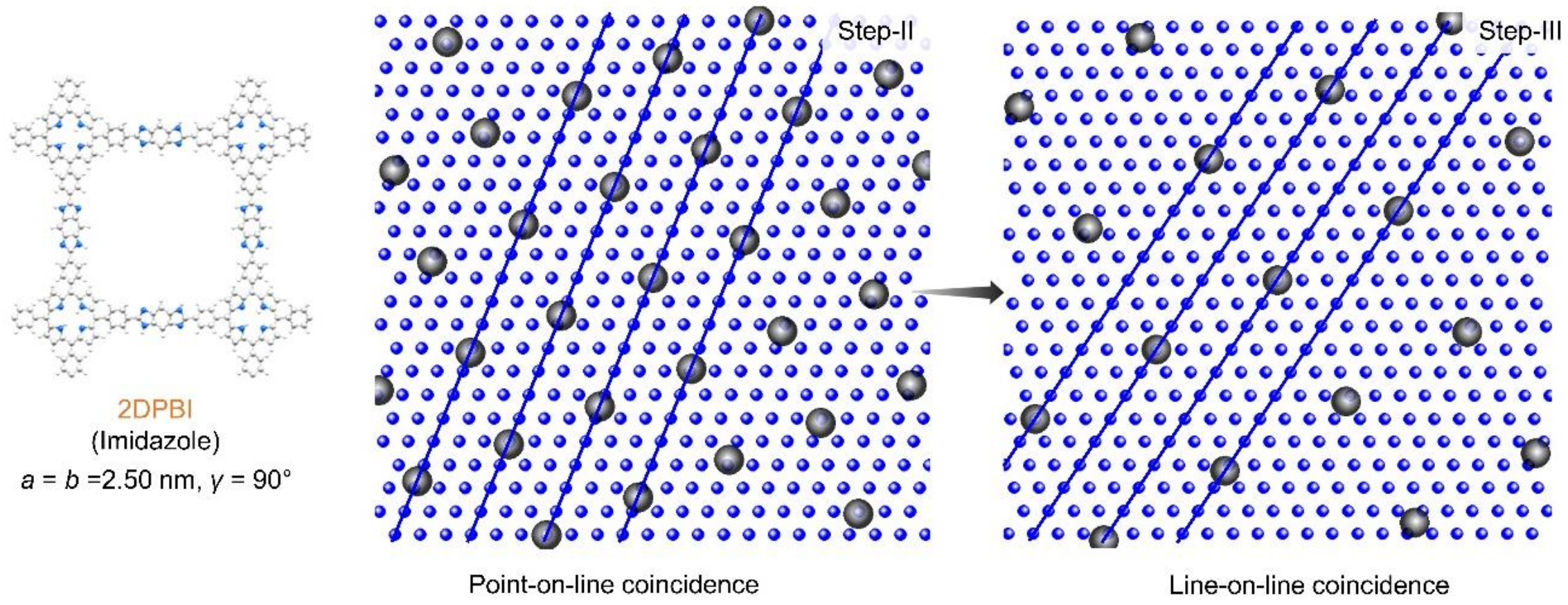

Figure 15 | Epitaxial transition in on-water surface synthesis of 2DPBI Schematic illustration of point-on-line coincidence during M1 pre-assembly in Step-II and line-on-line coincidence during 2DPID formation in Step-III.

Epitaxial growth is fundamentally driven by the minimization of total system energy, i.e. it depends on the balance between interfacial energy and the elastic strain required to match the interacting lattices. In our system, we would characterize the driving force for the transition from point-on-line to line-on-line epitaxy to be the dramatic lattice expansion that occurs during covalent polymerization (Step-III). During Step-II, the discrete monomer assemblies maintain a point-on-line registry with the charged crystalline surfactant. However, as the second monomer is added and covalent bonds form, the resulting 2D polymer lattice expands, and the much larger lattice will establish an epitaxial relationship that again minimizes energy (or none if none is available) – in our cases adopting line-on-line epitaxy in order to minimize the overall energy for the newly expanded lattice.

Since line-on-line epitaxy is the most general (every point-on-line lattice is thus also line-on-line), it therefore also represents the lowest degree of epitaxial matching, and it is possibly more common in systems where the elastic energy to distort the involved unit cells to higher degrees of epitaxy would exceed any potential gain in interface energy. However, because the epitaxial match in our systems involves soft crystals growing on soft templates, establishing a strict, quantitative energetic hierarchy would require comprehensive energy calculations; however, the qualitative driving force is clearly the strain-induced relaxation of the expanded polymer.

Regarding universality, we would state that this mechanism cannot be considered a general rule for all monomers or lattice systems. The preferred epitaxial state depends sensitively on the exact balance of intra- and interlayer interactions—specifically, the elastic moduli of the two lattices and their interlayer interaction potentials. If, for instance, a different 2D polymer had a chemical structure and dimensions that by chance allowed a higher degree of lattice match (e.g., commensurate epitaxy) with minimal distortion, yielding a high gain in interfacial energy, one would likely observe commensurate epitaxy. While we consistently observe the transition to line-on-line epitaxy for all square-lattice 2D polymers examined in this study, the exact epitaxial evolution in other systems would thus remain highly dependent on monomer geometry, charge complementarity, surfactant lattice parameters, and the final 2D polymer structure.

Action: Figures R12–R15 have been incorporated into the revised Supplementary Information. Figure R12 has been added as Supplementary Fig. 20, and Figures R13–R15 have been added as Supplementary Figs. 25–27, respectively. The corresponding discussion on the transition from point-on-line to line-on-line epitaxy for the 2DPI, 2DPID and 2DPBI square-lattice systems has also been added to the Supplementary Information. Detailed discussion is provided in Supplementary Figs. 20 and 25–27**.**

In summary, while the study constitutes a technical achievement and presents a comprehensive dataset, the authors should more clearly articulate its broader conceptual impact on the field.

Response: We sincerely thank the reviewer for this valuable comment. vdW heterostructures assembled from inorganic 2D materials have emerged as a versatile platform for exploring interlayer-coupled phenomena and for tailoring electronic and optoelectronic functionalities (Nature 499, 419-425 (2013), Nat. Rev. Mater. 1, 16042 (2016), Science 367, 537-542 (2020), Nature 645, 906–914 (2025), Science 391, eadz1655 (2026)). By contrast, analogous heterostructures based on organic 2D crystals—including 2DPs, their multilayer-stacked analogues 2D COFs, and coordination-bonded 2D MOFs—have remained largely inaccessible. The central challenge is yet the synthesis: because interlayer interactions in these systems are weak and non-directional, achieving defined **lattice registry at or near zero twist is exceptionally difficult** (Nat. Rev. Methods Primers 3, 1 (2023), Nat. Rev. Mater. 10, 147-166 (2025)). Here, we overcome this long-standing challenge through a bottom-up synthetic strategy based on sequential on-water-surface assembly, which enables layer-by-layer stacking of chemically distinct 2D polymers with defined lattice registry, stacking sequence, and few-layer thickness (~5–10 layers), yielding both lattice-matched and controlled lattice-mismatched heterostructures.

Therefore, we would like to highlight here the key groundbreaking features of our study:

1. Making the first vdW heterostructures accessible for organic 2D crystal materials at zero twist. We introduce a programmable bottom-up route that brings organic 2D lattices into a defined registry, addressing the long-standing synthetic challenge of forming well-defined, lattice-matched vdW heterostructures in organic 2D crystals (2DPs, 2D COFs, and 2D MOFs), where weak, non-directional interlayer interactions otherwise frustrate lattice registry.

2. A new lattice-control knob: programmable lattice matching and mismatching (0–18%) in organic 2D vdW heterostructures. Using in-situ sequential assembly on the water surface, we realize layer-by-layer stacking with controlled registry and create ten organic 2D vdW heterostructures from five structurally and electronically distinct 2DPs. This enables a systematic comparison of fully lattice-matched, near-matched, and strongly mismatched vdW interfaces. GIWAXS measurements and HRTEM reveal epitaxial growth with commensurate alignment and chemically sharp interfaces in all lattice-matched 2DP vdW heterostructures.

3. Designed interfacial electronic coupling, band alignment, and dipole formation at a chemically sharp vdW interface. Commensurate epitaxy and sharp interfaces enable strong interfacial electronic coupling and allow interfacial design to directly program band alignment, dipole alignment, built-in electric fields, and interfacial potential steps (0.3–2.1 V), together with ultrafast interfacial charge separation and photoluminescence quenching of up to 90%.

4. Record vertical rectification controlled by lattice registry. Lattice-matched heterostructures deliver diode-like rectification ratios >10^7 , and this rectification systematically decreases as the lattice mismatch increases, proving that lattice engineering is a quantitative control knob for vertical transport in organic vdW heterostructures. This rectification performance surpasses that of previously reported metal oxide-based (~10^2–10^4) and molecular diode devices (~10^2–10^5).

Overall, we believe our work will be of broad interest across the fields of 2D materials, organic 2D crystals (2DPs/2D COFs/2D MOFs), vdW heterostructures, and organic opto-electronics. Our findings establish programmable lattice engineering as a general principle for constructing organic vdW heterostructures, where controlled lattice registry and mismatch enable access to interfacial coupling, band alignment, dipoles, and out-of-plane device functionality that has so far remained largely inaccessible in organic vdW platforms. This level of control over lattice matching and mismatching positions organic vdW heterostructures as a versatile platform for electronics and emergent interfacial spin and quantum phenomena.

Action: To better clarify the key groundbreaking features of our study, we have added the following sentences to the Introduction of the main text, page 4:

“Here, we introduce a bottom-up route to organic van der Waals heterostructures in which chemically distinct crystalline 2D polymers are stacked with defined lattice registry and near-zero twist, and where the lattice mismatch between layers (0%, 1.6%, 16.6%, and 18.0%) is deliberately programmed at the molecular level. In contrast to our previous SMAIS studies that focused on single 2D polymer layers[44] or organic–inorganic hybrids[45], the present work realizes all-organic 2D–2D heterostructures with tunable lattice mismatch and directly correlates lattice registry with interfacial band alignment and vertical diode behavior.”

Point-to-point response to the comments from the reviewers

Referee #1 (Remarks to the Author):

The authors have carried out extensive additional experiments and provided satisfactory responses to all of my previous concerns. The revised manuscript has been significantly improved and now presents a compelling study of organic vdW heterostructures. I therefore recommend its publication in Nature.

Response: We greatly appreciate the reviewer for the highly positive comments with recommendation for publication.

Referee #2 (Remarks to the Author):

The authors have amde a major effort in revising the manuscript. All significant issues raised in the original set of reviews have been well-addressed. I recommend accepting the revised manuscript in its current form. Congratulations to the authors on this significant breakthrough in the field.

Response: We greatly appreciate the reviewer for the highly positive comments with recommendation for publication.

Referee #3 (Remarks to the Author):

The manuscript presents an extensive and carefully executed body of experimental work on organic van der Waals (vdW) heterostructures based on 2D polymers. The combination of sequential on-water surface assembly and structural characterization is highly compelling, and the authors demonstrate a remarkable level of control over layer stacking, lattice registry, and interfacial properties.

In particular, the study advances the development of fully organic 2D polymer–2D polymer vdW heterostructures under surfactant-guided epitaxial registry. This approach provides programmable control over lattice matching and mismatching across a wide range of organic 2D polymer systems.

This reviewer appreciates the authors' thoughtful point-by-point responses to the comments raised and finds that both the quality and clarity of the manuscript have been significantly improved. Accordingly, this reviewer supports its publication in Nature.

Response: We greatly appreciate the reviewer for the highly positive comments with recommendation for publication.
